# Structure–function relationships of donor–acceptor Stenhouse adduct photochromic switches†
†Electronic supplementary information (ESI) available: Detailed synthetic, spectroscopic, photoswitching, kinetic data, X-ray structures and DFT modelling. CCDC 1839144–1839152. For ESI and crystallographic data in CIF or other electronic format see DOI: 10.1039/c8sc03218a


**DOI:** 10.1039/c8sc03218a

**Published:** 2018-09-12

**Authors:** Neil Mallo, Eric D. Foley, Hasti Iranmanesh, Aaron D. W. Kennedy, Ena T. Luis, Junming Ho, Jason B. Harper, Jonathon E. Beves

**Affiliations:** a School of Chemistry , UNSW Sydney , High St, Kensington , Sydney , NSW , Australia . Email: j.beves@unsw.edu.au

## Abstract

The first in-depth, systematic study of the photoswitching properties of Donor–Acceptor Stenhouse Adducts (DASAs) is reported. Barbituric acid derived DASAs functionalised with 14 different amines ranging from dimethylamine to 4-methoxy-*N*-methylaniline were structurally characterised in solution using ^1^H and ^13^C NMR spectroscopy and, in eight cases, in the solid state by single crystal X-ray diffraction. The distribution of coloured and colourless isomers in the dark, their photostationary states under irradiation, apparent thermal half-lives, and fatigue resistance are systematically compared. A simple kinetic model is used to characterise photoswitching behaviour and reveals that minor structural modifications can significantly improve the photoswitching properties of DASA photochromes. These modifications result in excellent photoswitching properties for ‘1^st^ generation’ DASAs in chloroform, including exceptional fatigue resistance, opening the door for these photochromic molecules to find widespread applications.

## Abstract 2

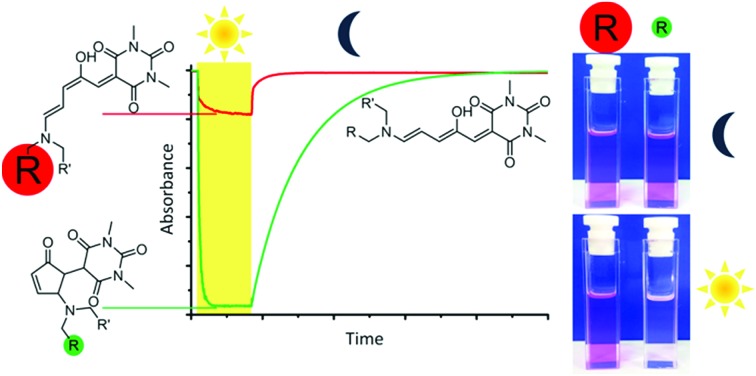
Surprisingly small structural changes in Donor–Acceptor Stenhouse Adducts (DASAs) result in predictable, robust and effective photochromic switches.

## Introduction

Photochromic molecules^1^ can be isomerised between two different forms upon irradiation with light. Those that can be addressed by visible light are particularly appealing^2^ as visible light is generally non-destructive, and selective addressing of the different isomers is straightforward where the two isomers absorb at sufficiently different wavelengths. In addition to the commercial applications such as in sunglass lenses, photochromic molecules allow light to be used to reversibly control molecular properties for the development of light-controllable molecular tools,^3^ controlling energy transfer^4^ or self-assembly,^5^ acting as receptors,^6^ powering molecular machines,^7^ for drug delivery,^8^ or modifying material properties.^9^ Dithienylethene,^10^ spiropyran,^5^,^11^ oxazine^12^ and azobenzene9a,^13^ are classic photochromic molecules and examples exist in all of these groups where visible light can be used to drive the photoisomerisation. More recently, hemithioindigo,^14^ indigos,^15^ heterodiazocines,^16^ arylhydrazones,^17^ azo-BF_2_ derivatives,^18^ imidazolyl-based radicals^19^ and coumarin-dienes^20^ have all demonstrated excellent visible-light switching properties, including examples of biphotochromic systems.^21^

**Scheme 1:**
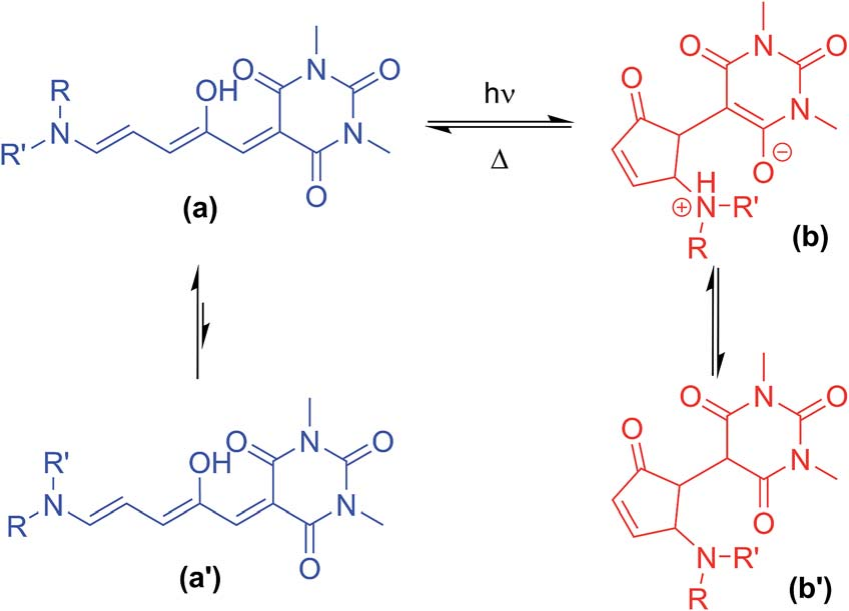
Donor–acceptor Stenhouse adducts based on barbituric acid. These molecules can be isomerised between a coloured linear form (**a**) and a colourless cyclic form (**b**) with visible light. Where unsymmetrical amines are used, two major linear isomers (**a**, **a′**) are present in solution with the most abundant isomer (**a**) having the smaller substituent in the R position. The cyclic isomer exists as either the zwitterionic enolate form (**b**), or the keto form (**b′**), depending on the amine.

Donor–acceptor Stenhouse adducts (DASAs) are a class of reverse photochromic^22^ molecules overlooked^23^ until 2014 24 and are the subject of an insightful recent review.^25^ These DASAs (Scheme 1) can be synthesised in two simple steps from commercially available materials – a major advantage over most other photochromic molecules. Irradiation with visible light can isomerise these DASAs from a coloured linear triene form (**a**, Scheme 1) to a colourless cyclic form (**b**). The mechanism of the isomerisation has been proposed,^26^ and probed computationally^27^ and with transient absorption spectroscopy,^28^ in addition to surface^29^ and gas phase^30^ studies, although much remains to be understood. Recent studies have also investigated the crucial role of the hydroxyl group for photochromic behavior,^31^ and the importance of solvent has been investigated.27*c* The appeal of this new class of photoswitch has led to many applications in the past four years including the detection of amines,^32^ surface patterning,^33^ photo printing,^34^ photoresponsive liquid crystals^35^ and the functionalisation of magnetic nanoparticles.^36^ DASA units have also been installed in polymers,^37^ polymer dots,^38^ and self-assembled polymersome nanoreactors,^39^ with applications as sensors^40^ and for drug release,^41^ and compatibility with orthogonal switches has been demonstrated.^34^,^42^ The introduction of aniline donors^43^ with fused alkyl rings^44^ to control the twist angle between the triene and the aniline ring (so-called ‘2^nd^ generation DASA’^44^) allows the conjugated system to be extended and thereby shift the absorption from the green into the far red.^44^ Tuning the electron donating/withdrawing properties of the aniline donor group can also influence the absorption maximum of DASAs,^43^,^44^ with more electron donating anilines resulting in a red-shift of the absorption, which also influences the relative stability of the linear and cyclic forms.^43^ Despite these studies showing the electronic properties of the donor groups can tune the optical properties of DASAs, to date there has been no systematic study of the photoswitching properties of DASAs (*e.g.* photostationary states, kinetics, fatigue resistance) to understand the parameters required for ideal photoswitching behaviour. Herein we report the first such study, preparing and analysing a series of electronically similar DASAs to reveal factors to allow the widespread use of DASAs, and also demonstrate that simple ‘1^st^ generation’ DASAs are excellent switches in common organic solvents.

## Results and discussion

**Fig. 1:**
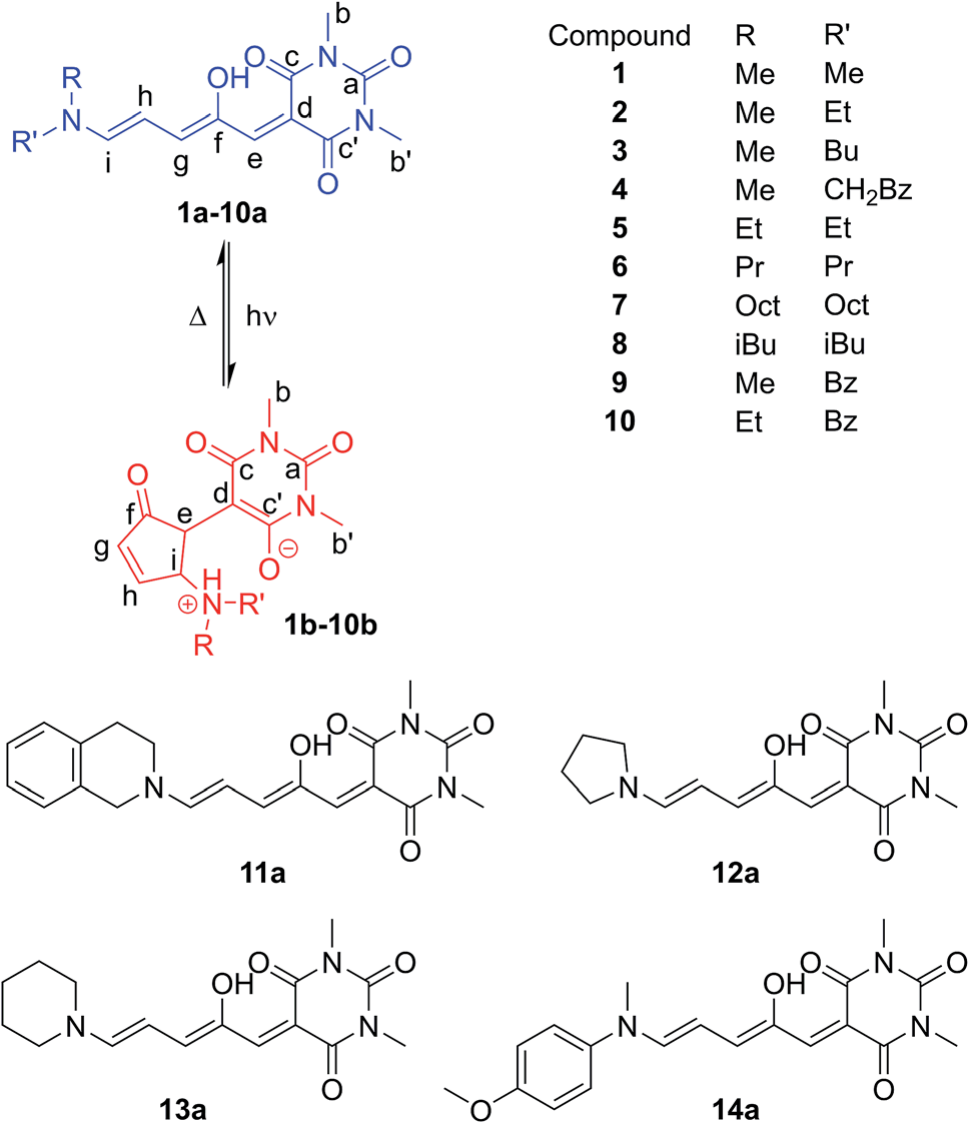
Donor–acceptor Stenhouse adducts (DASAs) in this study, including atom labelling system adopted.

The DASAs studied in this work are shown in Fig. 1, and were studied in chloroform to allow ready comparisons between UV-visible absorption and NMR data. Each of these compounds, except **14**, has a similar absorption maximum in chloroform (*λ*_max_ = 567 ± 3 nm) indicating the electronic transition responsible for driving the isomerisation is essentially identical for the series. DASAs **1–8** are all derived from secondary alkyl amines and were expected to function similarly. Compounds **9–11** are derived from benzyl amine derivatives with lower p*K*_a_ values than the alkyl amines of **1–8**, but nonetheless retain very similar absorption profiles. Compounds **12** and **13** are derived from cyclic alkyl amines with protonated forms having p*K*_a_ values higher than the remainder in the series and are also more conformationally restricted.^45^ Finally, compound **14** is an aniline derivative (‘2^nd^ generation DASA’), derived from an amine with a significantly lower p*K*_a_ value^46^ (and subsequently *λ*_max_ = 588 nm), which might be expected to behave differently to the other DASAs in this study and connects this work with our previous study of aniline derived DASAs.^43^

DASAs **1–14** were prepared through modified versions of reported procedures24a,^43^ for related DASAs and detailed synthetic procedures and characterisation is given in the ESI (ESI 2–17).† Compounds **5–9** (ref. 24*b*) and **11–13** (ref. 24*b*) have been previously reported, but with limited characterisation of their photoswitching properties. Before discussing the photoswitching properties, we will first analyse the structure of these compounds in solution and the solid state. The ^1^H and ^13^C NMR spectra for DASAs **1–14** were collected in both CDCl_3_ and CD_3_CN. The ^1^H and ^13^C NMR signals for all DASAs studied, except for **4** and **14**, follow a predictable pattern with little variation in chemical shifts between compounds (see ESI-1.2, Tables S1 and S2, ESI-18 for details†), allowing confident assignment of linear : cyclic ratios in solution (see below).

The ^1^H NMR spectra of CDCl_3_ solutions of **1–13** taken in the absence of light confirmed the major species in all cases are the linear isomer labelled **a** in Scheme 1. DASAs derived from non-symmetrical amines (*e.g.***2**) exist as mixtures of two major linear isomers (**a**, **a′** – see Scheme 1).^47^^1^H–^1^H NOESY NMR spectroscopy confirmed the most abundant isomer (**a**) has the smallest group oriented perpendicular to the triene chain (R = smaller substituent as shown in Fig. 1, also see ESI-6.4 and ESI-19†). In CDCl_3_ solution in the dark, all compounds have populations of the cyclic isomer (**b**, **b′**, see Fig. 1 for labelling), with the exception of compounds **6–8** where the concentration of the cyclic species is too low (<0.5%) to be determined using NMR spectroscopy.^48^ The observed ratios of the linear (**a** + **a′**) isomers to the cyclic isomers (**b** + **b′**) are shown in Table 1 and indicate the linear and cyclic isomers typically differ in energy by between 1–17 kJ mol^–1^ at room temperature, in line with previous computational work on related DASAs.^49^ The energy difference between the linear and cyclic forms is smaller for DASAs derived from cyclic amines (**11**, **12**, **13**) or aniline derivatives (**14**). Aniline derivative **14** exists as a majority (57%) cyclic form in chloroform in the dark indicating that, even for simple derivatives, the linear form is not always the most thermodynamically stable in organic solutions. Where the cyclic form could be observed in CDCl_3_ it was either as the enolate form (**4b**, **12b**), as the keto form (**5b′**, **9b′**, **10b′**, **11b′**, **14b′**), or as a mixture of both tautomers (**1b**/**b′**, **2b**/**b′**) as evident from both ^1^H and ^13^C chemical shifts, and ^1^H–^13^C HSQC analysis (ESI-19 and ESI-20†). At high concentrations of DASA **5** in solution, the enolate form (**5b**) is favoured over the keto form (**5b′**), possibly due to the formation of hydrogen-bonded dimers (ESI-20.5†). This keto-enolate distribution may be important in determining photoswitching properties in different solvents, and must be considered in any computational work. NMR data were also assigned in the more polar solvent CD_3_CN and these are given in the ESI-19.† These data indicate that the zwitterionic enolate form **b** is more stabilised in CD_3_CN in all cases, with the exception of DASA **4** where both **4b** and **4b′** are equally abundant (ESI-19†).

**Table 1:** Summary of photoswitching properties of DASA compounds **1–14**^*a*^

#^*b*^	R^*c*^	R'^*c*^	Dark equilibrium (CDCl_3_)^*d*^		Light equil.^*e*^	Fatigue recovery/cycle^*f*^ (%)
			% (**a** + **a′**)	% (**b**/**b′**)	% Δ*A*	
**1**	Me	Me	86	14	94	99.7
**2**	Me	Et	95	5	75	99.1
**3**	Me	Bu	97	3	58	99.4
**4**	Me	CH_2_Bz	98	2	86	99.7
**5**	Et	Et	>99.5	<0.5	26	99.1
**6**	Pr	Pr	>99.9	<0.1	18	99.4
**7**	Oct	Oct	>99.9	<0.1	14	99.4
**8**	iBu	iBu	>99.9	<0.1	15	99.5
**9**	Me	Bz	91	9	96	99.9
**10**	Et	Bz	97	3	82	99.7
**11**	Q		74	26	99	99.8
**12**	Pyr		83	17	65^*g*^	99.5
**13**	Pip		60	40	91^*h*^	99.5
**14**	Me	C_6_H_4_OMe	43	57	99	99.9

^*a*^All data in chloroform except where specified.

^*b*^DASA compound number, see Scheme 1 for structures.

^*c*^Me = methyl; Et = ethyl; Pr = propyl; CH_2_Bz = 2-phenylethyl; iBu = isobutyl; Oct = octyl; Bz = benzyl; Pyr = pyrrolidyl; Pip = piperidyl; Q = 1,2,3,4-tetrahydroisoquinoline; C_6_H_4_OMe = 4-methoxyphenyl.

^*d*^Measured by NMR integration. Letters refer to isomers in Schemes 1 and 2. Ratios were not sensitive to water content, see ESI-21.

^*e*^Change in absorption at *λ*_max_ measured by UV-vis absorption on samples with *A* = 0.95 ± 0.05 and irradiated with a 567 nm LED until no further change in absorption was observed, 45 seconds except where stated otherwise. The change in absorption depends strongly on the emission spectrum (see ESI-26.1) and light intensity of the LED.

^*f*^Recovery of absorption after 45 seconds of irradiation with a 567 nm LED, calculated from remaining absorption at *λ*_max_ after 100 cycles. Initial absorbance values were all 0.95 ± 0.05.

^*g*^Compound **12** requires irradiation for ∼5 min to reach PSS during which significant decomposition also occurs so the PSS absorption is calculated relative to the absorption after thermal recovery (data shown). Irradiation for 45 seconds results in a change in absorption of 31%.

^*h*^Compound **13** was slow to reach PSS after ∼90 s. During this irradiation significant decomposition occurs, so the PSS absorption is calculated relative to the absorption after thermal recovery (data shown). After 45 s the decrease in absorption is 82%.

Single crystal X-ray structures of **1b**, **2b**·2H_2_O, **4a**·THF, 2{**9b**}·7H_2_O, **12a**, 2{**12b**}·DCM·1.1H_2_O, **14a**·CDCl_3_ and **14b′** were determined, and structural details are included in the ESI (ESI-32).† The linear structures **4a**·THF and **12a** have very similar bond lengths along the triene core. As previously commented for the related Meldrum's acid derivatives,^43^ the bond lengths along the triene chain indicate alternating long and short bonds consistent with a dominant charge-separated resonance form (ESI-32.10†). The bond lengths of aniline derivative **14a** indicate the bonding along the triene chain is more delocalised than in the alkyl derivatives.

**Fig. 2:**
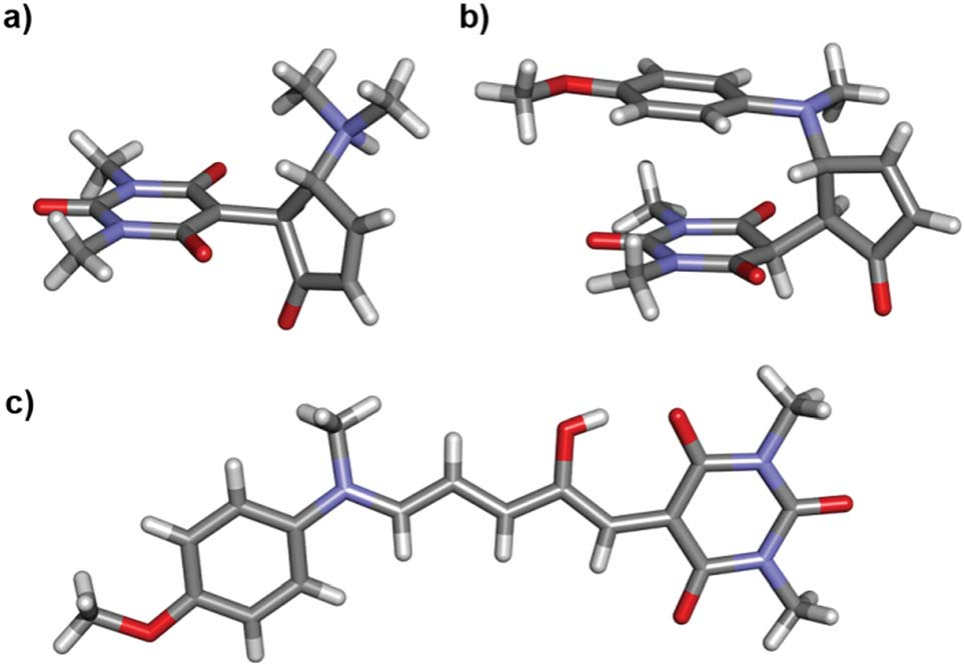
Single crystal X-ray structures of (a) **1b**, (b) **14b′** and (c) **14a**·CDCl_3_. Solvent omitted for clarity.

Of the cyclic isomers, the X-ray structures of **1b**, **2b** and **9b** are structurally similar and are the zwitterionic enolate tautomer (**b**) (Fig. 2). In each case, short intermolecular hydrogen bonds are present between the protonated amine and the oxygen atom of a water molecule or an amide group (N···O = 2.64–2.76 Å) to form dimers (**1b**, **2b**, **12b**), or hydrogen bonded chains bridged by water molecules (**9b**). These intermolecular hydrogen bonds act to stabilise the enolate (**b**) form of **9b**, despite this molecule adopting the keto (**b′**) form in solution, and may also be useful for controlling hydrogen bonding interactions in solution. The structure of **14b′** (Fig. 2) confirms that this molecule adopts the keto tautomer in the solid state as well as in solution, with a short C–H···N contact (C···N 3.528(2) Å; C–H···N 2.60 Å) between adjacent molecules. This molecule also adopts a conformation to position the methoxyphenyl ring and the barbituric acid ring for close stacking, with the centroid of the barbituric acid ring 3.33 Å from the plane of the phenyl ring (ESI-32.9†). The distance between the amide and methoxy methyl groups changes significantly between the **14a** (16 Å) and **14b′** (3.6 Å) isomers (Fig. 2) and could also find applications for mechanical switching.^50^

**Scheme 2:**
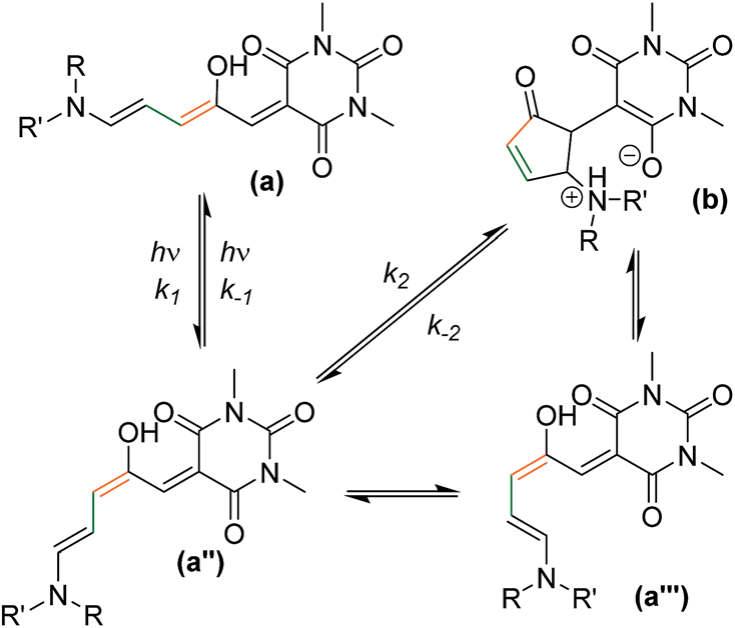
The proposed mechanism of DASA isomerism.^26^ The photo-driven step (*k*_1_/*k*_–1_) involves a double bond isomerisation, which is followed by a bond rotation. The second step involves ring closing and proton transfer. The measured *k*_2_/*k*_–2_ rates are the overall rate from the photoisomer to the closed product, although the mechanism does proceed *via***a′′′**, as previously reported.^26^

A simplified mechanism^26^ for DASA switching is shown in Scheme 2 (see ESI-23.1 for details†). Equilibration between linear (**a**) and cyclic forms (**b**) also occurs in the dark, suggesting a similar mechanism may be responsible for the thermal isomerisation. In this study we will consider the process from the linear isomer(s) (**a** + **a′**) to form intermediate isomer **a′′** to occur with independent rate constants in the dark (*k*d1, *k*d–1) and light (*k*l1, *k*l–1). The ring closing step to give the cyclic form (which we consider directly from **a′′** with rate constants *k*_2_, *k*_–2_)^51^ was expected to be the rate determining step, followed by a final fast proton transfer to give the final cyclic product (**b/b′**). As the bleaching of a DASA involves an actinic step followed by a thermal electrocyclisation, complete bleaching requires both steps to be successful. Under irradiation we are considering the overall change in absorption due to these two steps, which therefore underestimates the photostationary state (PSS)^52^ of the actinic step.

**Fig. 3:**
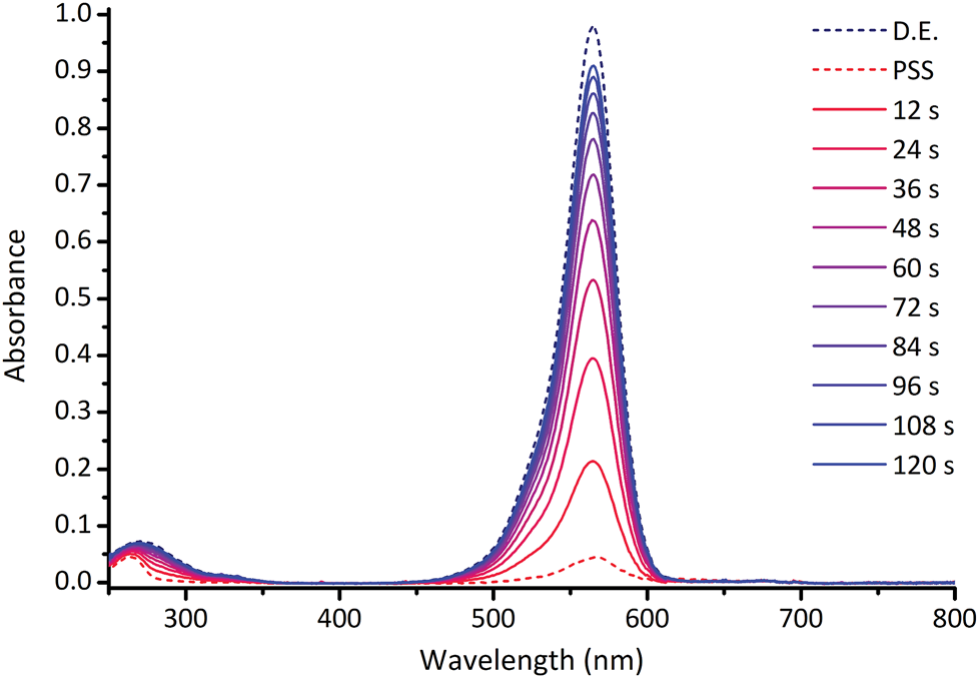
UV-visible absorption spectrum of DASA **1** (CHCl_3_, 298 K) upon irradiation at 567 nm for 60 s, followed by 120 s in the dark. D.E. = dark equilibrium; PSS = photostationary state; times refer to time after the light is switched off. See ESI-27† for equivalent spectra for DASAs **2–14**.

The photoswitching properties of DASAs **1–14** were studied in detail in chloroform solutions (ESI-27†). Fig. 3 shows a typical photoswitching cycle for DASA **1** in chloroform which undergoes a 94% decrease in absorption upon irradiation, followed by rapid equilibration in the dark.

This example demonstrates that, in contrast to previous reports,24b,^28^,^42^,^44^ simple ‘1^st^ generation’ DASAs based on alkyl amines can be excellent switches in polar halogenated solvents like chloroform. Before we present a detailed kinetic approach, three separate and important properties of photoswitches will be considered: the percent decrease in absorption at the photostationary state (PSS),^52^ the fatigue resistance over multiple switching cycles; and the thermal rates of equilibration. The key data is shown in Tables 1 and 2.

**Table 2:** Summary of kinetic data, and predicted photo-thermal distributions of DASA compounds **1–14**^*a*^

	Dark				Light	Predicted at PSS^*c*^		
	*t*_1/2_ ^*b*^/s	*k*_–2_/10^–3^ s^–1^	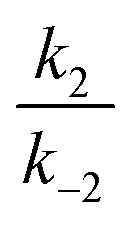	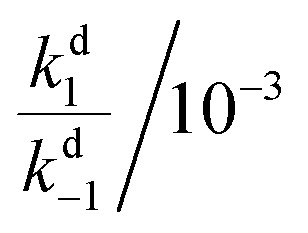	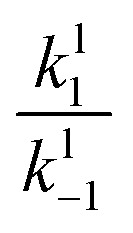 ^*d*^	% **A**	% **I**	% **B**
**1**	32	19	13	13	>100	0	7	93
**2**	20	33	3.8	14	1.5	12	18	70
**3**	13	50	2.1	15	1.0	24	25	51
**4**	10	65	3.6	5.7	>100	0	22	78
**5**	11	58	0.6	8.4	0.5	54	29	17
**6**	6	91	0.2	4.4	0.5	61	32	7
**7**	8	61	0.2	5.2	0.4	66	28	5
**8**	11	49	0.2	4.9	0.5	65	29	6
**9**	29	23	21	4.6	>200	0	4	96
**10**	18	38	4.6	6.7	2.7	6	17	77
**11**	73	7	75	4.7	125	0	1	99
**12**	92	7	3.7	55	0.5	31	15	54
**13**	91	5	31	22	0.6	5	3	92
**14**	265	1	3500	0.38	>150	0	0	100

^*a*^All data from UV-visible absorption in chloroform at 298 K, with photo-thermal equilibrium^52^ generated with a 567 nm LED light source. Rate constant data rounded to two significant figures.

^*b*^Apparent half-life calculated from (ESI-28).

^*c*^Predicted distributions based on kinetic model and the rate constants in this table; **A** = linear isomers (**a**, **a′**); **I** = intermediate (*e.g.***a′′**), **B** = cyclic isomers (*e.g.***b**, **b′**). See ESI-23.2 for description of kinetic model and ESI-24 for individual rate constants. Percentages may not add to 100 due to rounding.

^*d*^Rates *k*l1 and *k*l–1 depend on the light source. While this is consistent for the data in this table, a different light source or different light intensity would generate different values.

For many applications, the most important requirement for a functional switch is complete bleaching at the PSS.^52^ In this regard DASAs **1** (% bleaching at PSS = 94%), **9** (96%), **11** (99%) and **14** (99%) are the best performing photoswitches in this series. However, each of these switches also has a considerable proportion of the cyclic isomer present in the dark: **1** (14% **1b** in the dark), **9** (**9b′** = 9%), **11** (**11b′** = 26%) and **14** (**14b′** = 57%). Similarly, those DASAs with the highest proportion of the linear isomer (**a** in Scheme 1) in the dark also tended to be the poorest switches (*e.g.***7a** : **7b** in the dark is >99 : 1; PSS bleaching 14%), so some trade-off is made in this regard.

**Fig. 4:**
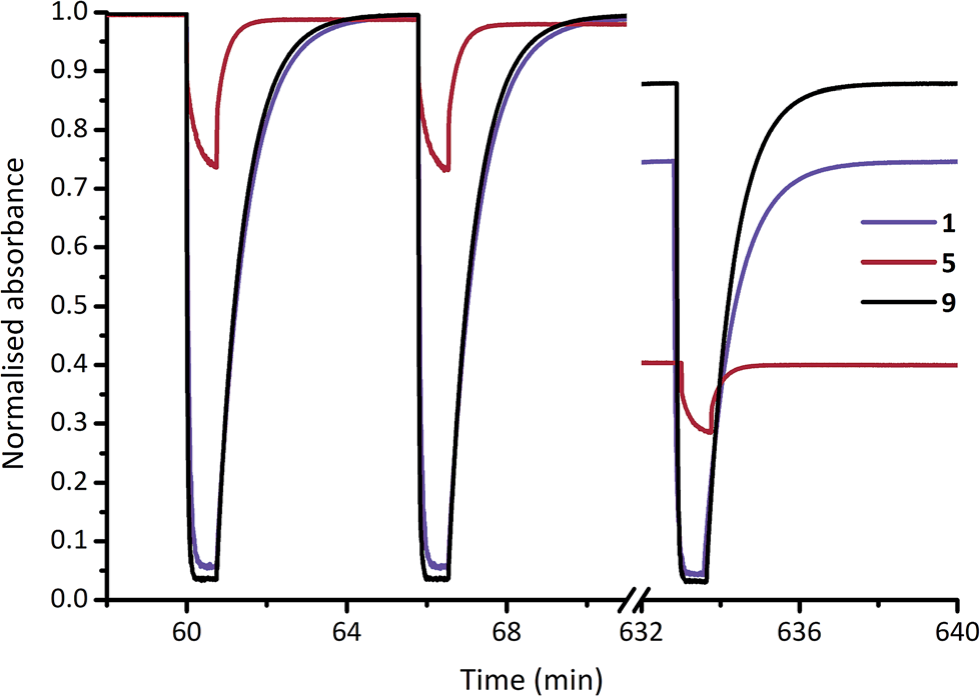
Fatigue resistance of DASAs **1**, **5** and **9** in chloroform. Each cycle is 45 s of irradiation (567 nm LED), and 300 s to equilibrate in the dark. The 1^st^, 2^nd^ and 100^th^ cycles are shown.

For a practical comparison of fatigue resistance, we subjected each of DASAs **1–14** to 100 cycles of irradiation (45 seconds), and dark (5 min) in chloroform (ESI-28†). All DASAs reached PSS during the irradiation time, except for **12** and **13**. The final absorption was used to calculate the fatigue resistance values shown in Table 1, and example data for **1**, **5** and **9** are shown in Fig. 4. The DASAs studied have fatigue resistance comparable to some of the best performing photochromic molecules.10d,11b,14e All DASAs **1–14** recovered over 99% of their initial absorption values each cycle, with the best performing (**9** and **14**) recovering over 99.9% of their initial absorption each switching cycle.

Compounds **2** and **5** have the poorest fatigue resistance—though even here the recovery was 99.1% per cycle—and these are also the poorest switches in terms of PSS^52^ (*i.e.* highest proportion linear at the PSS). This suggests that maximum PSS bleaching may lead to improved fatigue resistance for DASAs. Each of these DASAs were analysed under identical conditions, rather than optimised conditions, and optimisation (*e.g.* more selective irradiation wavelength range, shorter irradiation times) may result in significantly improved fatigue resistance. As a demonstration of robust fatigue resistance, DASA **4** was cycled 1000 times to demonstrate the exceptional fatigue resistance of these compounds, with over 610 cycles performed before 50% decomposition was observed (ESI-28.15†). By comparison, commonly used spiropyrans11*b*—also reverse photochromes—are generally stable for only a handful of switching cycles (*e.g.* 50% degradation after 13 cycles in solution^53^), although immobilising on supports can significantly improve fatigue resistance11*b* (*e.g.* 40% degradation after 50 cycles on polystyrene beads^54^), and removing oxygen can also marginally improve their fatigue performance.^55^ We performed fatigue measurements on DASA **2** in chloroform under an argon atmosphere and also found fatigue resistance significantly improved, from 41% decomposition after 100 cycles under air to just 25% under argon (ESI-28.16†). This suggests photodecomposition of DASAs may also be *via* triplet states.

The apparent half-lives^56^ for the thermal equilibration from PSS^52^ to the dark equilibrium for DASAs **1–14** are shown in Table 2 (see ESI-28† for details). These apparent half-lives range from 6 to 265 s, with the poorest switches (*i.e.* highest proportion of linear isomer at PSS, **3–8**) having the shortest half-lives. Generally, the switches with the greatest change in absorption at PSS had the longest apparent half-lives, as might be expected. Aniline derivative **14** has the longest half-life at 265 seconds, and cyclic amine derivatives **12** and **13** had the next longest half-lives (91 and 92 s). The latter two compounds were also the slowest to reach PSS, with irradiation required for 5 min and 1.5 min respectively, hinting at their unusual behaviour. The behaviour of DASA **4** was also unexpected: this switch has both an improved PSS and shorter thermal half-life compared to the other aliphatic derivatives. The ^1^H NMR data indicates the pendant phenyl ring interacts with the triene unit and this likely provides stabilisation during the isomerisation mechanism, including for intermediates such as **a′′** and **a′′′**. Importantly the behaviour of **4** indicates the potential for optimising PSS without compromising the rate of thermal equilibration.

To investigate the mechanistic causes of the observed photoswitching behaviour, the mechanism shown in Scheme 2 was simplified to a kinetic model involving three species: linear **A**, intermediate **I** and cyclic **B**, details are provide in the ESI (ESI-23.2).†

**Fig. 5:**
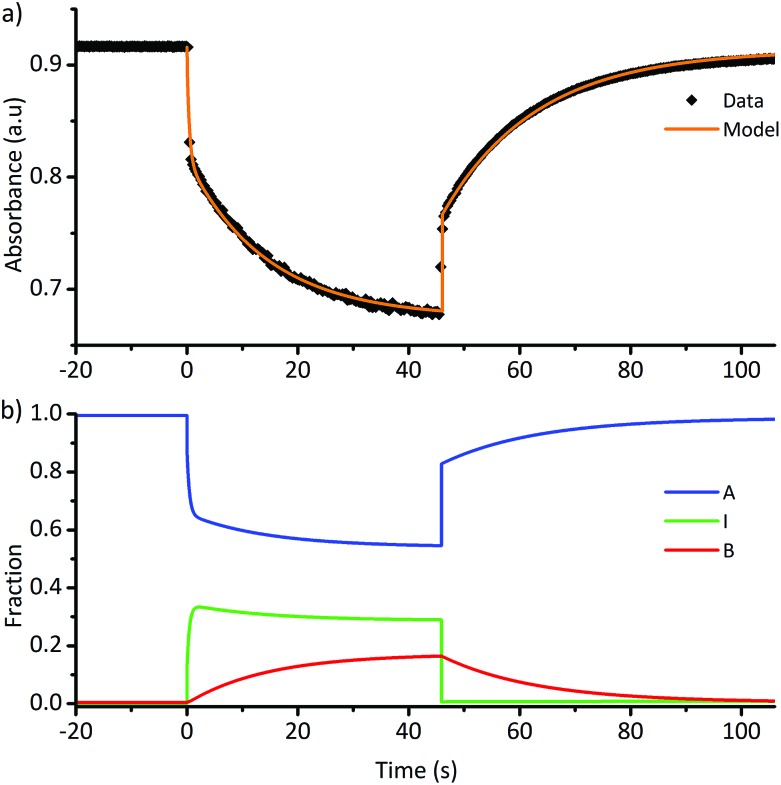
(a) Absorption of DASA **5** (*λ*_max_ = 566 nm in chloroform) during a single photoswitching cycle of 45 s of irradiation with a LED with *λ*_max_ of 567 nm. (b) Fitted populations of **A**, **I** and **B** using data in (a), where **A** = linear isomer; **I** = photoisomer, **B** = cyclic isomer. See ESI-29† for equivalent data for DASAs **1–14**.

An example photoswitching cycle is shown in Fig. 5. This type of profile is typical of DASA switches where a significant proportion of the linear form remains when the photostationary state is reached. This profile is due to the rapid formation of the photoisomer (*e.g.***a′′**), followed by slow equilibration between the cyclic and linear forms. When the light is switched off, the remaining photoisomer (**a′′**) rapidly isomerises back to the linear isomer **a**, followed by slow equilibration between the cyclic and linear forms. We modelled a single switching cycle for each of DASAs **1–14** using numerical integration with a least squares regression analysis (details given in the ESI-24 and ESI-29†).

Table 2 includes key kinetic parameters and allows a quantitative evaluation of the performance of these switches. We will break this discussion into three components: what determines the ratios of isomers at the dark equilibrium and PSS^52^ (thermodynamics); what determines the rates of photo- and thermal equilibration (kinetics); and what structure–function relationships can be established?

In all cases, the rate constant for the first step in the dark, *k*d1, is 2–4 orders of magnitude smaller than that of the reverse step (*k*d–1), indicating the intermediate is just 10–20 kJ mol^–1^ higher in energy than the initial isomer at 298 K. The equilibrium of the ring closing/opening step (*k*_2_/*k*_–2_) is strongly favoured forward for aniline derivative **14** (*k*_2_/*k*_–2_ = 3500), and is favoured forward for all others DASAs studied (*k*_2_/*k*_–2_ = 2–75), except for DASAs **5–8** (*k*_2_/*k*_–2_ = 0.2–0.6). These DASAs **5–8** are indeed the poorest photoswitches studied here, with changes in absorption at the PSS of just 15–26%.

Importantly, DASAs **5–8** also perform poorly in the first step in the light (*k*l1/*k*l–1 = 0.4–0.5), showing the worst switches perform poorly in *both* steps. The most striking observation is that for the best switches the equilibria of this first step in the light (*k*l1/*k*l–1) and the ring closing/opening step (*k*_2_/*k*_–2_) are important in determining the overall photo-thermal performance.

Considering the remaining DASAs, the equilibrium for the first step in the light (*k*l1/*k*l–1) shows that this step is strongly favoured (*k*l1/*k*l–1 ≥ 100) for **1**, **9**, **11** and **14** and these are the switches with amongst the greatest changes in absorption at PSS (94, 96, 99 and 99% bleached respectively). DASA **4** is an overall good switch (PSS 86% bleached) due to a very favourable first step (*k*l1/*k*l–1 > 100), despite a relatively low ratio of ring-closing to ring-opening rate constants (*k*_2_/*k*_–2_ = 3.6). Tetrahydroisoquinoline derivative **11**, on the other hand has an excellent PSS performance (99% bleached at PSS) due to the ring-closing equilibrium (*k*_2_/*k*_–2_ = 75) being more strongly favoured than these previous examples (except **14**). The cyclic amine derivatives **12** and **13** are unusual as the first step is unfavourable under irradiation (*k*l1/*k*l–1 = 0.5, 0.6 respectively), and yet these switches have PSS bleaching of 65 and 91% respectively due to the more favourable second step (*k*_2_/*k*_–2_ = 3.7, 31 respectively). These results highlight that the overall PSS performance of these photoswitches is not determined by the rate constant of a single step in the mechanism. To rapidly reach a PSS enriched in the cyclic isomer the rate constants *k*l1 and *k*_2_ must be high, and their product (*k*l1*k*_2_) provides a method of comparing the ‘speed’ of switching (ESI-24.1†).

These data confirm **14** (*k*l1*k*_2_ = 6700 × 10^–3^ s^–2^) as the fastest forward switch under irradiation, with **1**, **4**, **9**, **10** and **11** (*k*l1*k*_2_ = 100–1000 × 10^–3^ s^–2^) also undergoing fast equilibration under irradiation. DASAs **2**, **3** and **13** (*k*l1*k*_2_ = 50–70 × 10^–3^ s^–2^) are significantly slower and **5–8** and **12** are the slowest forward switches (*k*l1*k*_2_ < 30 × 10^–3^ s^–2^). Importantly, our data also suggests that, at least in chloroform, *k*_2_ is 1–2 orders of magnitude faster for the aniline derivative **14** than the alkyl derivatives **1–13**. The kinetic data also indicates the poor cyclisation of alkyl derivatives (*e.g.***5–8**) may not be simply due to energy levels and barriers in the thermal 4π electrocyclisation,^28^ but also in the steps between photoexcitation and the formation of the required intermediate (**a′′′**), (*i.e.* a low *k*l1/*k*l–1) and these early steps require further investigation.

For the thermal equilibration in the dark (apparent half-life *t*_1/2_ in Table 2) it is clear the rate of ring opening (*k*_–2_) essentially determines this rate. DASAs **12** and **13** are unusual as their rate of equilibration in the light is slow and their rate of thermal equilibration is also slow (*t*_1/2_ = 91, 92 s), suggesting that these cyclic amines are conformationally restricted to impede the isomerisation process. The remainder of the slow forward switches have fast thermal equilibration times (*e.g.***5–8** have *t*_1/2_ = 6–11 s).

Our model also allows us to evaluate the population of the intermediate (**I**) species at the PSS (*e.g.*Fig. 5, ESI-29†). This is an informative approach as it reveals that in some instances the change in absorption greatly overestimates the degree of cyclisation. For example, diethylamine derivative **5** undergoes a 26% decrease in absorption at the PSS, yet this corresponds to just 17% of the cyclic product being formed, which will be important where the chemical properties of the cyclic isomer (*e.g.* polarity) are required to drive functional changes in systems where DASAs are used. Significantly, for each of DASAs **2–8** we predict approximately 20–30% of the intermediate is present at the PSS we measure.

The ‘best’^57^ switches are those where *k*l1/*k*l–1 and *k*_2_/*k*_–2_ are both large to ensure complete switching at PSS, and for fast switching *k*_–2_ must also be large to ensure fast thermal equilibration. This identifies DASAs derived from dimethylamine (**1**) and methylbenzylamine (**9**) as the ‘best’ performing switches in the series, with PSS over 90% cyclic, and apparent thermal half-lives of around 30 seconds. If faster switching is desired, DASA **4** provides an excellent example where both fast thermal equilibration (*t*_1/2_ = 10 seconds) and a good PSS (78% cyclic and 86% change in absorption) are achieved. For where complete bleaching is required, DASAs **11** and **14** provide the largest changes in absorption at PSS but at a cost of both the linear : cyclic ratio in the dark (% **a** in the dark: **11** = 74%; **14** = 43%) and slower thermal equilibrations (*t*_1/2_: 73 s, 265 s).

Compounds **1–13** were selected for this study as they are structurally similar, and have similar absorption properties. Given the varied photoswitching properties, we aimed to identify some key structure–function relationships. The Swain–Lupton parameters^59^ for the field and resonance effects (and hence measures of electronic nature such as Hammett *σ* values) are essentially identical for most of different substituents in DASAs **1–14**. As a result, using these parameters to evaluate which substituents are most effective, and why, is unlikely to be useful. In contrast the Taft inductive substituent constants (σ*,^58^ also known as polar substituent constants) vary notably between the substituents considered here.^60^ Interestingly, there is a very good correlation between this parameter and the rate of ring closing (log(*k*_2_)); this correlation improves when the associated steric parameter, *E*_s_, is incorporated noting that the effect is most significant for those DASAs with two bulky substituents (*i.e.* R ≠ Me) (see Fig. S53†).

**Fig. 6:**
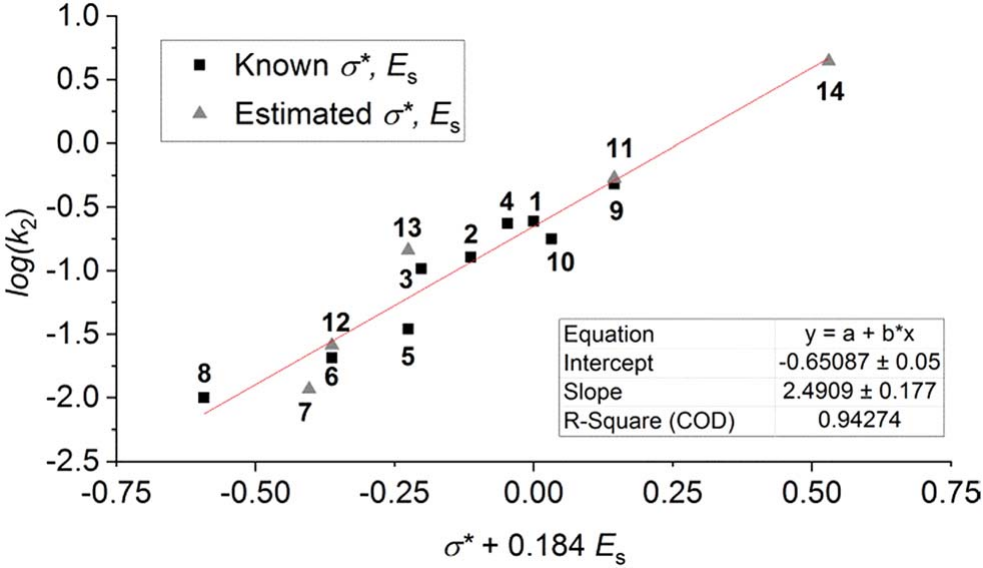
Correlations of the rate of ring closing (log(*k*_2_)) with a function of the Taft inductive substituent (σ*) and steric substituent (*E*_s_) constants, fitted by varying the co-efficient by which *E*_s_ is multiplied to optimise the linear fit.^58^ Values on the ordinate axis are determined by summing the values for each the amine substituents. Values of σ* + *E*_s_ are estimated for the following using the substituents listed: **7** estimated with 2× *n*-Bu; **11** with Bz and Me; **12** with 2× Pr; **13** with 2× Et; **14** with Me and Ph.

The correlation shown in Fig. 6 demonstrates that a larger value of σ* results in a greater rate constant for the cyclisation process. That is, an electron withdrawing substituent on the nitrogen centre favours the cyclisation step and electron donating substituents disfavour the cyclisation step. This is not unreasonable given that it might be considered that electron density is flowing towards the nitrogen centre as the reaction occurs. Similarly, the correlation shows that large groups disfavour the cyclisation. The rate of ring opening (*k*_–2_) shows the opposite trend with σ*, although the correlation is not as strong (ESI-25†). This result shows that despite the similar p*K*_a_ values of the protonated forms of the parent amines used to prepare DASAs **1–13**, and that the absorption profiles of **1–13** are almost identical compared with more drastic changes in donor groups,^44^ it is possible to tune photoswitching performance while retaining a constant chromophore.

The observation that the poorest switches (*i.e.* those that are disfavoured in both steps in the light) are also the switches with two bulky substituents on the amine, suggests steric effects may play an important role in both steps, even though electronic effects do contribute. DASAs formed with cyclic amines (**12**, **13**) are disfavoured in the first (*k*l1/*k*l–1) step, but are strongly favoured in the second (*k*_2_/*k*_–2_), indicating that factors influencing these steps can be tuned independently such that electronic properties can be used to favour rapid ring closing, while not inhibiting the initial step. Potentially the first steps of bond isomerisation and bond rotation may be significantly faster where at least one of the substituents on the amine is small (*i.e.* a methyl group).

**Fig. 7:**
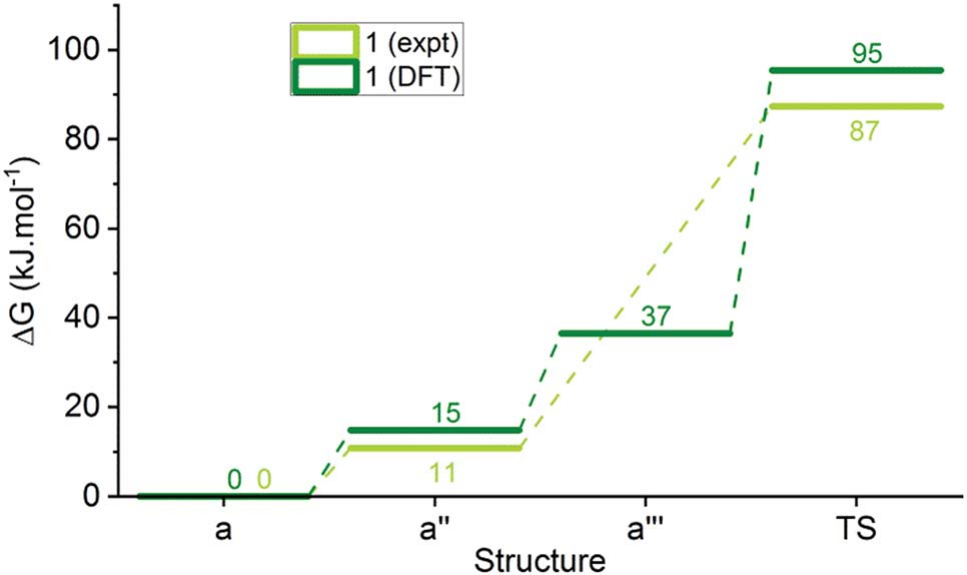
Reaction energy profile for DASA **1** determined from kinetic measurements and DFT calculations.

We performed DFT energy calculations (see ESI-33†) on the minimised structures of each of **1a**, **2a**, **5a**, **8a** and **14a**, the corresponding photoisomer **a′′** (considered our intermediate **I**), isomer **a′′′**, and the transition state (**TS**) of the ring closing reaction to cover a range of substituents in this study (Table 3, ESI-33†). These calculations indicate that proton transfer and ring closing is a concerted process, consistent with previous suggestions.^30^,^31^ The calculated transition state free energies are in good agreement with experimental values and an example for DASA **1** is shown in Fig. 7.

**Table 3:** Free energies of select DASAs relative to that of the linear isomer (**a**) for each molecule, determined by experiment and M06-2X/6-311+G(3df,2p) + SMD(chloroform) calculations^*a*^

DASA	**a′′** (expt)^*b*^	**a′′** (DFT)^*c*^	**a′′′** (DFT)^*c*^	**TS** (expt)^*d*^	**TS** (DFT)^*c*^	Δ*E* pyramidal–planar (DFT)^*e*^
**1**	11	15	37	87	95	–6.1
**2**	11	21	36	89	97	–5.3
**5**	12	24	39	93	103	–1.7
**8**	13	20	37	98	103	–1.0
**14**	20	19	39	89	92	—^*f*^

^*a*^All free energies at 298 K in kJ mol^–1^.

^*b*^Calculated from observed *k*d1/*k*d–1.

^*c*^See ESI-33 for computational details.

^*d*^Calculated from barrier corresponding to *k*_–2_ and the energy of cyclic isomer (from *k*_1_*k*_2_/*k*_–1_*k*_–2_ = [**B**]/[**A**]); also equal to energy of the intermediate **I** + barrier corresponding to *k*_2_.

^*e*^DFT calculation of the energy difference of model amines undergoing a transition from trigonal planar to pyramidal, see Scheme 3.

^*f*^Value not calculated for **14** as this molecule is electronically different to the others in the series and the cyclic form exists exclusively as the keto tautomer.

**Scheme 3:**
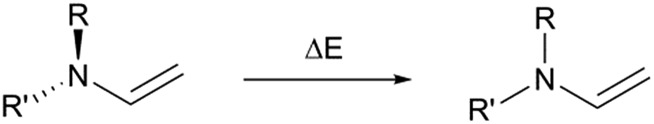
The modelled pyramidal to planar energy difference, with energies listed in Table 3.

The calculated relative energies of **a′′** and the transition state are higher in energy than the experimental values (Δ*G*_DFT_ – Δ*G*_expt_ = 3–10 and 1–12 kJ mol^–1^ respectively), but there is excellent agreement in the relative energies of these two species and the measured energy barriers. Although the DFT calculations of the transition state geometries for ring closing (ESI-33.2†) do not reveal any obvious steric interactions between the amine substituents and the bond forming reaction, we considered the change in geometry about the nitrogen going from trigonal planar to pyramidal could be key. The two amine substituents will be closer together in the cyclic product^61^ (and transition state) than in any of the linear structures where the amine retains its trigonal planar geometry, and this will introduce more steric strain with bulky groups. This idea is supported by DFT transition state geometries that show the distance between the two carbons attached to the nitrogen at the transition state increases in the order **14** < **1** < **2** ≈ **5** < **8**, which also parallels the calculated **TS** energies. To determine the relative energy contribution of this transition we used model imines (Scheme 3) to calculate the energy difference (Δ*E* in Table 3) between minimised trigonal planar and pyramidal geometries (ESI-33.2†). In all cases the pyramidal geometry is more stable; however, the energy difference decreases with increasing size of the R and R′ substituents presumably because the pyramidal form is destabilised by steric interactions. These calculated Δ*E* values correlate with the transition state energies for the ring closing reaction of the DASAs (Table 3), and suggests the steric interactions *between* the R and R′ groups associated with the pyramidalisation of the amine nitrogen are significant in determining the rate determining **TS** energy, and therefore of overall switching behaviour.

Finally we should note that the relative stabilities of the linear and cyclic forms are strongly solvent dependent (*e.g.* different ratios are observed in CD_3_CN, ESI-19†), and the rate constants of the two steps for photoisomerisation will also be solvent dependent. We have performed photoswitching experiments in 2-methyltetrahydrofuran (MeTHF) as an environmentally benign^62^ non-polar solvent (ESI-22, ESI-30 and ESI-31†). For all of DASAs **1–14** in MeTHF, fatigue resistance is improved (except **12a**), rates of thermal equilibration are slower (except for **12** and **13**), and the percent bleaching at PSS is higher, all compared to the same data in chloroform. Although we do not know the linear : cyclic composition in MeTHF in the dark, the linear form is expected to be the less polar and therefore should be more favoured in MeTHF than in chloroform, and a slower ring opening (*k*_–2_) is likely the key cause of the improved photoswitching performance. This combination of improved thermodynamics (more linear in the dark) and kinetics (slower ring opening) in non-polar solvents is likely general for DASA photoswitches.

## Conclusions

In conclusion, we have shown that minor structural modifications render this class of DASAs excellent switches in polar halogenated solvents, like chloroform. The use of a small methyl group as one of the amine substituents appears a prerequisite for favourable photoswitching properties. Our kinetic modelling has shown that the relative rate constants of both the photoisomerisation (*k*l1 and *k*l–1) and cyclisation (*k*_2_ and *k*_–2_) steps are sufficiently similar that both must be considered when analysing DASA photoswitching performance, and that it is possible to modulate these steps independently and without causing significant changes in the absorption properties. The use of substituent parameters such as σ* allows the relative rates of ring closing to be predicted, and will be instructive for designing tailored DASAs in future. Finally, we have demonstrated the fatigue resistance of DASAs are comparable with some of the best visible light photochromic molecules, and that DASAs have the potential to replace spiropyrans for many applications.

## Conflicts of interest

There are no conflicts to declare.

## Supplementary Material

Supplementary information

Crystal structure data
