# Supplementary material for: Structure–function relationships of donor–acceptor Stenhouse adduct photochromic switches
Source: Chem Sci. 2018 Sep 12;9(43):8242–52. doi: 10.1039/c8sc03218a (PMC6240811; doi:10.1039/c8sc03218a)
Supplement: Supplementary file 1 [file SC-009-C8SC03218A-s001.pdf]

Electronic Supplementary Material (ESI) for Chemical Science.
This journal is © The Royal Society of Chemistry 2018

Electronic Supporting Information

Structure-Function Relationships of Donor-Acceptor Stenhouse Adduct Photochromic Switches

Neil Mallo, Eric D. Foley, Hasti Iranmanesh, Aaron Kennedy, Ena T. Luis, Junming Ho, Jason B. Harper, Jonathon E. Beves

Table of Contents

1 General Experimental 7
1.1 General Experimental 7
1.2 General comment on NMR spectra 7
1.3 Synthetic overview 9

2 Summary of the compounds prepared in this study 10

3 Synthesis of precursor compounds 11
3.1 Synthesis of precursor **S1** 11

4 Synthesis and characterization of compound 1a/1b 12
4.1 Synthesis of **1a**/**1b** 12
4.2 ^{1}H NMR spectrum of **1** in CD_3CN 13
4.3 $^{13}C\{^{1}H\}$ NMR spectrum of **1** in CD_3CN 13

5 Synthesis and characterization of compound 2a/2b 14
5.1 Synthesis of **2a**/**2b** 14
5.2 ^{1}H NMR spectrum of **2** in CD_3CN 15
5.3 $^{13}C\{^{1}H\}$ NMR spectrum of **2** in CD_3CN 15

6 Synthesis and characterization of compound 3a/3b 16
6.1 Synthesis of **3a**/**3b** 16
6.2 ^{1}H NMR spectrum of **3** in CD_3CN 17
6.3 $^{13}C\{^{1}H\}$ NMR spectrum of **3** in CD_3CN 17
6.4 Conformational analysis of non-symmetrical substituted DASA **3** 18

7 Synthesis and characterization of compound 4a/4b 19
7.1 Synthesis of **4a**/**4b** 19
7.2 ^{1}H NMR spectrum of **4** in CD_3CN 20
7.3 $^{13}C\{^{1}H\}$ NMR spectrum of **4** in CD_3CN 20

8 Synthesis and characterization of compound 5a/5b 21
8.1 Synthesis of **5a**/**5b** 21
8.2 ^{1}H NMR spectrum of **5** in CD_3CN 22
8.3 $^{13}C\{^{1}H\}$ NMR spectrum of **5** in CD_3CN 22

9 Synthesis and characterization of compound 6a/6b 23
9.1 Synthesis of **6a**/**6b** 23
9.2 ^{1}H NMR spectrum of **6** in CD_3CN 24
9.3 $^{13}C\{^{1}H\}$ NMR spectrum of **6** in CD_3CN 24

10 Synthesis and characterization of compound 7a/7b 25
10.1 Synthesis of **7a**/**7b** 25
10.2 ^{1}H NMR spectrum of **7** in CD_3CN 26
10.3 $^{13}C\{^{1}H\}$ NMR spectrum of **7** in CD_3CN 26

11 Synthesis and characterization of compound 8a/8b 27
11.1 Synthesis of **8a**/**8b** 27
11.2 ^{1}H NMR spectrum of **8** in CD_3CN 28
11.3 $^{13}C\{^{1}H\}$ NMR spectrum of **8** in CD_3CN 28

12 Synthesis and characterization of compound 9a/9b 29

12.1 Synthesis of **9a/9b** 29
12.2 ^{1}H NMR spectrum of **9** in CD_3CN 30
12.3 $^{13}C\{^1H\}$ NMR spectrum of **9** in CD_3CN 30
12.4 ^1H-^{13}C HSQC NMR of **9** in CD_3CN 31

13 Synthesis and characterization of compound 10a/10b 32
13.1 Synthesis of **10a/10b** 32
13.2 ^{1}H NMR spectrum of **10** in CD_3CN 33
13.3 $^{13}C\{^1H\}$ NMR spectrum of **10** in CD_3CN 34
13.4 ^1H-^{13}C HSQC NMR of **10** in CD_3CN 35

14 Synthesis and characterization of compound 11a/11b 36
14.1 Synthesis of 11a/11b 36
14.2 ^{1}H NMR spectrum of 11 in CD_3CN 37
14.3 ^1H-^{13}C HSQC NMR spectrum of 11 in CD_3CN 37

15 Synthesis and characterization of compound 12a/12b 38
15.1 Synthesis of **12a/12b** 38
15.2 ^{1}H NMR spectrum of **12** in CD_3CN 39
15.3 $^{13}C\{^1H\}$ NMR spectrum of **12** in CD_3CN 39

16 Synthesis and characterization of compound 13a/13b 40
16.1 Synthesis of **13a/13b** 40
16.2 ^{1}H NMR spectrum of **13** in CD_3CN 41
16.3 ^1H-^{13}C HSQC NMR of **13** in CD_3CN 41

17 Synthesis and characterization of compound 14a/14b 42
17.1 Synthesis of **14a/14b** 42
17.2 ^{1}H NMR spectrum of **14** in CD_3CN 43
17.3 $^{13}C\{^1H\}$ NMR spectrum of **14** in CD_3CN 43
17.4 1D NOESY of cyclic DASA **14b'** 44

18 Comparison of ^{1}H NMR data of 1-14 in CD_3CN and $CDCl_3$ 45
18.1 Comparison of ^{1}H NMR spectra of **1-14** in CD_3CN 45
18.2 Comparison of NMR data in CD_3CN for **1-14** 46
18.3 Comparison of ^{1}H NMR spectra of **1-14** in $CDCl_3$ 47

19 Relative abundance of linear and cyclic isomers in $CDCl_3$ and CD_3CN for 1-14, measured by ^{1}H NMR 48

20 NMR spectroscopy assignment of cyclic isomers in $CDCl_3$: *enol* vs *keto* tautomers 49
20.1 2D NMR spectra of **12** in $CDCl_3$ 50
20.2 2D NMR spectra of **1** in $CDCl_3$ 51
20.3 2D NMR spectra of **9** in $CDCl_3$ 52
20.4 2D NMR spectra of **14** in $CDCl_3$ 53
20.5 Concentration dependency of *enol*/*keto* ratio 54

21 Influence of water on the linear:cyclic equilibrium of DASA 12 in $CDCl_3$ 55

22 Summary of absorption, fatigue resistance and apparent thermal half-life data in $CHCl_3$ and MeTHF 56

23 Kinetic modelling of UV-visible absorption data 57
23.1 Description of mechanistic model 57
23.2 Description of a simplified kinetic model 58

23.3 Modelling absorption ... 59
23.4 Kinetic data fitting ... 60
23.5 Determination of equilibrium concentrations ... 60

24 Summary of kinetic modelling data ... 62
24.1 Rate constants from kinetic modelling ... 62
24.2 Predicted and measured change in absorption and linear:cyclic dark equilibrium ratios ... 63
24.3 Comparison of relative energies of isomers A, I, the highest energy transition state (TS) and B. ... 64

25 Correlation of rate constants with Taft parameters ... 65

26 UV-vis absorption spectroscopy ... 68
26.1 Emission profile of the LED lamp ... 68

27 Single switching cycles (full spectra) in $CHCl_3$ ... 69
27.1 UV-vis spectra of **1** in chloroform during one photoswitching cycle ... 69
27.2 UV-vis spectra of **2** in chloroform during one photoswitching cycle ... 70
27.3 UV-vis spectra of **3** in chloroform during one photoswitching cycle ... 71
27.4 UV-vis spectra of **4** in chloroform during one photoswitching cycle ... 72
27.5 UV-vis spectra of **5** in chloroform during one photoswitching cycle ... 73
27.6 UV-vis spectra of **6** in chloroform during one photoswitching cycle ... 74
27.7 UV-vis spectra of **7** in chloroform during one photoswitching cycle ... 75
27.8 UV-vis spectra of **8** in chloroform during one photoswitching cycle ... 76
27.9 UV-vis spectra of **9** in chloroform during one photoswitching cycle ... 77
27.10 UV-vis spectra of **10** in chloroform during one photoswitching cycle ... 78
27.11 UV-vis spectra of **11** in chloroform during one photoswitching cycle ... 79
27.12 UV-vis spectra of **12** in chloroform during one photoswitching cycle ... 80
27.13 UV-vis spectra of **13** in chloroform during one photoswitching cycle ... 81
27.14 UV-vis spectra of **14** in chloroform during one photoswitching cycle ... 82

28 Fatigue resistance and thermal half-life calculations in chloroform ... 83
28.1 Fatigue resistance and thermal half-life calculation for **1** in chloroform ... 84
28.2 Fatigue resistance and thermal half-life calculation for **2** in chloroform ... 85
28.3 Fatigue resistance and thermal half-life calculation for **3** in chloroform ... 86
28.4 Fatigue resistance and thermal half-life calculation for **4** in chloroform ... 87
28.5 Fatigue resistance and thermal half-life calculation for **5** in chloroform ... 88
28.6 Fatigue resistance and thermal half-life calculation for **6** in chloroform ... 89
28.7 Fatigue resistance and thermal half-life calculation for **7** in chloroform ... 90
28.8 Fatigue resistance and thermal half-life calculation for **8** in chloroform ... 91
28.9 Fatigue resistance and thermal half-life calculation for **9** in chloroform ... 92
28.10 Fatigue resistance and thermal half-life calculation for **10** in chloroform ... 93
28.11 Fatigue resistance and thermal half-life calculation for **11** in chloroform ... 94
28.12 Fatigue resistance and thermal half-life calculation for **12** in chloroform ... 95
28.13 Fatigue resistance and thermal half-life calculation for **13** in chloroform ... 96
28.14 Fatigue resistance and thermal half-life calculation for **14** in chloroform ... 97
28.15 Fatigue resistance of **4** using optimized conditions ... 98
28.16 Oxygen sensitivity in chloroform ... 99

29 Modelled kinetic data ... 100
29.1 Modelled kinetic data for **1** in chloroform ... 100
29.2 Modelled kinetic data for **2** in chloroform ... 101
29.3 Modelled kinetic data for **3** in chloroform ... 102
29.4 Modelled kinetic data for **4** in chloroform ... 103

29.5 Modelled kinetic data for **5** in chloroform ... 104
29.6 Modelled kinetic data for **6** in chloroform ... 105
29.7 Modelled kinetic data for **7** in chloroform ... 106
29.8 Modelled kinetic data for **8** in chloroform ... 107
29.9 Modelled kinetic data for **9** in chloroform ... 108
29.10 Modelled kinetic data for **10** in chloroform ... 109
29.11 Modelled kinetic data for **11** in chloroform ... 110
29.12 Modelled kinetic data for **12** in chloroform ... 111
29.13 Modelled kinetic data for **13** in chloroform ... 112
29.14 Modelled kinetic data for **14** in chloroform ... 113

30 Single switching cycles (full spectra) in MeTHF ... 114
30.1 UV-vis spectra of **1** in MeTHF during one photoswitching cycle ... 114
30.2 UV-vis spectra of **2** in MeTHF during one photoswitching cycle ... 115
30.3 UV-vis spectra of **3** in MeTHF during one photoswitching cycle ... 116
30.4 UV-vis spectra of **4** in MeTHF during one photoswitching cycle ... 117
30.5 UV-vis spectra of **5** in MeTHF during one photoswitching cycle ... 118
30.6 UV-vis spectra of **6** in MeTHF during one photoswitching cycle ... 119
30.7 UV-vis spectra of **7** in MeTHF during one photoswitching cycle ... 120
30.8 UV-vis spectra of **8** in MeTHF during one photoswitching cycle ... 121
30.9 UV-vis spectra of **9** in MeTHF during one photoswitching cycle ... 122
30.10 UV-vis spectra of **10** in MeTHF during one photoswitching cycle ... 123
30.11 UV-vis spectra of **11** in MeTHF during one photoswitching cycle ... 124
30.12 UV-vis spectra of **12** in MeTHF during one photoswitching cycle ... 125
30.13 UV-vis spectra of **13** in MeTHF during one photoswitching cycle ... 126
30.14 UV-vis spectra of **14** in MeTHF during one photoswitching cycle ... 127

31 Fatigue resistance and thermal half-life calculations in MeTHF ... 128
31.1 Fatigue resistance and thermal half-life calculation for **1** in MeTHF ... 129
31.2 Fatigue resistance and thermal half-life calculation for **2** in MeTHF ... 130
31.3 Fatigue resistance and thermal half-life calculation for **3** in MeTHF ... 131
31.4 Fatigue resistance and thermal half-life calculation for **4** in MeTHF ... 132
31.5 Fatigue resistance and thermal half-life calculation for **5** in MeTHF ... 133
31.6 Fatigue resistance and thermal half-life calculation for **6** in MeTHF ... 134
31.7 Fatigue resistance and thermal half-life calculation for **7** in MeTHF ... 135
31.8 Fatigue resistance and thermal half-life calculation for **8** in MeTHF ... 136
31.9 Fatigue resistance and thermal half-life calculation for **9** in MeTHF ... 137
31.10 Fatigue resistance and thermal half-life calculation for **10** in MeTHF ... 138
31.11 Fatigue resistance and thermal half-life calculation for **11** in MeTHF ... 139
31.12 Fatigue resistance and thermal half-life calculation for **12** in MeTHF ... 140
31.13 Fatigue resistance and thermal half-life calculation for **13** in MeTHF ... 141
31.14 Fatigue resistance and thermal half-life calculation for **14** in MeTHF ... 142

32 X-ray crystallography data ... 143
32.1 Single crystal X-ray structure of **S1** ... 143
32.2 Single crystal X-ray structure of **1b** ... 144
32.3 Single crystal X-ray structure of **2b**$\cdot 2H_2O$ ... 145
32.4 Single crystal X-ray structure of **4a**·THF ... 146
32.5 Single crystal X-ray structure of 2{**9b**}$\cdot 7H_2O$ ... 147
32.6 Single crystal X-ray structure of **12a** ... 148
32.7 Single crystal X-ray structure of 2{**12b**}·DCM$\cdot 1.1H_2O$ ... 149
32.8 Single crystal X-ray structure of **14a**$\cdot CDCl_3$ ... 150
32.9 Single crystal X-ray structure of **14b** ... 151
32.10 Comparison of X-ray structure data ... 152

32.10.1 Comparison of X-ray data of linear structures 152
32.10.2 Comparison of X-ray data of cyclic structures 154

33 Computational studies 155
33.1 Computational details 155
33.2 DFT relative energies calculations for DASAs **1**, **2**, **5**, **8** and **14** 156
33.3 DFT Geometries and Relative Energies ΔE (M06-2X/6-31+G(d) in kJ mol^{-1}) of cyclic conformers of DASA **14** 156
33.4 Gas Phase basicity values 157
33.5 Gaussian archives for M06-2X/6-31+G(d)+SMD(chloroform) optimized geometries 158
33.5.1 **1a** 158
33.5.2 **1a''** 158
33.5.3 **1a'''** 159
33.5.4 **1-TS** 159
33.5.5 **2a** 160
33.5.6 **2a''** 160
33.5.7 **2a'''** 161
33.5.8 **2-TS** 161
33.5.9 **5a** 162
33.5.10 **5a''** 162
33.5.11 **5a'''** 163
33.5.12 **5-TS** 163
33.5.13 **8a** 164
33.5.14 **8a''** 165
33.5.15 **8a'''** 166
33.5.16 **8-TS** 167
33.5.17 **14a** 168
33.5.18 **14a''** 168
33.5.19 **14a'''** 169
33.5.20 14-TS 169

34 References 170

1 General Experimental

1.1 General Experimental

Reagents and solvents were purchased from either Sigma-Aldrich, Merck, Chem Supply, Combi-Blocks or Alfa Aeser, and were used without purification unless stated otherwise.

NMR spectroscopy was performed using a Bruker Avance III 400 with a Prodigy CryoProbe, a Bruker Avance III 500, a Bruker Avance III 600 or a Bruker Avance III HD 600 with a TCI CryoProbe. Samples were prepared using either CD_3CN or $CDCl_3$, purchased from Cambridge Isotope Laboratories, Inc. $CDCl_3$ was stored over K_2CO_3 to remove traces of acid. All chemical shifts were calibrated against residual solvent signals. All coupling constants (*J*) are reported in Hertz. Signals in the NMR spectra are reported as broad (br), singlet (s), doublets (d), triplets (t), quartets (q), quintets (qu), sextets (sx), septets (sept), or unclear multiplets (m). NMR spectra were processed with MestReNova 12.0.0 software. All NMR data are assigned unambiguously, except where specified. In cases were satisfactory $^{13}C\{^{1}H\}$ spectra could not be obtained due to very weak signals (likely due to exchange processes), ^{1}H-^{13}C HSQC spectra are provided.

UV-vis experiments were performed on an Agilent Cary 60 Bio UV-Visible Spectrophotometer equipped with a customized Cary Single Cell Peltier Accessory, keeping the samples at 25 °C unless stated otherwise. The cell holder was modified to allow for irradiation perpendicular to the direction of measurement, as previously described.[1] A Luxeon Rebel LED (lime, 567 nm, operated at 12 V, 1000 mA) was mounted on a heat sink positioned 4 cm away from the cell, and the beam was focused on the cuvette using a Carclo 20.0 mm Fibre Coupling Lens. All samples were stirred to ensure homogeneity. A timer relay module (FRM01) was used to control the irradiation cycles.

High-resolution mass spectrometry (HR-MS) experiments were performed on a hybrid linear quadrupole ion trap mass spectrometer (Thermo LTQ Orbitrap XL) equipped with an external nanospray ionisation (NSI) source.

1.2 General comment on NMR spectra

All ^{1}H NMR signals are reported as apparent multiplets. For example, ^{1}H NMR signals for H^{h} are doublets-of-doublets, but the coupling constants are sufficiently similar that these signals appear as triplets, and are reported as such below.

All ratios of linear-to-cyclic isomers are calculated using the integrals of all non-overlapping ^{1}H NMR signals for each isomer.

For **1a**–**3a** and **5a**–**8a**, the ^{1}H NMR signals of protons H^{b}, $H^{b'}$, the hydroxyl (OH), H^{e} and H^{g} (see Figure 1 for atom labelling) are relatively insensitive to substitution with differences under 0.05 ppm between compounds in this series. For benzyl derivatives **9a** and **10a**, and tetrahydroisoquinoline derivative **11a**, most ^{1}H NMR signals are also similar to the alkyl derivatives, with the notable exception of H^{e}, which is shifted downfield by 0.12, 0.13 and 0.24 ppm respectively, relative to the corresponding signal in **1a**. Given this proton is far from the donor amine, this change is likely due to an electronic through-bond effect rather than a through-space interaction, and demonstrates the delocalized nature of the triene bonds. DASA **4a** has ^{1}H NMR signals for H^{g}, H^{h} and H^{i} all shifted upfield ($\Delta\delta = \delta$(**4a**) – δ(**1a**) for H^{g}, H^{h}, H^{i} = –0.20, –0.12, –0.33, respectively) as the flexible linker allows the phenyl ring to shield these protons by through-space interactions.

The ^{13}C NMR signals of DASAs **1**–**14** show considerably less variation than the ^{1}H signals, with ^{13}C signals for carbons C^{a}–C^{h} varying less than 1 ppm between compounds **1**–**13**. Other than the

carbons attached directly to the amine (C^i, C^j, C^k), the signal showing the greatest variation is that of C^e ($\Delta\delta = \delta(\mathbf{x}) - \delta(\mathbf{1a})$ = –0.2 to +3.6), mirroring the behaviour of the signal for H^e in the ^{1}H NMR spectra. The reliable NMR chemical shifts of these carbon signals allow confident assignment of the ^{1}H spectra using ^{1}H–^{13}C HSQC spectroscopy, however in some cases the ^{13}C NMR signals of these species in saturated solutions were very weak or even unobservable (SI-1.2).

The different electronic properties of aniline-derived DASA **14**, as evidenced by the different absorption maximum, are also reflected in the ^{1}H NMR spectrum. NMR signals for protons along the triene are significantly shifted with respect to the alkyl derivatives **1-10**: the signal for OH is shifted upfield by 0.14 ppm, and the signals of H^e, H^g, H^h and H^i are all shifted downfield by 0.11–0.13 ppm as the bonds in the triene are more delocalized in the aniline derivative compared with the alkyl derivatives.

The ^{13}C NMR chemical shifts for most carbons were consistent between compounds **1**–**14** and this allowed confident assignment of the ^{1}H spectra by ^{1}H-^{13}C HSQC. However, in some cases the ^{13}C NMR signals of saturated solutions were very weak or even unobservable. Two factors contribute to this phenomenon. The first is simply solubility, with DASAs such as the dimethyl derivative **1** having much lower solubility in either $CDCl_3$ or CD_3CN than derivatives such as the dioctyl derivative. The other contributing factor is could be slow exchange processes. In most cases, multiple linear (**a**) conformers in slow exchange are observable using ^{1}H NMR spectroscopy. In the cases where sharp ^{13}C NMR signals are observed, multiple conformers in slow exchange are also observable using ^{13}C NMR spectroscopy. In cases with the weakest ^{13}C NMR signals the same exchange processes are presumably faster, becoming intermediate on the NMR timescale and resulting in broadened signals.

1.3 Synthetic overview

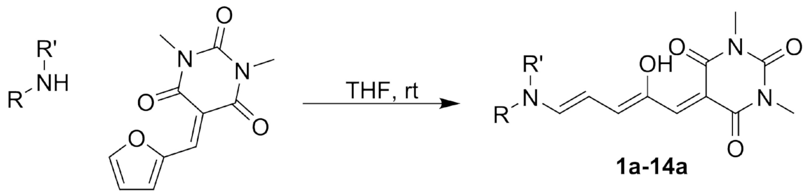

S1 **1a-14a**

Scheme S1. General synthesis of compounds **1a-14a**

2 Summary of the compounds prepared in this study

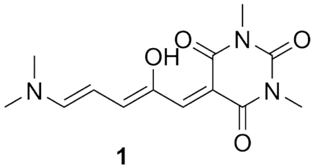

1

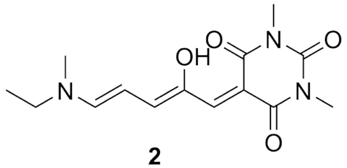

2

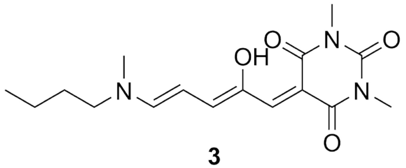

3

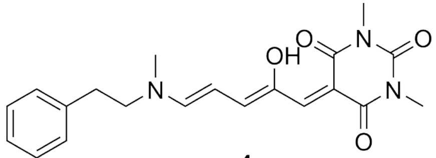

4

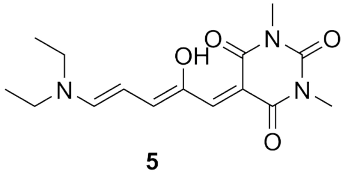

5

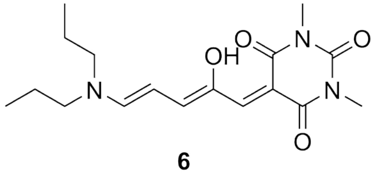

6

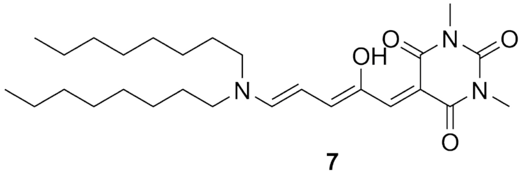

7

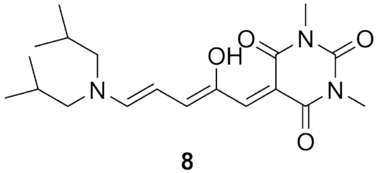

8

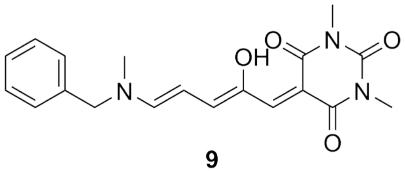

9

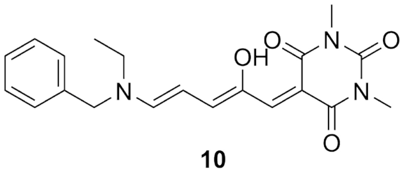

10

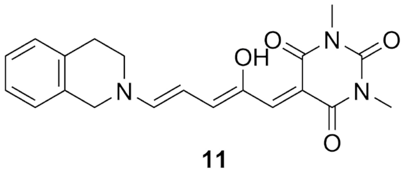

11

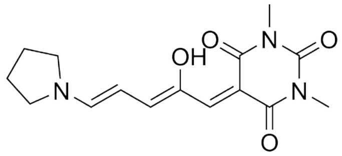

12

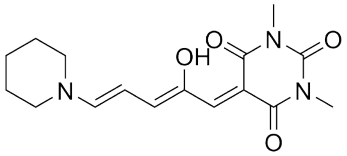

13

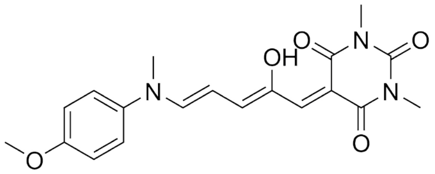

14

Figure S1. Structures of the donor-acceptor Stenhouse adducts prepared in this study.

3 Synthesis of precursor compounds

3.1 Synthesis of precursor S1

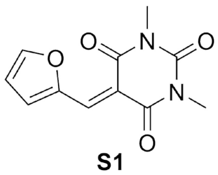

S1

Compound **S1** was prepared according to a reported procedure. Spectral properties matched previously reported values.[2]

4 Synthesis and characterization of compound 1a/1b

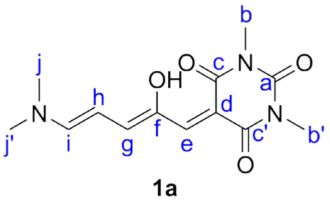

1a

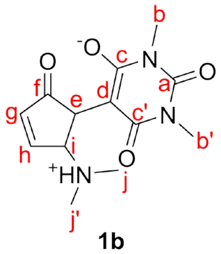

1b

4.1 Synthesis of 1a/1b

Dimethylamine (40% in H_2O, 140 µL, 1.1 mmol) was added to a stirred solution of **S1** (234 mg, 1.0 mmol) in THF (5 mL), resulting in an immediate change of colour from yellow to intense purple. The solution was stirred at room temperature for 30 min, during which a precipitate formed. The precipitate was collected by filtration, washed with cold THF (2 mL) and dried. The crude was purified by column chromatography (SiO_2, 7% MeOH in DCM). The product was triturated from the column fractions by the addition of hexane, cooled to -20 °C, collected by filtration and dried under vacuum. **1a** was isolated as a purple powder (65 mg, 0.23 mmol, 23%).

1a ^{1}H NMR (600 MHz, CD_3CN) δ 12.59 (s, 1H, H^{OH}), 7.61 (d, J = 12.0 Hz, 1H, H^{i}), 7.00 (dd J = 12.7 Hz, J = 1.0 Hz, 1H, H^{g}), 6.88 (s, 1H, H^{e}), 6.08 (t, J = 12.3 Hz, 1H, H^{h}), 3.30 (s, 3H, $H^{j'}$), 3.23 (s, 3H, H^{b}), 3.20 (s, 3H, $H^{b'}$), 3.16 (s, 3H, H^{j}).

1a ^{13}C NMR (151 MHz, CD_3CN) δ 162.9 (C^{i}), 153.8 (C^{g}), 146.0 (C^{f}), 133.0 (C^{e}), 105.2 (C^{h}), 47.4 ($C^{j'}$), 39.2 (C^{j}), 28.3 ($C^{b'}$), 28.2 (C^{b}).

^{13}C NMR signals for this compound were extremely week, even in DEPT spectra measured on saturated samples measured at 151 MHz. This is presumably due to exchange between conformers.

1b ^{1}H NMR (600 MHz, CD_3CN) δ 7.63 (br s, 1H, H^{h}), 6.49 (d, J = 5.5 Hz, 1H, H^{g}), 4.51 (s, 1H, H^{i}), 3.82 (s, 1H, H^{e}), 3.11 (s, 6H, H^{b}, $H^{b'}$), 2.87 (s, 6H, H^{j}, $H^{j'}$).

In solution (CD_3CN), **1** is present as a mixture isomers **1a** and **1b**, in a ratio of 1.0 : 0.22.

HR-NSI-MS m/z 280.12756 $[M+H]^+$ requires 280.12918, 302.10942 $[M+Na]^+$ requires 302.11113.

UV-vis ($CHCl_3$): λ_{max}/nm 565

4.2 ^{1}H NMR spectrum of 1 in CD_3CN

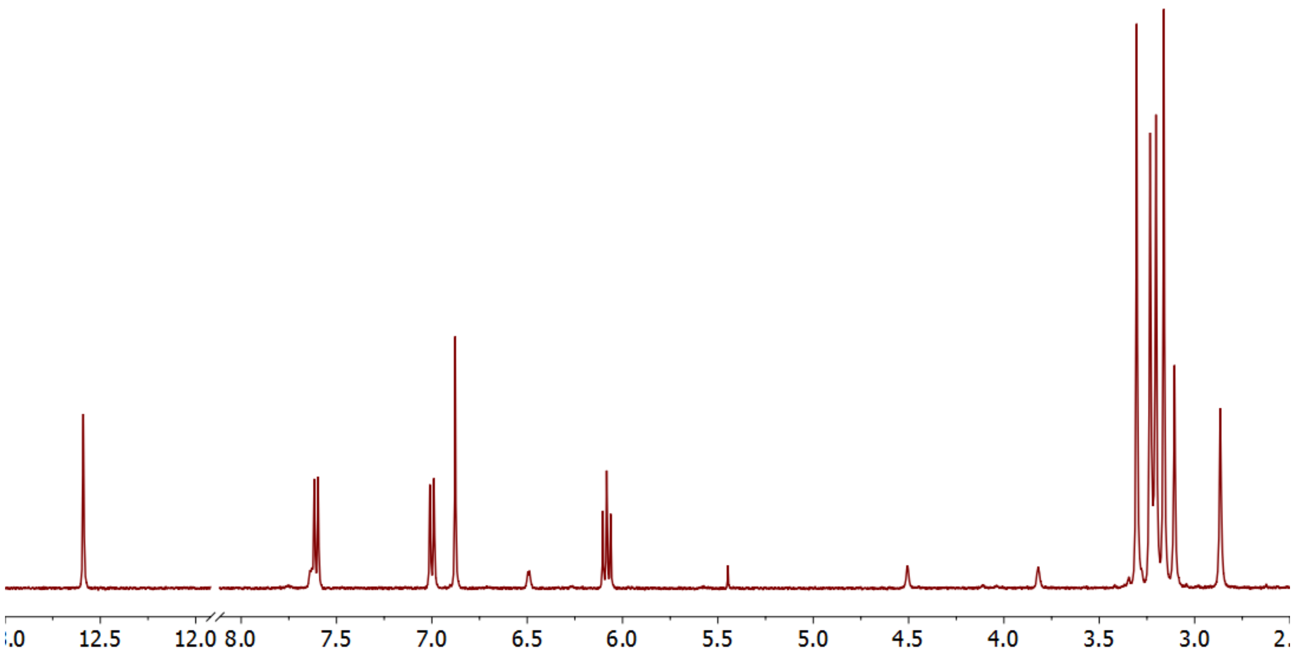

Figure S2. ^{1}H NMR (600 MHz, CD_3CN, 298 K) spectrum of **1**.

4.3 $^{13}C\{^1H\}$ NMR spectrum of 1 in CD_3CN

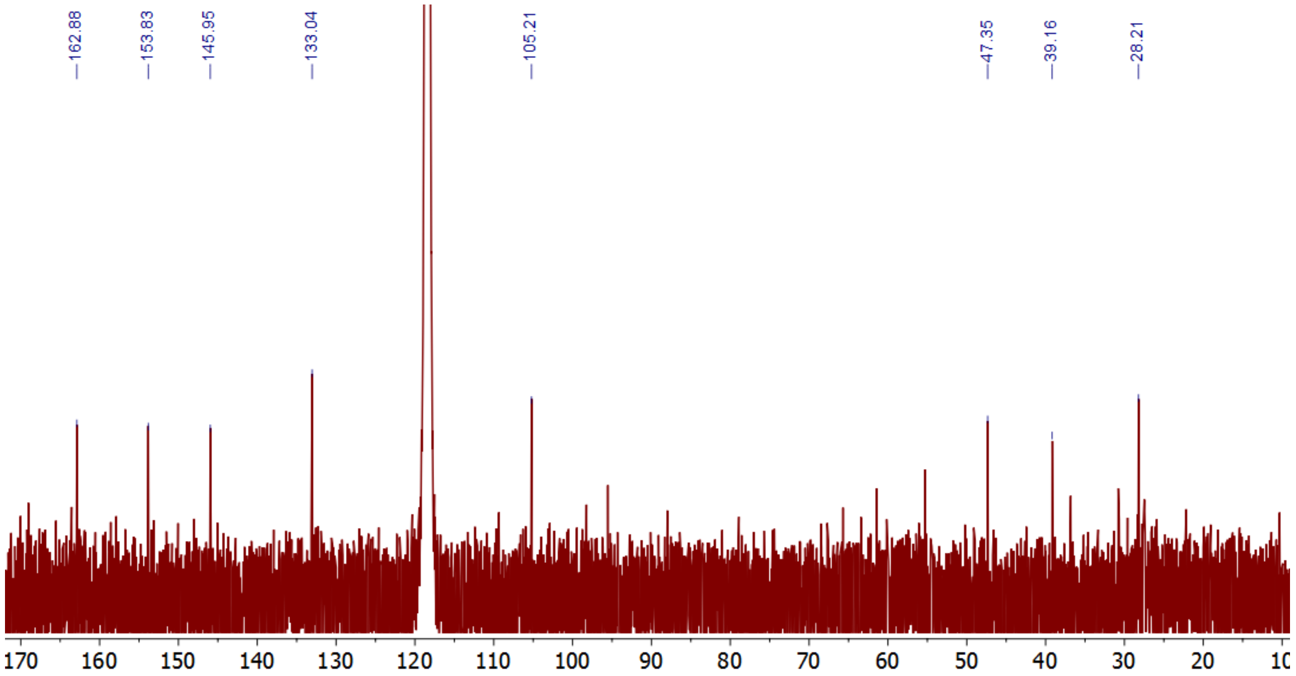

Figure S3. $^{13}C\{^1H\}$ NMR (151 MHz, CD_3CN, 298 K) spectrum of **1**.

5 Synthesis and characterization of compound 2a/2b

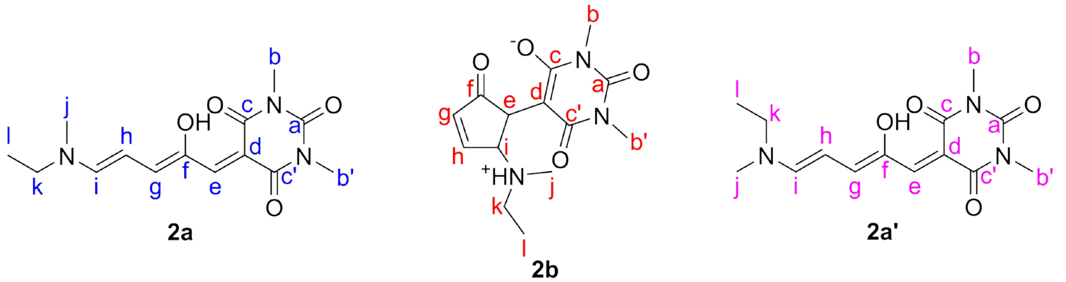

2a 2b 2a'

5.1 Synthesis of 2a/2b

Ethylmethylamine (100 µL, 1.1 mmol) was added to a stirred solution of **S1** (220 mg, 0.94 mmol) in THF (5 mL), resulting in an immediate change of colour from yellow to intense purple. The solution was stirred at room temperature for 30 min, during which a precipitate formed. The precipitate was collected by filtration, washed with cold THF (2 mL) and dried. The crude was purified by column chromatography (SiO_2, 7% MeOH in DCM). The product was triturated from the column fractions by the addition of hexane, cooled to -20 °C, collected by filtration and dried under vacuum. **2a** was isolated as a purple powder (68 mg, 0.23 mmol, 24%).

2a ^{1}H NMR (600 MHz, CD_3CN) δ 12.59 (s, 1H, H^{OH}), 7.66 (d, J = 11.8 Hz, 1H, H^{i}), 7.00 (br d J = 12.8 Hz, H^{g}), 6.88 (s, 1H, H^{e}), 6.08 (t, J = 12.3 Hz, 1H, H^{h}), 3.55 (q, J = 7.3 Hz, 2H, H^{k}), 3.23 (s, 3H, H^{b}), 3.20 (s, 3H, H$^{b'}$), 3.16 (s, 3H, H^{j}), 1.27 (t, J = 7.3 Hz, 3H, H^{l}).

2a ^{13}C NMR (151 MHz, CD_3CN) δ 165.9 (C^{c}), 163.8 (C$^{c'}$), 161.6 (C^{i}), 153.8 (C^{g}), 152.8 (C^{a}), 146.5 (C^{f}), 134.6 (C^{e}), 105.2 (C^{h}), 96.4 (C^{d}), 55.5 (C^{k}), 37.2 (C^{j}), 28.4 (C$^{b'}$), 28.4 (C^{b}), 13.8 (C^{l}).

2a' ^{1}H NMR (600 MHz, CD_3CN) δ 12.59 (s, 1H, H^{OH}), 7.56 (d, J = 11.9 Hz, 1H, H^{i}), 7.00 (br d J = 12.6 Hz, H^{g}), 6.87 (s, 1H, H^{e}), 6.14 (t, J = 12.4 Hz, 1H, H^{h}), 3.55 (q, J = 7.3 Hz, 2H, H^{k}), 3.29 (s, 3H, H^{j}), 3.23 (s, 3H, H^{b}), 3.20 (s, 3H, H$^{b'}$), 1.25 (t, J = 7.3 Hz, 3H, H^{l}).

2a' ^{13}C NMR (151 MHz, CD_3CN) δ 165.9 (C^{c}), 163.8 (C$^{c'}$) 162.3 (C^{i}), 154.1 (C^{g}), 152.8 (C^{a}), 146.4 (C^{f}), 134.2 (C^{e}), 104.9(C^{h}), 96.2 (C^{d}), 47.3 (C^{k}), 44.9 (C^{j}), 28.4 (C$^{b'}$), 28.4 (C^{b}), 11.8 (C^{l}).

2b ^{1}H NMR (600 MHz, CD_3CN) δ 7.62 (d, J = 5.6 Hz, 1H, H^{h}), 6.45 (d, J = 5.6 Hz, 1H, H^{g}), 4.58 (s, 1H, H^{i}), 3.82 (s, 1H, H^{e}), 3.11 (s, 6H, H^{b}, H$^{b'}$), 2.74 (s, 3H, H^{j}), 1.19 (t, J = 7.2 Hz, 3H, H^{l}). The signal for H^{k} is obscured by the signal for H^{j}; ^{1}H-^{1}H COSY and ^{1}H-^{13}C HSQC NMR spectroscopy indicate the signal occurs at 3.16 ppm.

In solution (CD_3CN), **2** is present as a mixture of conformers **2a** and **2a'** and isomer **2b**, in a ratio of 2.2 : 1.0 : 0.39.

HR-NSI-MS m/z 294.14352 $[M+H]^+$ requires 294.14483, 316.12532 $[M+Na]^+$ requires 316.12678.

UV-vis ($CHCl_3$): λ_{max}/nm 565

5.2 ^{1}H NMR spectrum of 2 in CD_3CN

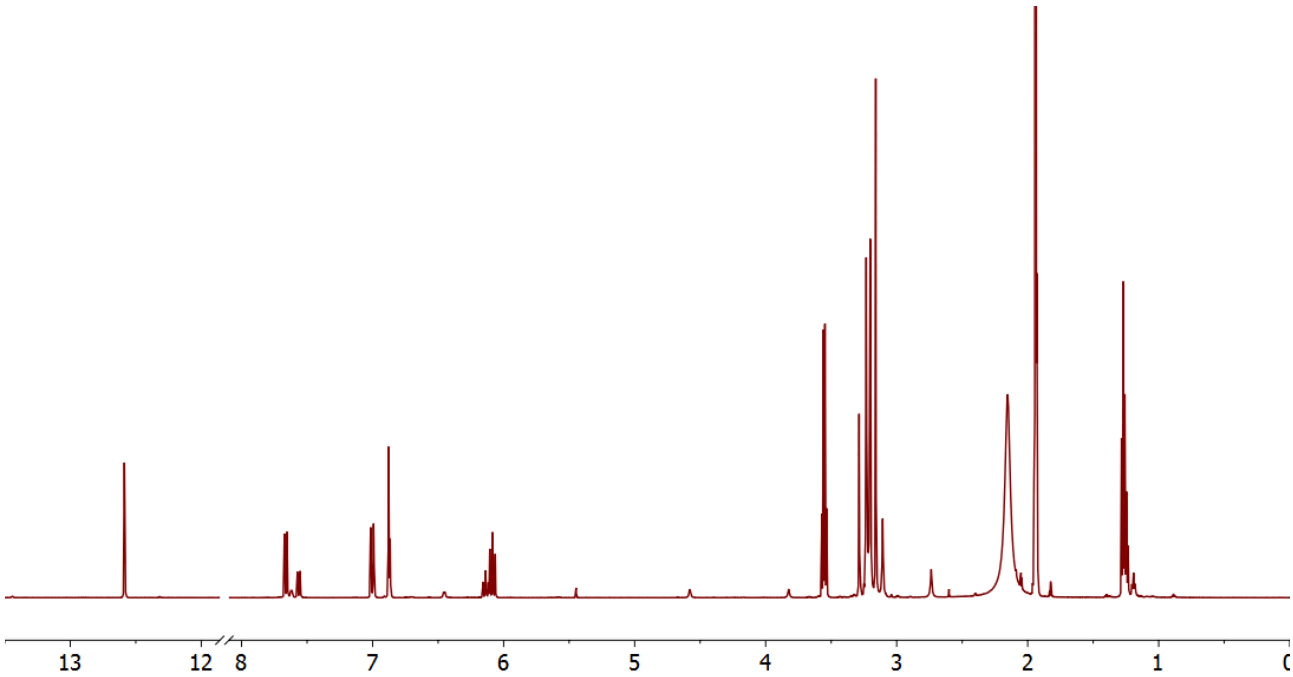

Figure S4. ^{1}H NMR (600 MHz, CD_3CN, 298 K) spectrum of **2**.

5.3 $^{13}C\{^{1}H\}$ NMR spectrum of 2 in CD_3CN

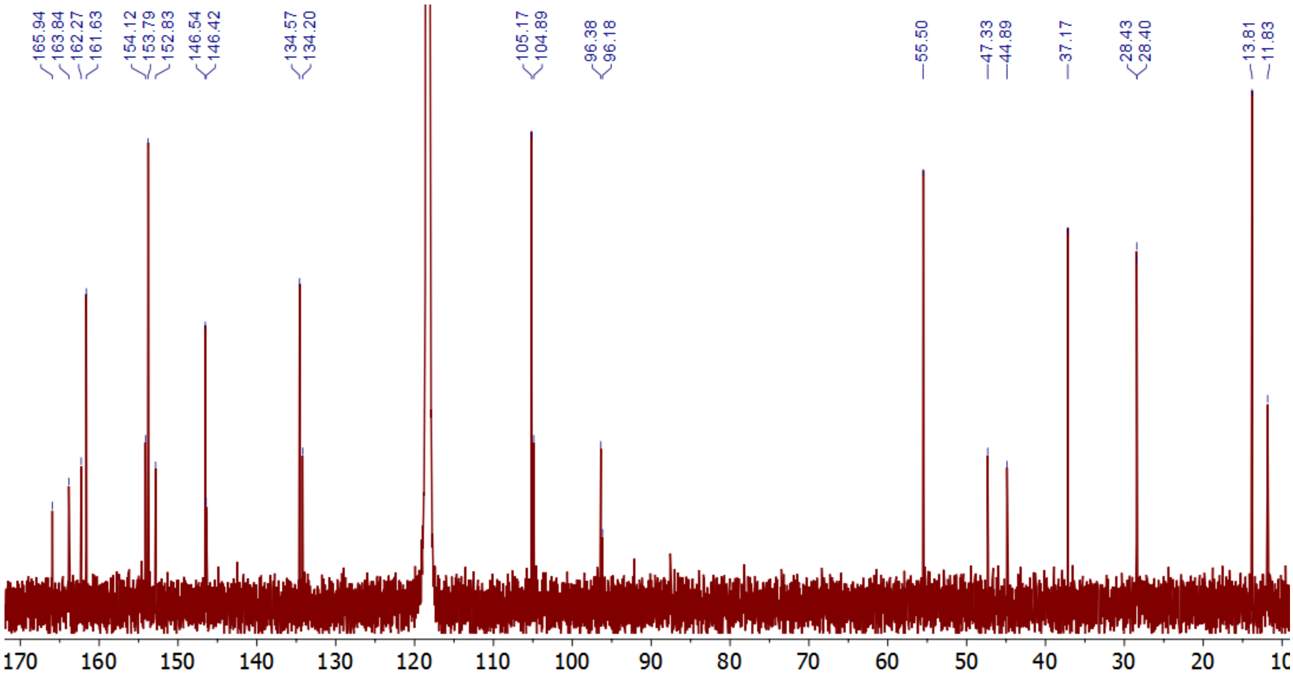

Figure S5. $^{13}C\{^{1}H\}$ NMR (151 MHz, CD_3CN, 298 K) spectrum of **2**.

6 Synthesis and characterization of compound 3a/3b

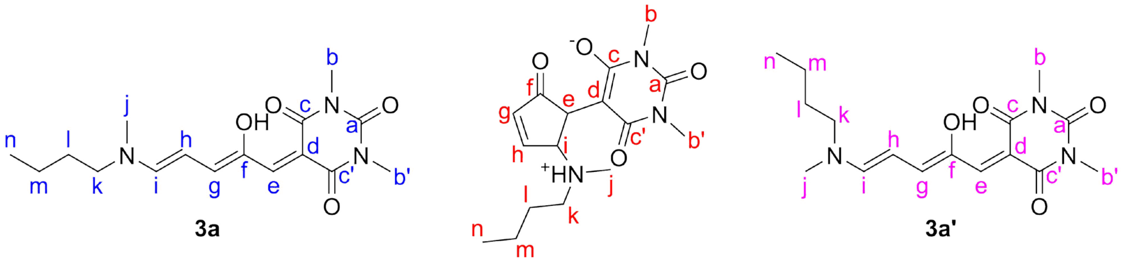

3a **3b** **3a'**

6.1 Synthesis of 3a/3b

Butylmethylamine (130 µL, 1.10 mmol) was added to a stirred suspension of **S1** (234 mg, 1.00 mmol) in THF (5 mL), resulting in an immediate change in colour from yellow to intense purple. The solution was stirred at room temperature overnight, during which a pink precipitate formed. The product was collected by filtration, washed with Et_2O, and dried under vacuum. **3a** was isolated as a pink solid (131 mg, 0.41 mmol, 41%).

3a ^{1}H NMR (600 MHz, CD_3CN) δ 12.58 (s, 1H, H^{OH}), 7.63 (d, J = 11.8 Hz, 1H, H^{i}), 6.99 (d, J = 12.8 Hz, 1H, H^{g}), 6.89 (s, 1H, H^{e}), 6.07 (t, J = 12.3 Hz, 1H, H^{h}), 3.50 (t, J = 7.2 Hz, 2H, H^{k}), 3.23 (s, 3H, H^{b}), 3.20 (s, 3H, $H^{b'}$), 3.15 (s, 3H, H^{j}), 1.65 (m, 2H, H^{l}), 1.31 (sx, J = 7.5 Hz, 2H, H^{m}), 0.94 (t, J = 7.4 Hz, 3H, H^{n}).

3a ^{13}C NMR (151 MHz, CD_3CN) δ 165.9 (C^{c}), 163.8 ($C^{c'}$), 161.9 (C^{i}), 153.6 (C^{g}), 152.8 (C^{a}), 146.6 (C^{f}), 134.8 (C^{e}), 104.9 (C^{h}), 96.5 (C^{d}), 60.3 (C^{k}), 37.4 (C^{j}), 30.6 (C^{l}), 28.4 ($C^{b'}$), 28.4 (C^{b}), 20.0 (C^{m}), 13.8 (C^{n}).

3a' ^{1}H NMR (600 MHz, CD_3CN) δ 12.58 (s, 1H, H^{OH}), 7.58 (d, J = 12.0 Hz, 1H, H^{i}), 6.99 (d, J = 12.8 Hz, 1H, H^{g}), 6.86 (s, 1H, H^{e}), 6.14 (t, J = 12.3 Hz, 1H, H^{h}), 3.50 (t, J = 7.2 Hz, 2H, H^{k}), 3.29 (s, 3H, H^{j}), 3.23 (s, 3H, H^{b}), 3.20 (s, 3H, $H^{b'}$), 1.65 (m, 2H, H^{l}), 1.38 (sx, J = 7.5 Hz, 2H, H^{m}), 0.96 (t, J = 7.4 Hz, 3H, H^{n}).

3a' ^{13}C NMR (151 MHz, CD_3CN) δ 165.9 (C^{c}), 163.8 ($C^{c'}$), 162.7 (C^{i}), 154.0 (C^{g}), 152.8 (C^{a}), 146.4 (C^{f}), 134.2 (C^{e}), 105.0 (C^{h}), 96.2 (C^{d}), 52.1 (C^{k}), 45.5 (C^{j}), 29.3 (C^{l}), 28.4 ($C^{b'}$), 28.4 (C^{b}), 20.6 (C^{m}), 13.9 (C^{n}).

3b ^{1}H NMR (600 MHz, CD_3CN) δ 7.68 (br d, J = 5.5 Hz, 1H, H^{h}), 6.49 (br d, J = 6.0 Hz, 1H, H^{g}), 4.63 (s, 1H, H^{i}), 3.92 (s, 1H, H^{e}), 3.11 (s, 6H, H^{b}, $H^{b'}$), 2.82 (s, 3H, H^{j}), 0.83 (t, J = 7.0 Hz, 3H, H^{n}). Signals for H^{k}, H^{l} and H^{m} are obscured by the signals of the linear isomer.

In solution (CD_3CN), **3** is present as a mixture of conformers **3a** and **3a'** and isomer **3b**, in a ratio of 2.6 : 1.0 : 0.35.

HR-NSI-MS m/z 322.17629 $[M+H]^+$ requires 322.17613, 344.15824 $[M+Na]^+$ requires 344.15808.

UV-vis ($CHCl_3$): λ_{max}/nm 566

6.2 ¹H NMR spectrum of 3 in CD_3CN

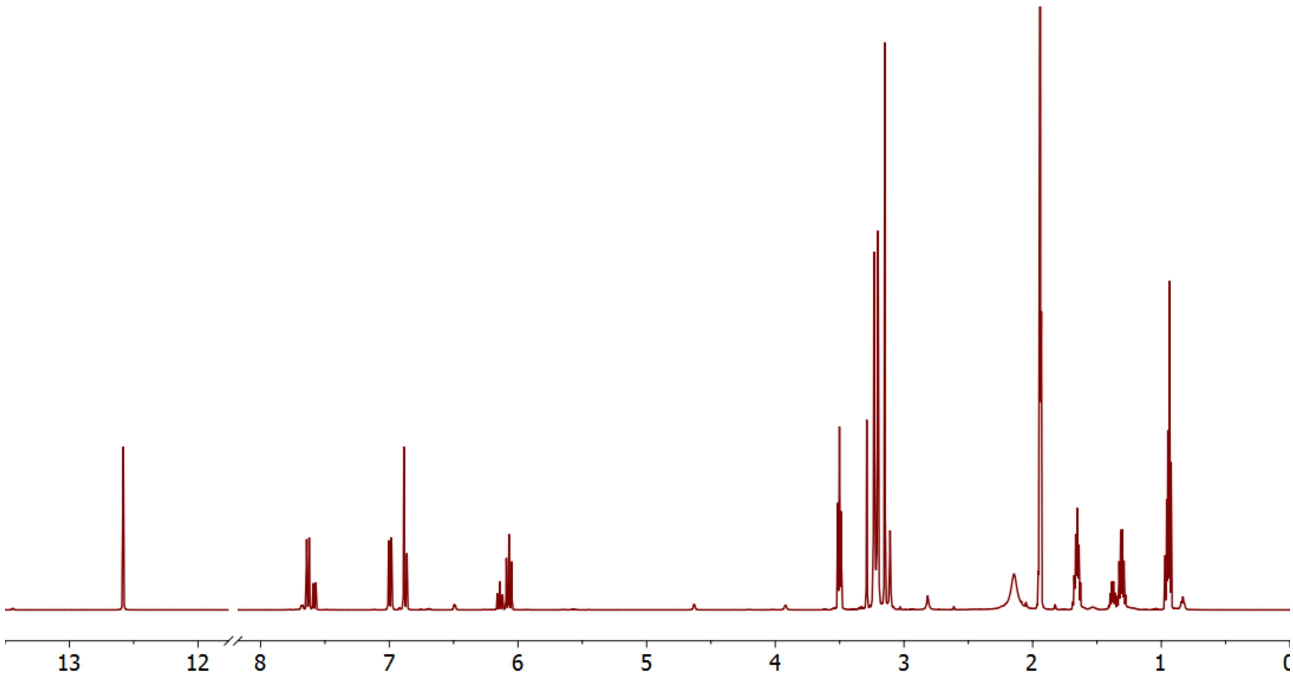

Figure S6. ¹H NMR (600 MHz, CD_3CN, 298 K) spectrum of **3**.

6.3 ¹³C{¹H} NMR spectrum of 3 in CD_3CN

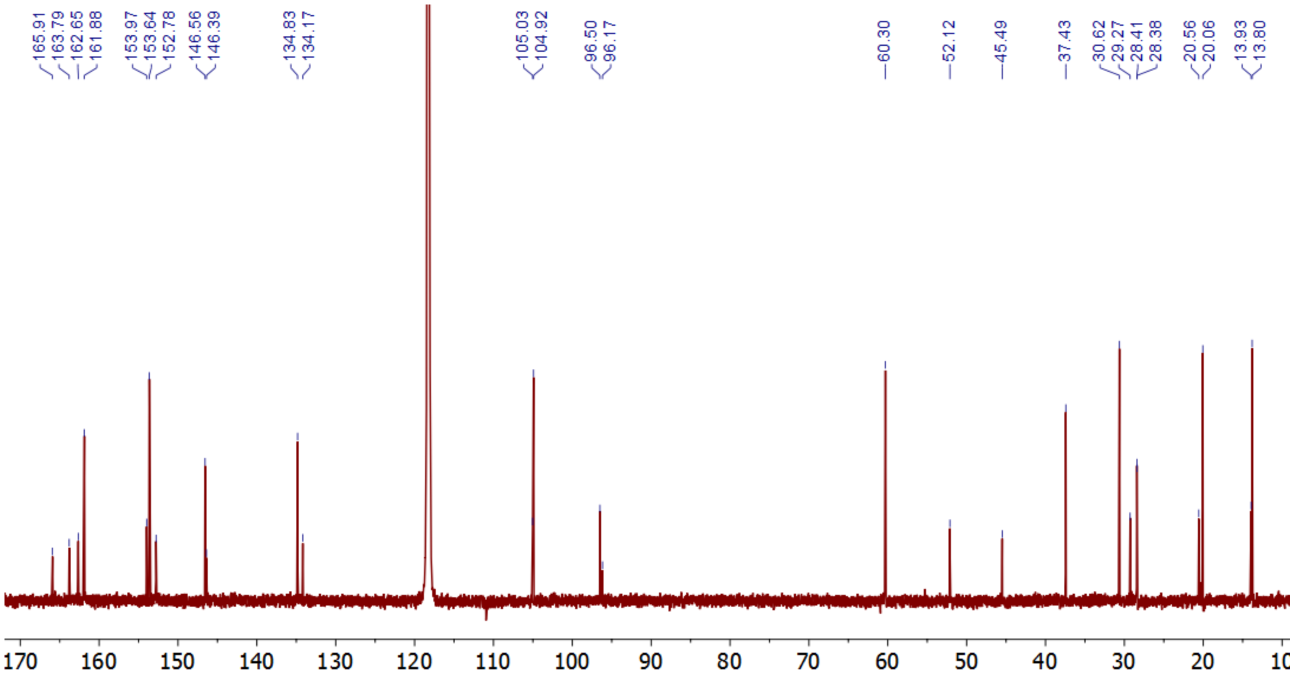

Figure S7. ¹³C{¹H} NMR (151 MHz, CD_3CN, 298 K) spectrum of **3**.

6.4 Conformational analysis of non-symmetrical substituted DASA 3

Restricted rotation around the C^i-N bond due to significant double bond character enables the detection of two conformers. At room temperature both conformers can be observed using ^1H NMR spectroscopy, the interconversion is too fast to get NOE data on the individual conformers. Cooling to 243 K slows the exchange enough to unambiguously assign the conformers.

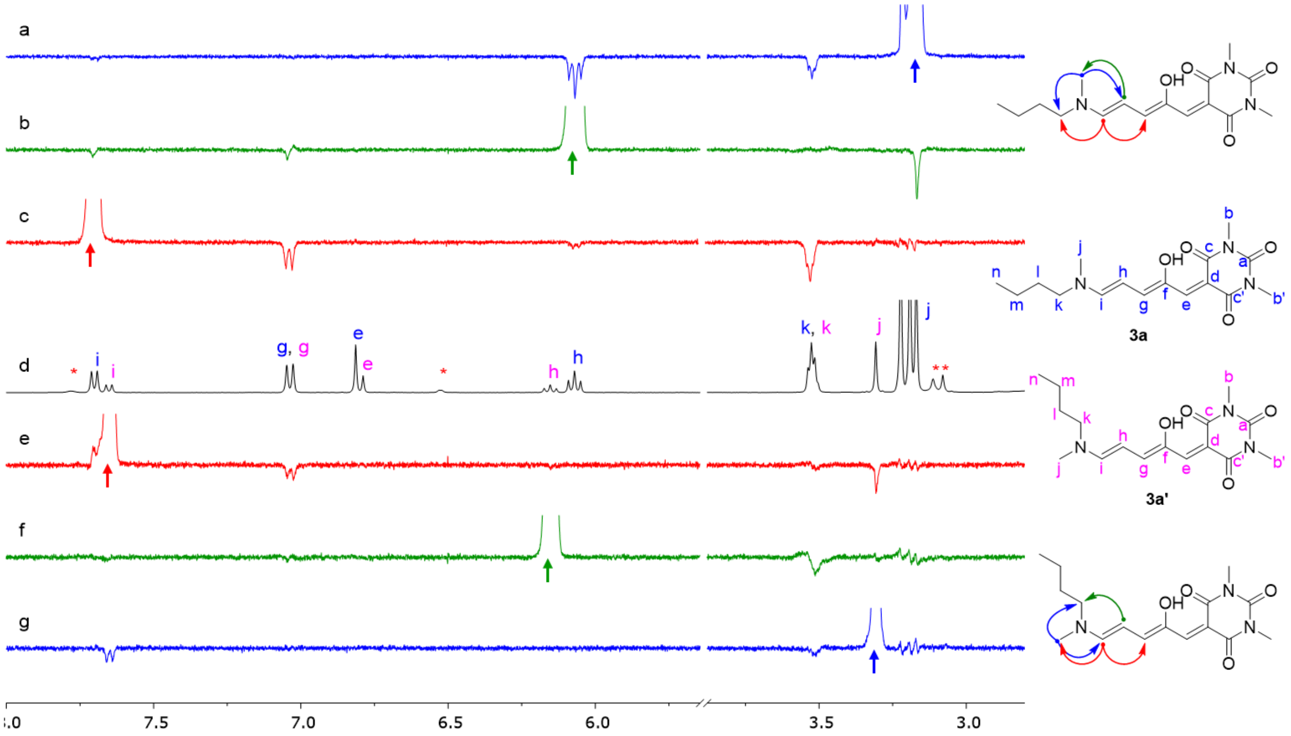

Figure S8. Stacked 1D-NOESY spectra (600 MHz, CD_3CN, 243 K) of the major isomer (a-c), minor isomer (e-g) and the ^1H reference spectrum (d) of DASA **3**. Signals marked with a red asterisk (*) are from the cyclic isomer.

7 Synthesis and characterization of compound 4a/4b

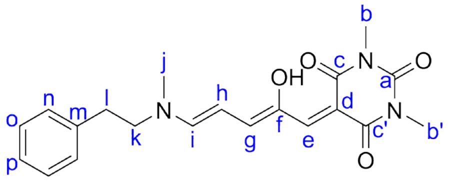

4a

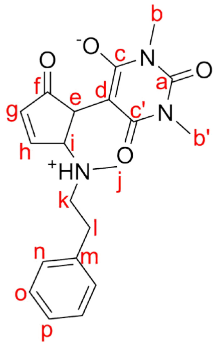

4b

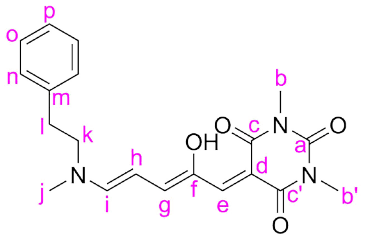

4a'

7.1 Synthesis of 4a/4b

N-Methylphenethylamine (150 µL, 1.00 mmol) was added to a stirred solution of **S1** (234 mg, 1.00 mmol) in THF (10 mL), resulting in an immediate change in colour from yellow to intense purple. The solution was stirred at room temperature for 4 days. Et_2O (10 mL) was added and the solution was stored at -20 °C, upon which solids precipitated. The product was collected by filtration, washed with cold Et_2O and dried under vacuum. The crude was purified by column chromatography (SiO_2, 5% MeOH in DCM). The product was triturated from the column fractions by the addition of hexane, cooled to -20 °C, collected by filtration and dried under vacuum. **4a** was isolated as a purple powder (65 mg, 0.18 mmol, 18%).

4a ^{1}H NMR (600 MHz, CD_3CN) δ 12.53 (br s, 1H, H^{OH}), 7.35-7.22 (m, 6H, H^n, H^o, H^p, H^i), 6.87 (s, 1H, H^e), 6.80 (dd, J = 12.7 Hz, J = 1.0 Hz, 1H, H^g), 5.96 (t, J = 12.3 Hz, 1H, H^h), 3.73 (t, J = 7.1 Hz, 2H, H^k), 3.23 (br s, 3H, H^b), 3.20 (br s, 3H, $H^{b'}$), 3.15 (s, 3H, H^j), 2.98, (br t, J = 7.0 Hz, 2H, H^l).

4a ^{13}C NMR (151 MHz, CD_3CN) δ 166.0 (C^c), 163.8 ($C^{c'}$), 161.6 (C^i), 153.2 (C^g), 152.8 (C^a), 146.7 (C^f), 138.5 (C^m), 135.8 (C^e), 129.9 ($C^{n/o/p}$), 129.7 ($C^{n/o/p}$), 127.9 ($C^{n/o/p}$), 104.6 (C^h), 97.0 (C^d), 61.7 (C^k), 37.6 (C^j), 35.0 (C^l), 28.5 ($C^{b'}$), 28.5 (C^b).

4a' ^{1}H NMR (600 MHz, CD_3CN) δ 12.53 (br s, 1H, H^{OH}), 7.52 (d, J = 12.1 Hz, 1H, H^i), 7.35-7.22 (m, 5H, H^n, H^o, H^p), 6.91 (d, J = 12.7 Hz, 1H, H^g) , 6.88 (s, 1H, H^e), 6.05 (t, J = 12.4 Hz, 1H, H^h), 3.73 (t, J = 7.1 Hz, 2H, H^k), 3.24 (br s, 3H, H^b), 3.20 (br s, 3H, $H^{b'}$), 3.20 (br s, 3H, H^j), 2.98, (br t, J = 7.0 Hz, 2H, H^l).

4a' ^{13}C NMR (151 MHz, CD_3CN) δ 162.2 (C^i), 153.6 (C^g), 146.6(C^f), 138.7 (C^m), 135.2 (C^e), 129.9 ($C^{n/o/p}$), 105.0 (C^h), 53.7 (C^k), 45.7 (C^j), 33.2 (C^l).

In solution (CD_3CN), **4** is present as 2 conformers, labelled **4a** and **4a'**, and isomer **4b** in a ratio of 3.0 : 1.0 : 0.09

HR-NSI-MS *m/z* 370.17388 $[M+H]^+$ requires 370.17613, 392.15567 $[M+Na]^+$ requires 392.15808.

UV-vis ($CHCl_3$): λ_{max}/nm 568

7.2 ^{1}H NMR spectrum of 4 in CD_3CN

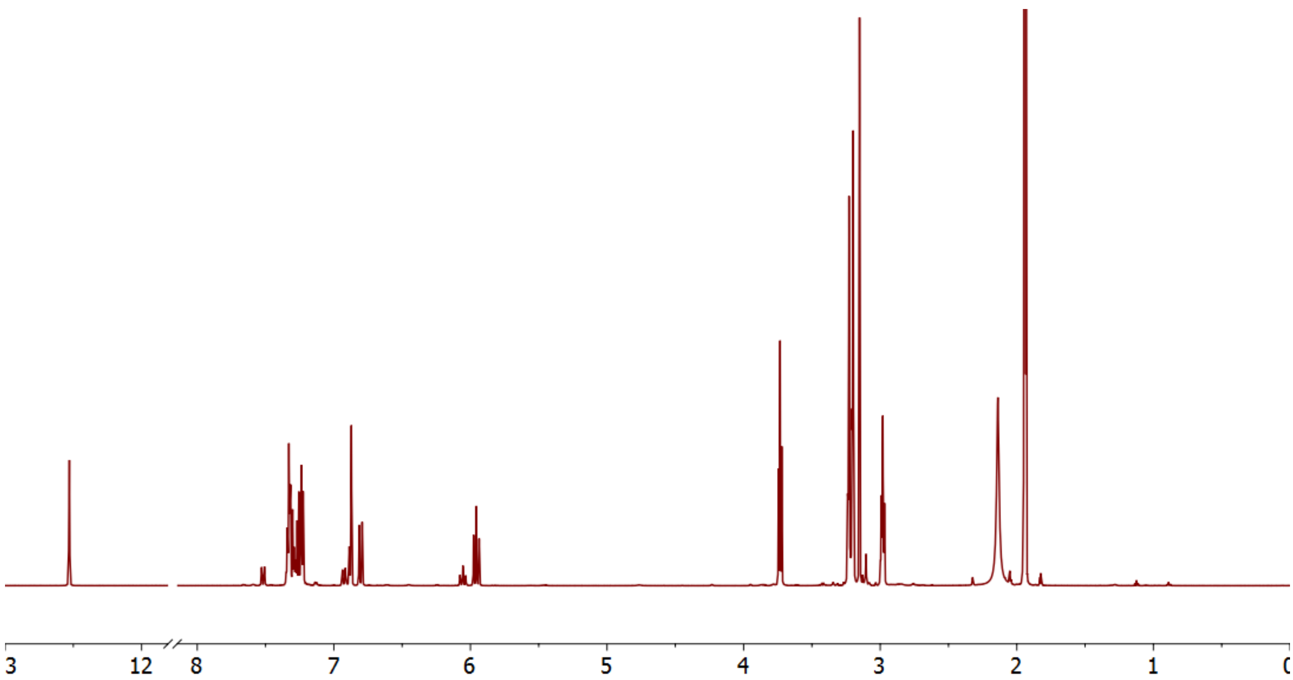

Figure S9. ^{1}H NMR (600 MHz, CD_3CN, 298 K) spectrum of **4**.

7.3 $^{13}C\{^{1}H\}$ NMR spectrum of 4 in CD_3CN

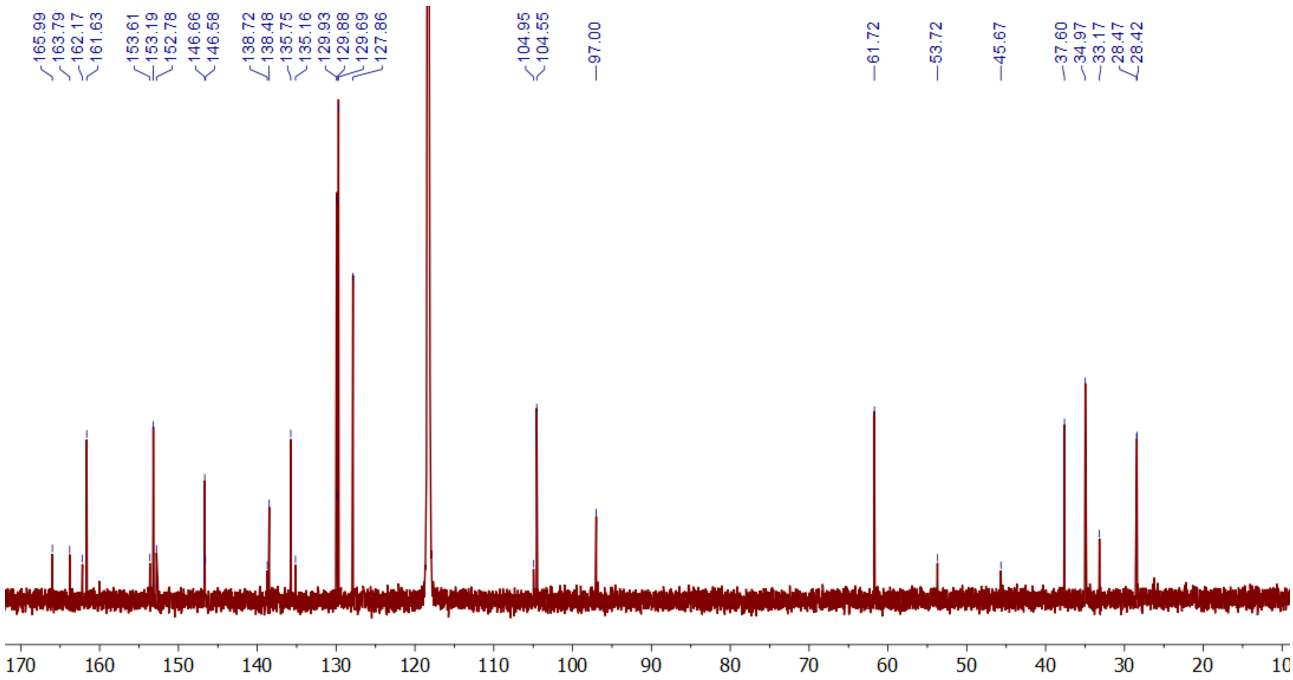

Figure S10. $^{13}C\{^{1}H\}$ NMR (151 MHz, CD_3CN, 298 K) spectrum of **4**.

8 Synthesis and characterization of compound 5a/5b

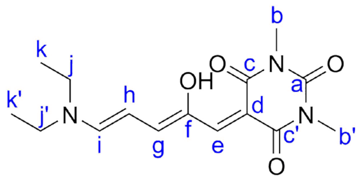

5a

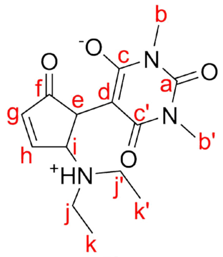

5b

8.1 Synthesis of 5a/5b

Diethylamine (120 µL, 1.16 mmol) was added to a stirred suspension of **S1** (234 mg, 1.00 mmol) in THF (2 mL), resulting in an immediate change in colour from yellow to intense purple. The solution was stirred at room temperature overnight. The solvent was removed under reduced pressure and the crude was purified by column chromatography (SiO_2, 5% MeOH in DCM). The product was triturated from the column fractions by the addition of hexane, cooled to -20 °C, collected by filtration and dried under vacuum. **5a** was isolated as a purple powder (65 mg, 0.21 mmol, 21%).

5a ^{1}H NMR (600 MHz, CD_3CN) δ 12.59 (br s, 1H, H^{OH}), 7.62 (d, J = 12.0 Hz, 1H, H^{i}), 7.01 (dd, J = 12.8 Hz, J = 0.9 Hz,1H, H^{g}), 6.87 (s, 1H, H^{e}), 6.15 (t, J = 12.4 Hz, 1H, H^{h}), 3.58 (q, J = 7.3 Hz, 2H, H^{j}), 3.55 (q, J = 7.3 Hz, 2H, $H^{j'}$), 3.23 (s, 3H, H^{b}), 3.20 (s, 3H, $H^{b'}$), 1.28 (t, J = 7.3 Hz, 3H, $H^{k'}$), 1.25 (t, J = 7.3 Hz, 3H, H^{k}).

5a ^{13}C NMR (151 MHz, CD_3CN) δ 165.9 (C^{c}), 163.9 ($C^{c'}$), 161.2 (C^{i}), 154.2 (C^{g}), 152.8 (C^{a}), 146.4 (C^{f}), 134.2 (C^{e}), 105.1 (C^{h}), 96.2 (C^{d}), 53.1 ($C^{j'}$), 45.4 (C^{j}), 28.4 ($C^{b'}$), 28.4 (C^{b}), 14.4 ($C^{k'}$), 12.7 (C^{k}).

In solution (CD_3CN), **5** is present as a mixture isomers **5a** and **5b**, in a ratio of 1.0 : 0.03.

HR-NSI-MS *m/z* 308.15941 $[M+H]^+$ requires 308.16048, 330.14131 $[M+Na]^+$ requires 330.14243.

UV-vis ($CHCl_3$): λ_{max}/nm 566

8.2 ^{1}H NMR spectrum of 5 in CD_3CN

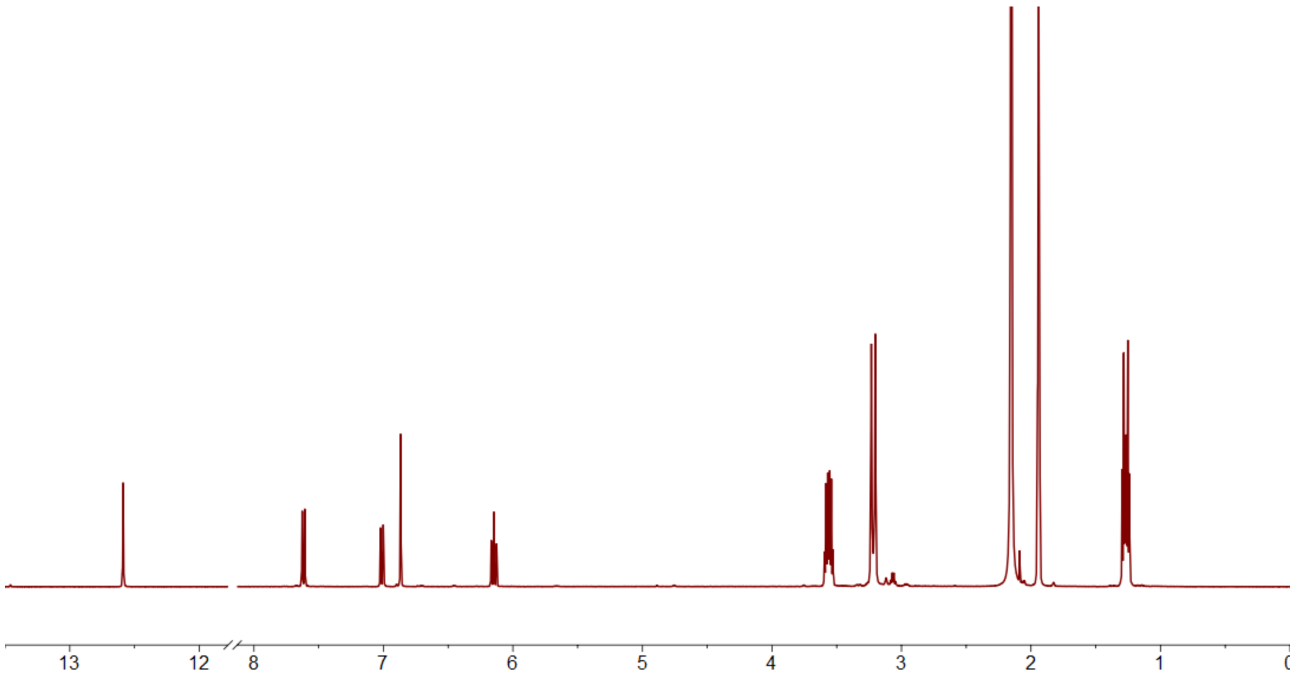

Figure S11. ^{1}H NMR (600 MHz, CD_3CN, 298 K) spectrum of **5**.

8.3 ^{13}C{^{1}H} NMR spectrum of 5 in CD_3CN

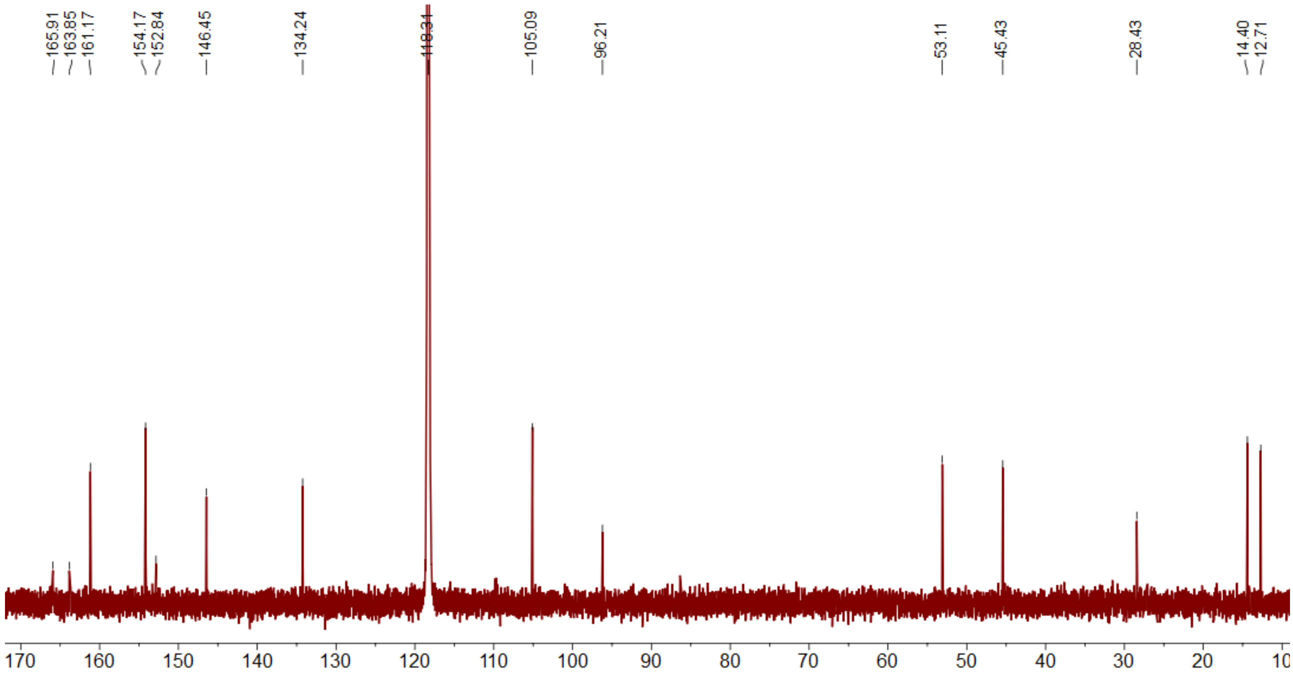

Figure S12. ^{13}C{^{1}H} NMR (151 MHz, CD_3CN, 298 K) spectrum of **5**.

9 Synthesis and characterization of compound 6a/6b

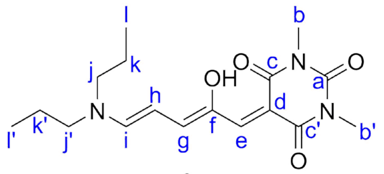

6a

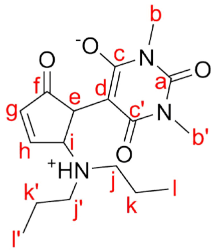

6b

9.1 Synthesis of 6a/6b

Dipropylamine (150 µl, 1.10 mmol) was added to a stirred suspension of **S1** (234 mg, 1.00 mmol) in THF (5 mL), resulting in an immediate change in colour from yellow to intense purple. The solution was stirred at room temperature overnight before Et_2O (10 mL) was added and the solution was cooled to -18°C to triturate the crude product, which was collected by filtration. The crude was purified by column chromatography (SiO_2, 7% MeOH in DCM) and dried under vacuum. **6a** was isolated as a purple solid (70 mg, 0.21 mmol, 21%).

6a ^{1}H NMR (600 MHz, CD_3CN) δ 12.58 (s, 1H, H^{OH}), 7.61 (d, J = 12.0 Hz, 1H, H^{i}), 7.00 (dd J = 12.7 Hz, J = 1.1 Hz, 1H, H^{g}), 6.88 (s, 1H, H^{e}), 6.14 (t, J = 12.4 Hz, 1H, H^{h}), 3.47 (t, J = 7.1 Hz, 2H, H^{j}), 3.45 (t, J = 6.4 Hz, 2H, $H^{j'}$), 3.23 (s, 3H, H^{b}), 3.20 (s, 3H, $H^{b'}$), 1.70 (m, 4H, H^{k}, $H^{k'}$), 0.96 (t, J = 7.4 Hz, 3H, H^{l}), 0.91 (t, J = 7.4 Hz, 3H, $H^{l'}$).

6a ^{13}C NMR (151 MHz, CD_3CN) δ 165.9 (C^{c}), 163.8 ($C^{c'}$), 162.0 (C^{i}), 154.0 (C^{g}), 152.8 (C^{a}), 146.5 (C^{f}), 134.6 (C^{e}), 105.1 (C^{h}), 96.4 (C^{d}), 60.0 ($C^{j'}$), 52.0 (C^{j}), 28.4 ($C^{b'}$), 28.4 (C^{b}), 22.6 ($C^{k'}$), 21.4 (C^{k}), 11.4 (C^{l}), 11.0 ($C^{l'}$).

In solution (CD_3CN), less than 1% of **6** is present as the cyclic isomer **6b**.

HR-NSI-MS *m/z* 336.19058 $[M+H]^+$ requires 336.19178, 358.17238 $[M+Na]^+$ requires 358.17373.

UV-vis ($CHCl_3$): λ_{max}/nm 567

9.2 ^{1}H NMR spectrum of 6 in CD_3CN

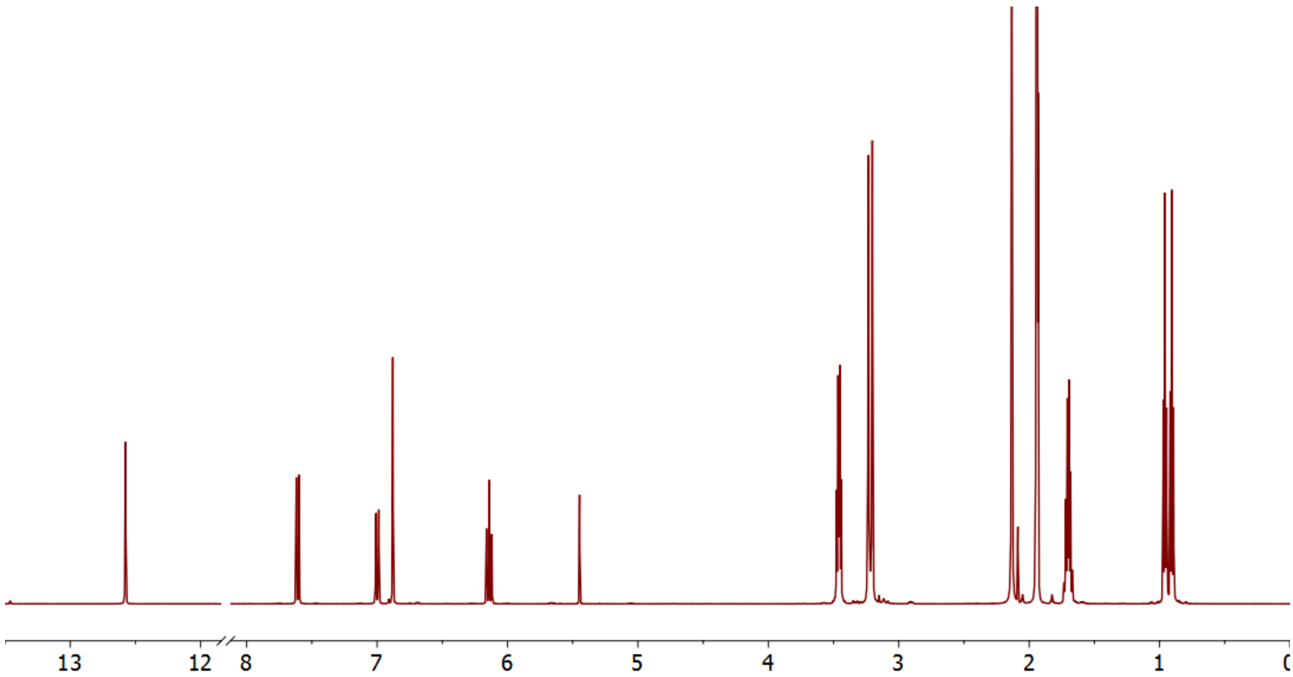

Figure S13. ^{1}H NMR (600 MHz, CD_3CN, 298 K) spectrum of **6**.

9.3 $^{13}C\{^1H\}$ NMR spectrum of 6 in CD_3CN

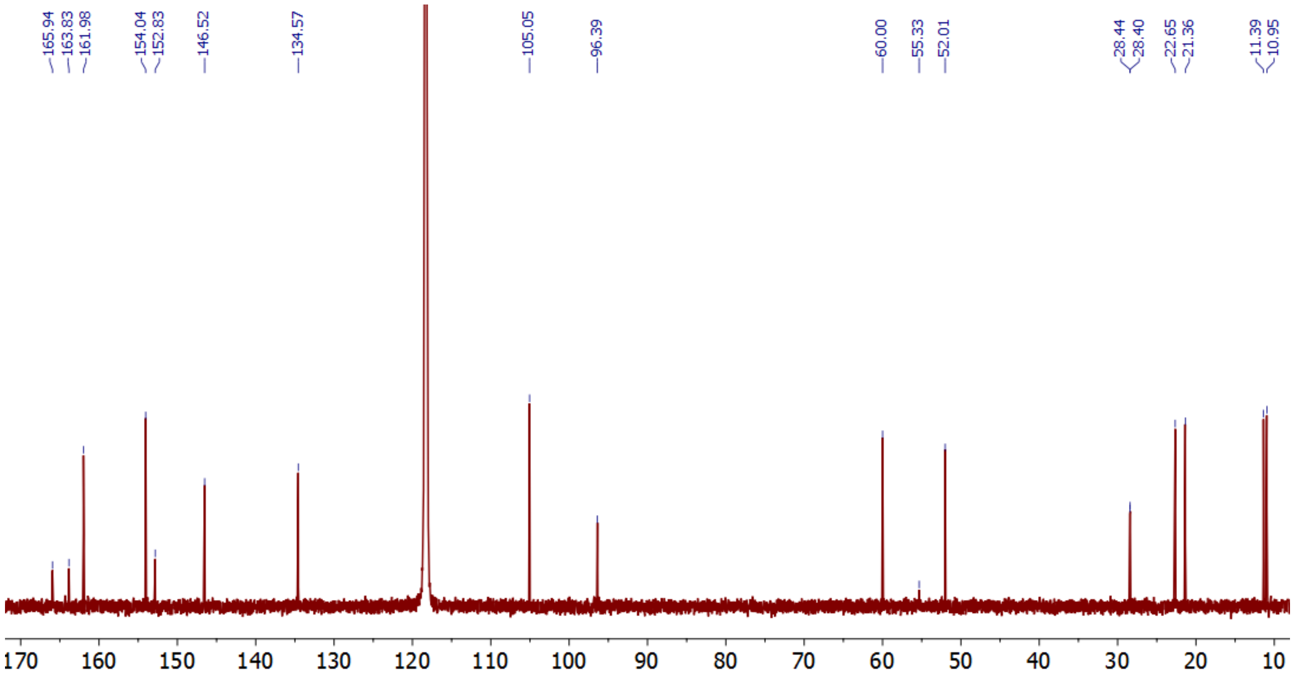

Figure S14. $^{13}C\{^1H\}$ NMR (151 MHz, CD_3CN, 298 K) spectrum of **6**.

10 Synthesis and characterization of compound 7a/7b

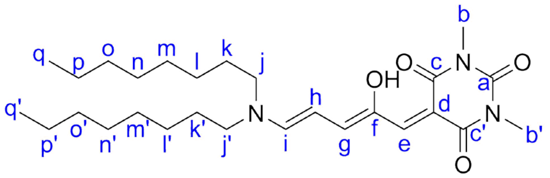

7a

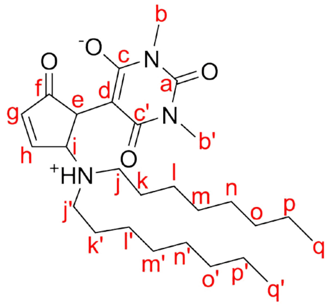

7b

10.1 Synthesis of 7a/7b

Dioctylamine (400 µL, 1.3 mmol) was added to a stirred solution of **S1** (234 mg, 1.0 mmol) in THF (5 mL), resulting in an immediate change of colour from yellow to intense purple. The solution was stirred at room temperature overnight, and solvent was removed under reduced pressure. The product was purified by column chromatography (SiO_2, 3% MeOH in DCM) to afford **7a** as a purple viscous oil (260 mg, 0.55 mmol, 55%).

7a ^{1}H NMR (600 MHz, CD_3CN) δ 12.57 (br s, 1H, H^{OH}), 7.59 (d, J = 12.0 Hz, 1H, H^{i}), 6.99 (dd, J = 12.7 Hz, J = 1.1 Hz, 1H, H^{g}), 6.87 (s, 1H, H^{e}), 6.12 (t, J = 12.4 Hz, 1H, H^{h}), 3.47 (m, 4H, H^{j}, H$^{j'}$), 3.23 (s, 3H, H^{b}), 3.20 (s, 3H, H$^{b'}$), 1.66 (m , 4H, H^{k}, H$^{k'}$), 1.37-1.24 (m, 20H, H^{l}, H$^{l'}$, H^{m}, H$^{m'}$, H^{n}, H$^{n'}$, H^{o}, H$^{o'}$, H^{p}, H$^{p'}$), 0.89 (m, 6H, H^{q}, H$^{q'}$).

7a ^{13}C NMR (151 MHz, CD_3CN) δ 165.9 (C^{c}), 163.8 (C$^{c'}$), 161.8 (C^{i}), 153.9 (C^{g}), 152.8 (C^{a}), 146.5 (C^{f}), 134.5 (C^{e}), 105.1 (C^{h}), 96.4 (C^{d}), 58.5 (C$^{j'}$), 50.6 (C^{j}), 32.5 (C^{o}, C$^{o'}$), 29.8 (C^{n}, C$^{n'}$), 29.8 (C^{m}/C$^{m'}$), 29.7 (C^{m}/C$^{m'}$), 29.3 (C$^{k'}$), 28.4 (C$^{b'}$), 28.4 (C^{b}), 27.9 (C^{k}), 27.4 (C^{l}), 26.9 (C$^{l'}$), 23.3 (C^{p}, C$^{p'}$), 14.4 (C^{q}, C$^{q'}$).

In solution (CD_3CN), less than 1% of **7** is present as the cyclic isomer **7b**.

HR-NSI-MS *m/z* 476.34792 $[M+H]^+$ requires 476.34881, 498.33000 $[M+Na]^+$ requires 498.33076.

UV-vis ($CHCl_3$): λ_{max}/nm 568

10.2 ^{1}H NMR spectrum of 7 in CD_3CN

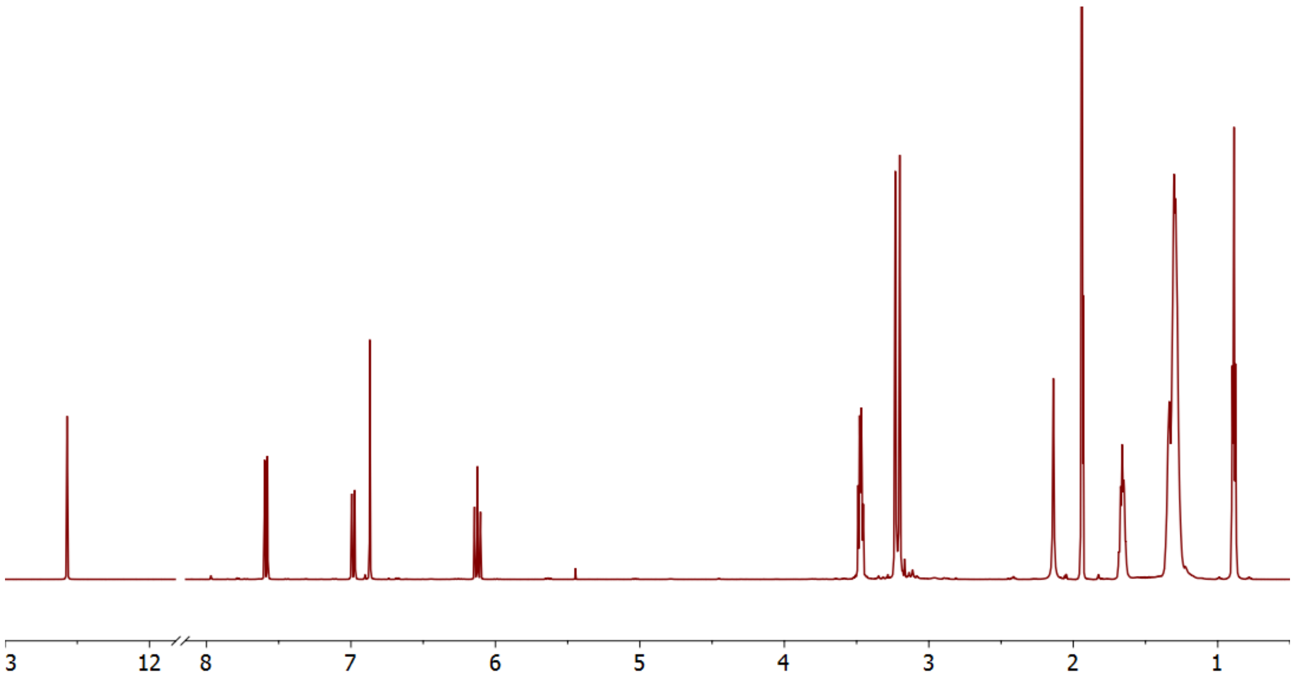

Figure S15. ^{1}H NMR (600 MHz, CD_3CN, 298 K) spectrum of **7**.

10.3 ^{13}C{^{1}H} NMR spectrum of 7 in CD_3CN

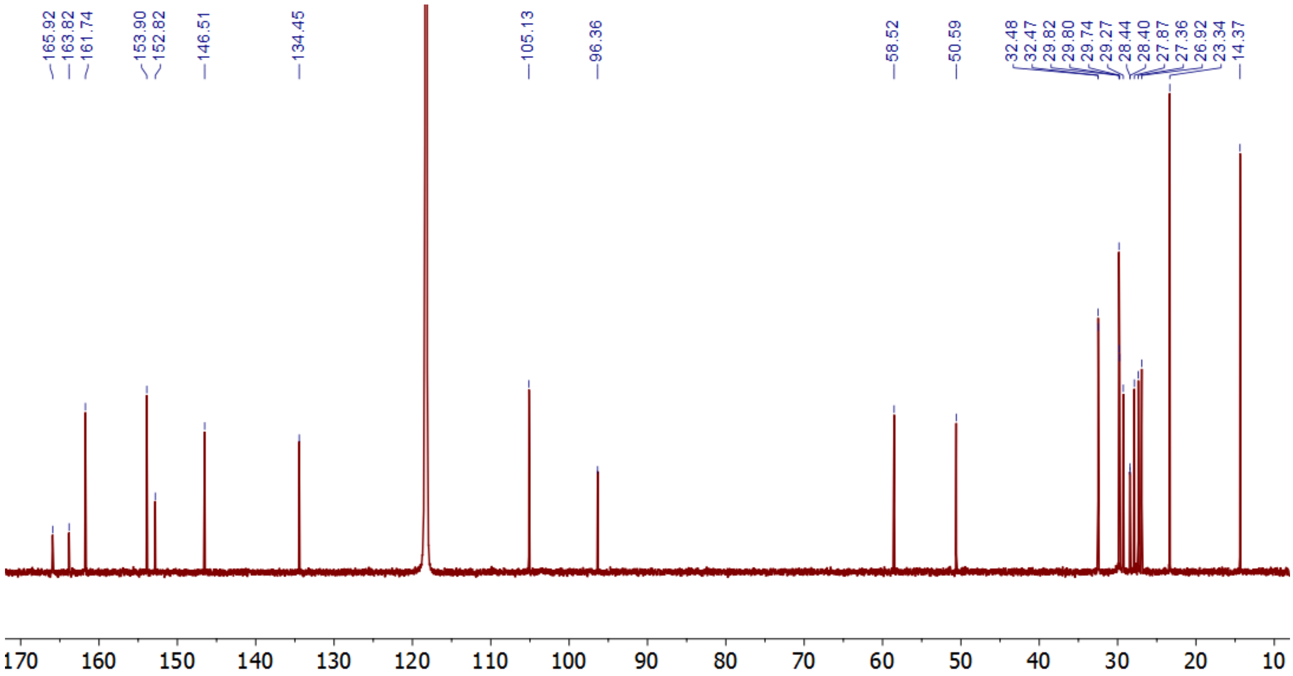

Figure S16. ^{13}C{^{1}H} NMR (151 MHz, CD_3CN, 298 K) spectrum of **7**.

11 Synthesis and characterization of compound 8a/8b

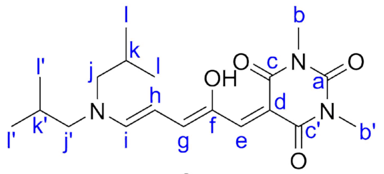

8a

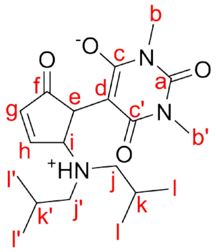

8b

11.1 Synthesis of 8a/8b

Diisobutylamine (3 mL, 17 mmol) was added to a stirred suspension of **S1** (234 mg, 1.00 mmol) in THF (3 mL) and stirred at room temperature overnight. The crude mixture was precipitated by the addition of hexane, and solvents were decanted. The crude was purified by column chromatography (SiO_2, 4% MeOH in DCM) and dried under vacuum. **8a** was isolated as a purple solid (40 mg, 0.11 mmol, 11%).

8a ^{1}H NMR (600 MHz, CD_3CN) δ 12.56 (s, 1H, H^{OH}), 7.60 (d, J = 12.1 Hz, 1H, H^{i}), 6.99 (dd J = 12.7 Hz, J = 1.0 Hz, 1H, H^{g}), 6.89 (s, 1H, H^{e}), 6.15 (t, J = 12.3 Hz, 1H, H^{h}), 3.33 (d, J = 7.6 Hz, 2H, H^{j}), 3.30 (d, J = 7.6 Hz, 2H, $H^{j'}$), 3.23 (s, 3H, H^{b}), 3.20 (s, 3H, $H^{b'}$), 2.16-2.09 (m, 1H, H^{k}), 2.06-2.00 (m, 1H, $H^{k'}$), 0.95 (d, J = 6.7 Hz, 6H, H^{l}), 0.90 (d, J = 6.7 Hz, 6H, $H^{l'}$).

8a ^{13}C NMR (151 MHz, CD_3CN) δ 165.9 (C^{c}), 163.8 ($C^{c'}$), 162.5 (C^{i}), 153.9 (C^{g}), 152.7 (C^{a}), 146.6 (C^{f}), 135.0 (C^{e}), 105.2 (C^{h}), 96.6 (C^{d}), 65.8 ($C^{j'}$), 57.5 (C^{j}), 28.4 (C^{b}), 28.4 ($C^{k'}$), 28.4 (C^{b}), 27.9 (C^{k}), 20.1 (C^{l}), 19.7 ($C^{l'}$).

In solution (CD_3CN), less than 1% of **8** is present as the cyclic isomer **8b**.

HR-NSI-MS *m/z* 364.22160 $[M+H]^{+}$ requires 364.22308, 386.20439 $[M+Na]^{+}$ requires 386.20503.

UV-vis ($CHCl_3$): λ_{max}/nm 569

11.2 ^{1}H NMR spectrum of 8 in CD_3CN

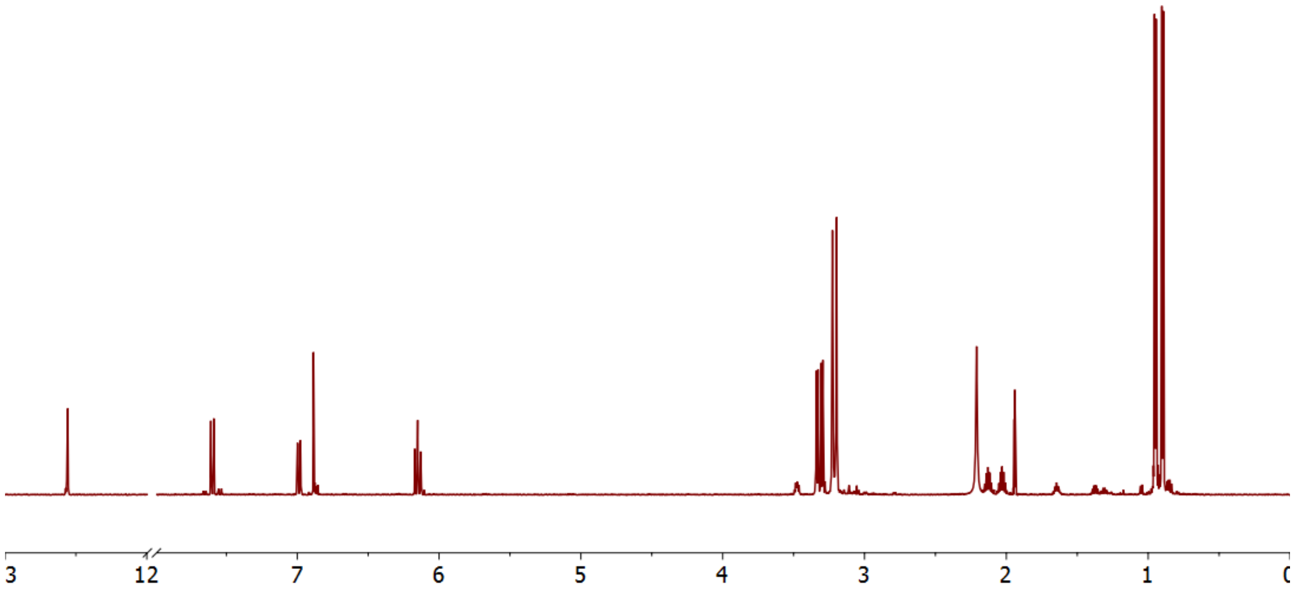

Figure S17. ^{1}H NMR (600 MHz, CD_3CN, 298 K) spectrum of **8**. This compound has several similar energy conformers in slow exchange on the NMR timescale.

11.3 ^{13}C{^{1}H} NMR spectrum of 8 in CD_3CN

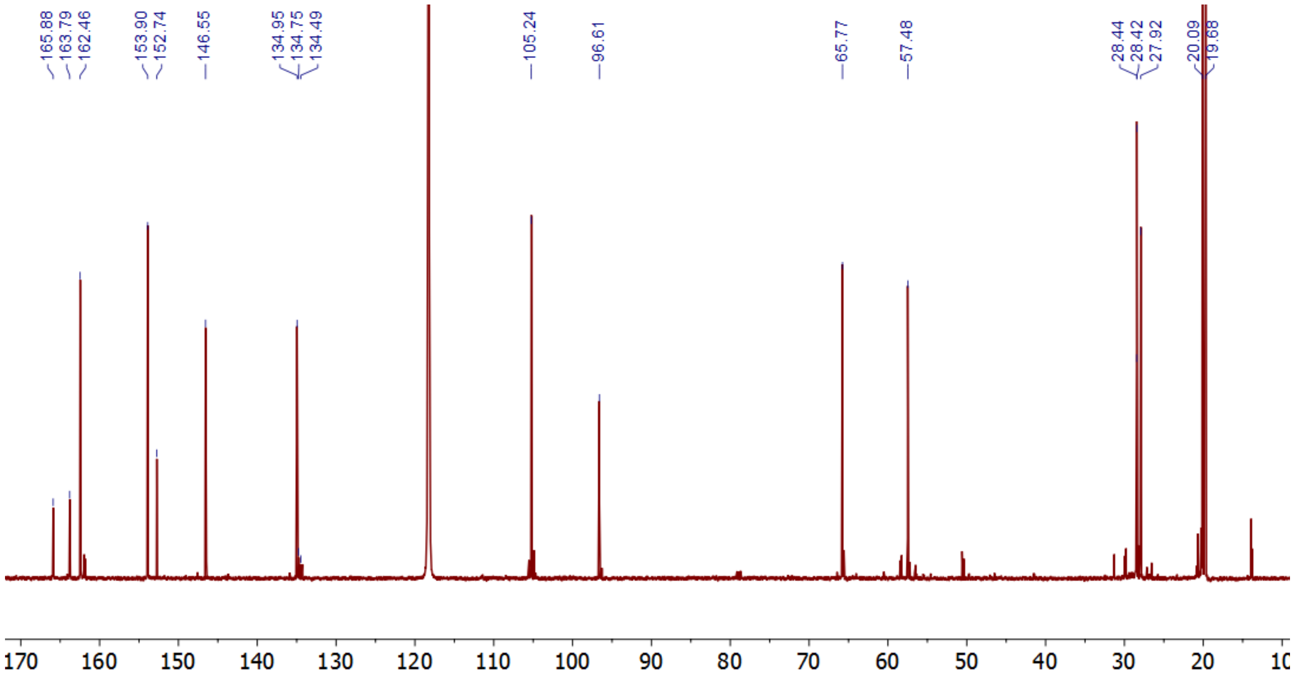

Figure S18. ^{13}C{^{1}H} NMR (151 MHz, CD_3CN, 298 K) spectrum of **8**.

12 Synthesis and characterization of compound 9a/9b

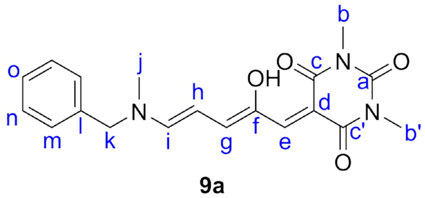

9a

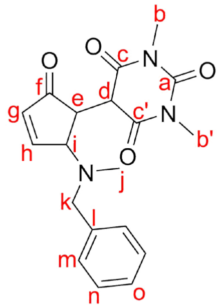

9b'

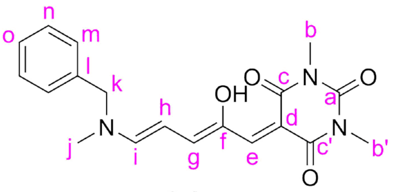

9a'

12.1 Synthesis of 9a/9b

N-Benzylmethylamine (130 µL, 1.00 mmol) was added to a stirred suspension of **S1** (234 mg, 1.00 mmol) in THF (2 mL), resulting in an immediate change in colour from yellow to intense purple. The solution was stirred at room temperature overnight before hexane (15 mL) was added to triturate the crude product, which was collected by filtration. The crude was purified by column chromatography (SiO_2, 5% MeOH in DCM). The product was triturated from the column fractions by the addition of hexane, cooled to -20 °C, collected by filtration and dried under vacuum. **9a** was isolated as a purple powder (35 mg, 0.10 mmol, 10%).

9a ^{1}H NMR (600 MHz, CD_3CN) δ 12.54 (s, 1H, H^{OH}), 7.79 (d, J = 12.0 Hz, 1H, H^{i}), 7.44-7.28 (m, 5H, H^{m}, H^{n}, H^{o}), 7.02 (d, J = 12.8 Hz, 1H, H^{g}), 7.00 (s, 1H, H^{e}), 6.09 (t, J = 12.3 Hz, 1H, H^{h}), 4.66 (s, 2H, H^{k}), 3.24 (s, 3H, H^{b}), 3.21 (s, 3H, $H^{b'}$), 3.05 (s, 3H, H^{j}).

9a ^{13}C NMR (151 MHz, CD_3CN) δ 166.1 (C^{c}), 163.8 ($C^{c'}$), 161.4 (C^{i}), 153.2 (C^{g}), 152.8 (C^{a}), 147.0 (C^{f}), 137.0 (C^{e}), 135.5 (C^{l}), 130.0 (C^{n}), 129.6 (C^{o}), 129.1 (C^{m}), 104.5 (C^{h}), 97.7 (C^{d}), 63.5 (C^{k}), 37.2 (C^{j}), 28.5 ($C^{b'}$), 28.5 (C^{b}).

9a' ^{1}H NMR (600 MHz, CD_3CN) δ 12.49 (s, 1H, H^{OH}), 7.68 (d, J = 12.1 Hz, 1H, H^{i}), 7.44-7.28 (m, 5H, H^{m}, H^{n}, H^{o}), 7.04-6.97 (m, 2H, H^{g}, H^{e}), 6.18 (t, J = 12.3 Hz, 1H, H^{h}), 4.71 (s, 2H, H^{k}), 3.28 (s, 3H, H^{j}), 3.24-3.21 (m, 6H, H^{b}, $H^{b'}$).

9b' ^{1}H NMR (600 MHz, CD_3CN) δ 3.09 (s, 6H, H^{b}, $H^{b'}$), 2.26 (s, 3H, H^{j}).

In solution (CD_3CN), **9** is present as a mixture of conformers **9a** and **9a'** and isomer **9b'**, in a ratio of 2.6 : 1.0 : 0.42.

HR-NSI-MS *m/z* 356.15910 $[M+H]^+$ requires 356.16048, 378.14088 $[M+Na]^+$ requires 378.14243.

UV-vis ($CHCl_3$): λ_{max}/nm 569

12.2 ^{1}H NMR spectrum of 9 in CD_3CN

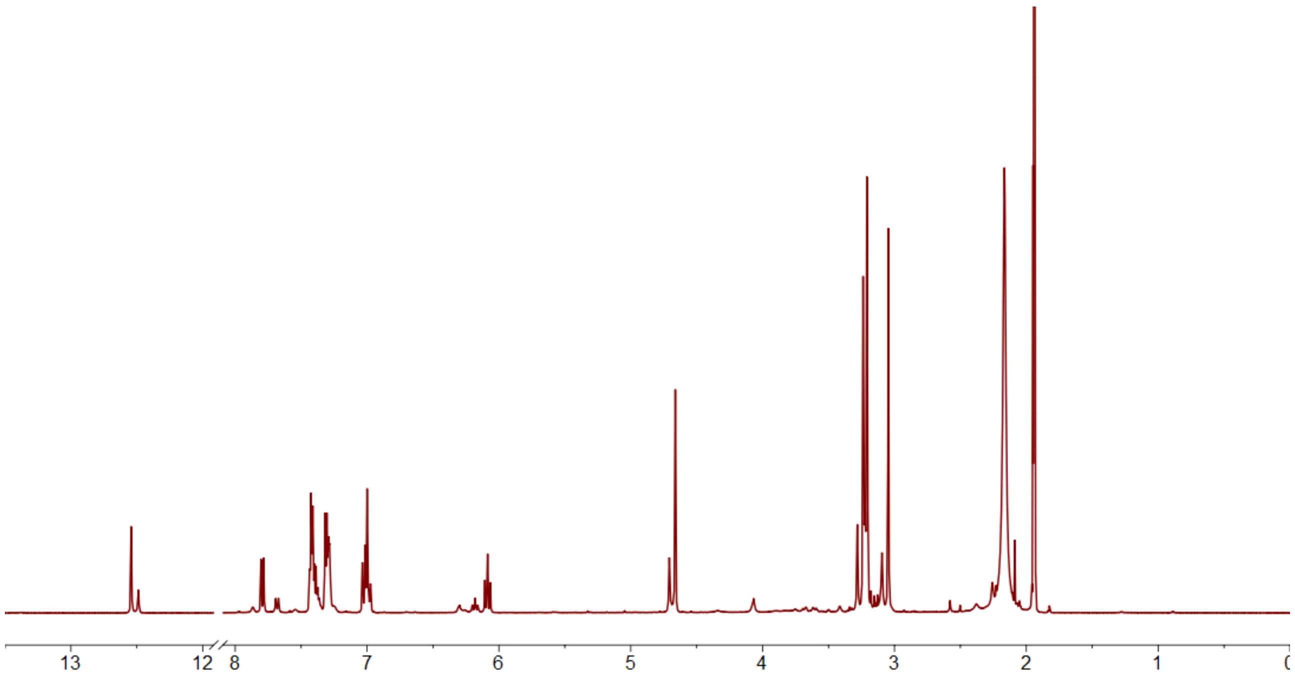

Figure S19. ^{1}H NMR (600 MHz, CD_3CN, 298 K) spectrum of **9**.

12.3 ^{13}C{^{1}H} NMR spectrum of 9 in CD_3CN

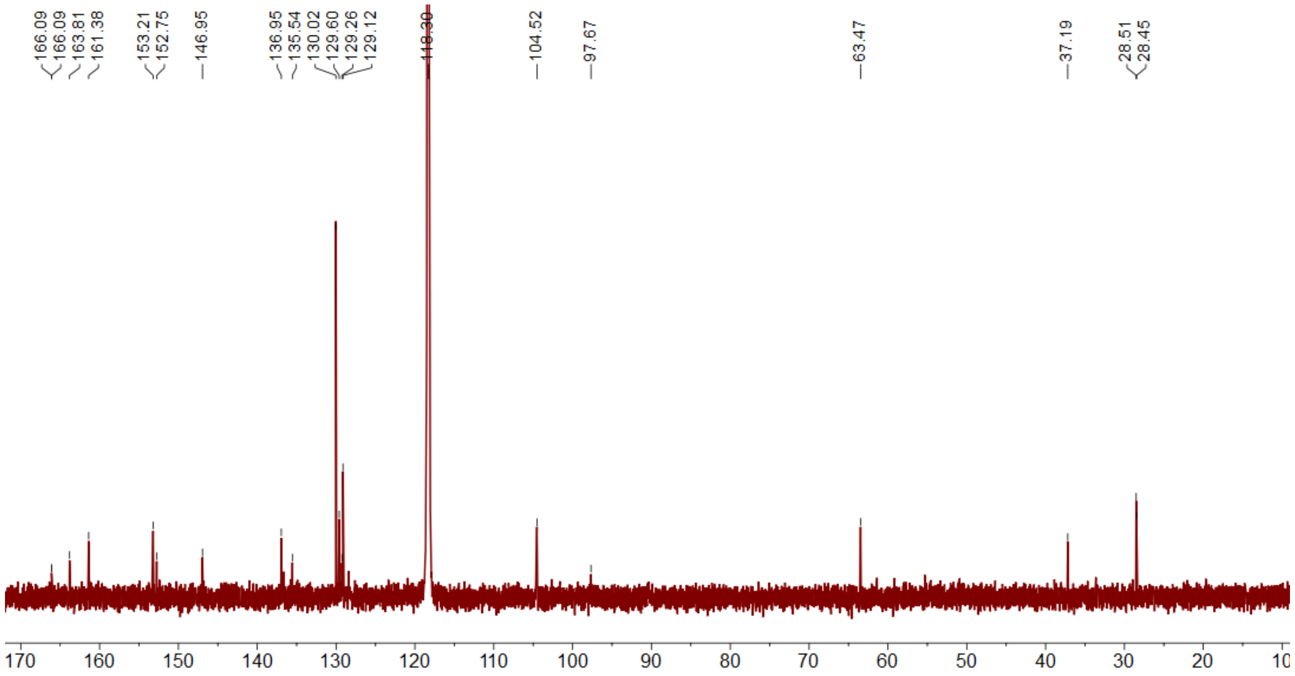

Figure S20. ^{13}C{^{1}H} NMR (151 MHz, CD_3CN, 298 K) spectrum of **9**.

12.4 ^{1}H-^{13}C HSQC NMR of 9 in CD_3CN

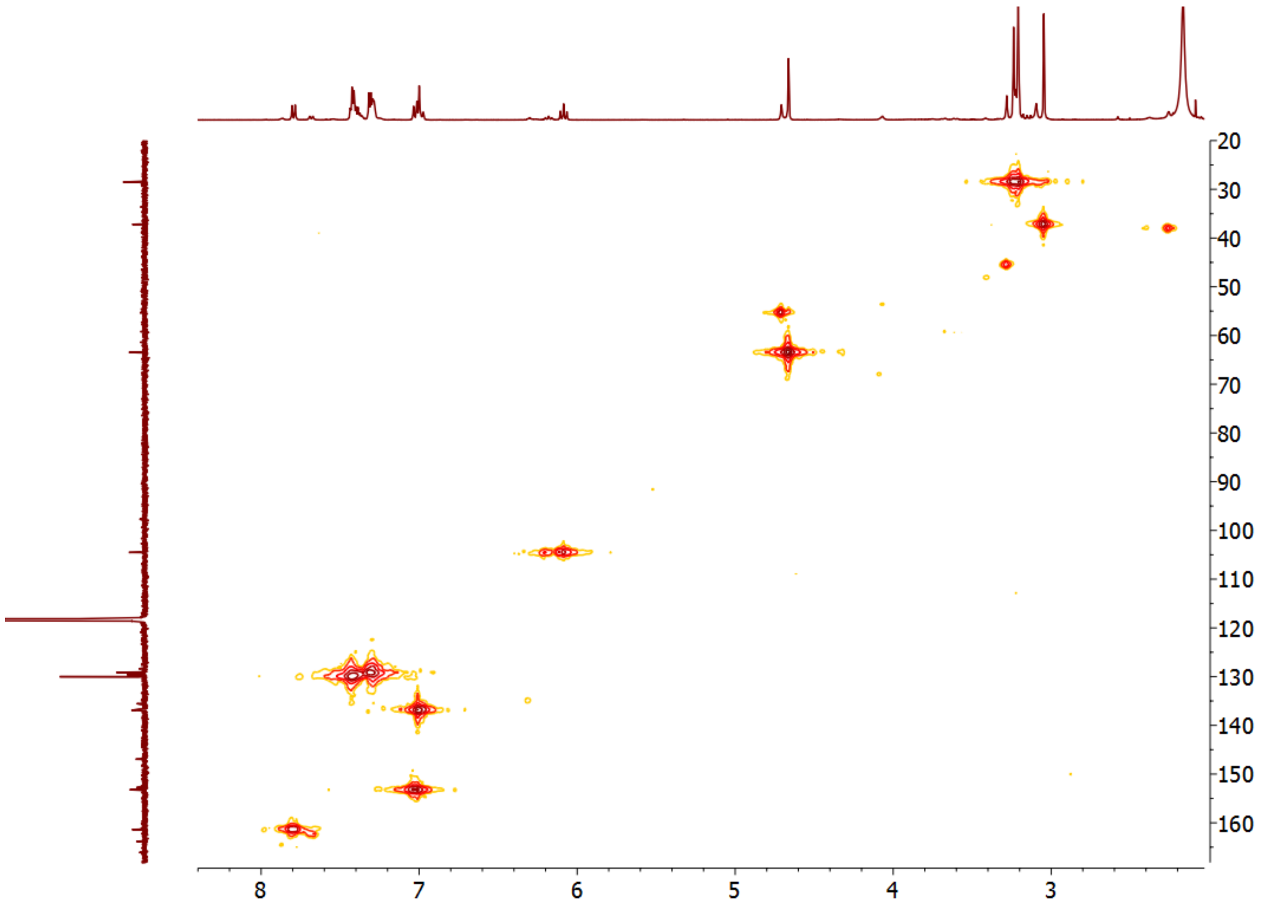

Figure S21. ^{1}H- ^{13}C HSQC NMR (600 MHz, CD_3CN, 298 K) spectrum of **9**.

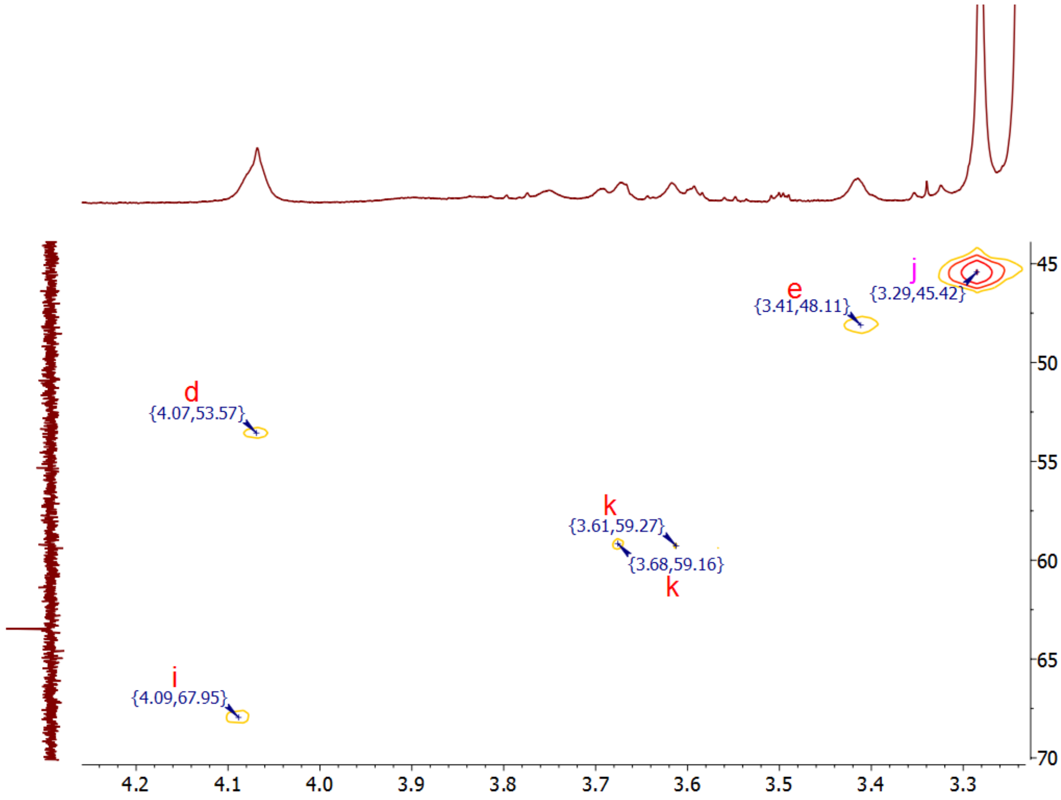

Figure S22. ^{1}H- ^{13}C HSQC NMR (600 MHz, CD_3CN, 298 K) spectrum of **9**, expansion of Figure S21, showing cross peaks for d, e and i, as observed for aromatic compound **14**, indicating it is present as the neutral *keto*-tautomer in solution. The signals are too weak to show in the ^{13}C (151 MHz, CD_3CN, 298 K) spectrum (vertical trace).

13 Synthesis and characterization of compound 10a/10b

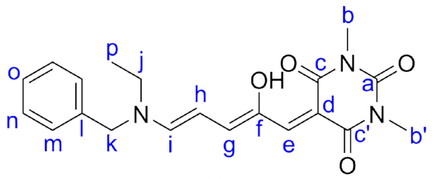

10a

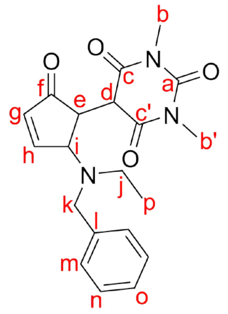

10b'

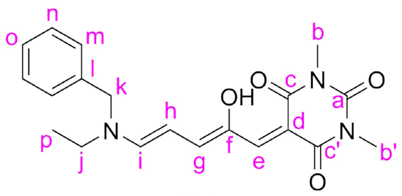

10a'

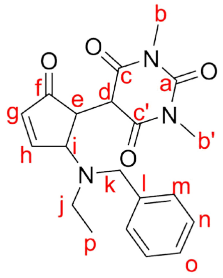

10b''

13.1 Synthesis of 10a/10b

N-Benzylethylamine (165 µL, 1.10 mmol) was added to a stirred suspension of **S1** (234 mg, 1.00 mmol) in THF (2 mL), resulting in an immediate change in colour from yellow to intense purple. The solution was stirred at room temperature overnight before hexane (15 mL) was added to triturate the crude product, which was collected by filtration. The crude was purified by column chromatography (SiO_2, 7% MeOH in DCM). The product was triturated from the column fractions by the addition of hexane, cooled to -20 °C, collected by filtration and dried under vacuum. **10a** was isolated as a purple powder (59 mg, 0.16 mmol, 16%).

10a ^{1}H NMR (600 MHz, CD_3CN) δ 12.55 (s, 1H, H^{OH}), 7.72 (d, J = 12.1 Hz, 1H, H^{i}), 7.45-7.26 (m, 5H, H^{m}, H^{n}, H^{o}), 7.04-6.98 (m, 2H, H^{e}, H^{g}), 6.15 (t, J = 12.4 Hz, 1H, H^{h}), 4.67 (s, 2H, H^{k}), 3.48 (q, J = 7.2 Hz, 2H, H^{j}), 3.24 (s, 3H, H^{b}), 3.21 (s, 3H, $H^{b'}$), 1.16 (t, J = 7.2 Hz, 3H, H^{p}).

10a ^{13}C NMR (151 MHz, CD_3CN) δ 166.0 (C^{c}), 163.8 ($C^{c'}$), 160.9 (C^{i}), 153.6 (C^{g}), 152.7 (C^{a}), 146.8 (C^{f}), 136.6 (C^{e}), 135.8 (C^{l}), 130.0 (C^{n}), 129.6 (C^{o}), 129.3 (C^{m}), 104.4 (C^{h}), 97.5 (C^{d}), 61.3 (C^{k}), 45.2 (C^{j}), 28.5 ($C^{b'}$), 28.5 (C^{b}), 12.3 (C^{p}).

10a' ^{1}H NMR (600 MHz, CD_3CN) δ 12.46 (s, 1H, H^{OH}), 7.75 (d, J = 12.1 Hz, 1H, H^{i}), 7.45-7.26 (m, 5H, H^{m}, H^{n}, H^{o}), 7.04-6.98 (m, 2H, H^{e}, H^{g}), 6.11 (t, J = 12.4 Hz, 1H, H^{h}), 4.75 (s, 2H, H^{k}), 3.58 (q, J = 7.2 Hz, 2H, H^{j}), 3.22 (s, 3H, H^{b}), 3.21 (s, 3H, $H^{b'}$), 1.28 (t, J = 7.2 Hz, 3H, H^{p}).

10a' ^{13}C NMR (151 MHz, CD_3CN) δ 166.0 (C^{c}), 163.8 ($C^{c'}$), 161.1 (C^{i}), 153.2 (C^{g}), 152.7 (C^{a}), 146.7 (C^{f}), 136.8 (C^{e}), 135.7 (C^{l}), 129.9 (C^{n}), 129.0 (C^{o}), 128.0 (C^{m}), 105.0 (C^{h}), 97.5 (C^{d}), 53.5 (C^{j}), 53.3 (C^{k}), 28.5 ($C^{b'}$), 28.5 (C^{b}) 14.4 (C^{p}).

10b' ^{1}H NMR (600 MHz, CD_3CN) δ 7.89 (dd, J = 6.0, J = 1.9 Hz, 1H, H^{h}), 6.29 (dd, J = 6.1, J = 2.0 Hz, 1H, H^{g}), 4.06-4.02 (m, 1H, H^{i}), 4.01 (d, J = 1.4 Hz, 1H, H^{d}), 3.75 (s, 2H, H^{k}), 3.72 (s, 2H, H$^{k'}$), 3.33 (dd, J = 4.0, J = 1.8 Hz, 1H, H^{e}), 3.04 (s, 6H, H^{b}, H$^{b'}$), 2.62-2.52 (m, 2H, H^{j}), 1.04 (t, J = 7.1 Hz, 3H, H^{p}). H^{m}, H^{n}, H^{o} are obscured by other signals; H$^{k'}$ is due to a minor conformer around the N-C^{i} bond (structure **10b''**).

10b' ^{13}C-from HSQC: δ 164.5 (C^{h}), 134.7 (C^{g}), 65.4 (C^{i}), 55.3 (C^{k}), 55.1 (C^{k}), 50.1 (C^{e}), 48.3 (C^{d}), 45.1 (C^{j}), 28.5 (C^{b}), 14.1 (C^{p}).

In solution (CD_3CN), **10** is present as a mixture of conformers **10a** and **10a'** and isomer **10b'**, in a ratio of 1.2 : 1.0 : 0.09.

HR-NSI-MS m/z 370.17421 $[M+H]^+$ requires 370.17613, 392.15616 $[M+Na]^+$ requires 392.15808.

UV-vis ($CHCl_3$): λ_{max}/nm 570

13.2 ^{1}H NMR spectrum of 10 in CD_3CN

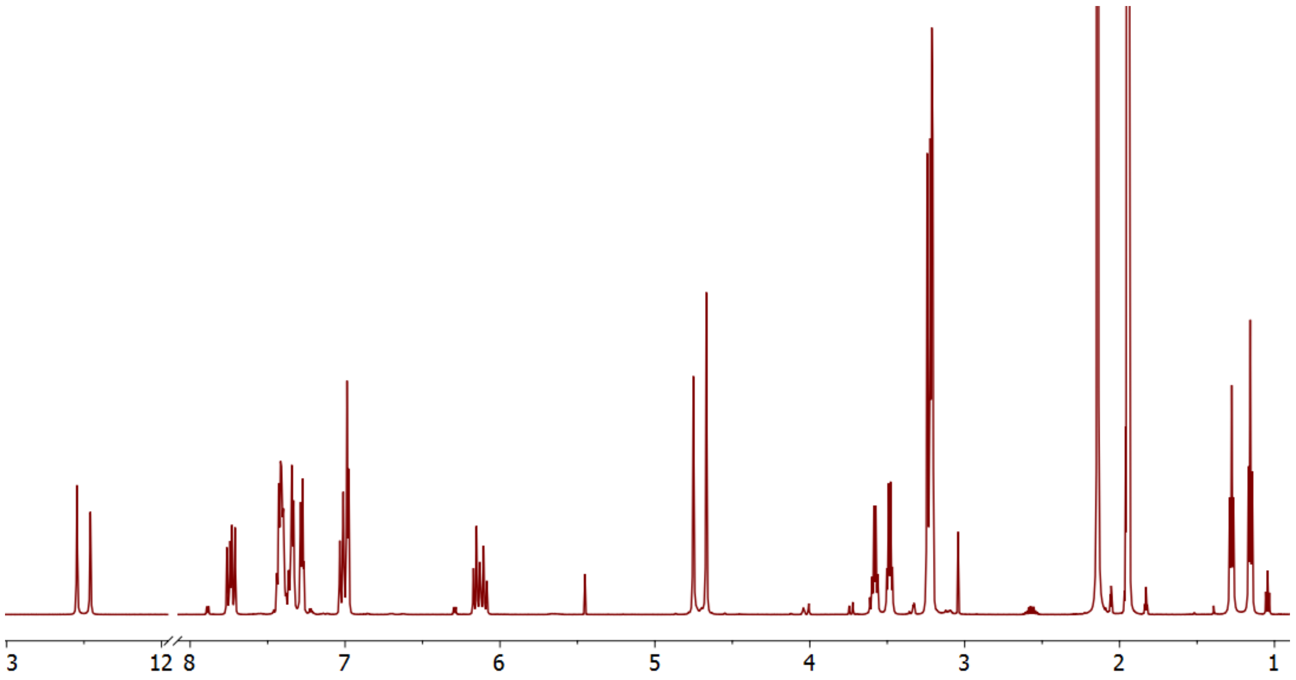

Figure S23. ^{1}H NMR (600 MHz, CD_3CN, 298 K) spectrum of **10**.

13.3 $^{13}C\{^1H\}$ NMR spectrum of 10 in CD_3CN

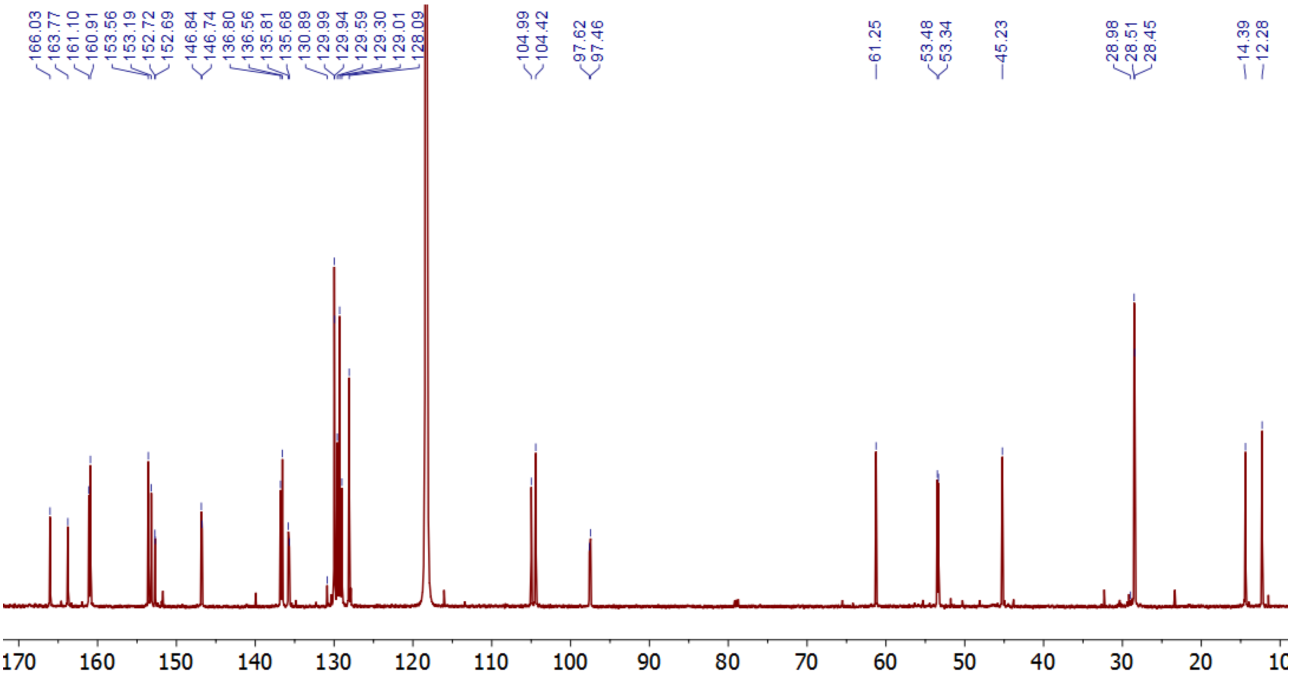

Figure S24. $^{13}C\{^1H\}$ NMR (151 MHz, CD_3CN, 298 K) spectrum of **10**.

13.4 ^{1}H-^{13}C HSQC NMR of 10 in CD_3CN

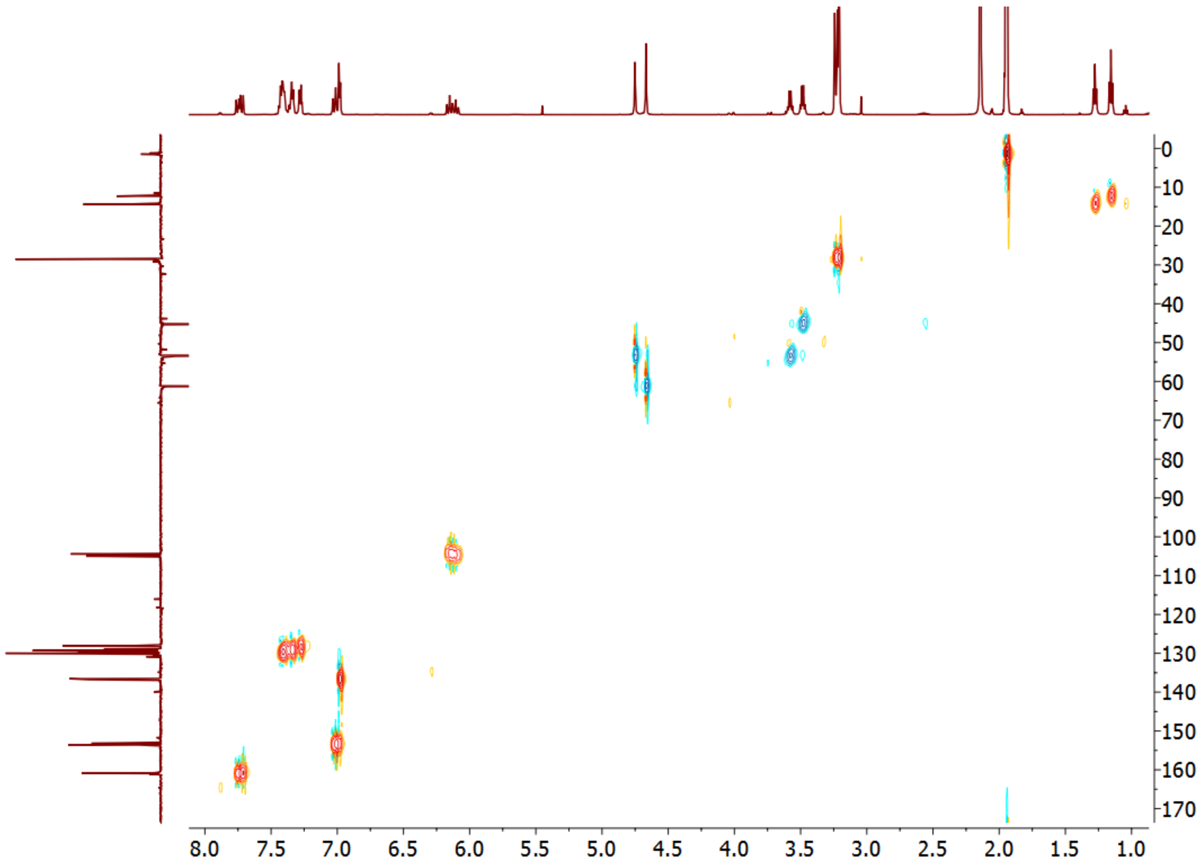

Figure S25. ^{1}H- ^{13}C HSQC NMR (600 MHz, CD_3CN, 298 K) spectrum of **10**.

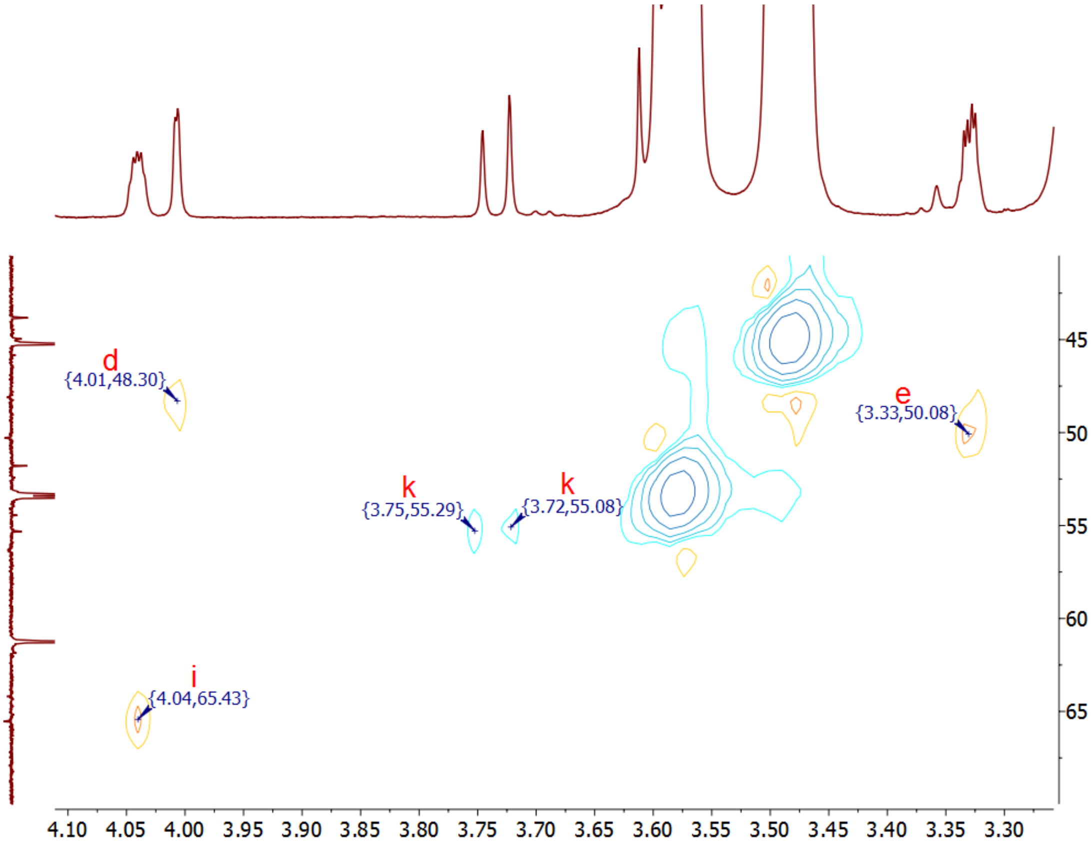

Figure S26. ^{1}H- ^{13}C HSQC NMR (600 MHz, CD_3CN, 298 K) spectrum of **10**, expansion of Figure S25, showing cross peaks for d, e and i, as observed for aromatic compound **14**, indicating it is present as the neutral *keto*-tautomer in solution. ^{13}C NMR signals are present in the DEPT-135 spectrum (151 MHz, CD_3CN, 298 K), used as vertical trace.

14 Synthesis and characterization of compound 11a/11b

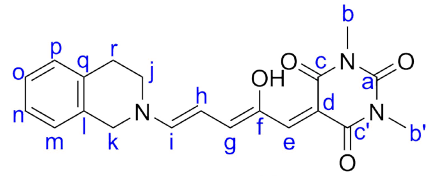

11a

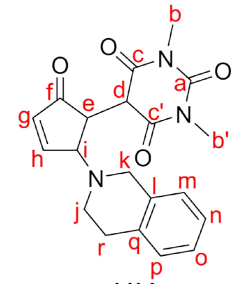

11b'

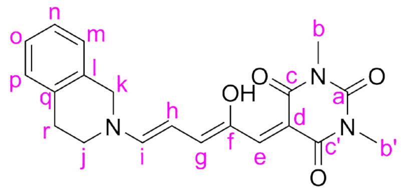

11a'

14.1 Synthesis of 11a/11b

Freshly distilled 1,2,3,4-tetrahydroisoquinoline (143 µL, 1.1 mmol) was added to a stirred solution of **S1** (234 mg, 1.0 mmol) in THF (4 mL), resulting in an immediate change of colour from yellow to intense purple. The solution was stirred at room temperature for 2 hours, during which a precipitate formed. The mixture was cooled to -20 °C before the crude product was collected by filtration. The product was recrystallized from chloroform/hexane to afford **11** as a purple solid (42 mg, 0.11 mmol, 11%).

11a ^{1}H NMR (600 MHz, CD_3CN) δ 12.57 (s, 1H, H^{OH}), 7.78-7.69 (m, 1H, H^{i}), 7.30-6.93 (m, 6H, H^{e}, H^{g}, H^{m}, H^{n}, H^{o}, H^{p}), 6.28-6.17 (m, 1H, H^{h}), 4.80 (s, 2H, H^{k}), 3.88-3.81 (m, 2H, H^{j}), 3.24 (s, 3H, H^{b}), 3.21 (s, 3H, $H^{b'}$), 3.03-2.98 (m, 2H, H^{r}).

11a ^{13}C-from HSQC: δ 160.2 (C^{i}), 153.3 (C^{g}), 136.3 (C^{e}), 129.4 ($C^{aromatic}$), 127.7 ($C^{aromatic}$), 127.0 ($C^{aromatic}$), 126.7 ($C^{aromatic}$), 104.2 (C^{h}), 56.5 (C^{k}), 45.9 (C^{j}), 29.4 (C^{r}), 28.0 (C^{b}).

11b' ^{1}H NMR (600 MHz, CD_3CN) δ 7.83 (br s, 1H, H^{h}), 7.64 (br s, 1H, $H^{h'}$), 7.30-6.93 (m, 4H, H^{m}, H^{n}, H^{o}, H^{p}), 6.31 (br s, 1H, H^{g}), 4.36 (br s, 1H, H^{i}), 4.24-3.46 (m, 5H, H^{e}, H^{k}, H^{j}), 3.36-2.68 (m, 10H, H^{b}, $H^{b'}$, H^{r}). This molecule exists as two conformers in slow exchange. $H^{h'}$ can be observed as a separate signal to H^{h}, all other signals are overlapped between conformers.

11a' ^{1}H NMR (600 MHz, CD_3CN) δ 12.57 (s, 1H, H^{OH}), 7.78-7.69 (m, 1H, H^{i}), 7.30-6.93 (m, 6H, H^{e}, H^{g}, H^{m}, H^{n}, H^{o}, H^{p}), 6.28-617 (m, 1H, H^{h}), 4.80 (s, 2H, H^{k}), 3.88-3.81 (m, 2H, H^{j}), 3.24 (s, 3H, H^{b}), 3.21 (s, 3H, $H^{b'}$), 3.03-2.98 (m, 2H, H^{r}).

11a' ^{13}C-from HSQC: δ 53.2 (C^{j}), 48.9 (C^{k}).

In solution (CD_3CN), **11** is present as a mixture of conformers **11a** and **11a',** but the spectral overlap in the ^{1}H NMR spectra prevents the determination of the ratio. The ratio linear: cyclic is roughly 1.0 : 0.70.

HR-NSI-MS *m/z* 368.15931 $[M+H]^+$ requires 368.16048, 390.14113 $[M+Na]^+$ requires 390.14243.

UV-vis ($CHCl_3$): λ_{max}/nm 570

14.2 ^{1}H NMR spectrum of 11 in CD_3CN

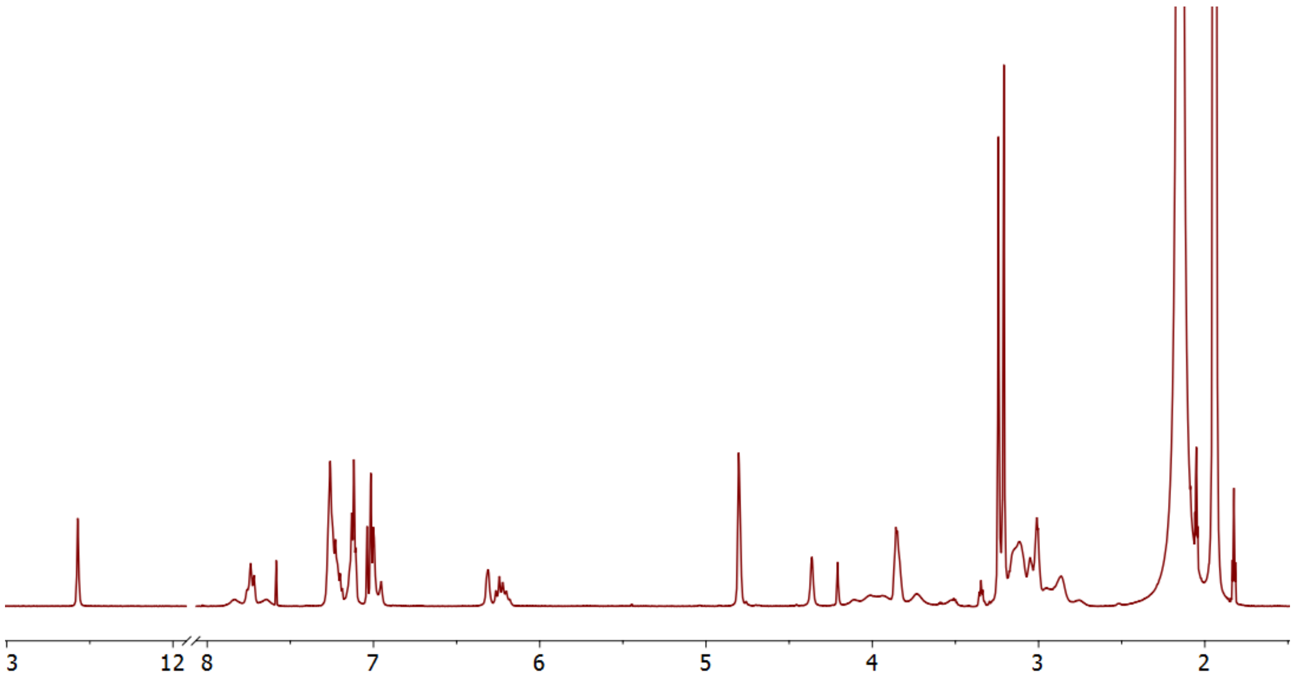

Figure S27. ^{1}H NMR (600 MHz, CD_3CN, 298 K) spectrum of **11.**

14.3 ^{1}H-^{13}C HSQC NMR spectrum of 11 in CD_3CN

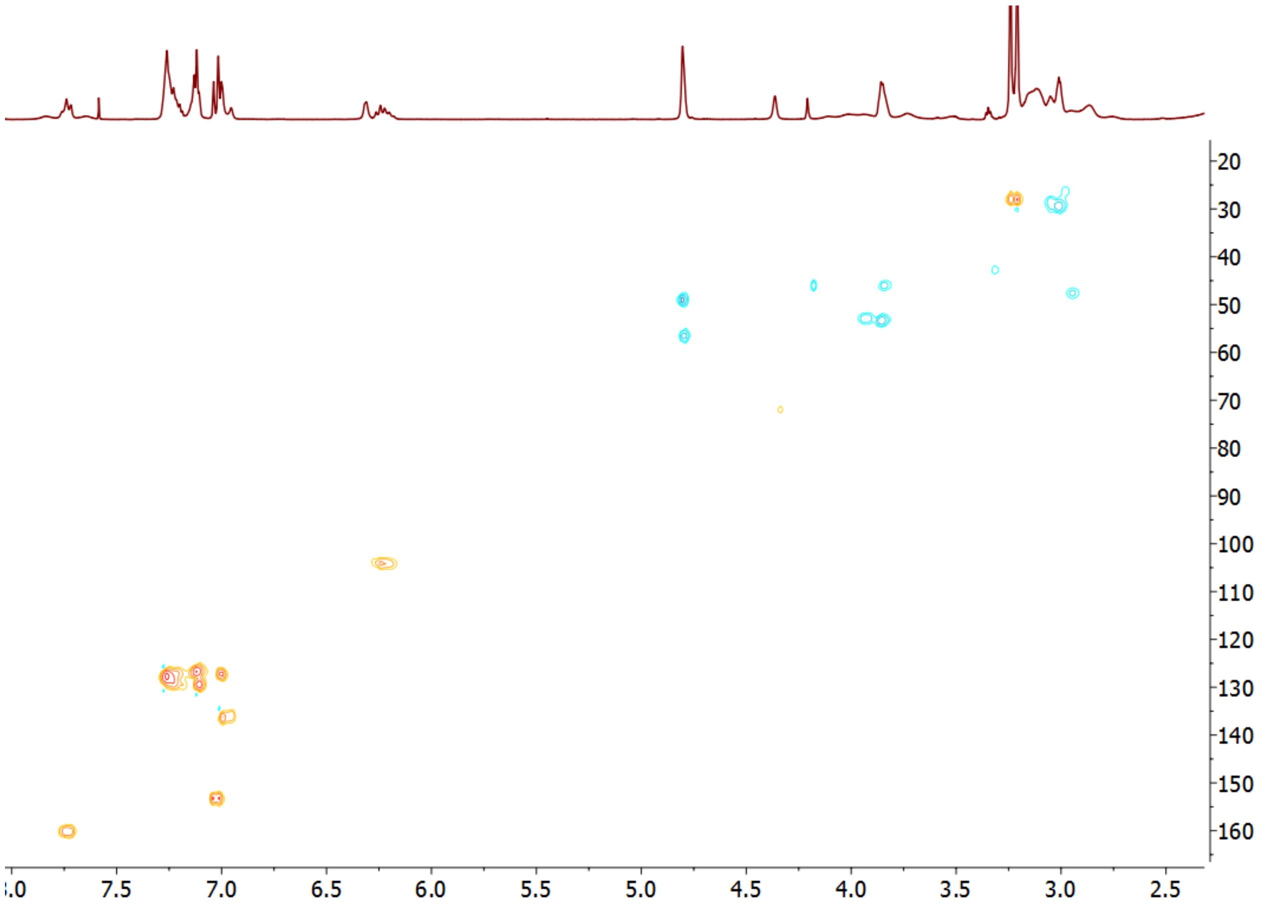

Figure S28. ^{1}H- ^{13}C HSQC NMR (600 MHz, CD_3CN, 298 K) spectrum of **11.**

15 Synthesis and characterization of compound 12a/12b

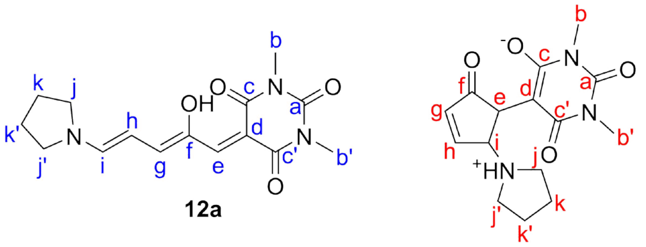

15.1 Synthesis of 12a/12b

Pyrrolidine (100 µL, 1.2 mmol) was added to a stirred solution of **S1** (234 mg, 1.0 mmol) in THF (4 mL), resulting in an immediate change of colour from yellow to intense purple. The solution was stirred at room temperature for 2 hours, during which a precipitate formed. The mixture was diluted with Et_2O (15 mL), the precipitate was collected by filtration, washed with cold THF (2 mL) and dried. The crude was purified by column chromatography (SiO_2, 5% MeOH in DCM). The product was triturated from the column fractions by the addition of hexane, cooled to -20 °C, collected by filtration and dried under vacuum. **12a** was isolated as a purple powder (43 mg, 0.14 mmol, 14%).

12a ^{1}H NMR (600 MHz, CD_3CN) δ 12.60 (s, 1H, H^{OH}), 7.79 (d, J = 11.8 Hz, 1H, H^i), 6.99 (dd, J = 12.9 Hz, J = 1.2 Hz, 1H, H^g), 6.84 (s, 1H, H^e), 6.02 (dd, J = 12.9 Hz, J = 11.8 Hz, 1H, H^h), 3.75 (t, J = 6.8 Hz, 2H, $H^{j'}$), 3.58 (m, 2H, H^j), 3.23 (s, 3H, H^b), 3.20 (s, 3H, $H^{b'}$), 2.06 (m, 2H, H^k), 1.97 (m, 2H, $H^{k'}$).

12a ^{13}C NMR (151 MHz, CD_3CN) δ 165.9 (C^c), 163.9 ($C^{c'}$), 158.6 (C^i), 153.3 (C^g), 152.9 (C^a), 146.5 (C^f), 133.8 (C^e), 106.5 (C^h), 96.1 (C^d), 55.4 ($C^{j'}$), 49.9 (C^j), 28.4 ($C^{b'}$), 28.4 (C^b), 25.4 ($C^{k'}$), 25.4 (C^k).

12b ^{1}H NMR (600 MHz, CD_3CN) δ 10.23 (NH^+), 7.69 (dd, J = 6.1 Hz, J = 1.9 Hz, 1H, H^h), 6.50 (dd, J = 6.1 Hz, J = 1.8 Hz, 1H, H^g), 4.46 (br s, 1H, H^i), 3.95 (d, 3.4 Hz, 1H, H^e), 3.11 (s, 6H, H^b, $H^{b'}$). H^j and H^k overlap with the analogous signals from **12a**.

In solution (CD_3CN), **12** is present as a mixture of isomers **12a** and **12b**, in a ratio of 1.0 : 0.20.

HR-NSI-MS *m/z*. 306.14500 $[M+H]^+$ requires 306.14537, 328.12694 $[M+Na]^+$ requires 328.12732.

UV-vis ($CHCl_3$): λ_{max}/nm 570

15.2 ^{1}H NMR spectrum of 12 in CD_3CN

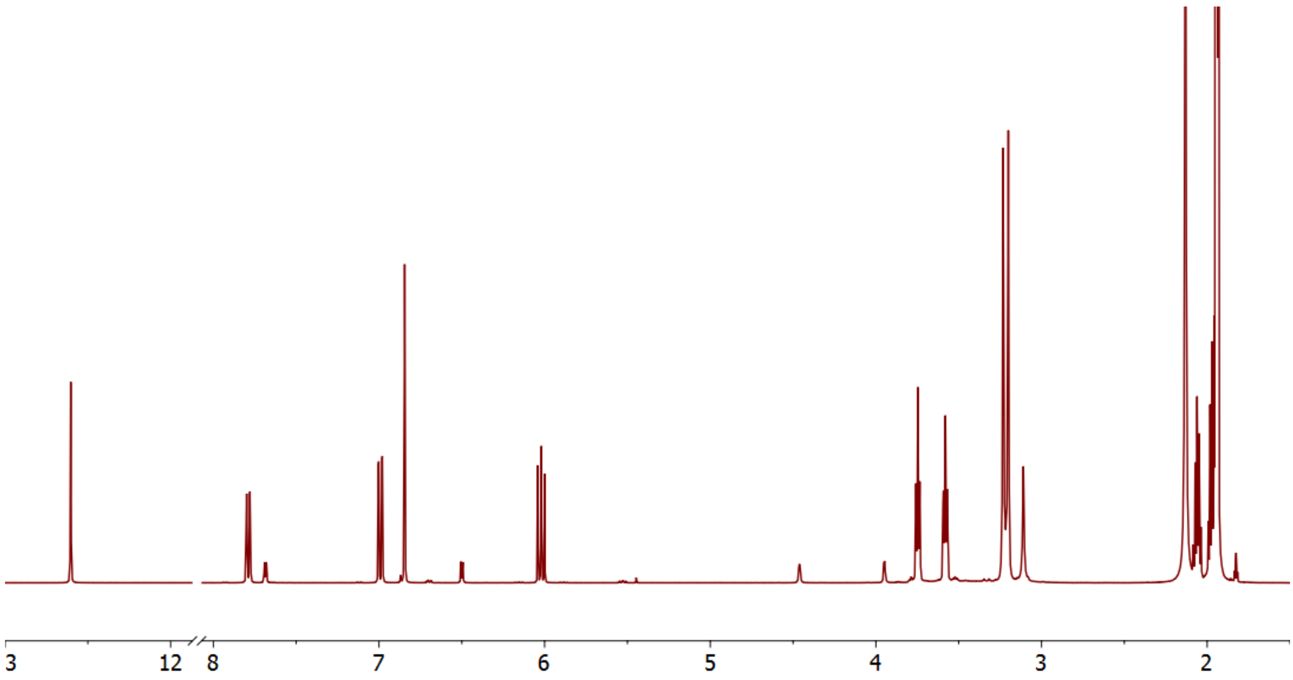

Figure S29. ^{1}H NMR (600 MHz, CD_3CN, 298 K) spectrum of **12**.

15.3 $^{13}C\{^{1}H\}$ NMR spectrum of 12 in CD_3CN

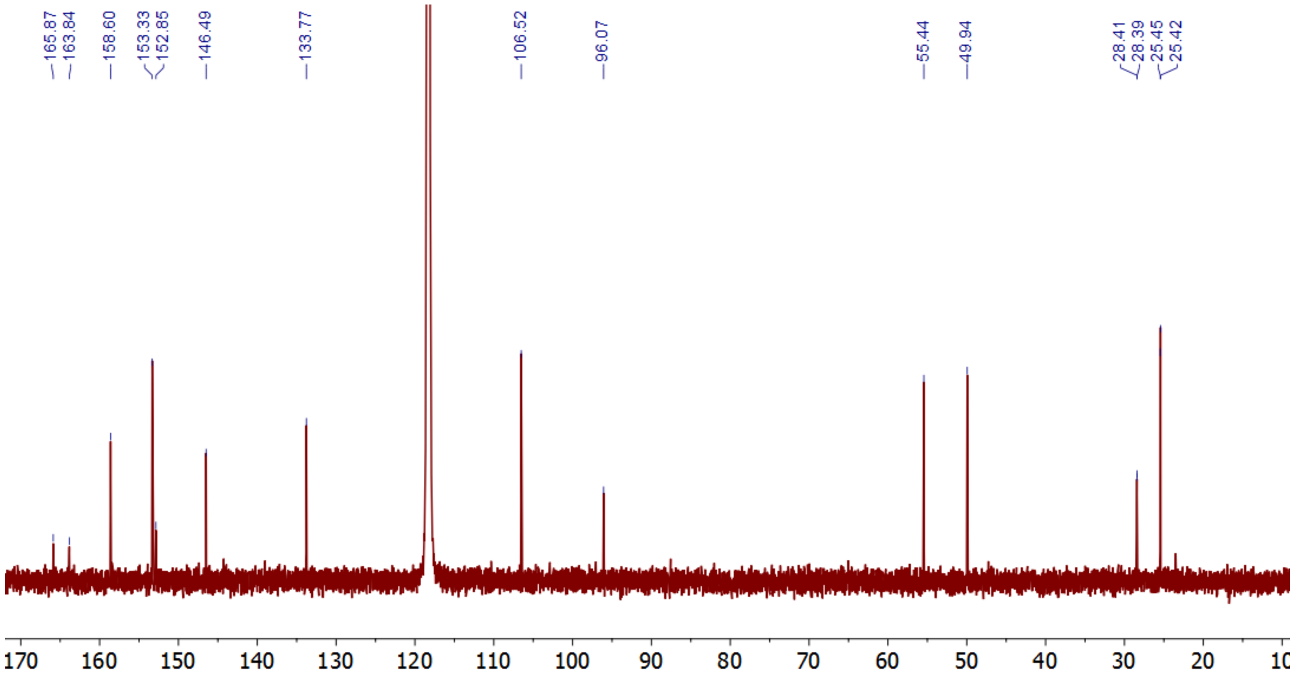

Figure S30. $^{13}C\{^{1}H\}$ NMR (151 MHz, CD_3CN, 298 K) spectrum of **12**.

16 Synthesis and characterization of compound 13a/13b

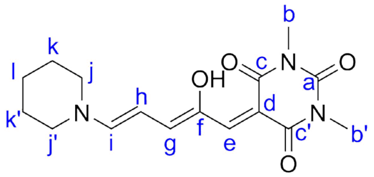

13a

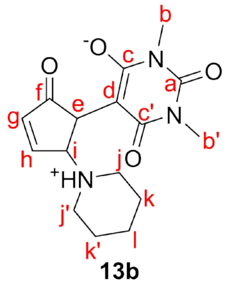

13b

16.1 Synthesis of 13a/13b

Freshly distilled piperidine (110 µL, 1.1 mmol) was added to a stirred solution of **S1** (234 mg, 1.0 mmol) in THF (4 mL), resulting in an immediate change of colour from yellow to intense purple. The solution was stirred at room temperature for 1 hour, during which a precipitate formed. The mixture was cooled to -20 °C before the crude product was collected by filtration. The product was purified by trituration from DCM by the addition of Et_2O (3x), and dried under vacuum. **13** was isolated as an off-white powder (122 mg, 0.38 mmol, 38%)

13a ^{1}H NMR (600 MHz, CD_3CN) δ 12.60 (s, 1H, H^{OH}), 7.60 (d, J = 11.9 Hz, 1H, H^i), 7.03 (dd, J = 12.8 Hz, J = 1.0 Hz, 1H, H^g), 6.83 (s, 1H, H^e), 6.22 (d, J = 12.3 Hz, 1H, H^h), 3.67-3.63 (m, 2H, H^j), 3.63-3.59 (m, 2H, $H^{j'}$), 3.23 (s, 3H, H^b), 3.20 (s, 3H, $H^{b'}$), 1.77-1.67 (m, 6H, H^k, $H^{k'}$, H^l).

13a ^{13}C From HSQC δ 160.6 (C^i), 154.1 (C^g), 132.8 (C^e), 104.3 (C^h), 57.7 ($C^{j'}$), 48.9 (C^j), 27.6 ($C^{b'}$), 27.6 (C^b), 27.2 (C^l), 25.4 ($C^{k'}$), 25.4 (C^k).

13b ^{1}H NMR (600 MHz, CD_3CN) δ 7.64 (d, J = 5.5 Hz, 1H, H^h), 6.41 (d, J = 3.3 Hz, 1H, H^g), 4.44 (br s, 1H, H^i), 3.83 (br s, 1H, H^e), 3.11 (s, 6H, H^b, $H^{b'}$).

In solution (CD_3CN), **13** is present as a mixture of isomers **13a** and **13b**, in a ratio of 1.0 : 0.53.

HR-NSI-MS m/z 320.16030 $[M+H]^+$ requires 320.16102, 342.14227 $[M+Na]^+$ requires 342.14297.

UV-vis ($CHCl_3$): λ_{max}/nm 566

16.2 ^{1}H NMR spectrum of 13 in CD_3CN

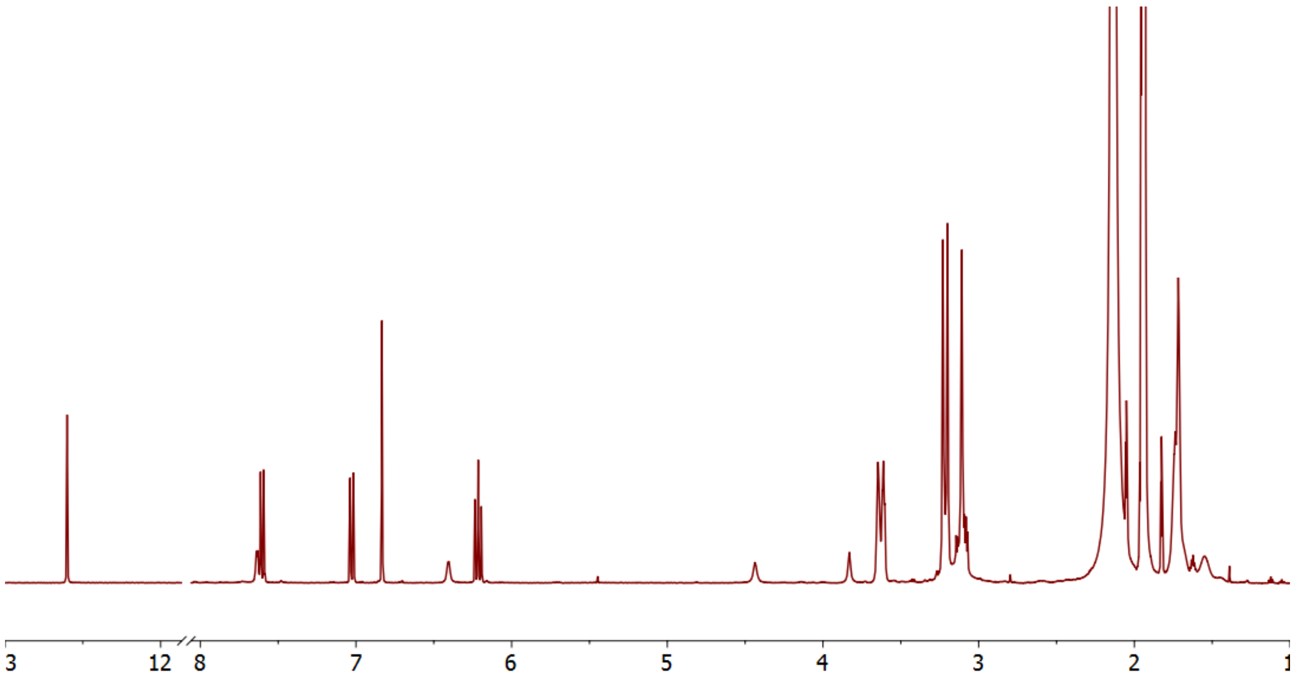

Figure S31. ^{1}H NMR (600 MHz, CD_3CN, 298 K) spectrum of **13.**

16.3 ^{1}H-^{13}C HSQC NMR of 13 in CD_3CN

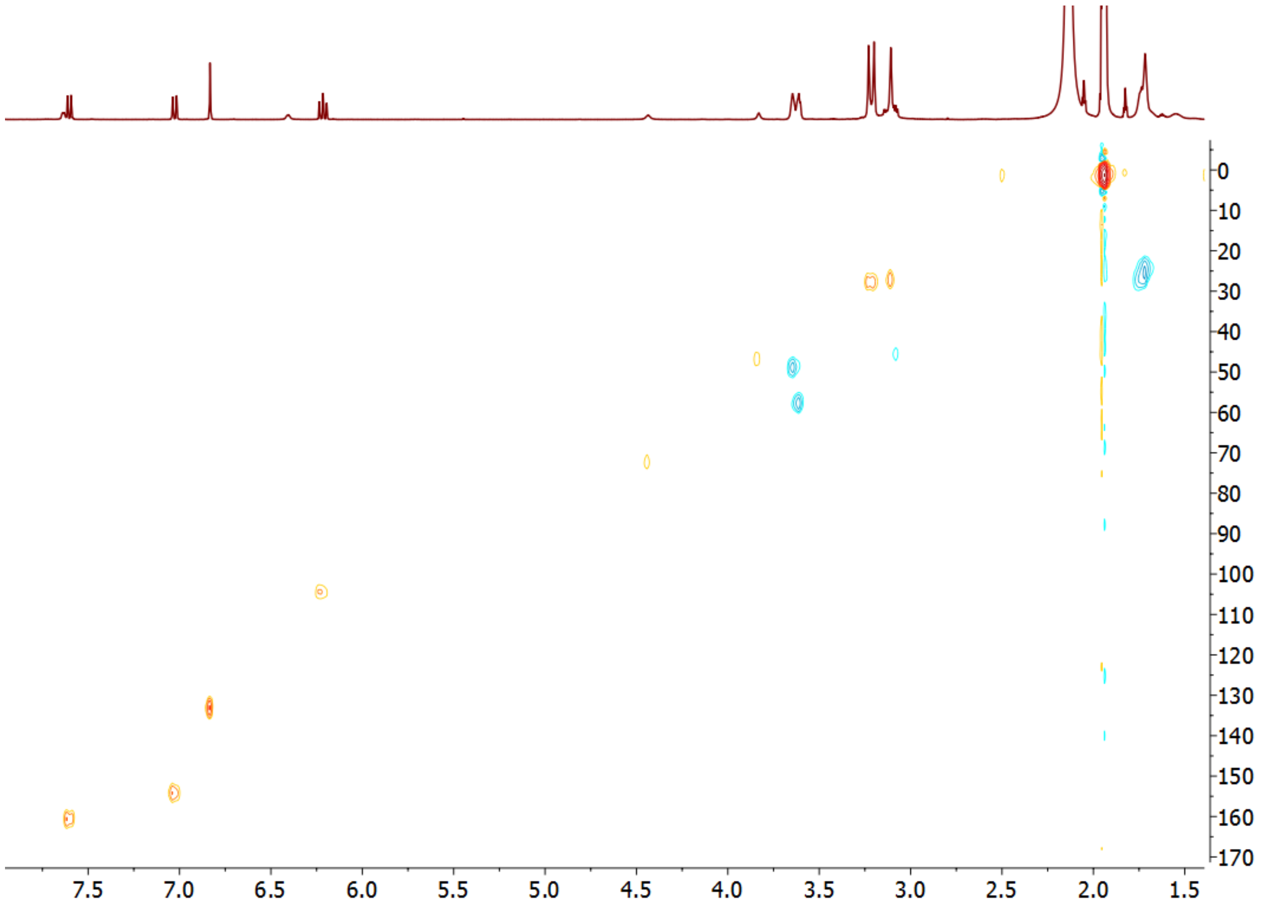

Figure S32. ^{1}H-^{13}C HSQC NMR (600 MHz, CD_3CN, 298 K) spectrum of **13.**

17 Synthesis and characterization of compound 14a/14b

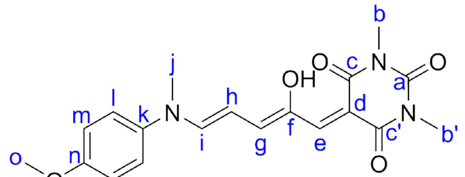

14a

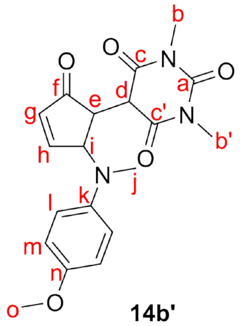

14b'

17.1 Synthesis of 14a/14b

4-Methoxy-*N*-methylaniline (136 mg, 0.99 mmol) was added to a stirred solution of **S1** (232 mg, 0.99 mmol) in THF (4 mL), resulting in an immediate change of colour from yellow to deep blue. The solution was stirred at room temperature overnight, during which a precipitate formed. The crude was collected by filtration and purified by column chromatography (SiO_2, 4% MeOH in DCM). The product was triturated from the column fractions by the addition of hexane, cooled to -20 °C, collected by filtration and dried under vacuum. **14a** was isolated as a blue powder (88 mg, 0.24 mmol, 24%).

14a ^{1}H NMR (600 MHz, CD_3CN) δ 12.45 (br s, 1H, H^{OH}), 7.74 (m, 1H, H^{i}), 7.28 (d, *J* = 9.0 Hz, 2H, Hl or Hm), 7.13 (br s, 1H, H^{g}), 7.04-6.98 (m, 3H, H^{l} or H^{m}, H^{e}), 6.19 (br s, 1H, H^{h}), 3.82 (s, 3H, H^{o}), 3.52 (s, 3H, H^{j}), 3.25 (s, 3H, H^{b}), 3.22 (s, 3H, H$^{b'}$).

^{13}C NMR signals for **14a** were too weak to be assigned.

14b' ^{1}H NMR (600 MHz, CD_3CN) δ 7.75 (dd, *J* = 5.9 Hz, *J* = 2.0 Hz, 1H, H^{h}), 6.78-6.74 (m, 4H H^{l}, H^{m}), 6.38 (dd, *J* = 5.9 Hz, *J* = 2.3 Hz, 1H, H^{g}), 5.14 (m, 1H, H^{i}), 4.00 (d, *J* = 1.3 Hz, 1H, H^{d}), 3.70 (s, 3H, H^{o}), 3.42 (dd, *J* = 4.0 Hz, *J* = 1.6 Hz, 1H, H^{e}), 3.17 (s, 3H, H^{b}), 2.86 (s, 3H, H$^{b'}$), 2.60 (s, 3H, H^{j}).

14b' ^{13}C NMR (151 MHz, CD_3CN) δ 204.0 (C^{f}), 168.4 (C$^{c'}$), 167.9 (C^{c}), 165.2 (C^{h}), 154.0 (C^{n}), 151.9 (C^{a}), 144.6 (C^{k}), 134.5 (C^{g}), 117.7 (C^{l} or C^{m}), 115.3 (C^{l} or C^{m}), 66.9 (C^{i}), 56.0 (C^{o}), 48.3 (C^{e}), 47.8 (C^{d}), 33.2 (C^{j}), 28.9 (C^{b}), 28.8 (C$^{b'}$).

In solution (CD_3CN), **14** is present as a mixture of isomers **14a** and **14b'**, in a ratio of 0.2 : 1.0.

HR-NSI-MS *m/z* 372.15384 [M+H]$^{+}$ requires 372.15540, 394.13560 [M+Na]$^{+}$ requires 394.13734

UV-vis ($CHCl_3$): λ_{max}/nm 588

17.2 ^{1}H NMR spectrum of 14 in CD_3CN

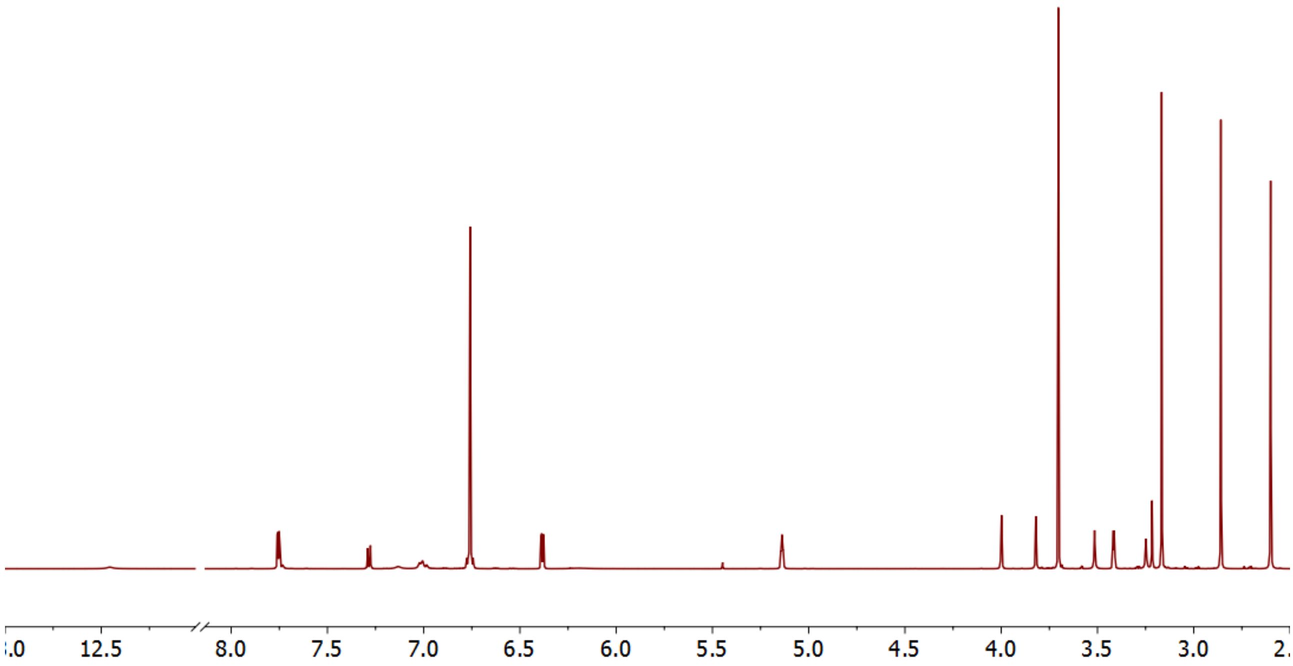

Figure S33. ^{1}H NMR (600 MHz, CD_3CN, 298 K) spectrum of **14**.

17.3 $^{13}C\{^{1}H\}$ NMR spectrum of 14 in CD_3CN

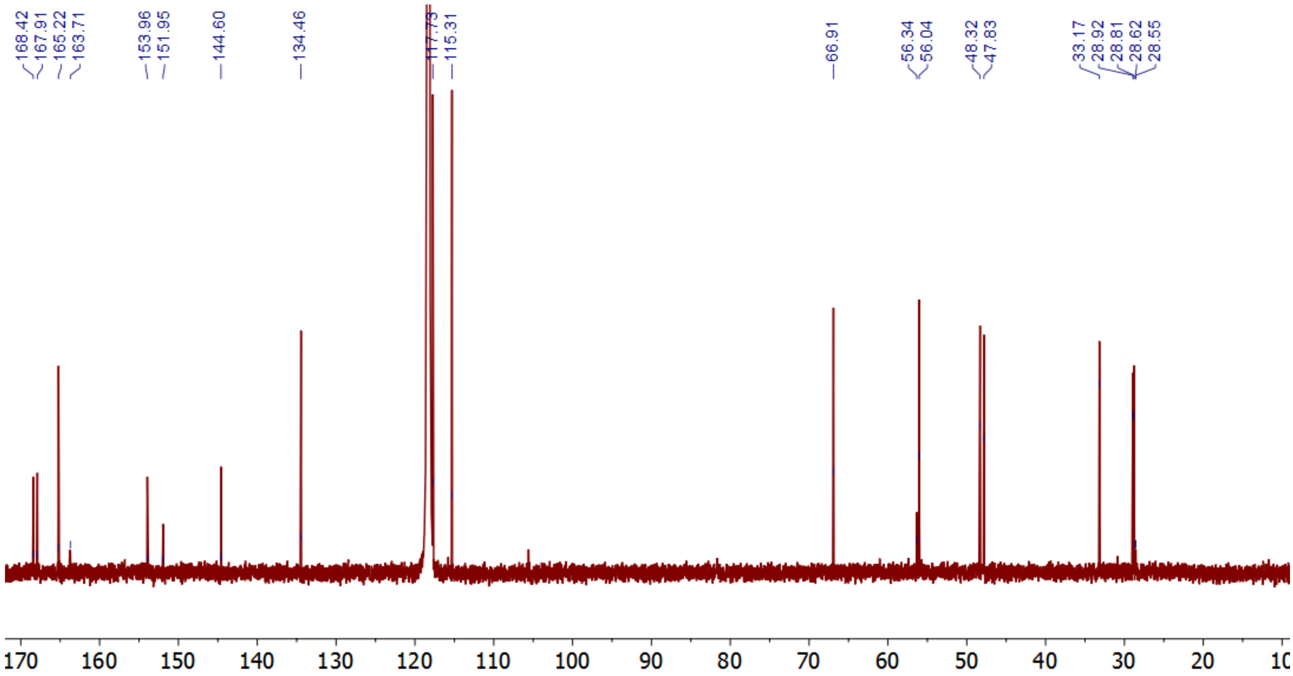

Figure S34. $^{13}C\{^{1}H\}$ NMR (151 MHz, CD_3CN, 298 K) spectrum of **14**.

17.4 1D NOESY of cyclic DASA 14b'

The X-ray crystal structure (see Section 32.9) shows compound **14b'** adopts a stacked conformation, where the methoxyphenyl ring interacts with the barbituric acid ring *via* π-π stacking. Low temperature 1D NOE NMR spectroscopy shows that this conformation is also present in solution, with NOE interactions between the phenyl protons and the methyl groups on the barbituric acid ring.

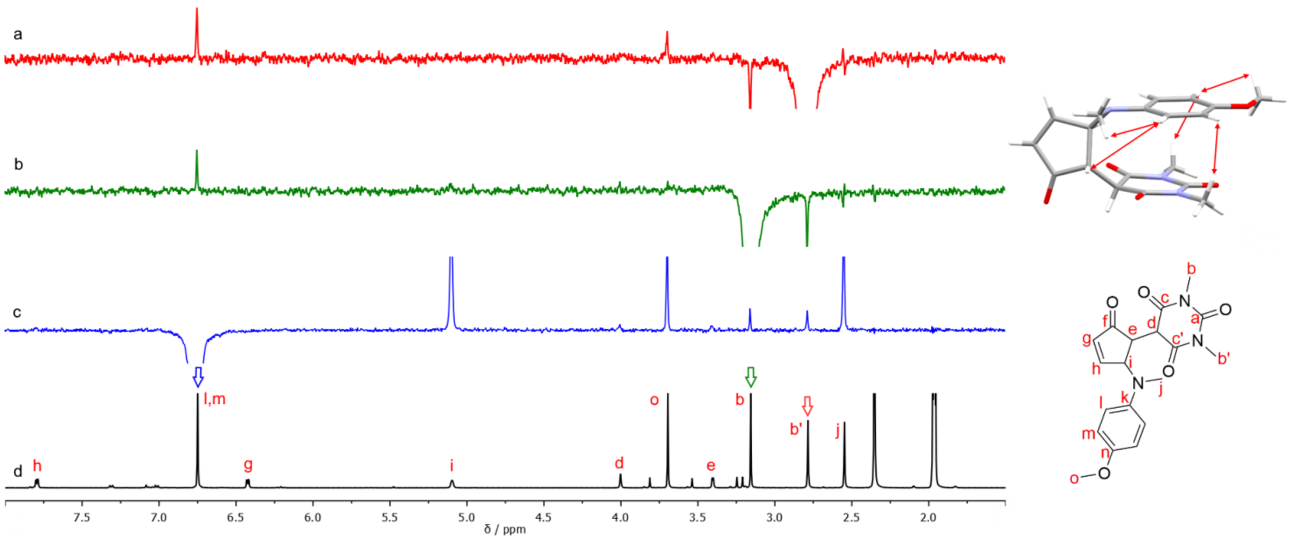

Figure S35. Stacked 1D-NOESY (500 MHz, CD_3CN, 243 K) spectra for H^b (a), $H^{b'}$ (b), H^l and H^m (c) and the ^1H reference spectrum (d).

18 Comparison of ^{1}H NMR data of 1-14 in CD_3CN and $CDCl_3$

18.1 Comparison of ^{1}H NMR spectra of 1-14 in CD_3CN

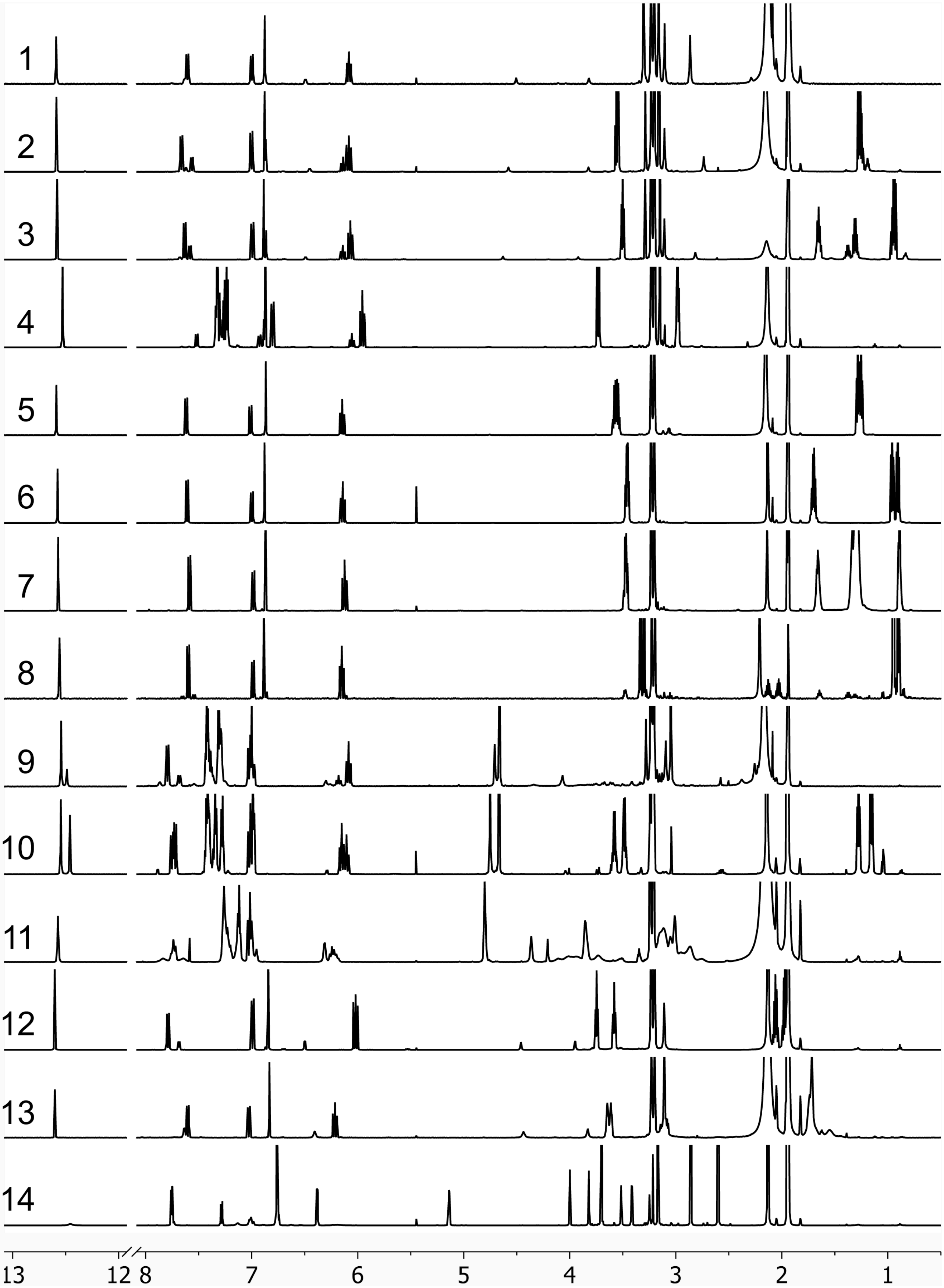

Figure S36. Stacked ^{1}H NMR (600 MHz, CD_3CN, 298 K) spectra of compounds **1-14**.

18.2 Comparison of NMR data in CD_3CN for 1-14

Table S1. ^{1}H NMR (CD_3CN, 600 MHz) comparison of DASAs **1a**–**14a**

	H^b	$H^{b'}$	OH	H^e	H^g	H^h	H^i	H^j	H^k
**1a**	3.23	3.20	12.59	6.88	7.00	6.08	7.61	3.16	
**2a**	3.23	3.20	12.59	6.88	7.00	6.08	7.66	3.16	3.55
**3a**	3.23	3.20	12.58	6.89	6.99	6.07	7.63	3.15	3.50
**4a**	3.23	3.20	12.53	6.87	6.80	5.96	7.29	3.15	3.73
**5a**	3.23	3.20	12.59	6.87	7.01	6.15	7.62	3.58	1.25
**6a**	3.23	3.20	12.58	6.88	7.00	6.14	7.61	3.47	1.70
**7a**	3.23	3.20	12.57	6.87	6.99	6.12	7.59	3.47	1.66
**8a**	3.23	3.20	12.56	6.89	6.99	6.15	7.60	3.33	2.13
**9a**	3.24	3.21	12.54	7.00	7.02	6.09	7.79	3.05	4.66
**10a**	3.24	3.21	12.55	7.01	7.01	6.15	7.72	3.48	4.67
**11a**	3.24	3.21	12.57	7.12	7.12		7.74	3.85	4.80
**12a**	3.23	3.20	12.60	6.84	6.99	6.02	7.79	3.58	2.06
**13a**	3.23	3.20	12.60	6.83	7.03	6.22	7.76	3.65	1.72
**14a**	3.25	3.22	12.45	7.01	7.13	6.19	7.74	3.52	

Table S2. ^{13}C NMR (CD_3CN, 600 MHz) comparison of DASAs **1a**–**14a**

	C^a	C^b	$C^{b'}$	C^c	$C^{c'}$	C^d	C^e	C^f	C^g	C^h	C^i
**1a**		28.3	28.2				133.0	146.0	153.8	105.2	162.9
**2a**	152.8	28.4	28.4	165.9	163.8	96.4	134.6	146.5	153.8	105.2	161.6
**3a**	152.8	28.4	28.4	165.9	163.8	96.5	134.8	146.6	153.6	104.9	161.9
**4a**	152.8	28.5	28.5	166.0	163.8	97.0	135.8	146.7	153.2	104.4	161.6
**5a**	152.8	28.4	28.4	165.9	163.9	96.2	134.2	146.4	154.2	105.1	161.2
**6a**	152.8	28.4	28.4	165.9	163.8	96.4	134.6	146.5	154.0	105.1	162.0
**7a**	152.8	28.4	28.4	165.9	163.8	96.4	134.5	146.5	153.9	105.1	161.8
**8a**	152.7	28.4	28.4	165.9	163.8	96.6	135.0	146.6	153.9	105.2	162.5
**9a**	152.8	28.5	28.5	166.1	163.8	97.7	137.0	147.0	153.2	104.5	161.4
**10a**	152.7	28.5	28.5	166.0	163.8	97.5	136.6	146.8	153.6	104.4	160.9
**11a**		28.0	28.0				136.3		153.3	104.2	160.2
**12a**	152.9	28.4	28.4	165.9	163.9	96.1	133.8	146.5	153.3	106.5	158.6
**13a**		27.6	27.6				132.8		154.1	104.3	160.6
**14a**											

18.3 Comparison of ^{1}H NMR spectra of 1-14 in $CDCl_3$

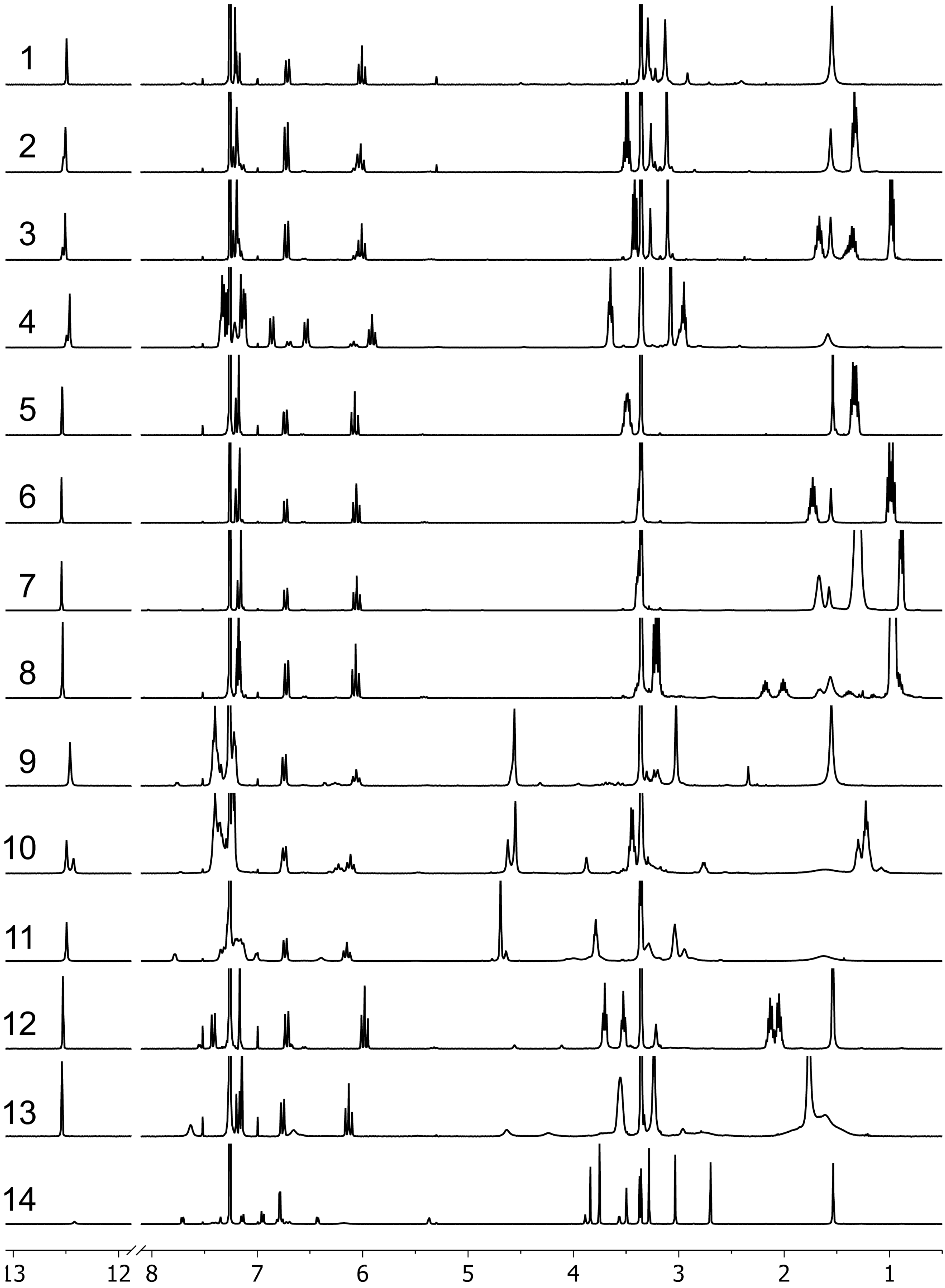

Figure S37. Stacked ^{1}H NMR (400 MHz, $CDCl_3$, 298 K) spectra of compounds **1-14**.

19 Relative abundance of linear and cyclic isomers in $CDCl_3$ and CD_3CN for 1-14, measured by ^1H NMR

Table S3. Relative percentages of conformers **a**, **a'** and isomers: **b**, **b'** for DASAs **1-14** in $CDCl_3$ and CD_3CN, measured by ^1H NMR.

	R	R'	CDCl₃				CD₃CN			
			**a**	**a'**	**b**	**b'**	**a**	**a'**	**b**	**b'**
**1a**	Me	Me	86		8	6	82		18	
**2a**	Me	Et	66	29	4	1	61	28	11	
**3a**	Me	Bu	71	26	3[a]		66	25	9	
**4a**	Me	EtPh	74	24	2		73	23	2	2
**5a**	Et	Et	99.5			0.5	97		3	
**6a**	Pr	Pr	>99.5		<0.5		>99		<1	
**7a**	Oct	Oct	>99.5		<0.5		>99		<1	
**8a**	iBu	iBu	>99.5		<0.5		>99		<1	
**9a**	Me	Bz	70	21		9	65	25		10
**10a**	Et	Bz	60	37		3	52	44		4
**11a**	Q		74[b]			26	59[b]			41
**12a**	Pyr		83		16.5	0.5	83		17	
**13a**	Pip		60		40[a]		65		35	
**14a**	Me	PhOMe	43[b]			57	17[b]			83

[a] The signals for **b** and **b'** are overlapping so could not be distinguished.
[b] The signals for **a** and **a'** are overlapping so could not be distinguished.

The general trend of linear:cyclic ratio is the same in $CDCl_3$ and CD_3CN, with the cyclic form slightly more favored in the more polar CD_3CN solvent in all cases (except **13**). Unsurprisingly, those with a large proportion of cyclic are the most sensitive to solvent changes as the two isomers (linear and cyclic) are closer in energy so small differences in relative energies will be more noticeable in terms of observed linear:cyclic ratios.

20 NMR spectroscopy assignment of cyclic isomers in $CDCl_3$: *enol* vs *keto* tautomers

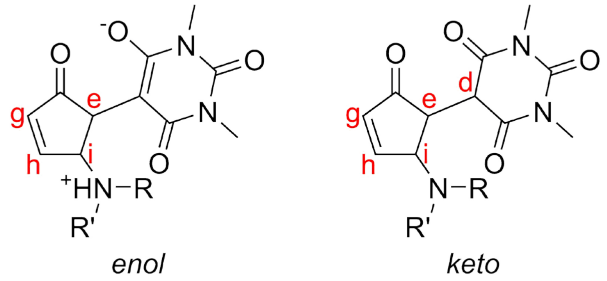

Figure S38. The cyclic isomers can undergo *enol-keto* tautomerization; the adopted conformer is strongly dependent on the substituents on the amine.

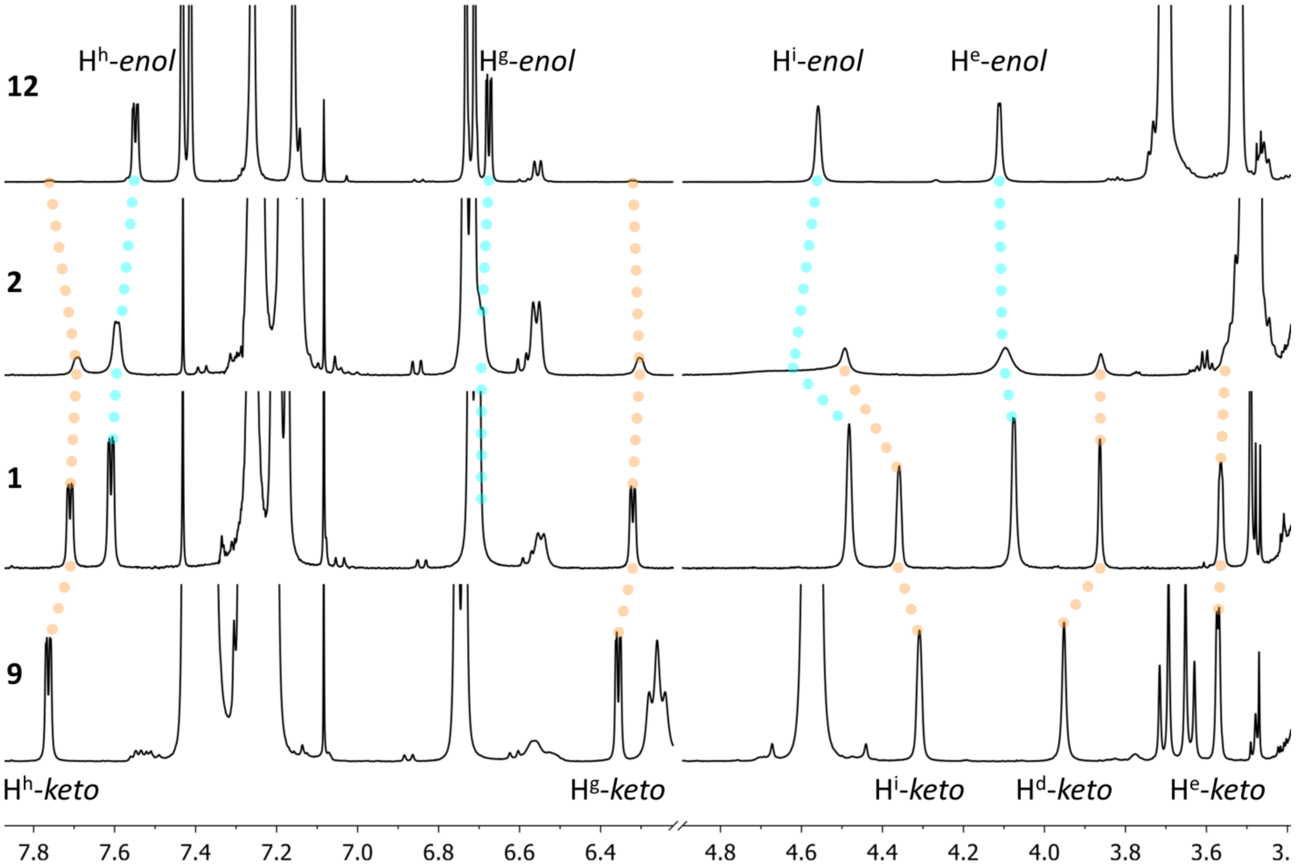

Figure S39. Stacked ^{1}H NMR (600 MHz, $CDCl_3$, 298 K) spectra of **12**, **2**, **1** and **9**. The cyclic isomer of **12** is almost exclusively present as *enol* tautomer, and **9** is almost exclusively *keto*. Compounds **2** and **1** have respectively a 1:3 and a 2:3 mixture of the *enol* and *keto*.

20.1 2D NMR spectra of 12 in $CDCl_3$

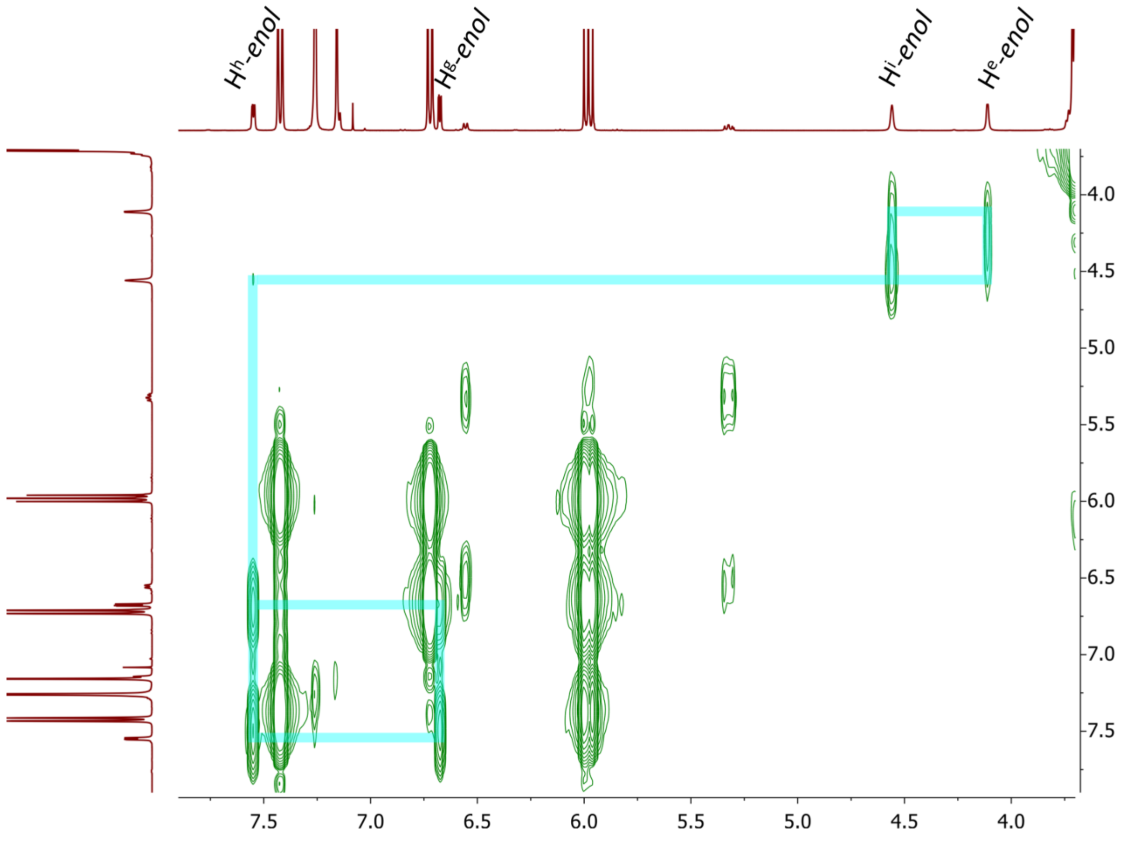

Figure S40. . ^{1}H-^{1}H COSY NMR (600 MHz, $CDCl_3$, 298 K) spectrum of **12** shows that the major cyclic isomer is the *enol* tautomer.

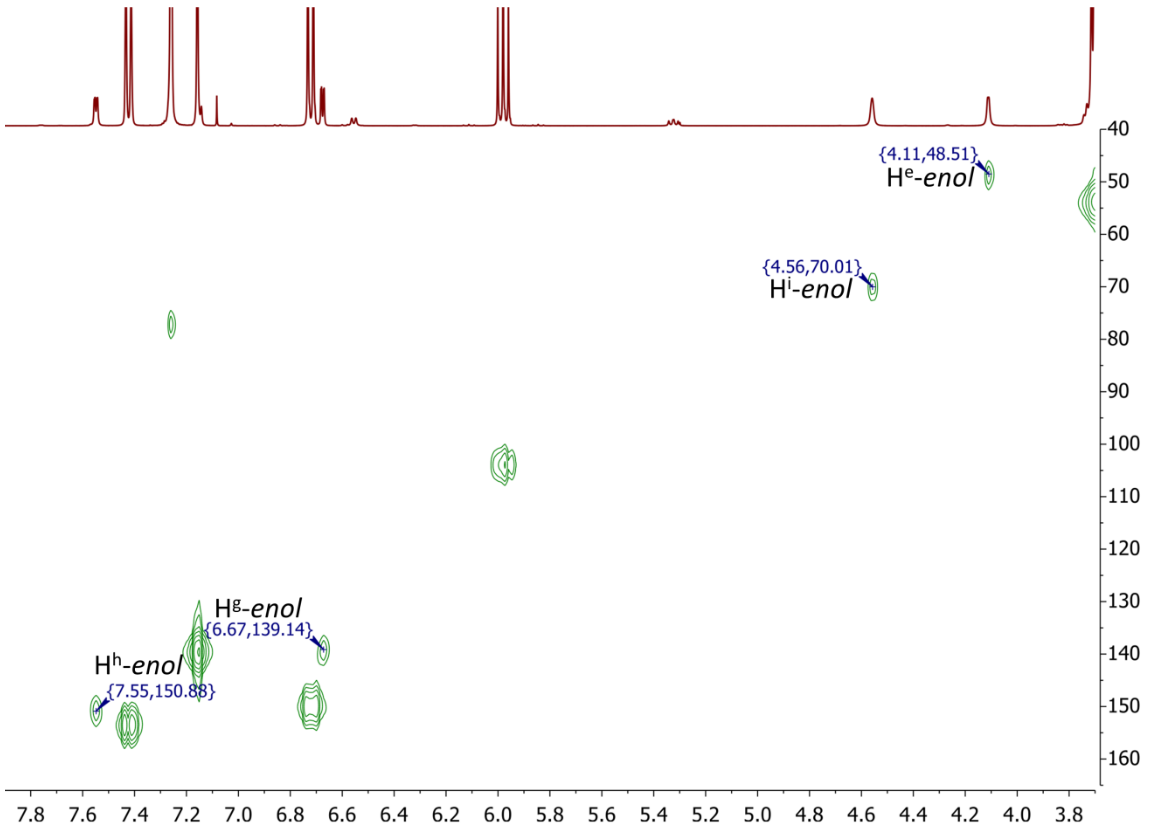

Figure S41. ^{1}H-^{13}C HSQC NMR (600 MHz, $CDCl_3$, 298 K) spectrum of **12**. The ^{13}C chemical shifts of C^h and C^g (150.9 and 139.1 respectively) are indicative for the *enol* tautomer.

20.2 2D NMR spectra of 1 in $CDCl_3$

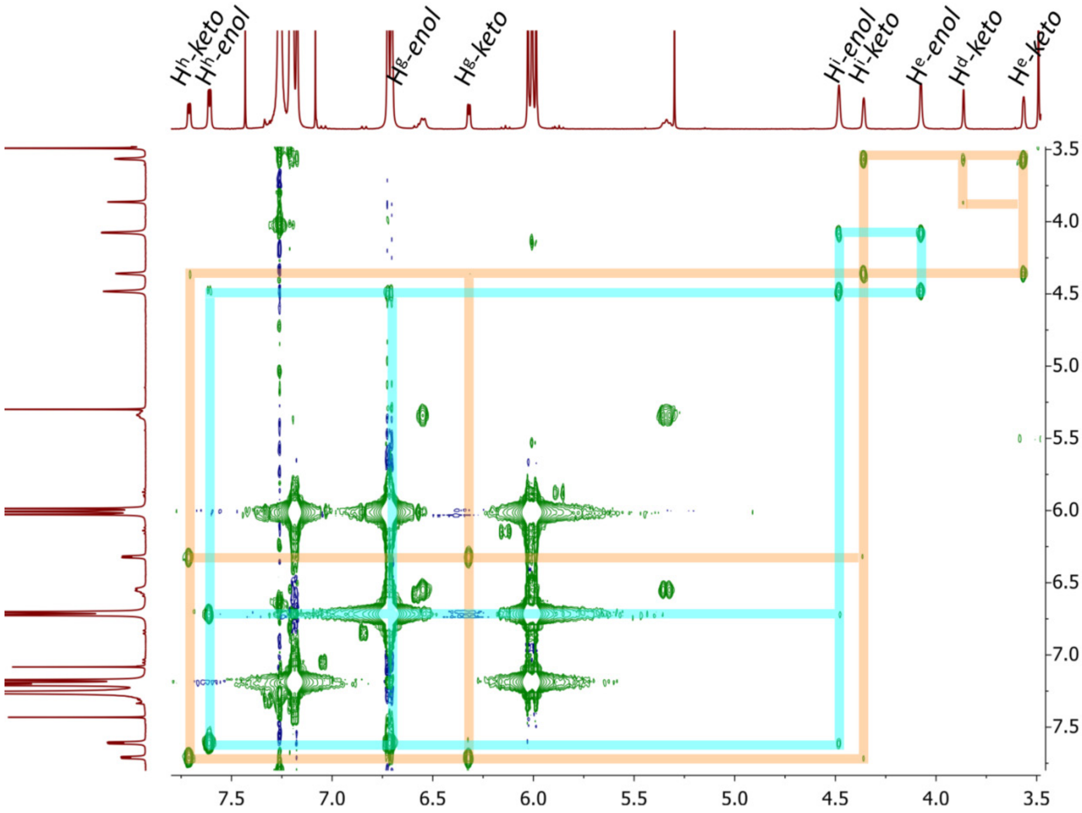

Figure S42. ^{1}H-^{1}H COSY NMR (600 MHz, $CDCl_3$, 298 K) spectrum of **1** shows that there are two independent cyclic isomers, which differ by *enol-keto* tautomerization. The *keto* (orange) has three proton signals in the aliphatic region, while the *enol* (cyan) has only two.

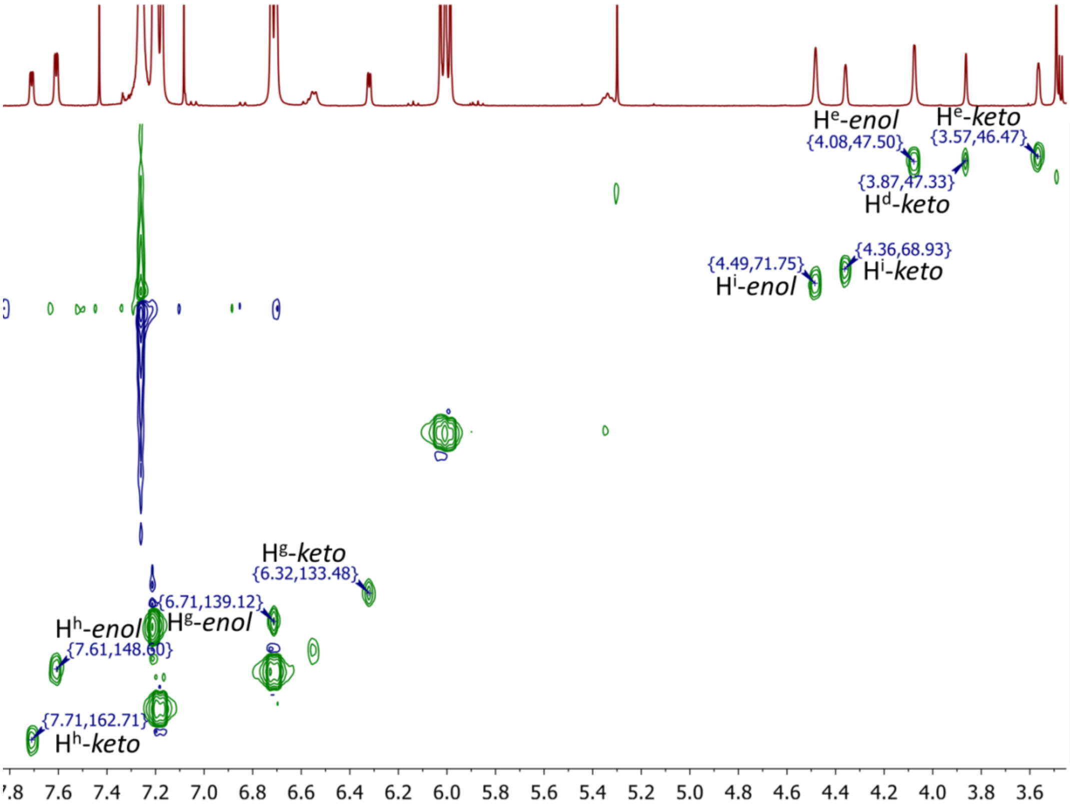

Figure S43. ^{1}H-^{13}C HSQC NMR (600 MHz, $CDCl_3$, 298 K) spectrum of **1**. Despite the small chemical shift difference between H^h (*enol vs keto*) in the ^{1}H spectrum, there is a difference of 14 ppm in the ^{13}C chemical shift of C^h, making it a reliable handle to assign the tautomers.

20.3 2D NMR spectra of 9 in $CDCl_3$

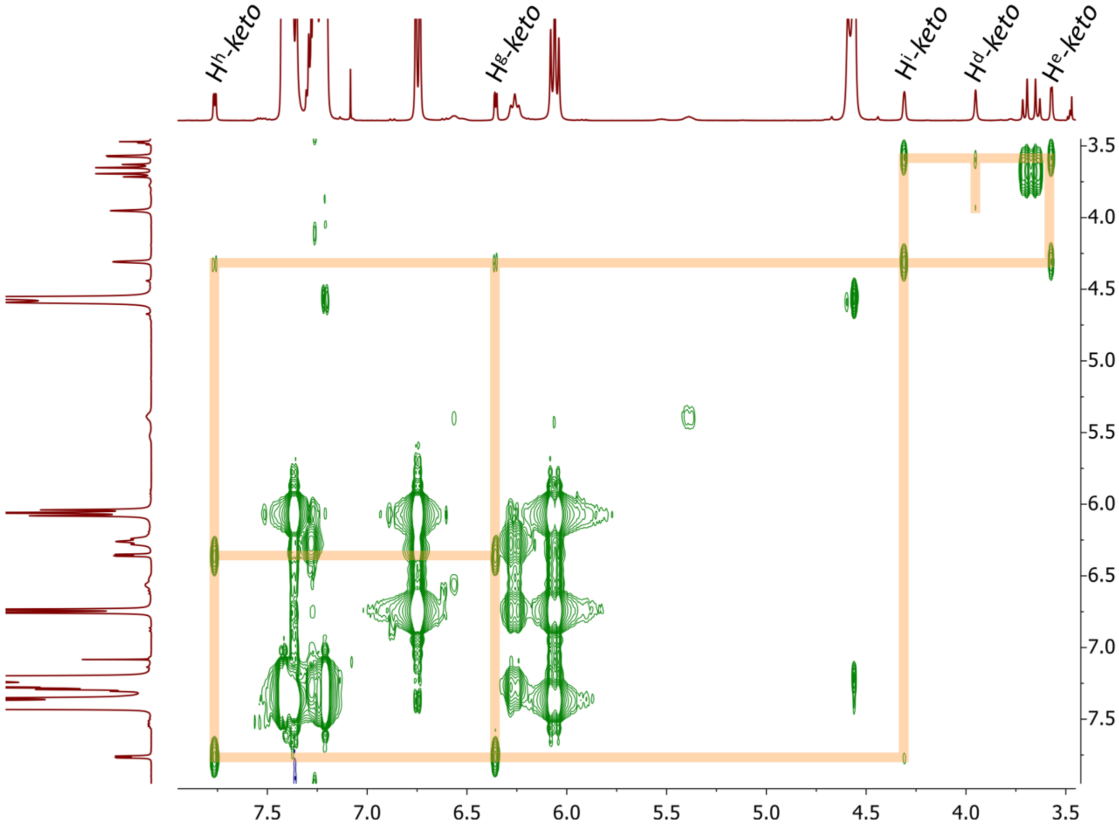

Figure S44. ^{1}H-^{1}H COSY NMR (600 MHz, $CDCl_3$, 298 K) spectrum of **9** shows three aliphatic signals are present and couple as expected in the cyclic *keto* tautomer.

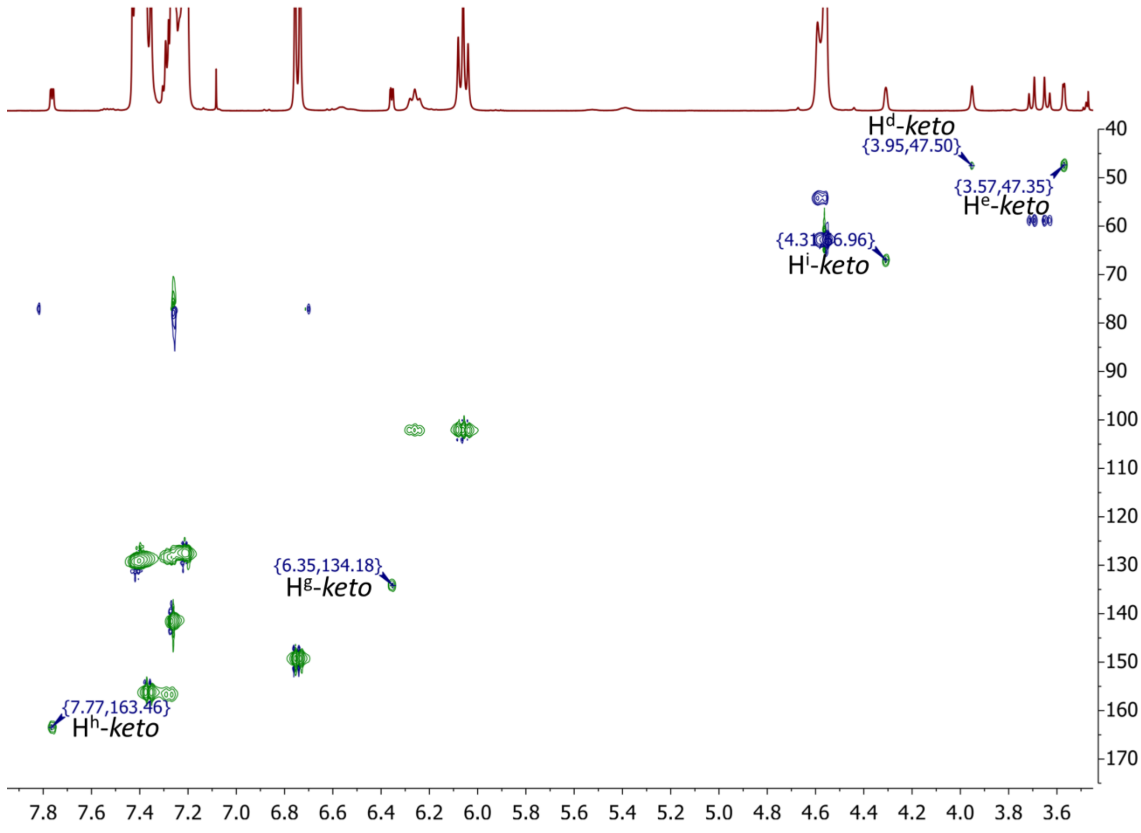

Figure S45. ^{1}H-^{13}C HSQC NMR (600 MHz, $CDCl_3$, 298 K) spectrum of **9**. The ^{13}C chemical shifts of C^h and C^g (163.5 and 134.2 respectively) are indicative for the *keto* tautomer.

20.4 2D NMR spectra of 14 in $CDCl_3$

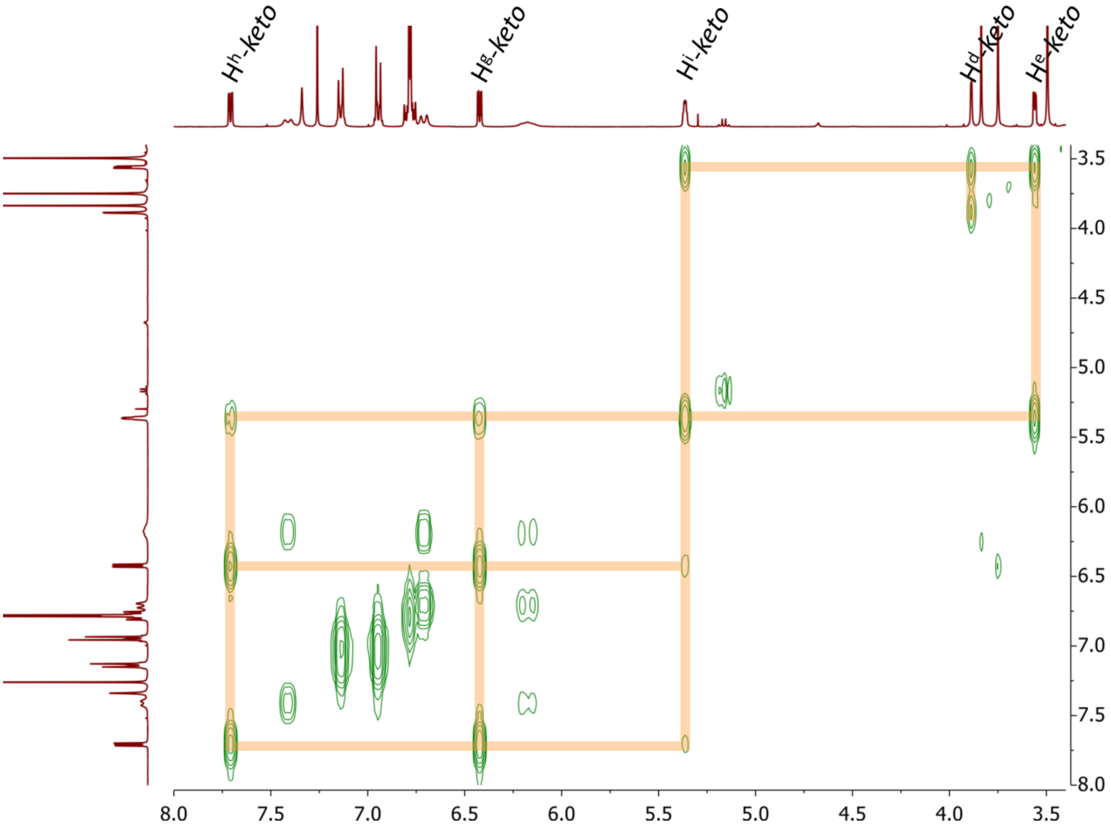

Figure S46. ^{1}H-^{1}H COSY NMR (400 MHz, $CDCl_3$, 298 K) spectrum of **14** shows three aliphatic signals are present and couple as expected in the cyclic *keto* tautomer.

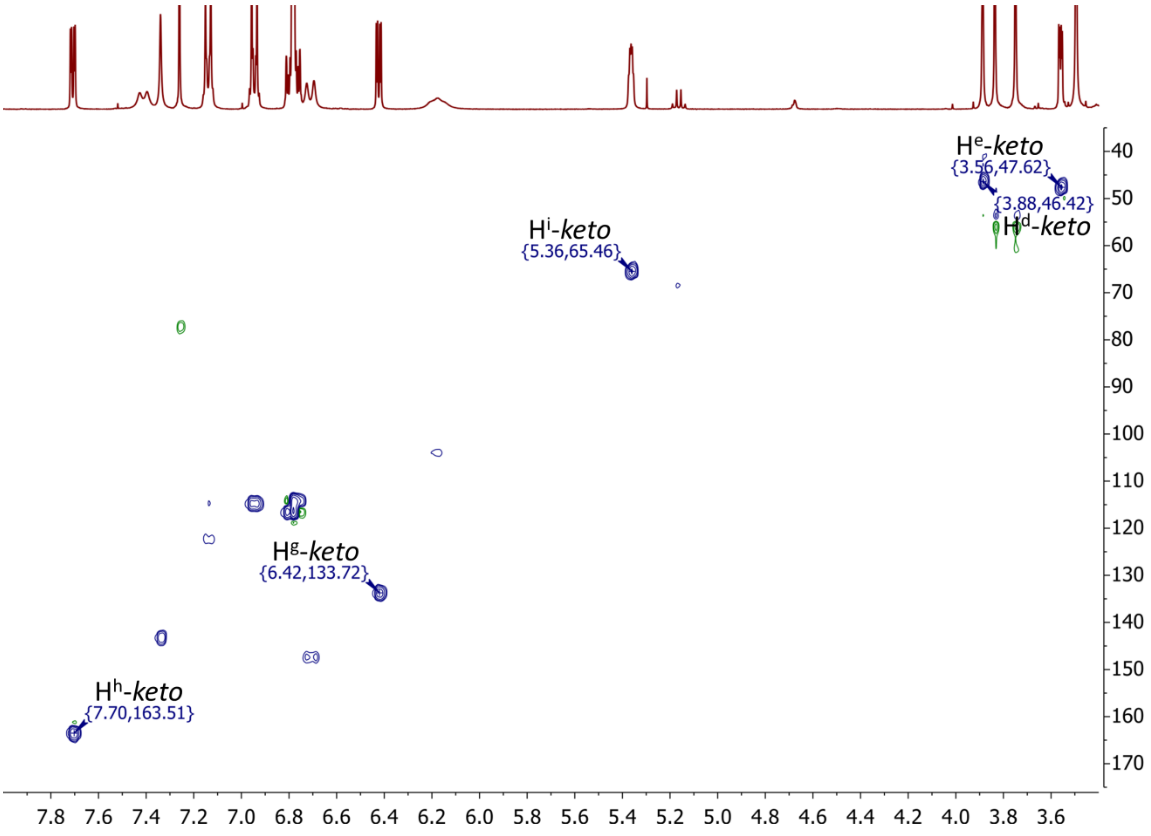

Figure S47. ^{1}H-^{13}C HSQC NMR (400 MHz, $CDCl_3$, 298 K) spectrum of **14**. The ^{13}C chemical shifts of C^h and C^g (163.5 and 133.7 respectively) are indicative for the *enol* tautomer.

20.5 Concentration dependency of *enol*/*keto* ratio

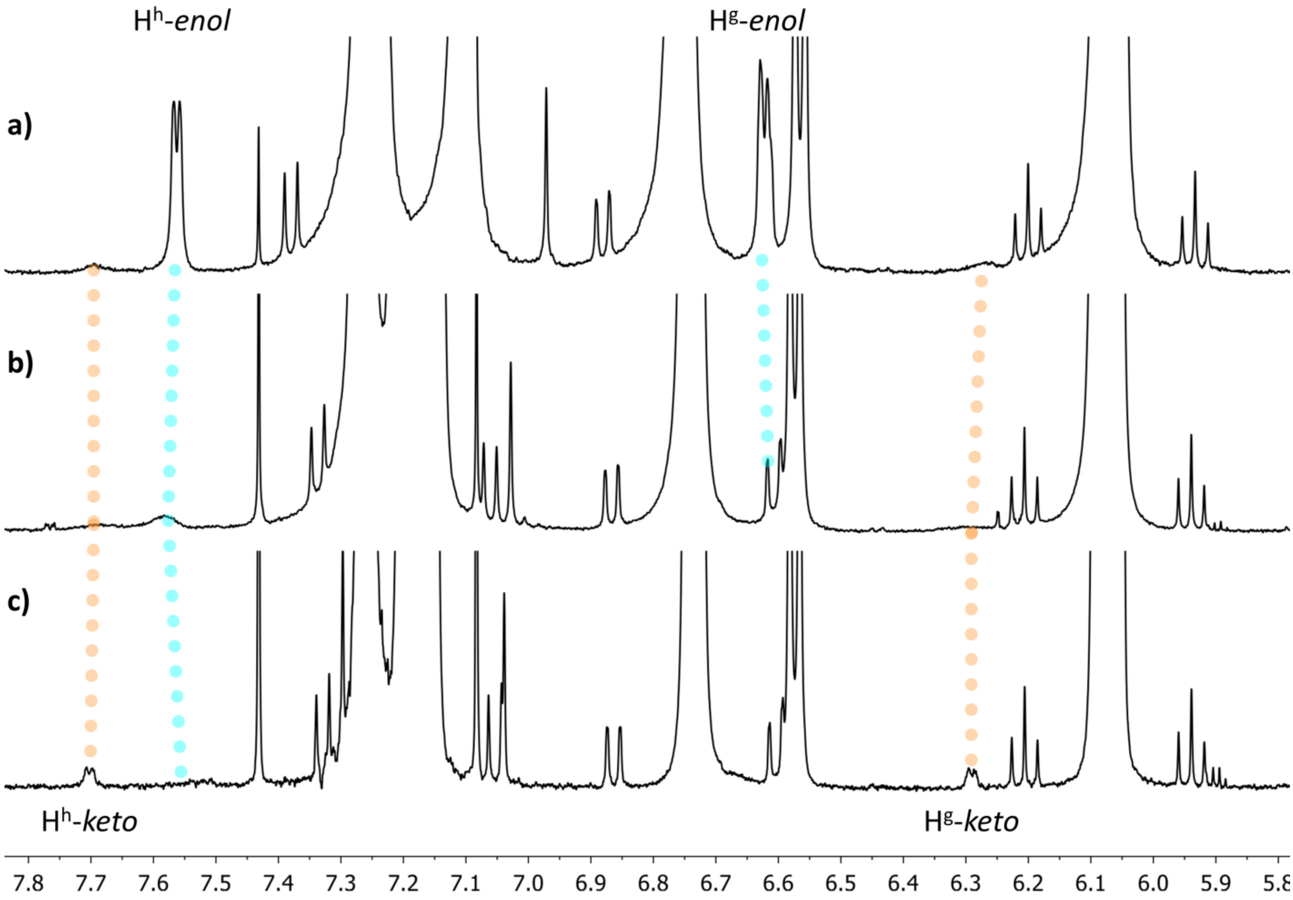

Figure S48. Stacked ^{1}H NMR (600 MHz, $CDCl_3$, 298 K) spectra of **5** at various concentrations, shown at the same intensities for the linear isomer for comparison. a) In a near saturated solution there is a large fraction of *enol* present, most likely due to intermolecular hydrogen bonding, as observed in the solid state (see 32.7 for an example). Small broad signals can be observed for the *keto* tautomer. b) After 5 × dilution most of the *enol* tautomer has disappeared, but no increase in *keto* is observed, indicating the equilibrium is pushed to favor the linear isomer. c) In dilute samples, as used throughout the characterization (~15 mM), the *keto*-isomer is favored.

21 Influence of water on the linear:cyclic equilibrium of DASA 12 in $CDCl_3$

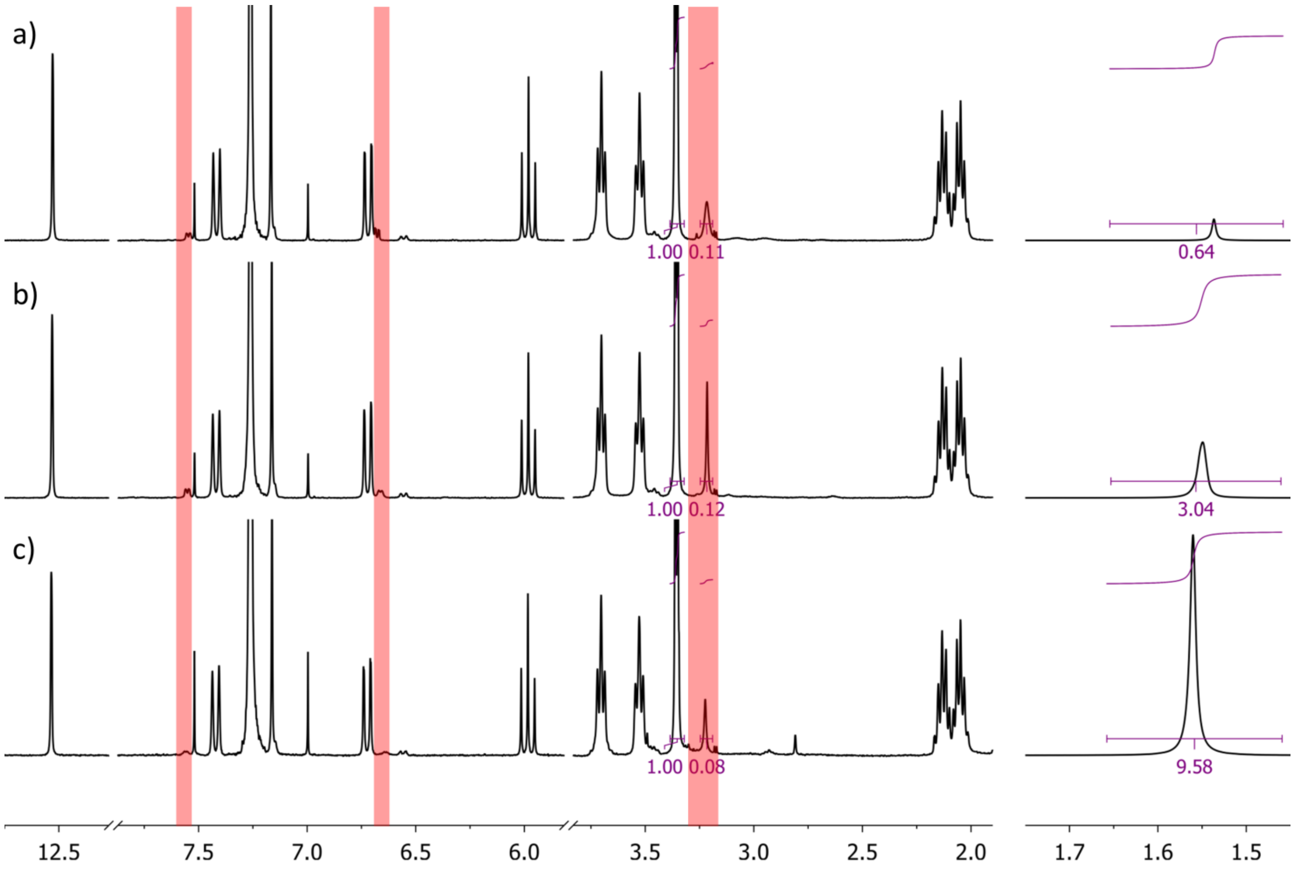

Figure S49. Stacked ^{1}H NMR (400 MHz, $CDCl_3$, 298 K) spectra of **12**. a) Sample before addition of water, prepared using $CDCl_3$ dried over K_2CO_3. b) After the addition of ~1 µL water to the NMR sample. c) Sample equilibrated in the dark for 3 days. The ratio between the linear and cyclic isomer (observable signals indicated with red boxes) does not change significantly as result of the addition of water. The integrals for the Barbituric acid N-Me groups of both the linear and cyclic isomer are given for comparison.

22 Summary of absorption, fatigue resistance and apparent thermal half-life data in $CHCl_3$ and MeTHF

Table S4. Absorption maxima, apparent thermal half-lives and fatigue for DASAs **1**–**14** in chloroform and 2-MeTHF.

	$CHCl_3$					2-MeTHF						
	λ_{max} (nm)[a]	$t_{½}$ (s) 1st cycle	$t_{½}$ (s) 100th cycle	Recovery /cycle[b] (%)	% Abs at PSS [c]	λ_{max} (nm)[a]	$t_{½}$ (s) 1st cycle	$t_{½}$ (s) 50th cycle	Recovery /cycle[b] (%)	% Abs at PSS[c]	Change in $t_{1/2}$ from $CHCl_3$ to MeTHF (s)	Change in % Abs at PSS from $CHCl_3$ to MeTHF (%)
**1a**	565	32	32	99.7	6	561	48	47	99.84	2	16	-4
**2a**	565	20	20	99.1	25	562	27	27	99.79	4	7	-21
**3a**	566	13	14	99.4	42	563	25	25	99.81	5	12	-37
**4a**	568	10	11	99.7	14	565	31	30	99.88	3	21	-11
**5a**	565	11	12	99.1	74	563	18	18	99.48	17	7	-57
**6a**	567	6	6	99.4	82	565	22	22	99.52	15	16	-67
**7a**	568	8	8	99.4	86	566	19	19	99.58	22	11	-64
**8a**	569	11	8	99.5	85	566	57	57	99.61	9	46	-76
**9a**	569	29	29	99.9	4	565	104	103	99.87	0	75	-4
**10a**	570	18	17	99.7	18	567	81	80	99.90	1	63	-17
**11a**	570	73	75	99.8	1	566	161	163	99.87	0	88	-1
**12a**	570	92	91	99.5	35[d]	567	37	36	99.32	18	-55	-17
**13a**	566	91	90	99.5	9[e]	563	78	78	99.86	1	-13	-8
**14a**	588	265	270	99.9	1	583	1558	1556	99.96[f]	0	1293	-1

[a] All data measured with absorbance of 0.95 ± 0.05 in $CHCl_3$. In 2-MeTHF the absorbance was 1.00 ± 0.05 for compounds **1-13**, and 0.81 for compound **14**. In all cases the λ_{max} in 2-MeTHF is blue shifted by 2–5 nm compared to the values in $CHCl_3$.

[b] Calculated over all switching cycles $\left(\frac{A_{end}}{A_{Start}}\right)^{\frac{1}{n}}$, where n is the number of cycles.

[c] Remaining absorbance at λ_{max} after 45 s of irradiation.

[d] Required 5 min of irradiation to reach a PSS.

[e] Required 90 s of irradiation to reach a PSS.

[f] The sample did not reach a thermal equilibrium at the end of the experiment; A_{end} was calculated from exponential regression.

23 Kinetic modelling of UV-visible absorption data

23.1 Description of mechanistic model

An incomplete, but useful picture of the possible reactions of the DASAs of this study is shown below, based on the reported mechanism,[3] and our observations.

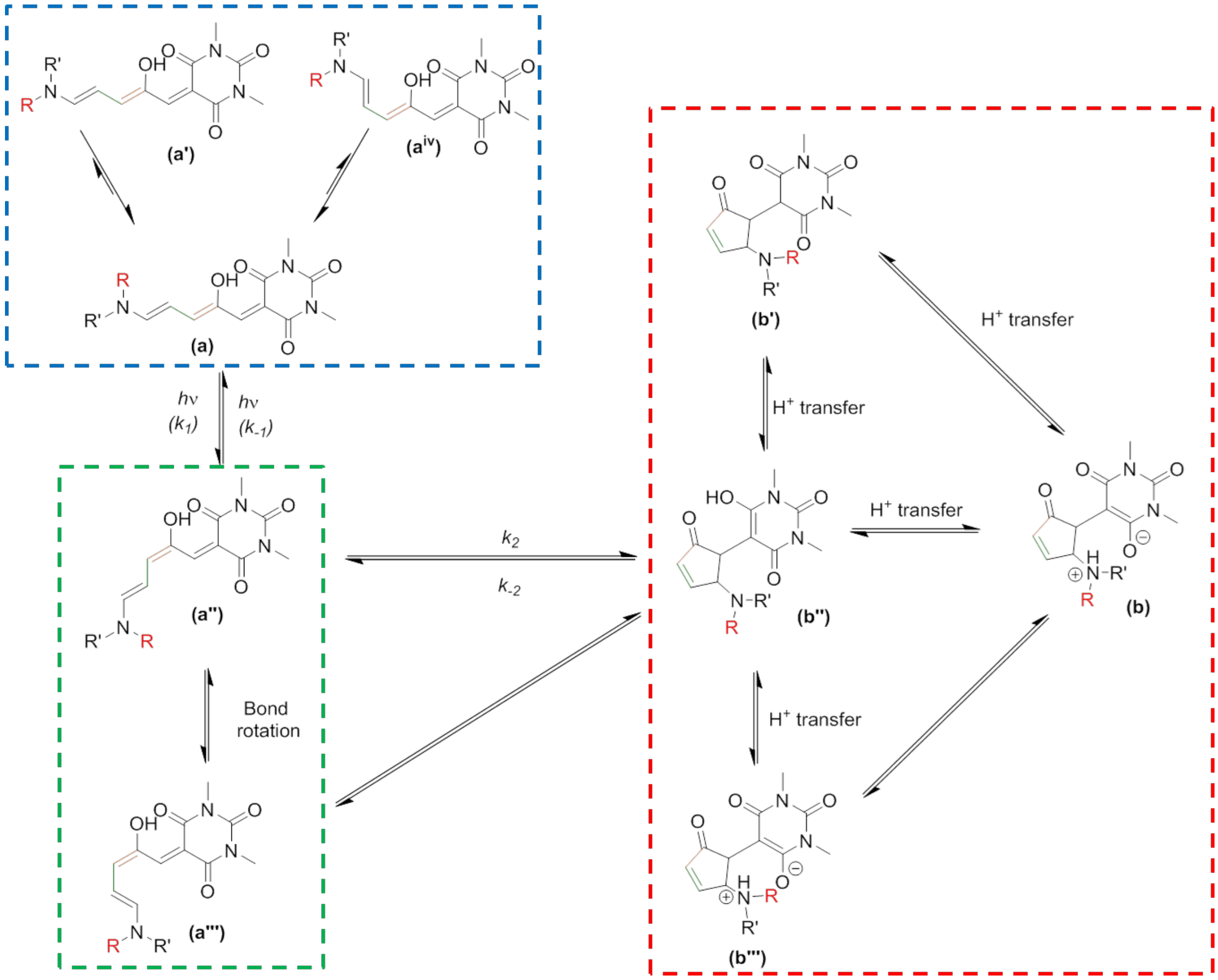

Figure S50. Proposed mechanism for isomerization.

In cases where non-symmetrical amines are used the major linear isomer (**a**) has the smallest substituent (R in the figure) *cis* to the triene, which is in equilibrium with a minor isomer (**a'**) which interchange by a N-C bond rotation. All DASAs also appear to be in equilibrium with other conformers, such as **a**^{iv}, and it remains unclear the role, if any, of these isomers in the isomerisation process. In the kinetic model will consider all these linear isomers together, as component **A**.

The initial photoisomer has been assigned[3] as structure **a''**, which must undergo a further bond rotation to generate structure **a'''**. In this study we will not identify which species is the photoisomer but simply model this species as the colored species intermediate **I**.

The ring closing reaction may involve a concerted proton transfer to give **b''**, and is assumed to be the slowest step of the isomerisation. This step is followed by rapid proton transfer to form **b.** Finally cyclic form **b** is potentially in equilibrium with tautomer (**b')**, and in cases with non-symmetrical amines, further conformers (e.g. **b'''**) are observable in some instances. In this kinetic model we will consider all these cyclic isomers together as colorless component **B**.

23.2 Description of a simplified kinetic model

The simplified kinetic model is shown below.

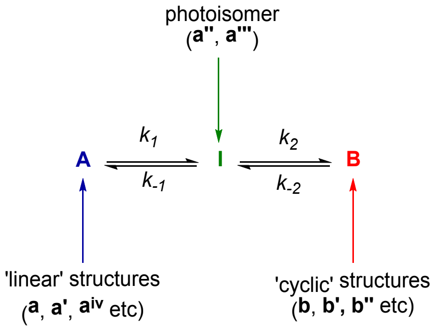

Figure S51. Simplified mechanism for isomerization used to model UV-visible switching data.

The initial step is reversible with rate constants k_1 and k_{-1}. Both steps k_1 and k_{-1} will be light dependant as both the starting linear isomer (**a**) and the photoisomer (**a''/a'''**) have absorption overlapping within the irradiation region (a band from the photoisomer visible at longer wavelengths ~630 nm upon irradiation). However, both these steps also occur in the dark as the systems are observed to equilibrate in the dark, so k_1 and k_{-1} were fitted with different values in the light (k_1^l and k_{-1}^l) and dark (k_1^d and k_{-1}^d).

This ring closing/opening process (k_2 and k_{-2}) should be light-independent as the ring closed product (**b**), and presumably tautomers (**b'**) and (**b''**) and conformer (**b'''**), have no absorption in the visible part of the spectrum where the irradiation occurs, and therefore it is reasonable these processes are not influenced by light. After completing fitting with the ring closing (k_2) and ring opening (k_{-2}) in the light and dark unconstrained, no significant differences were found between the values either in the presence or in the absence of irradiation. Therefore, to simplify the process, the fitting was carried out with the values of k_2 and k_{-2} constrained to be the same in the light and in the dark.

The ratio of linear:cyclic isomers ([A]:[B]) in the dark at equilibrium was determined using ^{1}H NMR spectroscopy, including all linear and cyclic conformers/isomers that were present (*i.e.* [A] = **a** + **a'** + **a**^{iv} and [B] = **b** + **b''** + **b'''**). In our kinetic model all these linear, and all the cyclic isomers are considered as two species only. This ratio of the [A]/[B] measured using NMR spectroscopy can be correlated to the relative products of the rate constants in the dark:

$$\frac{[A]}{[B]} = \frac{k_{-1}^d k_{-2}}{k_1^d k_2}$$

So if the system is at equilibrium at the start of the measurement in the dark:

$$\frac{k_1^d}{k_{-1}^d} = \frac{[B]_{t=0}}{[A]_{t=0}} \frac{k_{-2}}{k_2}$$

As k_1^d is very small with respect to k_{-1}^d, modelling these variables independently gave poor reproducibility, likely due to the limited amount of data in the region where these rate constants are most important (immediately after the irradiation is switched off). Therefore, the above ratio $\frac{k_1^d}{k_{-1}^d}$ was determined; these data were effectively modelled in all cases and we are confident with their relative rates.

23.3 Modelling absorption

The measured absorption being modelled is:

$$A_{\lambda_{max}} = \varepsilon_A[A] + \varepsilon_I[I]$$

Where:

$A_{\lambda_{max}}$ = measured absorption at λ_{max} for each DASA

ε_A = molar extinction coefficient for A

ε_I = molar extinction coefficient for I

Molar absorptivities were determined for DASAs **2a**, **5a** and, **6a** in chloroform as these compounds exist as 98 %, >99.5 %, >99.5 % linear (**a**+**a'**) isomers in solution respectively (determined using ^{1}H NMR spectroscopy). These compounds all have similar extinction co-efficients (corrected for % cyclic) at λ_{max} of 142 ± 2 × 10^3 L·mol^{-1}·cm^{-1}. These values do differ from previous values reported in toluene (i.e. **5a**: 100 × 10^3 L·mol^{-1}·cm^{-1},[2] 176 ± 2 × 10^3 L·mol^{-1}·cm^{-1}),[4] but is in line with the non-hydroxyl derivative of **5a** (in toluene), reported as 143 × 10^3 L·mol^{-1}·cm^{-1}.[5]

For the sake of the kinetic model, we have assumed the extinction coefficients of the remaining DASAs also have the same value, so that ε_A = 142 × 10^3 L·mol^{-1}·cm^{-1} for all DASAs studied. Changing this value has insignificant effects on the data reported.

Although there is some variation in the absorption profiles of the compounds studied, overlap with the emission of the LED is very similar, except for DASA **14** (see Figure S56). All measurements were performed with an initial absorption at the λ_{max} for each sample of 0.95 ± 0.05 to ensure, as close as possible, the different DASAs are compared with the same overall absorption of light.

Upon irradiation the formation of the photoisomer is observed by an absorption band at longer wavelengths,[3] and this isomer also has considerable absorption at the λ_{max} of the major linear isomer. The extinction coefficient of the photoisomer (ε_I) cannot be measured directly, and was initially unconstrained in the fitting process; fitted values range from 20–120 × 10^3 L·mol^{-1}·cm^{-1}. In order to have reasonable data to compare, we assume the ratio of the absorption of the two isomers is the same for all compounds, *i.e.* $\varepsilon_I / \varepsilon_A$ does not vary significantly through the series. Therefore ε_I was fixed as 95 × 10^3 L·mol^{-1}·cm^{-1} for all DASAs studied. Changing the value of ε_I does not change the fitted values for k_{-2}, but as $\varepsilon_I / \varepsilon_A$ increases the fitted values of k_2 systematically decrease and k_1^d/k_{-1}^d systematically increase. The observed trends in relative rate constants between compounds remains the same regardless of the value of $\varepsilon_I / \varepsilon_A$, giving confidence in the conclusions reached.

23.4 Kinetic data fitting

The absorption data was fitted over one complete switching cycle for each compound. That is, the data from an equilibrated solution in the dark to an equilibrated sample at the photostationary state, and the thermal recovery in the dark. The data was modelled as light (k_1^l and k_{-1}^l) and dark (k_1^d and k_{-1}^d) periods using numerical integration and the global fit optimised using solver in Microsoft Excel.

Variables:

k_1^l

k_{-1}^l

$\frac{k_1^d}{k_{-1}^d}$

k_2

k_{-2}

Subject to:

$\varepsilon_A = 142 \times 10^3$ L·mol^{-1}·cm^{-1}

$\varepsilon_I = 95 \times 10^3$ L·mol^{-1}·cm^{-1}

$$\frac{k_1^d}{k_{-1}^d} = \frac{[B]_{t=0}}{[A]_{t=0}} \frac{k_{-2}}{k_2}$$

where $[B]_{t=0}$ and $[A]_{t=0}$ are the concentrations of A and B in the dark at equilibrium.

23.5 Determination of equilibrium concentrations

At equilibrium (dark):

$$\frac{[A]}{[I]} = \frac{k_{-1}^d}{k_1^d}$$

$$\frac{[A]}{[B]} = \frac{k_{-1}^d k_{-2}}{k_1^d k_2}$$

(which can be measured directly using ^{1}H NMR spectroscopy)

$$[I] = \frac{k_1^d [A]}{k_{-1}^d} = \frac{k_{-2}[B]}{k_2}$$

Similarly at the PSS:

$$\frac{[A]}{[I]} = \frac{k_{-1}^l}{k_1^l}$$

$$\frac{[A]}{[B]} = \frac{k_{-1}^l k_{-2}}{k_1^l k_2}$$

$$[I] = \frac{k_1^l [A]}{k_{-1}^l} = \frac{k_{-2}[B]}{k_2}$$

The concentration of [A] + [I] + [B] is assumed to be constant during each measurement. That is, that there is no decomposition during the measured cycle, which is a reasonable assumption as the

highest decomposition measured was just 0.9% over the cycle used to model the kinetics. Therefore the relative concentrations at equilibrium (dark or PSS) from this kinetic model can be expressed as:

$$\frac{[A]}{[total]} = \frac{\frac{k_{-1}}{k_1}}{1 + \frac{k_{-1}}{k_1} + \frac{k_{-1}}{k_1}\frac{k_1 k_2}{k_{-1}k_{-2}}} = \frac{\frac{k_{-1}}{k_1}}{1 + \frac{k_{-1}}{k_1} + \frac{k_2}{k_{-2}}}$$

$$\frac{[I]}{[total]} = \frac{1}{1 + \frac{k_{-1}}{k_1} + \frac{k_{-1}}{k_1}\frac{k_1 k_2}{k_{-1}k_{-2}}} = \frac{1}{1 + \frac{k_{-1}}{k_1} + \frac{k_2}{k_{-2}}}$$

$$\frac{[B]}{[total]} = \frac{\frac{k_{-1}}{k_1}\frac{k_1 k_2}{k_{-1}k_{-2}}}{1 + \frac{k_{-1}}{k_1} + \frac{k_{-1}}{k_1}\frac{k_1 k_2}{k_{-1}k_{-2}}} = \frac{\frac{k_2}{k_{-2}}}{1 + \frac{k_{-1}}{k_1} + \frac{k_2}{k_{-2}}}$$

Where the appropriate k_1 and k_{-1} values are used for the PSS (k_1^l and k_{-1}^l) or the dark (k_1^d and k_{-1}^d).

24 Summary of kinetic modelling data

24.1 Rate constants from kinetic modelling

Table S5. Modelled rate constants for photoswitching of DASAs **1**–**14** in chloroform, sorted by apparent thermal half-life ($t_{½}$).[a]

			Dark					Light				
#	R	R'	$t_{½}$ (s^{-1})[b]	k_2 (× 10^{-3} s^{-1})	k_{-2} (× 10^{-3} s^{-1})	$\frac{k_2}{k_{-2}}$	$\frac{k_1^d}{k_{-1}^d}$ (× 10^{-3})	k_1^l (s^{-1})	k_{-1}^l (s^{-1})	$\frac{k_1^l}{k_{-1}^l}$	$k_1\ k_2$ (× 10^{-3} s^{-2})	$\frac{k_1^l k_2}{k_{-1}^l k_{-2}}$ = $[B]^l/[A]^l$
**6**	Pr	Pr	6	21	91	0.2	4.4	0.60	1.1	0.5	12	0.1
**7**	Oct	Oct	8	12	61	0.2	5.2	0.4	0.9	0.4	5	0.1
**4**	Me	EtPh	10	240	65	3.6	5.7	1.0	<0.01	>100	240	>370
**5**	Et	Et	11	35	58	0.6	8.4	0.78	1.5	0.5	27	0.3
**8**	iBu	iBu	11	10	49	0.2	4.9	0.62	1.4	0.5	6	0.1
**3**	Me	Bu	13	100	50	2.1	15	0.48	0.46	1.0	49	2.2
**10**	Et	Bz	18	180	38	4.6	6.7	0.57	0.22	2.7	100	12
**2**	Me	Et	20	130	33	3.8	14	0.54	0.36	1.5	70	5.9
**9**	Me	Bz	29	480	23	21	4.6	2.0	<0.01	>200	950	>4200
**1**	Me	Me	32	250	19	13	13	1.0	<0.01	>100	240	>1300
**11**	Q		73	530	7	75	4.7	1.3	0.01	125	680	9400
**13**	Pip		91	150	5	31	22	0.38	0.63	0.6	55	19
**12**	Pyr		92	26	7	3.7	55	0.49	1.1	0.5	13	1.7
**14**	Me	PhOMe	265	4400	1	3500	0.38	1.5	<0.01	>150	6700	>500000

[a] Rate constants refer to kinetic model in Section S23. Values for rate constants are rounded to two significant figures. Red, orange, green colors are arbitrary within each column.
[b] Apparent half-life calculated from data in Section S28

24.2 Predicted and measured change in absorption and linear:cyclic dark equilibrium ratios.

Table S6. Comparison of measured data with predicted from the kinetic model.

			%Δ Abs		% linear isomer in the dark		Predicted distribution at PSS[c]		
DASA	R	R'	Measured % Abs at PSS[a]	Predicted % Abs at PSS[b]	Predicted % **A** in the dark[c]	Measured % (**a+a'**) in the dark (NMR)[d]	% **A**	% **I**	% **B**
**6**	Pr	Pr	82	82	100	>99	61	32	7
**7**	Oct	Oct	86	85	100	>99	66	28	5
**4**	Me	EtPh	14	15	98	98	0	22	78
**5**	Et	Et	74	73	100	>99	54	29	17
**8**	iBu	iBu	85	84	100	>99	65	29	6
**3**	Me	Bu	42	40	97	97	24	25	51
**10**	Et	Bz	18	18	97	97	6	17	77
**2**	Me	Et	25	24	95	95	12	18	70
**9**	Me	Bz	4	3	91	91	0	4	96
**1**	Me	Me	6	5	86	86	0	7	93
**11**	Q		1	1	74	74	0	1	99
**13**	Pip		9[e]	7	60	60	5	3	92
**12**	Pyr		35[f]	41	83	83	31	15	54
**14**	Me	PhOMe	1	0	43	43	0	0	100

[a] Measured change in absorption at λ_{max}, measured as proportion of initial (dark) absorption, requiring 45 s of irradiation in all cases, except for compound **12** (5 min) and **13** (90 s) that are slower to isomerize.
[b] Calculated from relative abundance of **a** and **b** at PSS with fixed extinction coefficients of 142×10^3 and 95×10^3 $L\cdot mol^{-1}\cdot cm^{-1}$ respectively.
[c] Predicted distributions based on simplified kinetic model and rate constants in Table S5.
[d] Measured by ^1H NMR in $CDCl_3$.
[e] Required 5 min of irradiation to reach a PSS.
[f] Required 90 s of irradiation to reach a PSS.

24.3 Comparison of relative energies of isomers A, I, the highest energy transition state (TS) and B.

Table S7. Comparison of relative free energies of DASA isomers determined from our measure rate constants in Table S6.[a]

DASA	**A**	**I**[b]	**TS**[c]	**B**[d]	**b/b'**[e] (from NMR)
**1**	0	11	87	4	4
**2**	0	11	89	7	7
**3**	0	10	89	9	9
**4**	0	13	89	10	10
**5**	0	12	93	13	13
**6**	0	13	96	17	>13
**7**	0	13	97	17	>13
**8**	0	13	98	17	>13
**9**	0	13	88	6	6
**10**	0	12	89	8	8
**11**	0	13	88	3	3
**12**	0	7	89	4	4
**13**	0	10	87	1	1
**14**	0	20	89	-1	-1

[a] All free energies at 298 K in $kJ.mol^{-1}$, relative to A for each DASA. A, I and B refer to our simplified kinetic model. A = **a**+**a'**, **I** is likely **a''** and B = **b**+**b'**.
[b] Calculated from k_1^d/k_{-1}^d.
[c] Calculated from barrier corresponding to k_{-2} and the energy of cyclic isomer (from $k_1k_2/k_{-1}k_{-2}$ = [B]/[A]); also equal to energy of the intermediate I + barrier corresponding to k_2.
[d] Calculated from $k_1k_2/k_{-1}k_{-2}$ = [B]/[A].
[e] Measured by integration of non-overlapping ^1H NMR signals of **b** and/or **b'** relative to those of **a**+**a'**.

25 Correlation of rate constants with Taft parameters

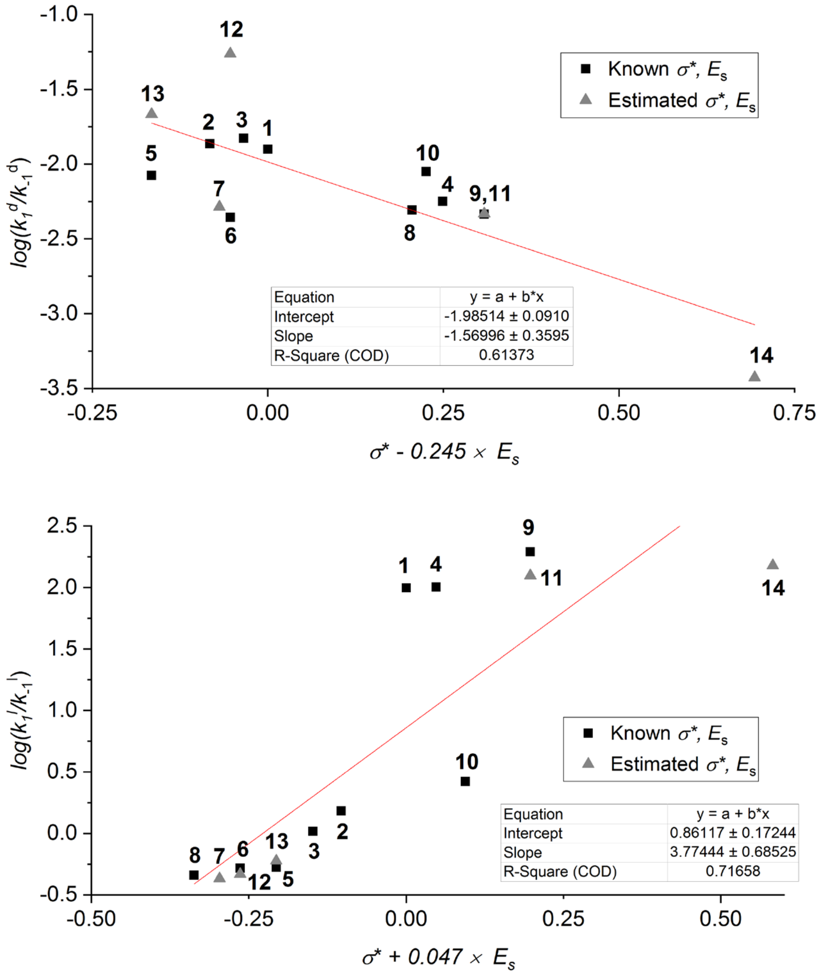

Figure S52. The correlations of the logarithm of rate constants k_1/k_{-1} with Taft parameter (σ*) and steric contribution (E_s)[6] in (top) the light or (bottom) the dark. All fittings were performed on all data points. The values of σ* + E_s are estimated for some cases where data is not available: **7** = 2 × n-Bu; **11** = Bz and Me; **12** = 2 × Pr; **13** = 2 × Et; **14** = Me and Ph.

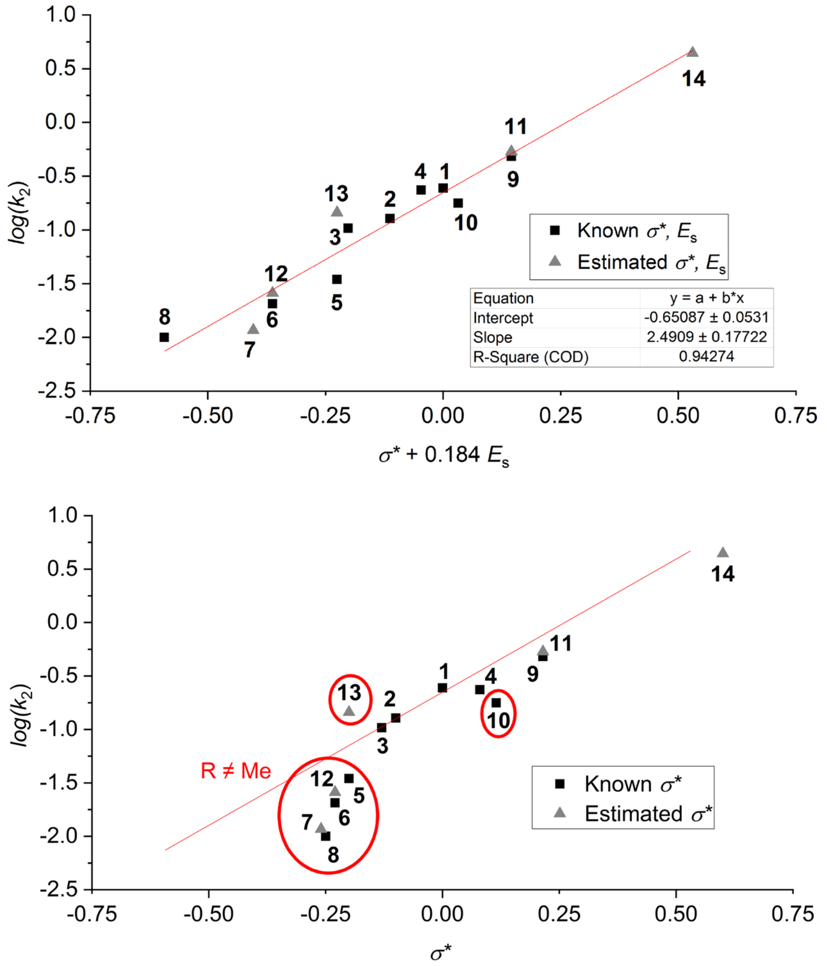

Figure S53. The correlations of the logarithm of rate constants k_2 with Taft parameter (σ*) and steric contribution (E_s) (top) or Taft parameter alone (bottom).[6] Fittings were performed on all data points in the top plot, and reproduced on the bottom plot for comparison. The values of σ* + E_s are estimated for some cases where data is not available: **7** = 2 × n-Bu; **11** = Bz and Me; **12** = 2 × Pr; **13** = 2 × Et; **14** = Me and Ph. Compounds with bulk R groups (i.e. where R ≠ Me) are circled in red.

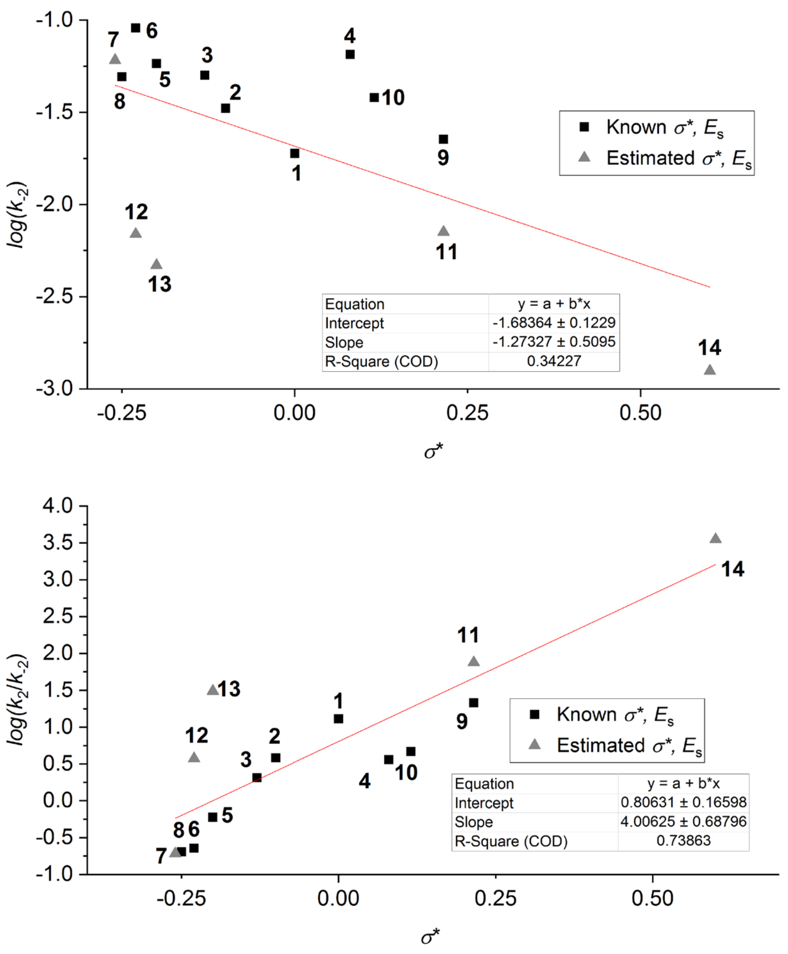

Figure S54. The correlations of the logarithm of rate constants k_{-2} and k_2/k_{-2} with Taft parameter (σ*) and steric contribution (E_s).[6] All fittings were performed on all data points. The values of σ* + E_s are estimated for some cases where data is not available: **7** = 2 × n-Bu; **11** = Bz and Me; **12** = 2 × Pr; **13** = 2 × Et; **14** = Me and Ph.

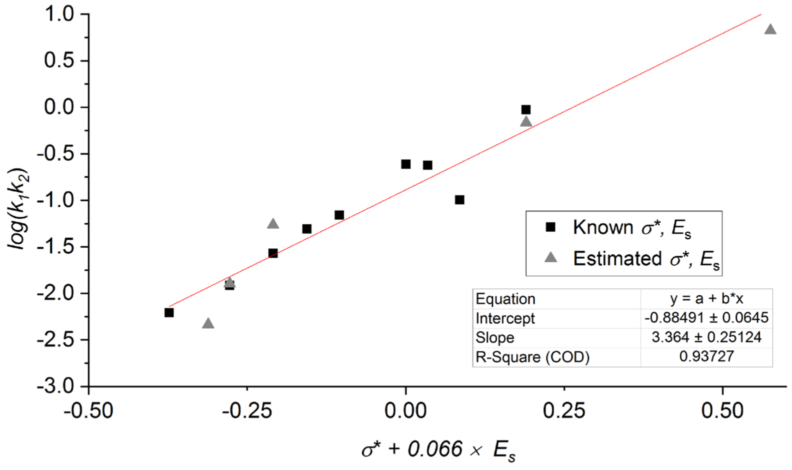

Figure S55. The correlations of the logarithm of the product of rate constants k_1 and k_2 with Taft parameter (σ*) and steric contribution (E_s).[6] All fittings were performed on all data points. The values of σ* + E_s are estimated for some cases where data is not available: **7** = 2 × n-Bu; **11** = Bz and Me; **12** = 2 × Pr; **13** = 2 × Et; **14** = Me and Ph.

26 UV-vis absorption spectroscopy

Absorption spectra of compound **1**–**14** were measured in chloroform and MeTHF.

UV-vis experiments were performed on an Agilent Cary 60 Bio UV-Visible Spectrophotometer equipped with a customized Cary Single Cell Peltier Accessory, keeping the samples at 25 °C, unless stated otherwise. The cell holder was modified to allow for irradiation perpendicular to the direction of measurement, as previously described.[1] A Luxeon Rebel LED (lime, 567 nm, operated at 12 V, 1000 mA) was mounted on a heat sink positioned 4 cm away from the cell, and the beam was focused on the cuvette using a Carclo 20.0 mm Fibre Coupling Lens. All samples were stirred to ensure homogeneity. A timer relay module (FRM01) was used to control the irradiation cycles. The emission of the LED was measured using an Ocean Optics HR4000 high-resolution spectrometer.

26.1 Emission profile of the LED lamp

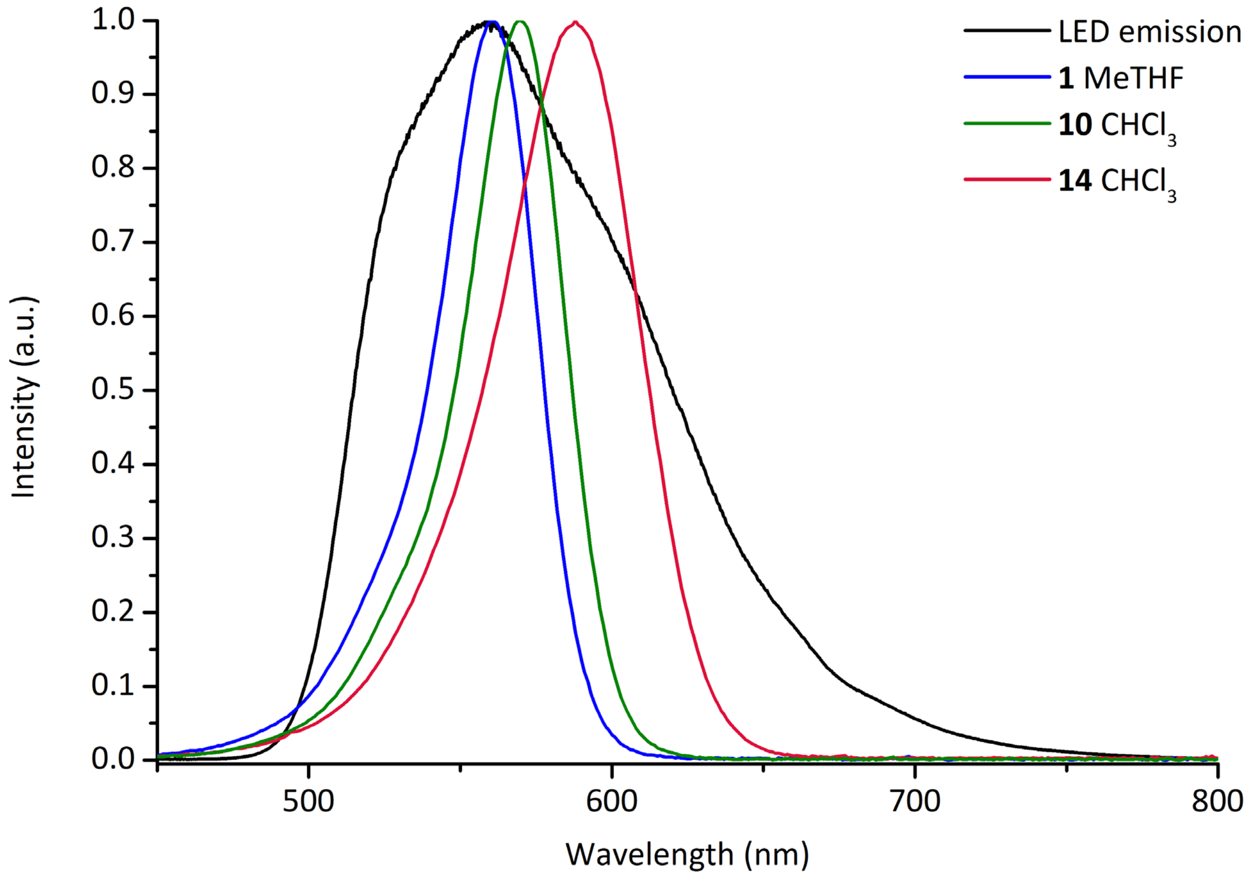

Figure S56. Normalized emission spectrum (black line) of the LED (Luxeon Rebel, lime, 567 nm) used in the photoswitching experiments. Absorption spectra of **1** in MeTHF (blue line, most blue-shifted λ_{max}), **10** in $CHCl_3$ (most red-shifted aliphatic derivative, green line) and **14** in $CHCl_3$ (most red-shifted derivative, crimson line) show good overlap with the LED emission.

27 Single switching cycles (full spectra) in $CHCl_3$

27.1 UV-vis spectra of 1 in chloroform during one photoswitching cycle

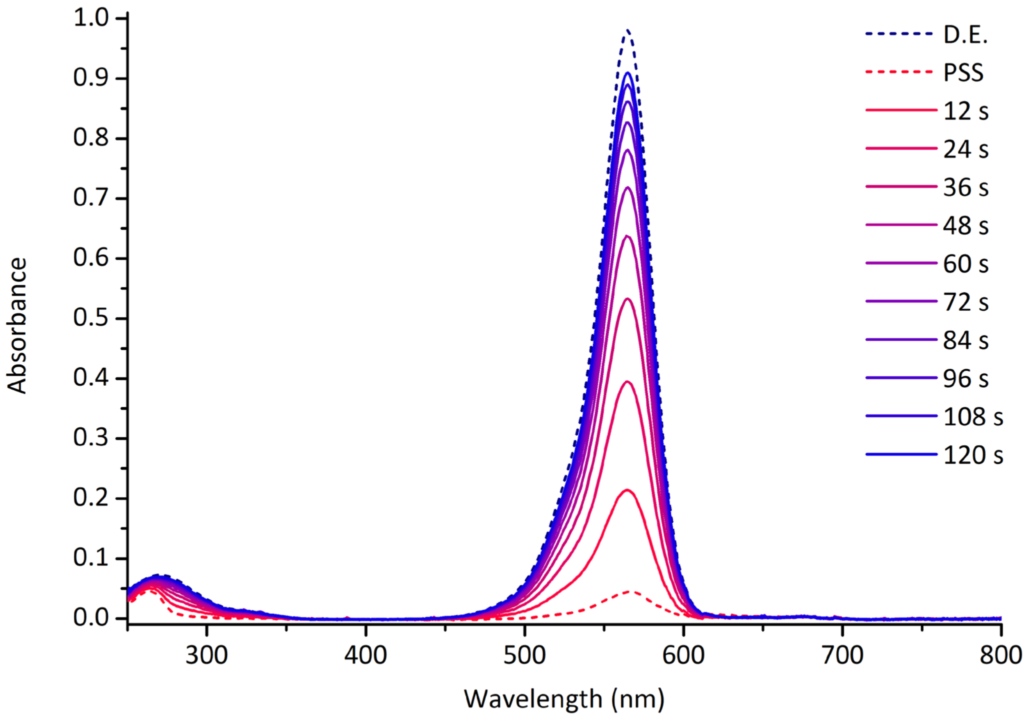

Figure S57. Photoswitching of **1** in $CHCl_3$, followed by UV-vis spectroscopy. Before the switching cycle the sample was kept in the dark to reach a dark equilibrium (D.E.) state. The sample was irradiated (567 nm LED) for one minute, during which it reached a photostationary state (PSS), followed by 2 min in the dark. The times refer to time since the irradiation is switched off.

27.2 UV-vis spectra of 2 in chloroform during one photoswitching cycle

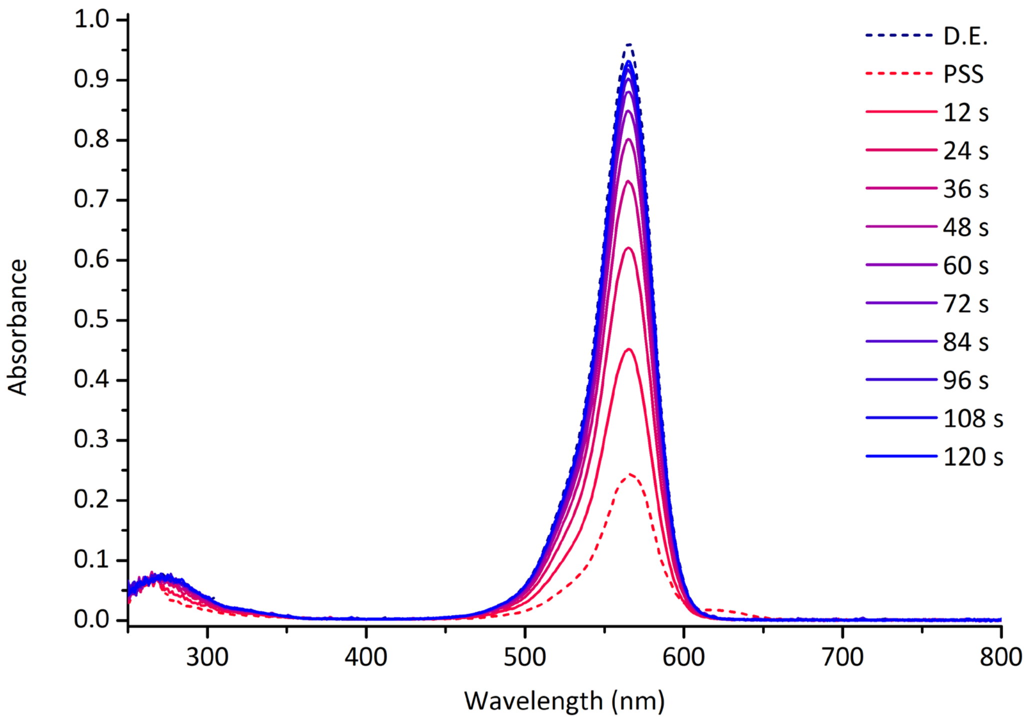

Figure S58. Photoswitching of **2** in $CHCl_3$, followed by UV-vis spectroscopy. Before the switching cycle the sample was kept in the dark to reach a dark equilibrium (D.E.) state. The sample was irradiated (567 nm LED) for one minute, during which it reached a photostationary state (PSS), followed by 2 min in the dark. The times refer to time since the irradiation is switched off.

27.3 UV-vis spectra of 3 in chloroform during one photoswitching cycle

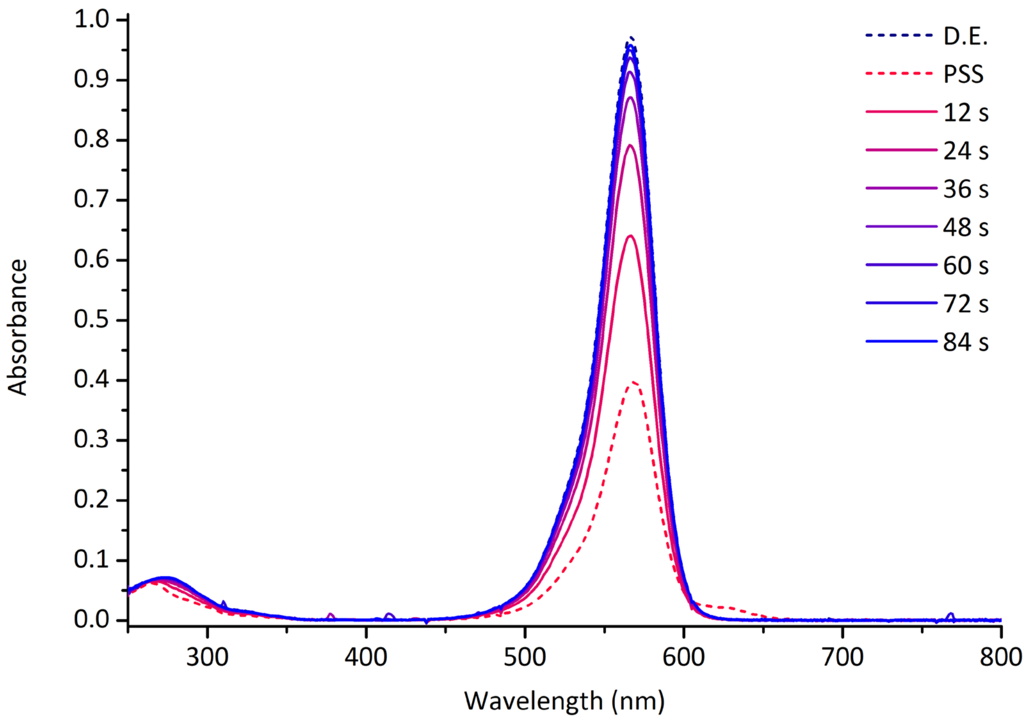

Figure S59. Photoswitching of **3** in $CHCl_3$, followed by UV-vis spectroscopy. Before the switching cycle the sample was kept in the dark to reach a dark equilibrium (D.E.) state. The sample was irradiated (567 nm LED) for one minute, during which it reached a photostationary state (PSS), followed by 84 s in the dark. The times refer to time since the irradiation is switched off.

27.4 UV-vis spectra of 4 in chloroform during one photoswitching cycle

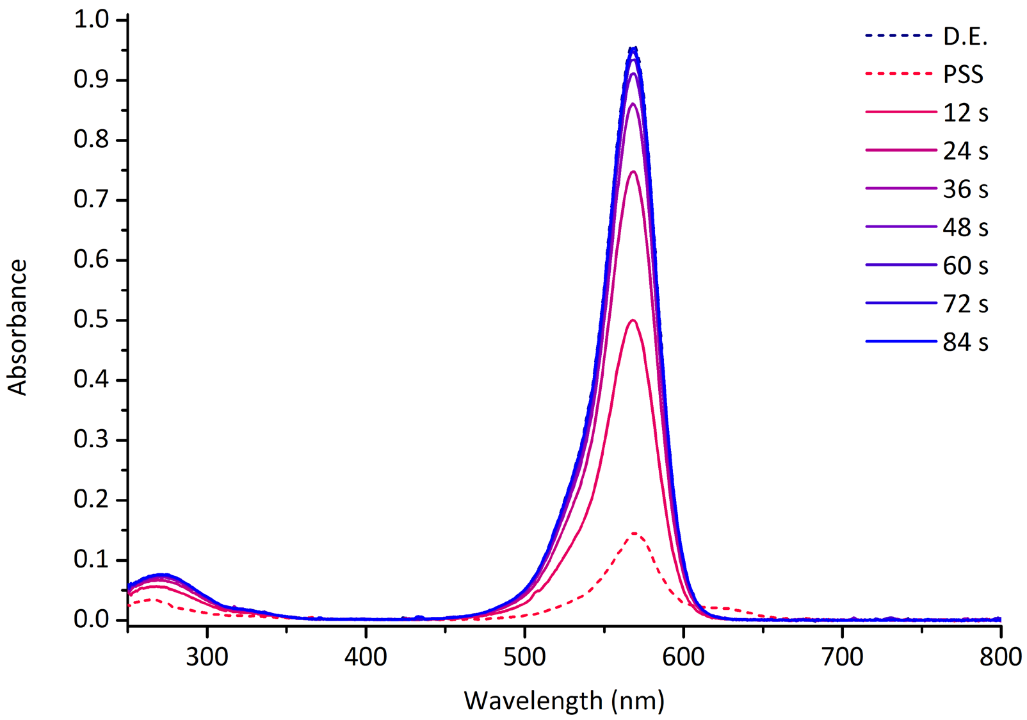

Figure S60. Photoswitching of **4** in $CHCl_3$, followed by UV-vis spectroscopy. Before the switching cycle the sample was kept in the dark to reach a dark equilibrium (D.E.) state. The sample was irradiated (567 nm LED) for one minute, during which it reached a photostationary state (PSS), followed by 84 s in the dark. The times refer to time since the irradiation is switched off.

27.5 UV-vis spectra of 5 in chloroform during one photoswitching cycle

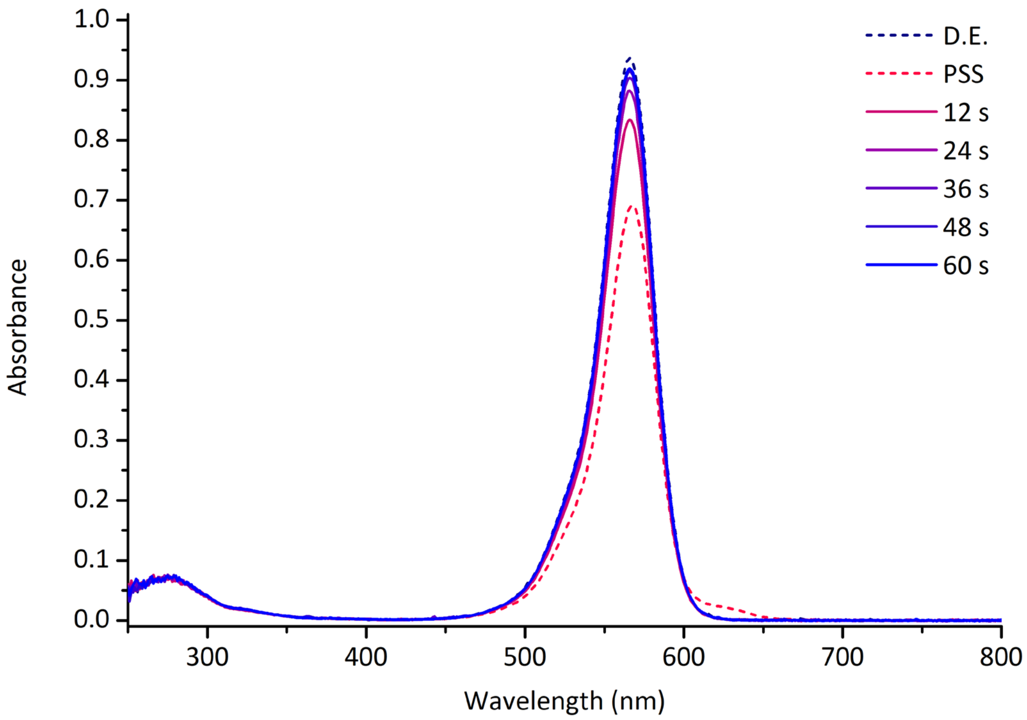

Figure S61. Photoswitching of **5** in $CHCl_3$, followed by UV-vis spectroscopy. Before the switching cycle the sample was kept in the dark to reach a dark equilibrium (D.E.) state. The sample was irradiated (567 nm LED) for one minute, during which it reached a photostationary state (PSS), followed by 1 min in the dark. The times refer to time since the irradiation is switched off.

27.6 UV-vis spectra of 6 in chloroform during one photoswitching cycle

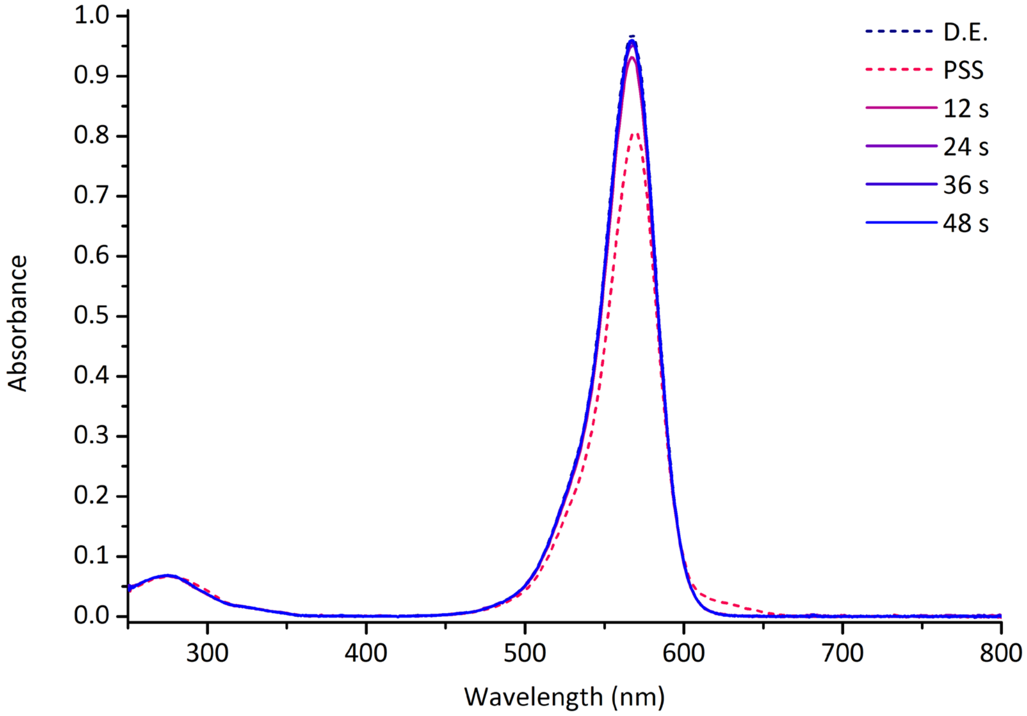

Figure S62. Photoswitching of **6** in $CHCl_3$, followed by UV-vis spectroscopy. Before the switching cycle the sample was kept in the dark to reach a dark equilibrium (D.E.) state. The sample was irradiated (567 nm LED) for one minute, during which it reached a photostationary state (PSS), followed by 48 s in the dark. The times refer to time since the irradiation is switched off.

27.7 UV-vis spectra of 7 in chloroform during one photoswitching cycle

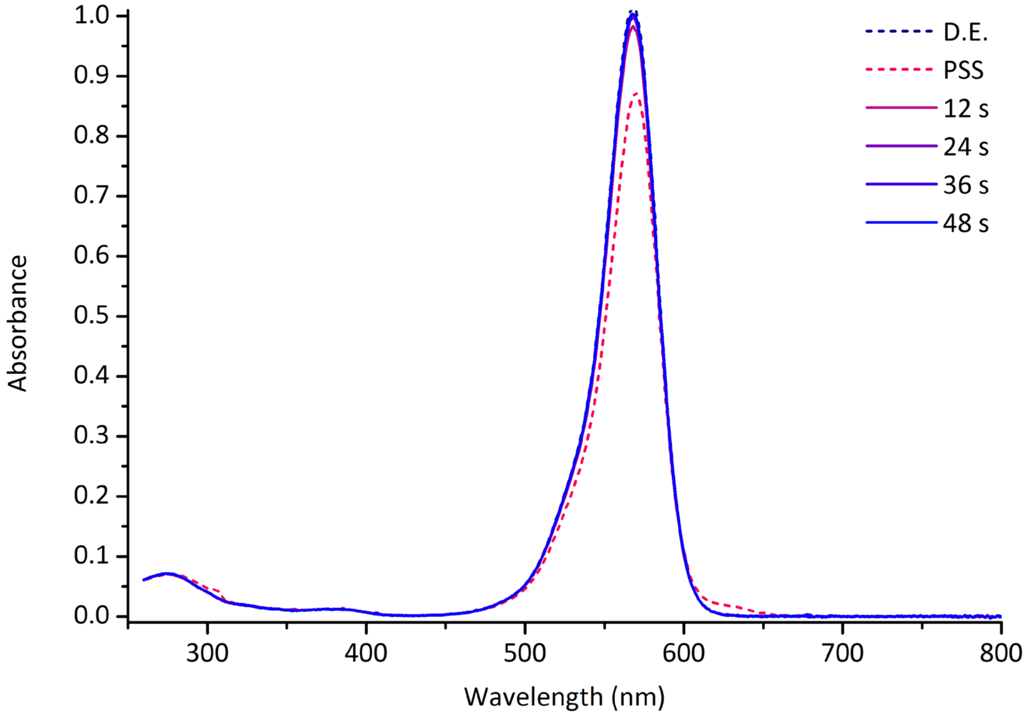

Figure S63. Photoswitching of **7** in $CHCl_3$, followed by UV-vis spectroscopy. Before the switching cycle the sample was kept in the dark to reach a dark equilibrium (D.E.) state. The sample was irradiated (567 nm LED) for one minute, during which it reached a photostationary state (PSS), followed by 48 s in the dark. The times refer to time since the irradiation is switched off.

27.8 UV-vis spectra of 8 in chloroform during one photoswitching cycle

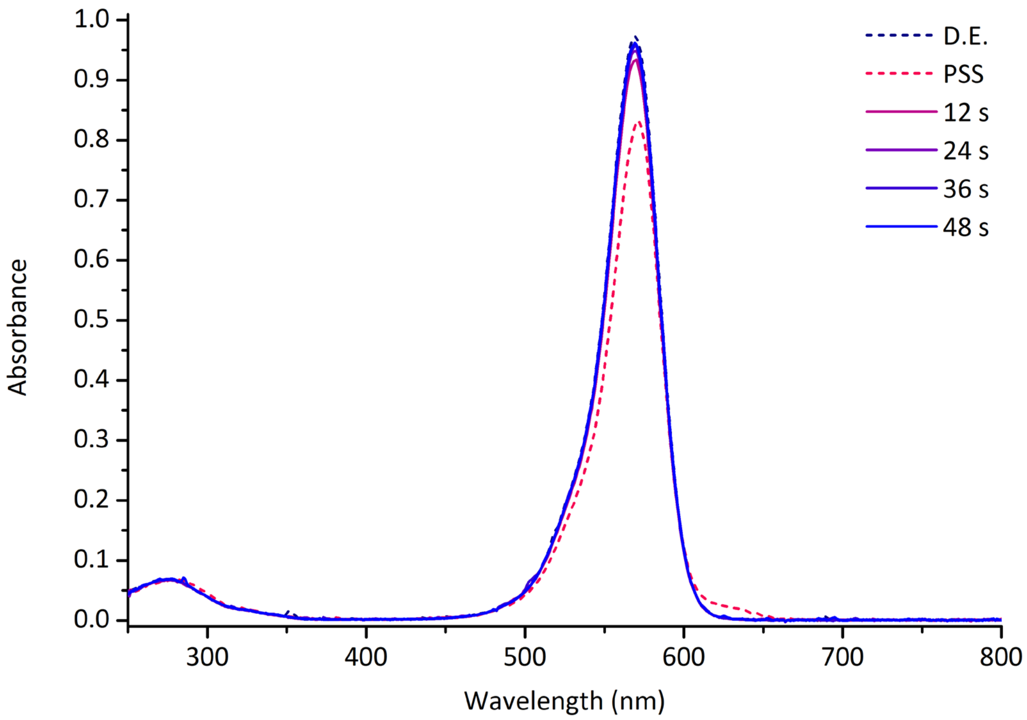

Figure S64. Photoswitching of **8** in $CHCl_3$, followed by UV-vis spectroscopy. Before the switching cycle the sample was kept in the dark to reach a dark equilibrium (D.E.) state. The sample was irradiated (567 nm LED) for one minute, during which it reached a photostationary state (PSS), followed by 48 s in the dark. The times refer to time since the irradiation is switched off.

27.9 UV-vis spectra of 9 in chloroform during one photoswitching cycle

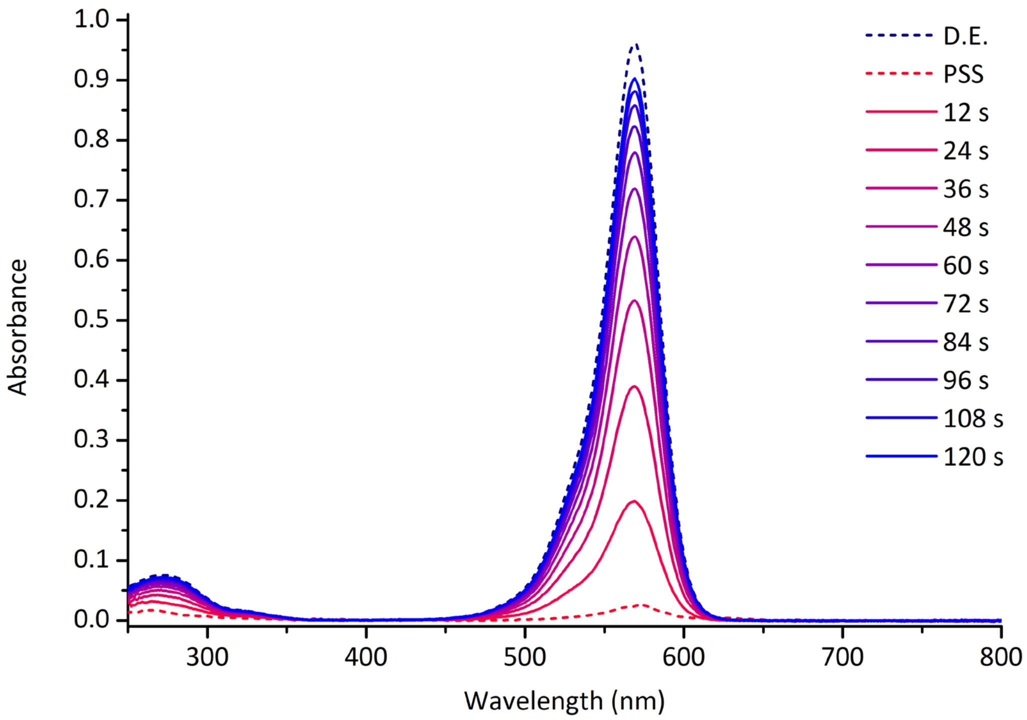

Figure S65. Photoswitching of **9** in $CHCl_3$, followed by UV-vis spectroscopy. Before the switching cycle the sample was kept in the dark to reach a dark equilibrium (D.E.) state. The sample was irradiated (567 nm LED) for one minute, during which it reached a photostationary state (PSS), followed by 2 min in the dark. The times refer to time since the irradiation is switched off.

27.10 UV-vis spectra of 10 in chloroform during one photoswitching cycle

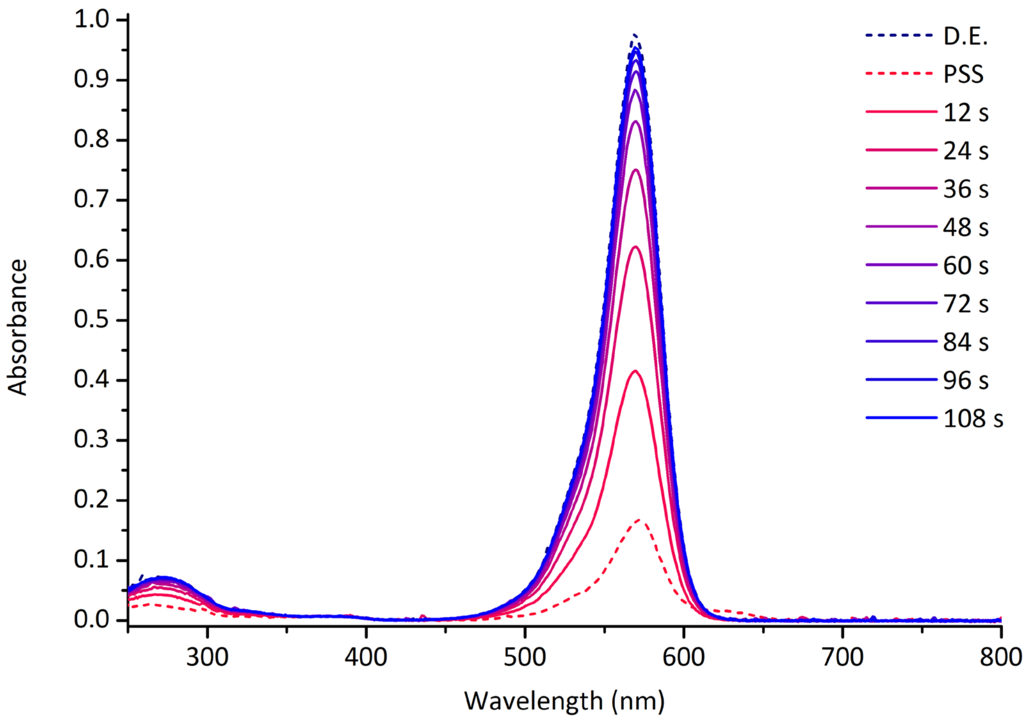

Figure S66. Photoswitching of **10** in $CHCl_3$, followed by UV-vis spectroscopy. Before the switching cycle the sample was kept in the dark to reach a dark equilibrium (D.E.) state. The sample was irradiated (567 nm LED) for one minute, during which it reached a photostationary state (PSS), followed by 108 s in the dark. The times refer to time since the irradiation is switched off.

27.11 UV-vis spectra of 11 in chloroform during one photoswitching cycle

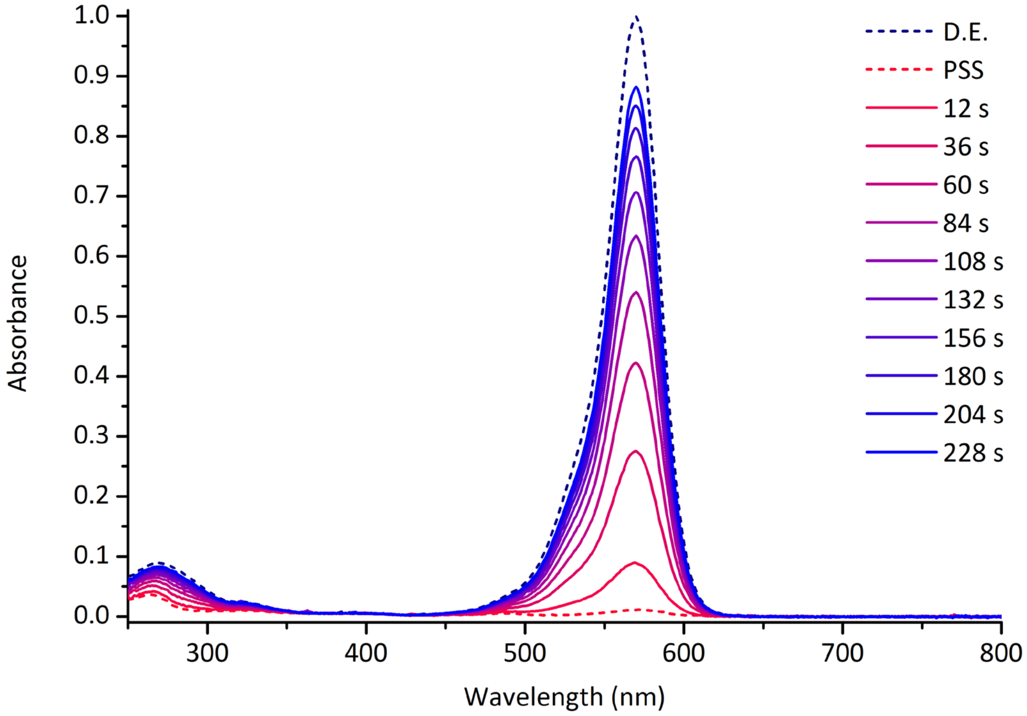

Figure S67. Photoswitching of **11** in $CHCl_3$, followed by UV-vis spectroscopy. Before the switching cycle the sample was kept in the dark to reach a dark equilibrium (D.E.) state. The sample was irradiated (567 nm LED) for one minute, during which it reached a photostationary state (PSS), followed by 228 s in the dark. The times refer to time since the irradiation is switched off.

27.12 UV-vis spectra of 12 in chloroform during one photoswitching cycle

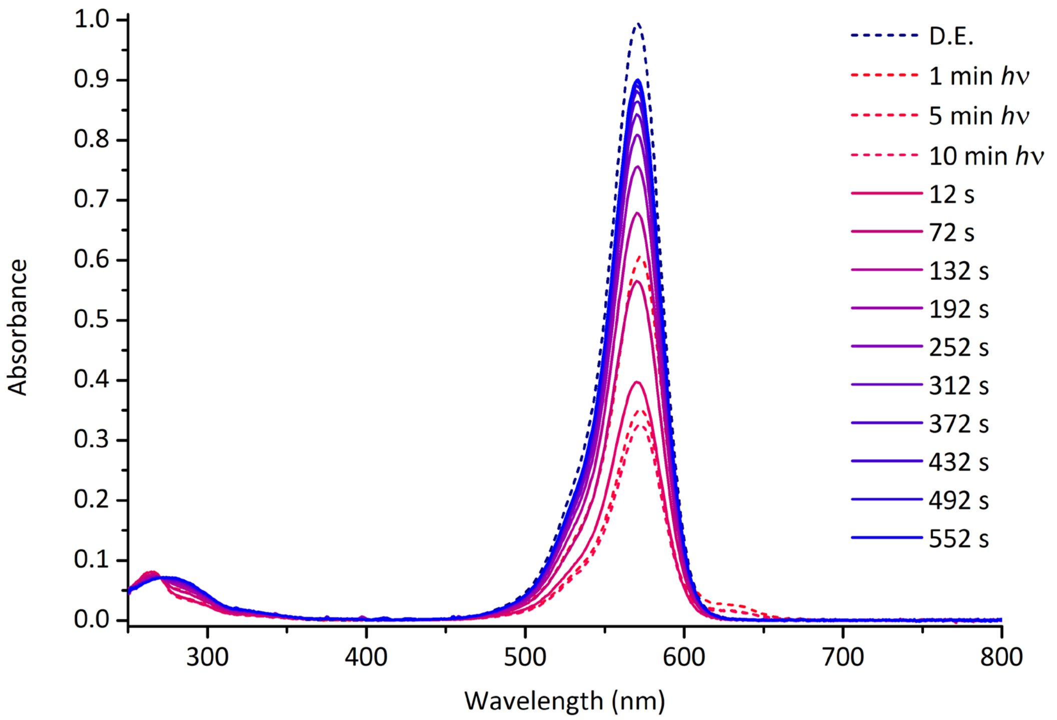

Figure S68. Photoswitching of **12** in $CHCl_3$, followed by UV-vis spectroscopy. Before the switching cycle the sample was kept in the dark to reach a dark equilibrium (D.E.) state. After one minute of irradiation (567 nm LED), the sample did not reach a photostationary state (PSS). After five minutes of irradiation, the sample appears to have reached a PSS, and further irradiation results in a decrease in absorbance due to photodecomposition. After the thermal reversion the decomposition is evident as the initial absorbance is not regained. The times refer to time since the irradiation is switched off.

27.13 UV-vis spectra of 13 in chloroform during one photoswitching cycle

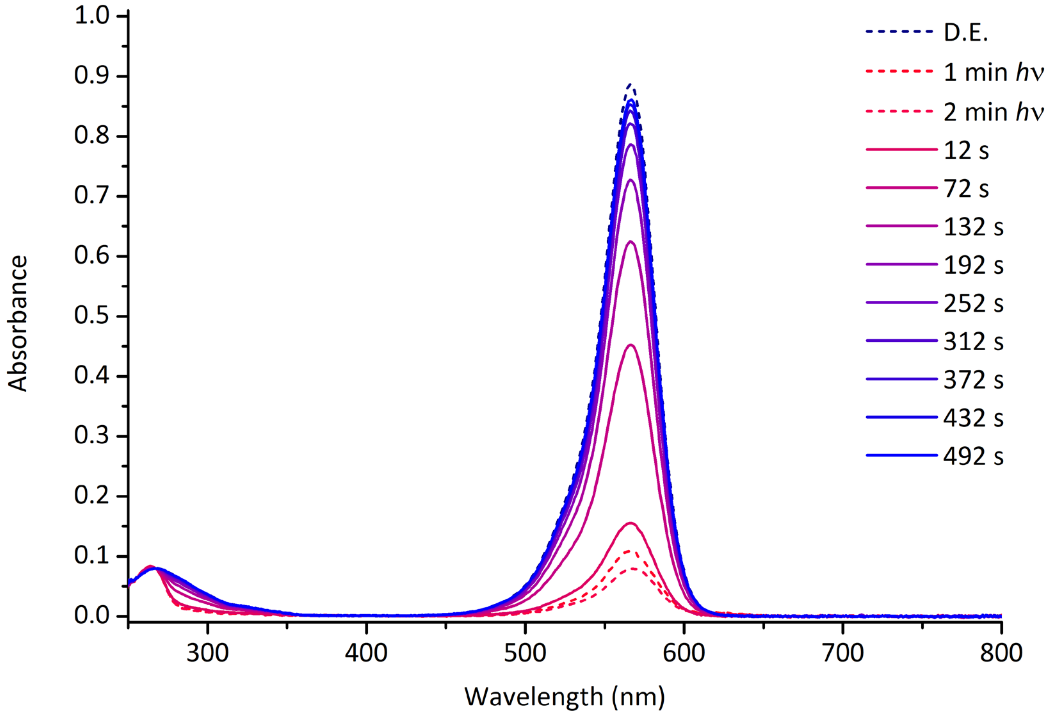

Figure S69. Photoswitching of **13** in $CHCl_3$, followed by UV-vis spectroscopy. Before the switching cycle the sample was kept in the dark to reach a dark equilibrium (D.E.) state. The sample was irradiated (567 nm LED) for two minutes, during which it reached a photostationary state (PSS), followed by 8 min in the dark. The times refer to time since the irradiation is switched off.

27.14 UV-vis spectra of 14 in chloroform during one photoswitching cycle

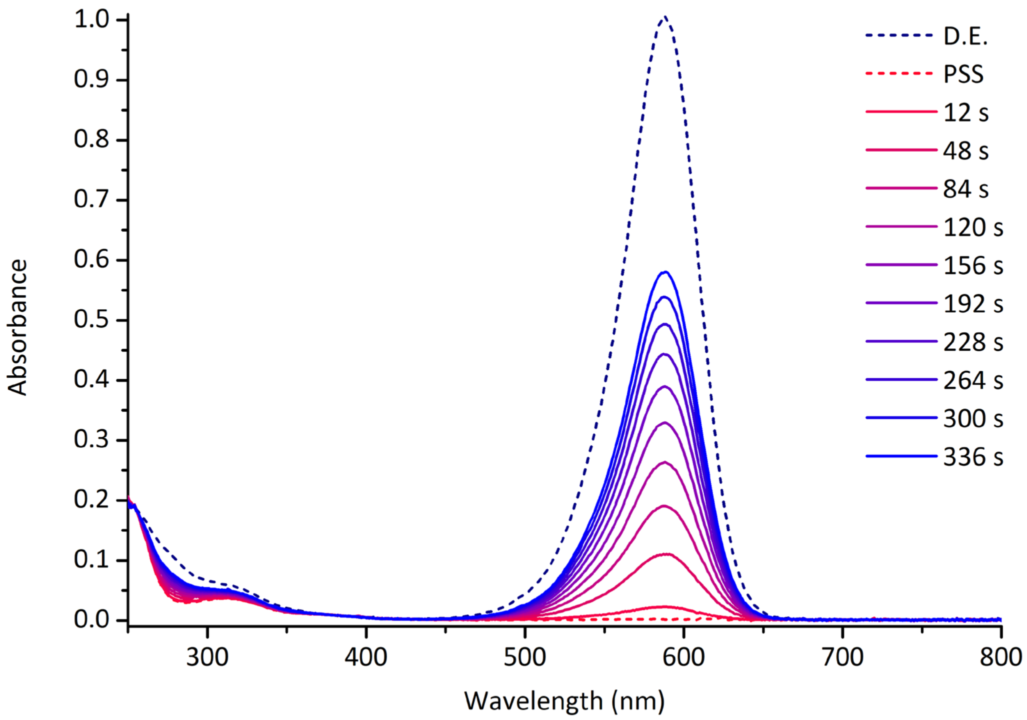

Figure S70. Photoswitching of **14** in $CHCl_3$, followed by UV-vis spectroscopy. Before the switching cycle the sample was kept in the dark to reach a dark equilibrium (D.E.) state. The sample was irradiated (567 nm LED) for one minute, during which it reached a photostationary state (PSS), followed by 336 s in the dark. The times refer to time since the irradiation is switched off.

28 Fatigue resistance and thermal half-life calculations in chloroform

UV-vis experiments were performed on an Agilent Cary 60 Bio UV-Visible Spectrophotometer equipped with a customized Cary Single Cell Peltier Accessory, keeping the samples at 25 °C, unless stated otherwise. The cell holder was modified to allow for irradiation perpendicular to the direction of measurement, as previously described.[1] A Luxeon Rebel LED (lime, 567 nm, operated at 12 V, 1000 mA) was mounted on a heat sink positioned 4 cm away from the cell, and the beam was focused on the cuvette using a Carclo 20.0 mm Fibre Coupling Lens. All samples were stirred to ensure homogeneity. A timer relay module (FRM01) was used to control the irradiation cycles.

Fatigue measurements for compounds **1**–**14** were conducted in $CHCl_3$, using the same conditions and same solvent bottle for all samples. The absorbance of the solutions was 0.95 ± 0.05 at the start of the experiment, measured at λ_{max} (see Table S4). After equilibrating in the dark, the absorbance was measured for 1 hour without irradiating to analyse for any the decomposition in the dark. Subsequently the samples were subjected to 100 photoswitching cycles of 45 s irradiation (567 nm LED) followed by 300 s of darkness. After the 100 cycles the absorbance was measured for a further 60 minutes without irradiation to analyse for any further decomposition in the dark. In all cases the DASAs were found to be stable in the dark, with decomposition half-lives of 4-30 days. The temperature of the samples was kept at 298 K during the entire measurement.

Exponential regression analysis (performed in Origin) was used to determine the apparent thermal half-life times ($t_{½}$) of the first and last switching cycles. A curve of the form $y = y_0 + Ae^{(R_0 * x)}$ was fitted to the data, where y = absorbance, y_0 = y offset, A = amplitude, R_0 = rate constant and x = time in minutes. From this fit, the apparent thermal half-life time (in seconds) was derived as $t_{½} = \frac{60 * \ln 0.5}{R_0}$.

28.1 Fatigue resistance and thermal half-life calculation for 1 in chloroform

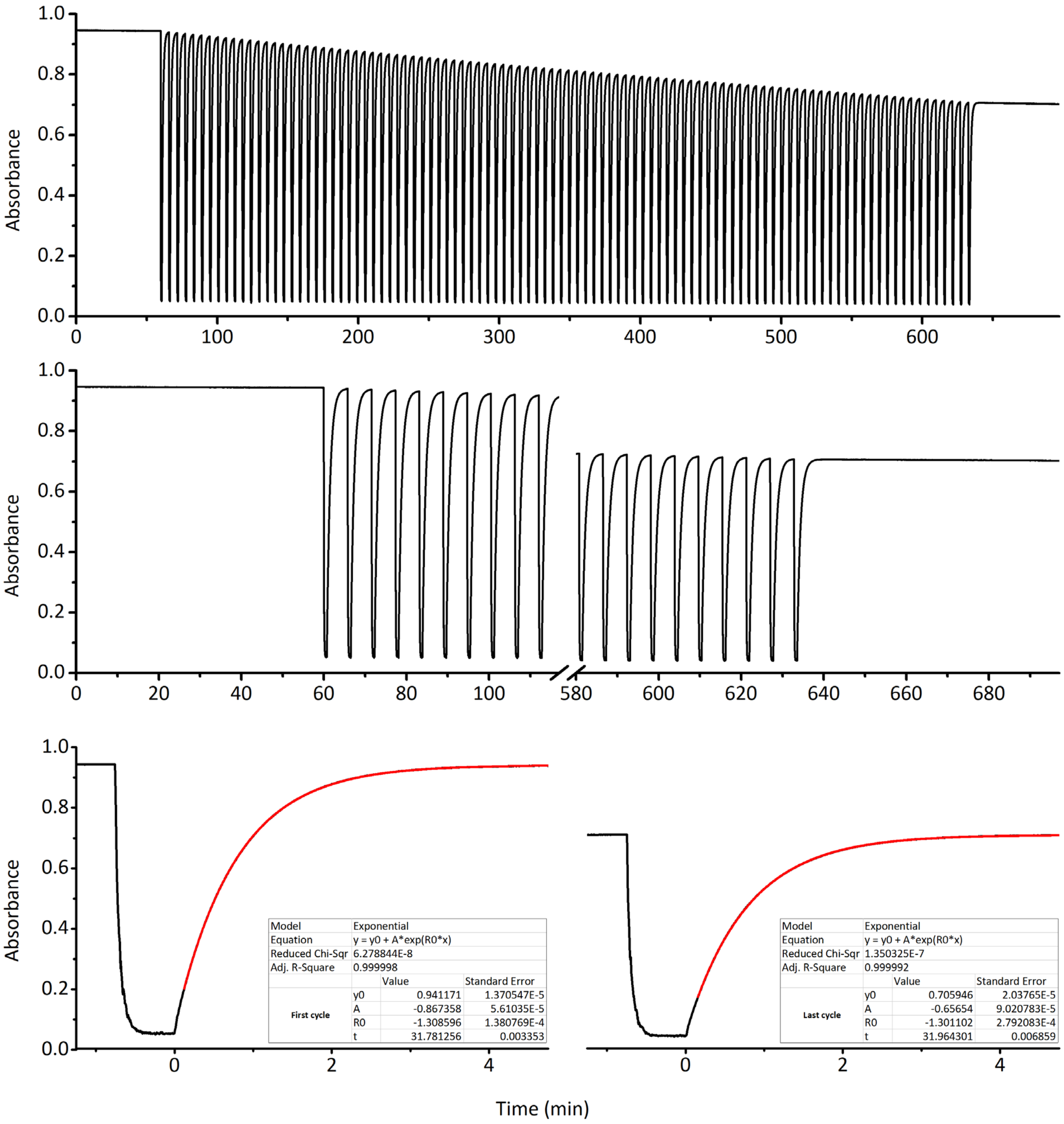

Figure S71. The fatigue resistance of **1** ($CHCl_3$, 298 K) during 100 switching cycles of irradiation by 567 nm light. The absorbance (black lines) was measured at λ_{max} = 565 nm. Top: The complete 100 switching cycles. The first hour of measurement was in the dark and no decomposition was observed. Middle: Expansion of the first 10 and last 10 switching cycles. Bottom: Expansions of the first (left) and last (right) switching cycle with exponential curves (red) fitted to determine the apparent thermal half-life time (table inserts, time in s).

28.2 Fatigue resistance and thermal half-life calculation for 2 in chloroform

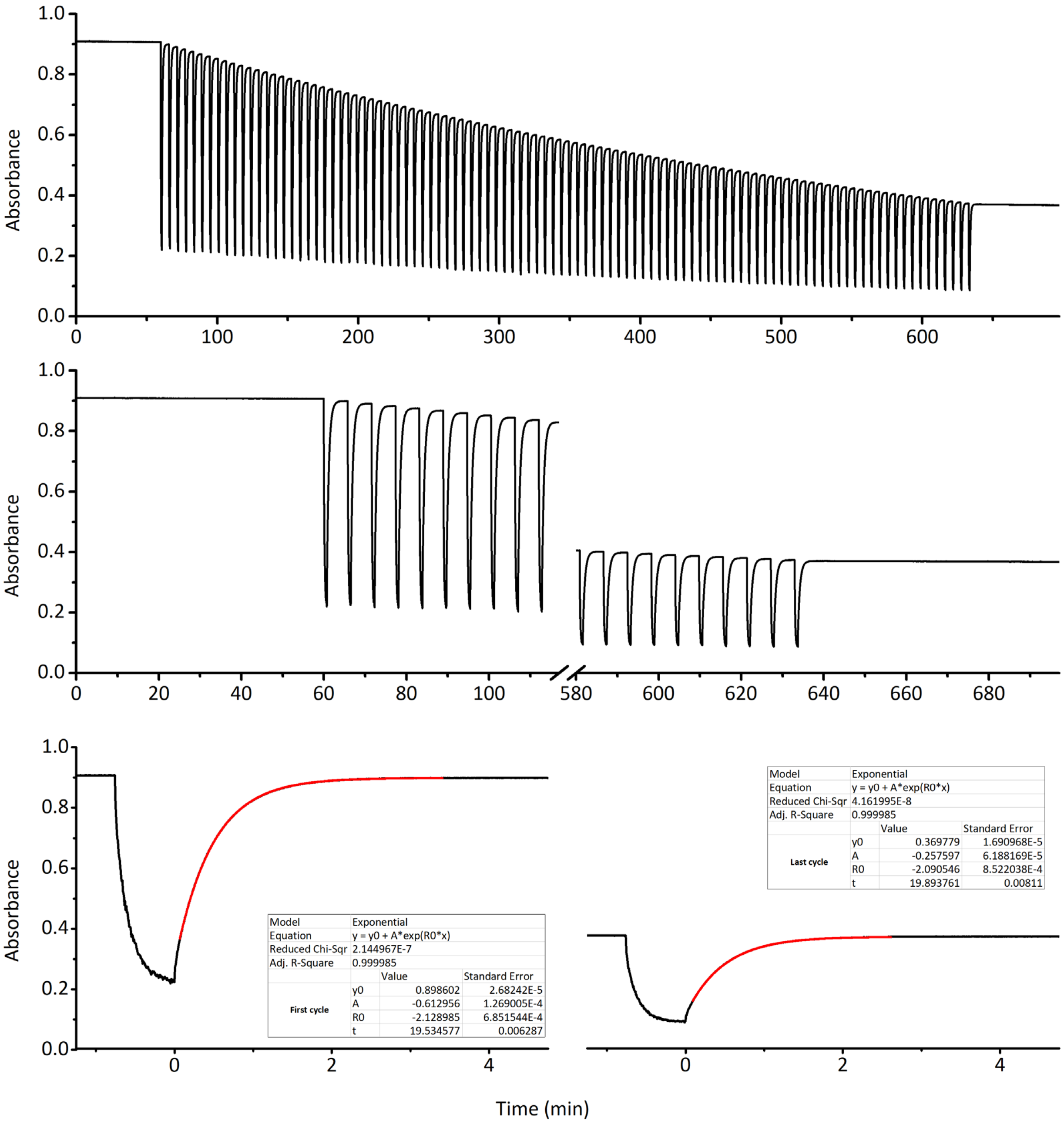

Figure S72. The fatigue resistance of **2** ($CHCl_3$, 298 K) during 100 switching cycles of irradiation by 567 nm light. The absorbance (black lines) was measured at λ_{max} = 565 nm. Top: The complete 100 switching cycles. The first hour of measurement was in the dark and no decomposition was observed. Middle: Expansion of the first 10 and last 10 switching cycles. Bottom: Expansions of the first (left) and last (right) switching cycle with exponential curves (red) fitted to determine the apparent thermal half-life time (table inserts, time in s).

28.3 Fatigue resistance and thermal half-life calculation for 3 in chloroform

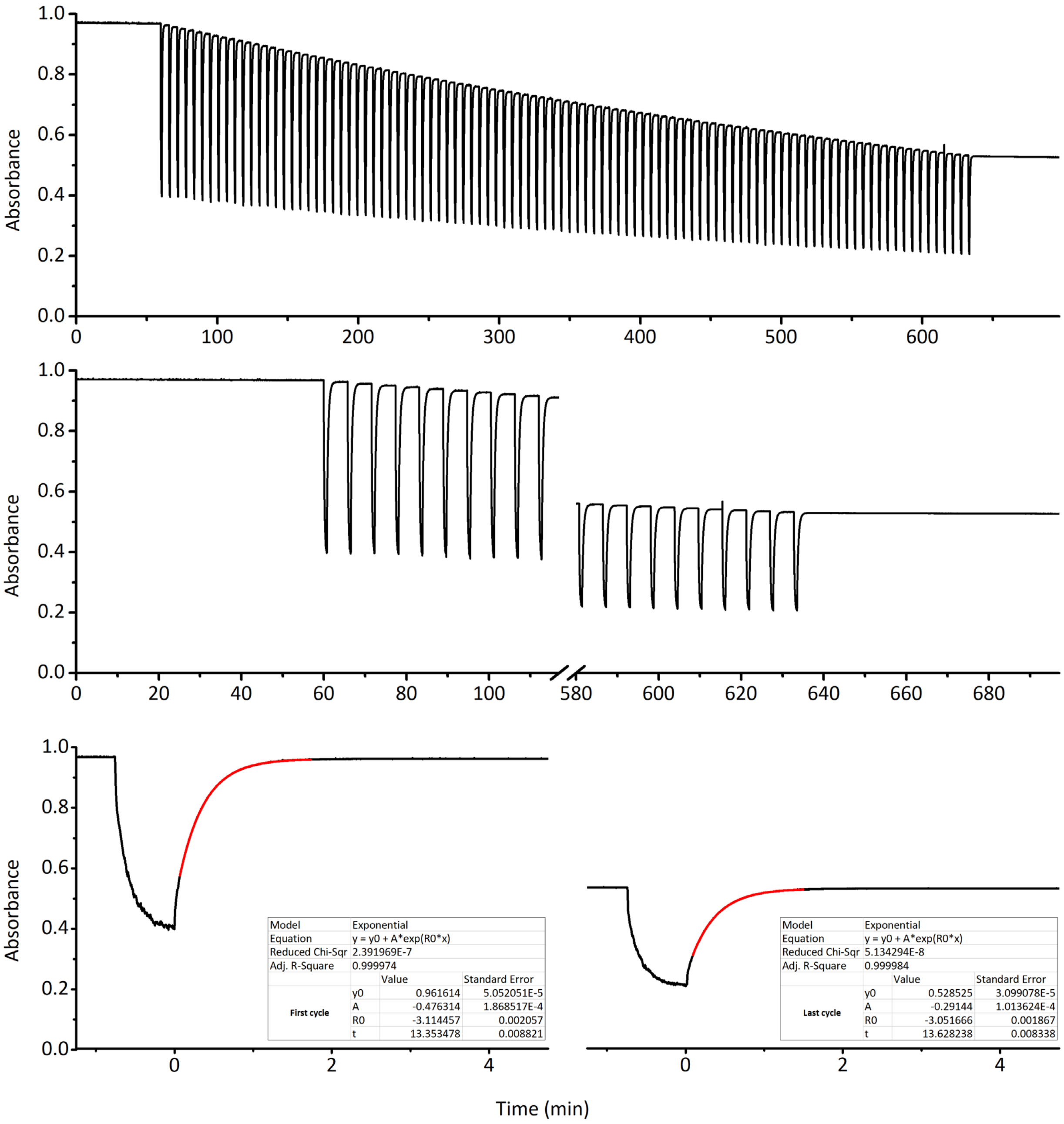

Figure S73. The fatigue resistance of **3** ($CHCl_3$, 298 K) during 100 switching cycles of irradiation by 567 nm light. The absorbance (black lines) was measured at λ_{max} = 566 nm. Top: The complete 100 switching cycles. The first hour of measurement was in the dark and no decomposition was observed. Middle: Expansion of the first 10 and last 10 switching cycles. Bottom: Expansions of the first (left) and last (right) switching cycle with exponential curves (red) fitted to determine the apparent thermal half-life time (table inserts, time in s).

28.4 Fatigue resistance and thermal half-life calculation for 4 in chloroform

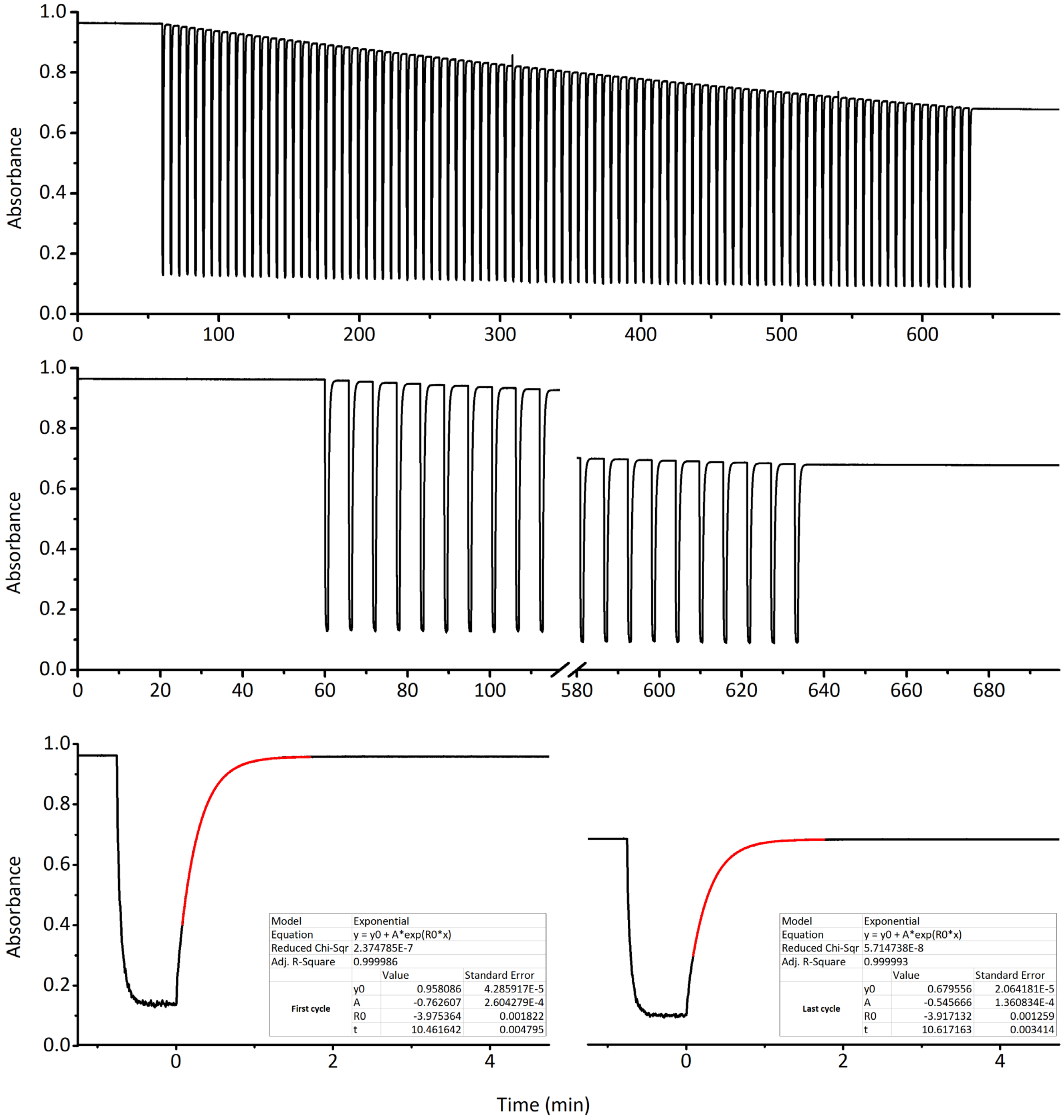

Figure S74. The fatigue resistance of **4** ($CHCl_3$, 298 K) during 100 switching cycles of irradiation by 567 nm light. The absorbance (black lines) was measured at λ_{max} = 568 nm. Top: The complete 100 switching cycles. The first hour of measurement was in the dark and no decomposition was observed. Middle: Expansion of the first 10 and last 10 switching cycles. Bottom: Expansions of the first (left) and last (right) switching cycle with exponential curves (red) fitted to determine the apparent thermal half-life time (table inserts, time in s).

28.5 Fatigue resistance and thermal half-life calculation for 5 in chloroform

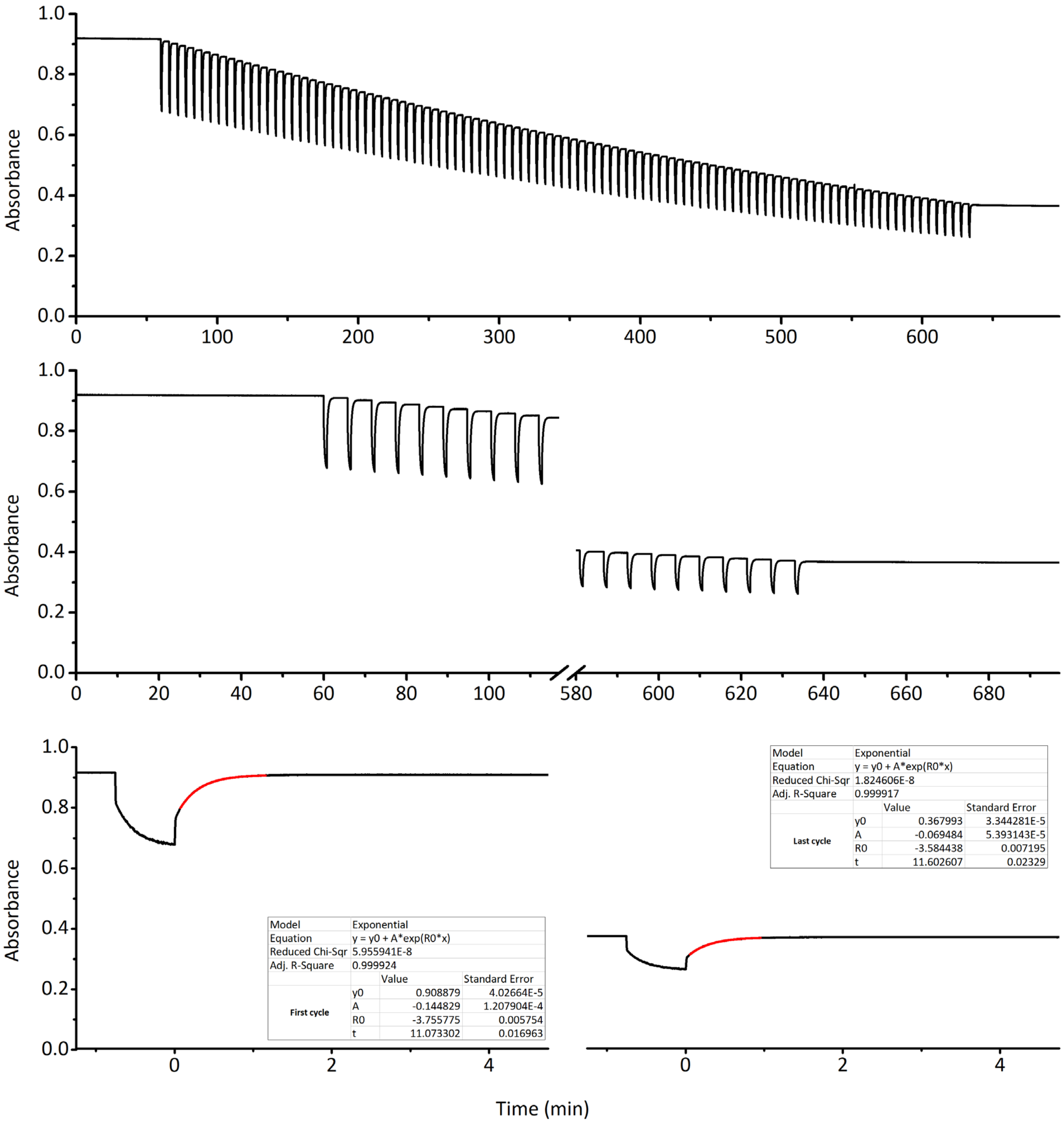

Figure S75. The fatigue resistance of **5** ($CHCl_3$, 298 K) during 100 switching cycles of irradiation by 567 nm light. The absorbance (black lines) was measured at λ_{max} = 565 nm. Top: The complete 100 switching cycles. The first hour of measurement was in the dark and no decomposition was observed. Middle: Expansion of the first 10 and last 10 switching cycles. Bottom: Expansions of the first (left) and last (right) switching cycle with exponential curves (red) fitted to determine the apparent thermal half-life time (table inserts, time in s).

28.6 Fatigue resistance and thermal half-life calculation for 6 in chloroform

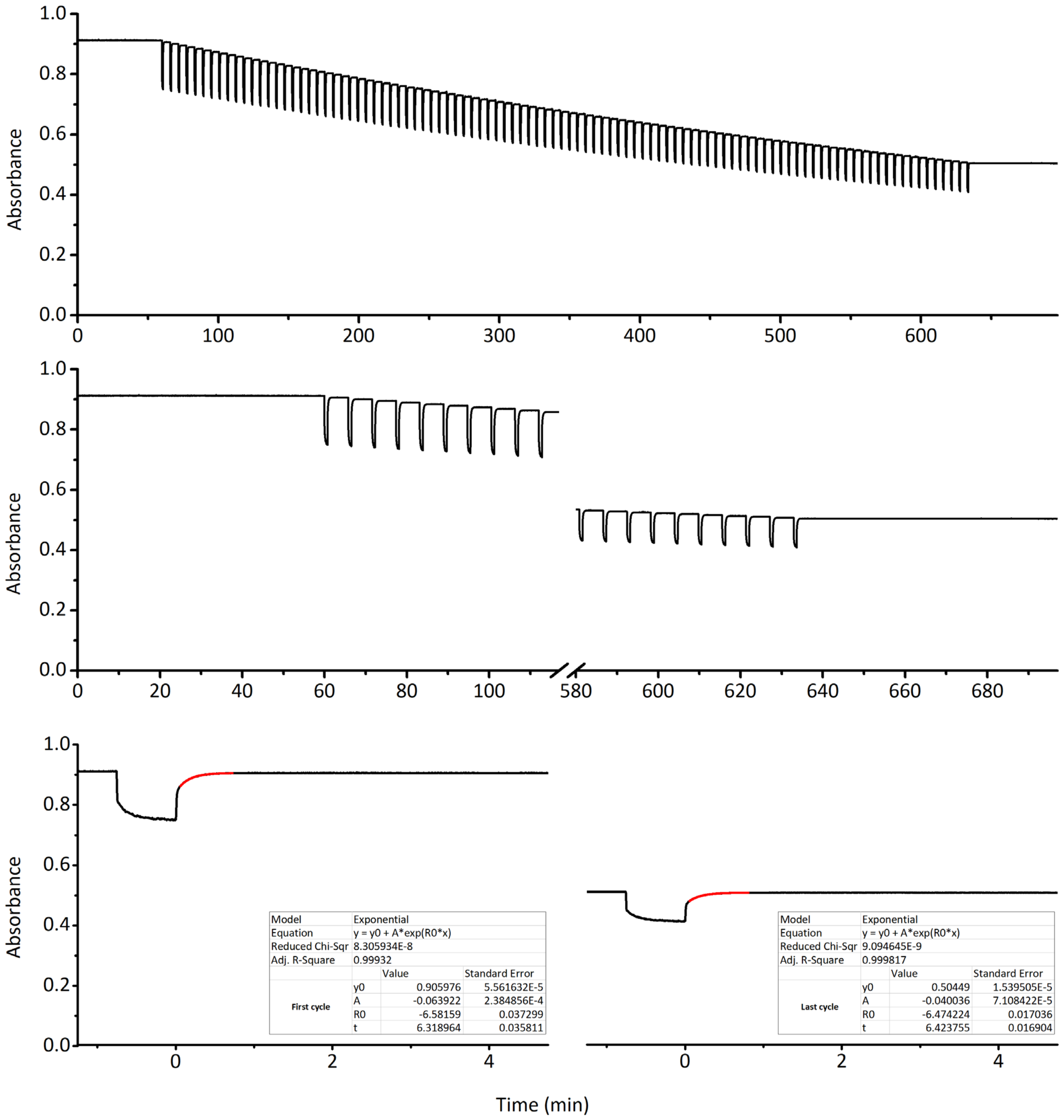

Figure S76. The fatigue resistance of **6** ($CHCl_3$, 298 K) during 100 switching cycles of irradiation by 567 nm light. The absorbance (black lines) was measured at λ_{max} = 567 nm. Top: The complete 100 switching cycles. The first hour of measurement was in the dark and no decomposition was observed. Middle: Expansion of the first 10 and last 10 switching cycles. Bottom: Expansions of the first (left) and last (right) switching cycle with exponential curves (red) fitted to determine the apparent thermal half-life time (table inserts, time in s).

28.7 Fatigue resistance and thermal half-life calculation for 7 in chloroform

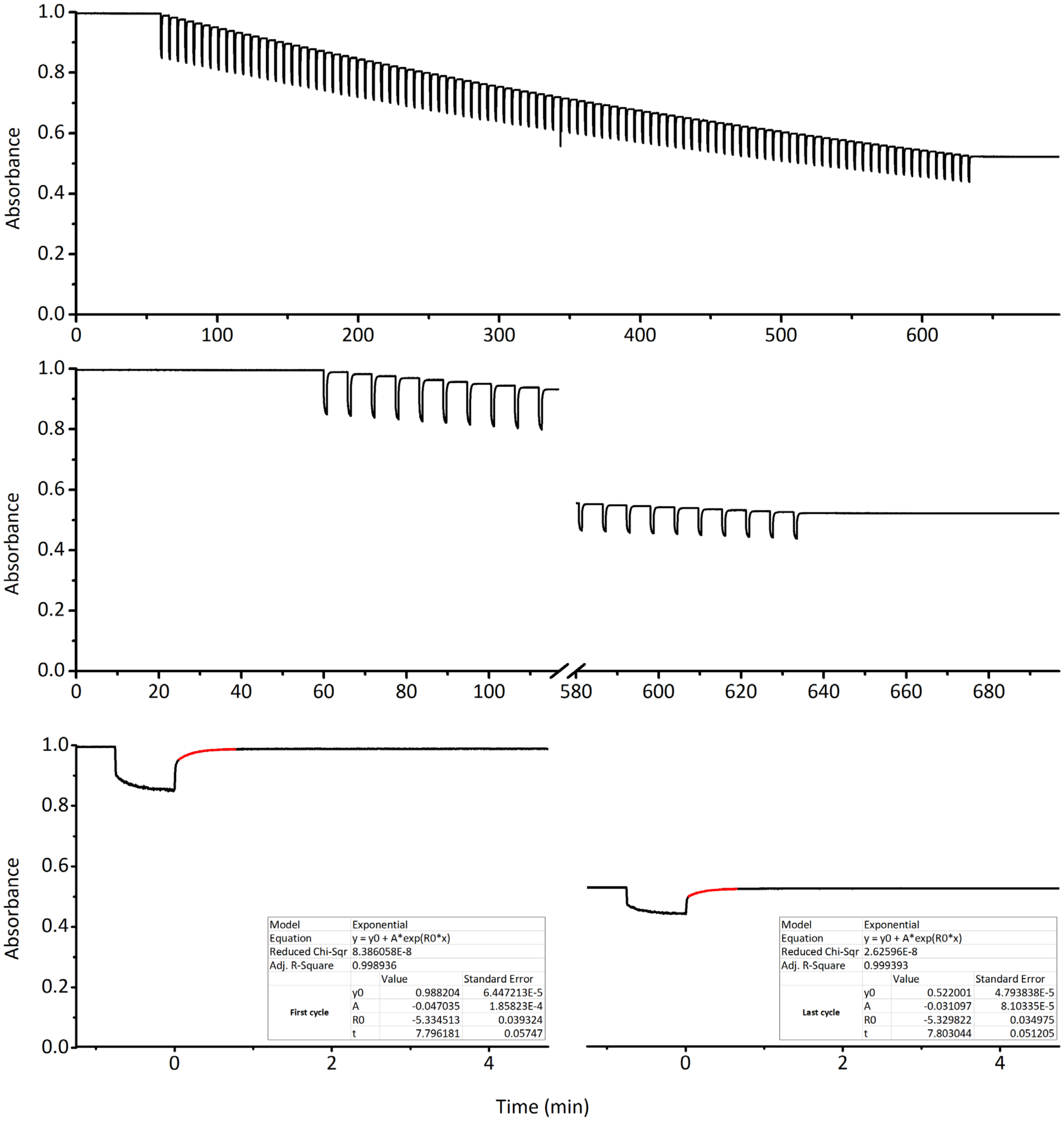

Figure S77. The fatigue resistance of **7** ($CHCl_3$, 298 K) during 100 switching cycles of irradiation by 567 nm light. The absorbance (black lines) was measured at λ_{max} = 568 nm. Top: The complete 100 switching cycles. The first hour of measurement was in the dark and no decomposition was observed. Middle: Expansion of the first 10 and last 10 switching cycles. Bottom: Expansions of the first (left) and last (right) switching cycle with exponential curves (red) fitted to determine the apparent thermal half-life time (table inserts, time in s).

28.8 Fatigue resistance and thermal half-life calculation for 8 in chloroform

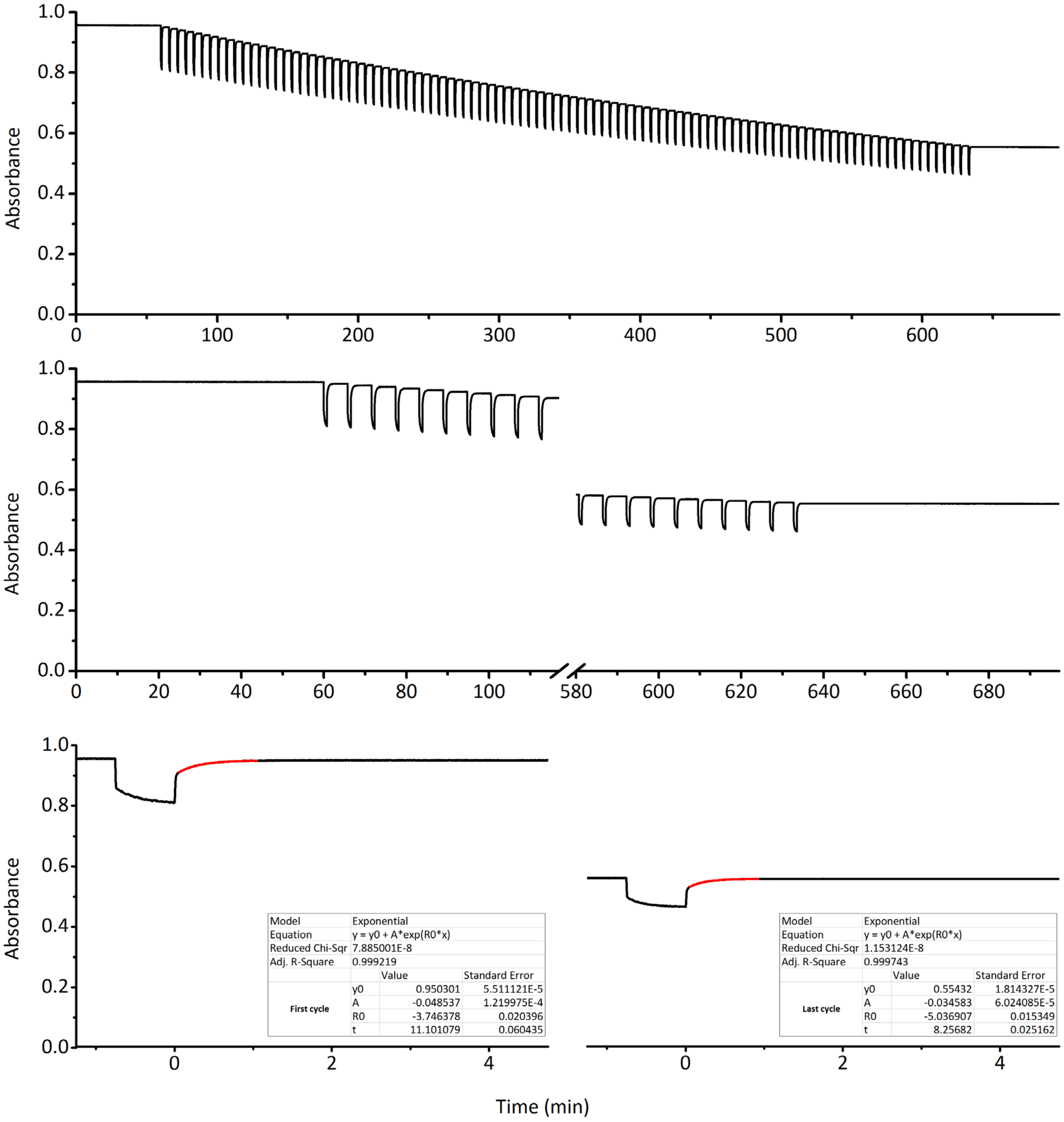

Figure S78. The fatigue resistance of **8** ($CHCl_3$, 298 K) during 100 switching cycles of irradiation by 567 nm light. The absorbance (black lines) was measured at λ_{max} = 569 nm. Top: The complete 100 switching cycles. The first hour of measurement was in the dark and no decomposition was observed. Middle: Expansion of the first 10 and last 10 switching cycles. Bottom: Expansions of the first (left) and last (right) switching cycle with exponential curves (red) fitted to determine the apparent thermal half-life time (table inserts, time in s).

28.9 Fatigue resistance and thermal half-life calculation for 9 in chloroform

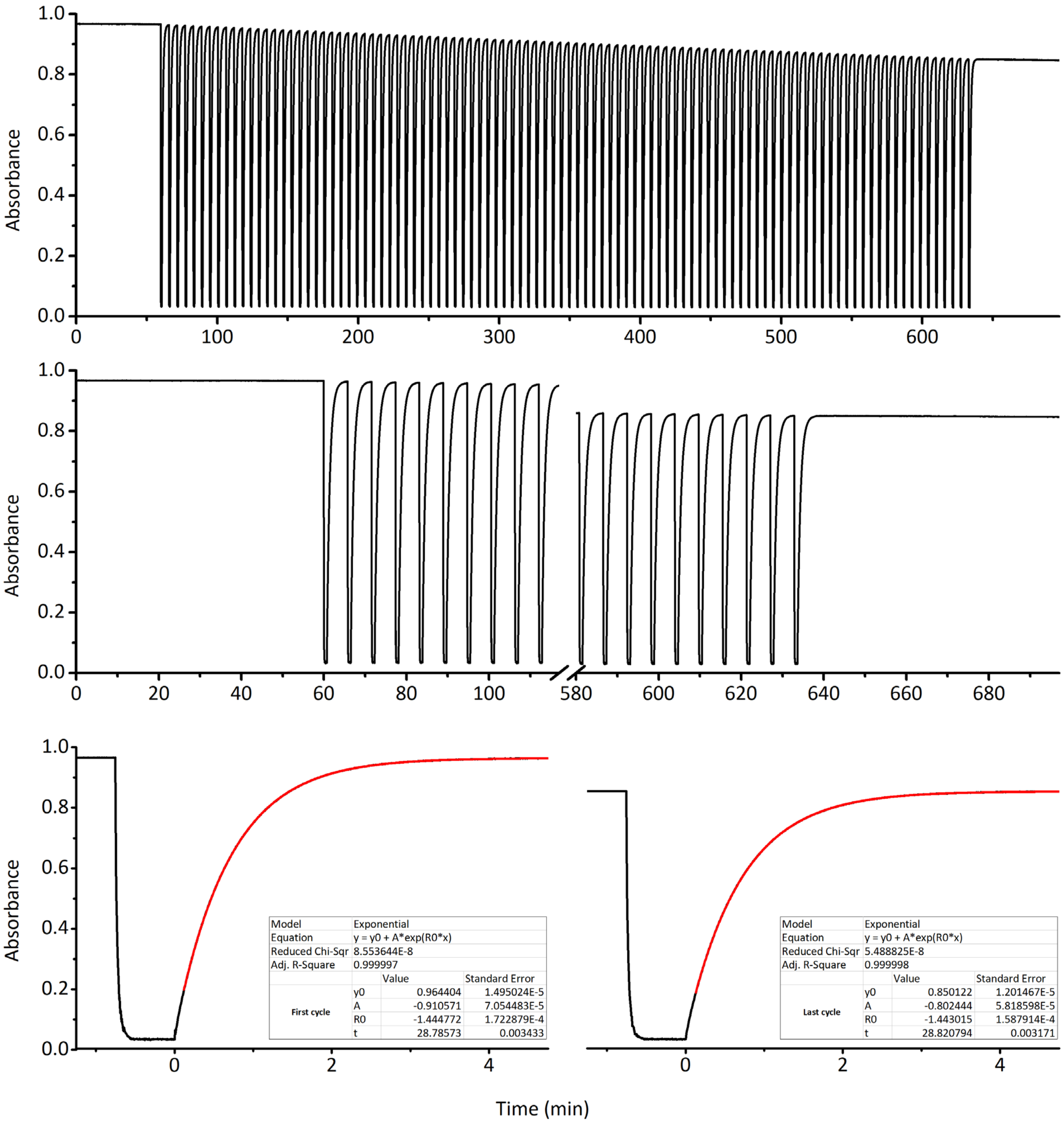

Figure S79. The fatigue resistance of **9** ($CHCl_3$, 298 K) during 100 switching cycles of irradiation by 567 nm light. The absorbance (black lines) was measured at λ_{max} = 569 nm. Top: The complete 100 switching cycles. The first hour of measurement was in the dark and no decomposition was observed. Middle: Expansion of the first 10 and last 10 switching cycles. Bottom: Expansions of the first (left) and last (right) switching cycle with exponential curves (red) fitted to determine the apparent thermal half-life time (table inserts, time in s).

28.10 Fatigue resistance and thermal half-life calculation for **10** in chloroform

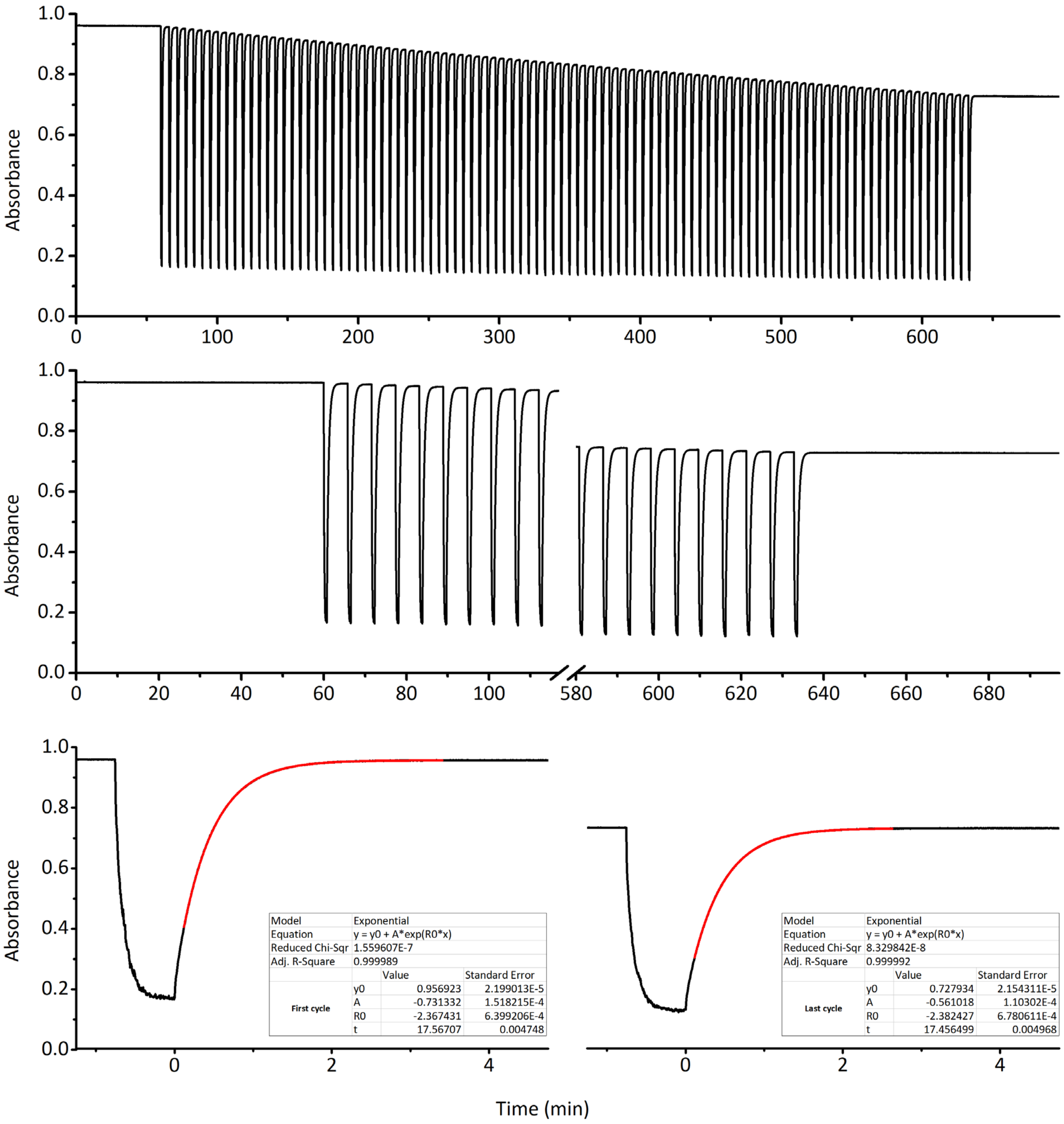

Figure S80. The fatigue resistance of **10** ($CHCl_3$, 298 K) during 100 switching cycles of irradiation by 567 nm light. The absorbance (black lines) was measured at λ_{max} = 570 nm. Top: The complete 100 switching cycles. The first hour of measurement was in the dark and no decomposition was observed. Middle: Expansion of the first 10 and last 10 switching cycles. Bottom: Expansions of the first (left) and last (right) switching cycle with exponential curves (red) fitted to determine the apparent thermal half-life time (table inserts, time in s).

28.11 Fatigue resistance and thermal half-life calculation for 11 in chloroform

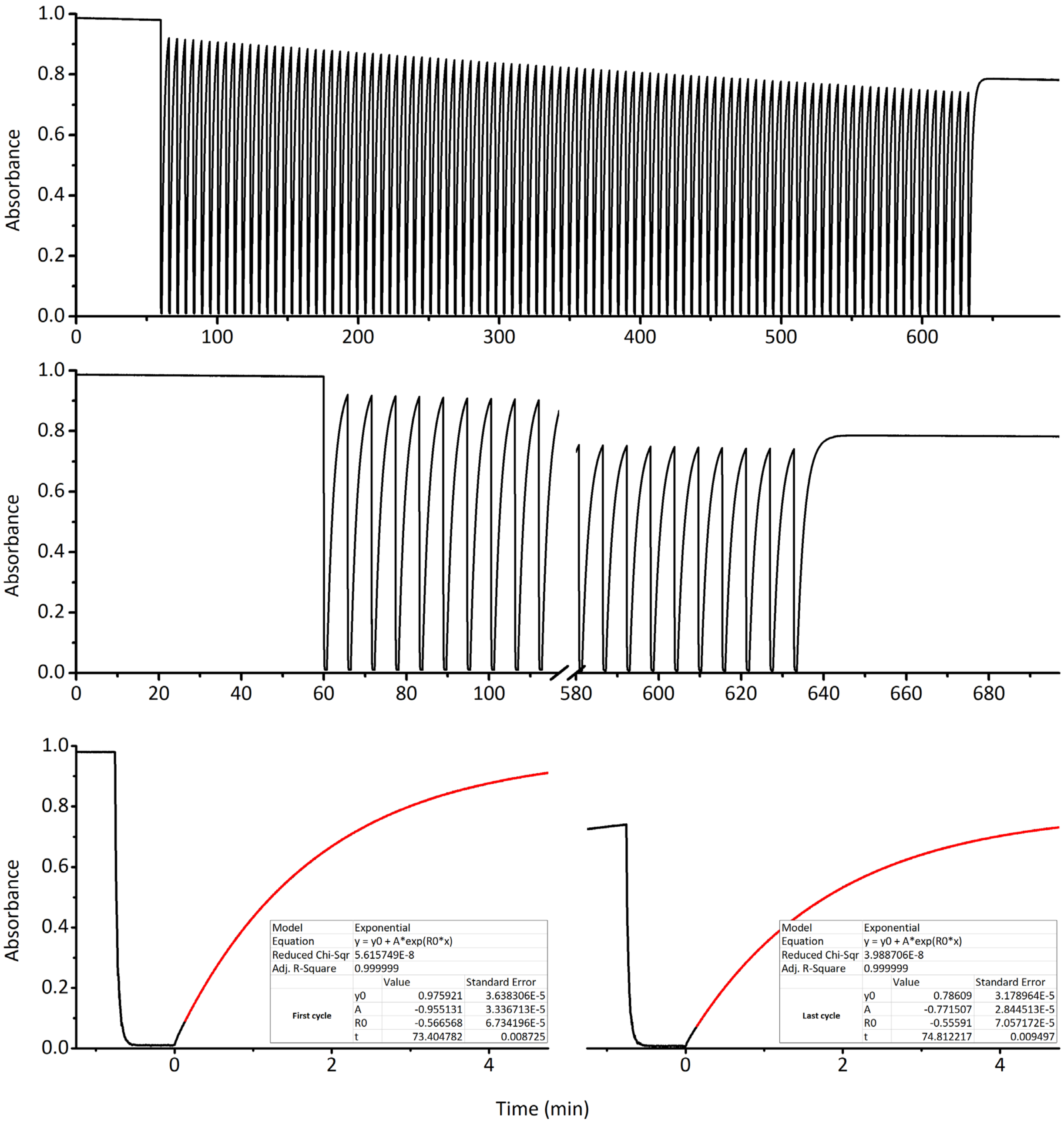

Figure S81. The fatigue resistance of **11** ($CHCl_3$, 298 K) during 100 switching cycles of irradiation by 567 nm light. The absorbance (black lines) was measured at λ_{max} = 570 nm. Top: The complete 100 switching cycles. The first hour of measurement was in the dark and no decomposition was observed. Middle: Expansion of the first 10 and last 10 switching cycles. Bottom: Expansions of the first (left) and last (right) switching cycle with exponential curves (red) fitted to determine the apparent thermal half-life time (table inserts, time in s).

28.12 Fatigue resistance and thermal half-life calculation for 12 in chloroform

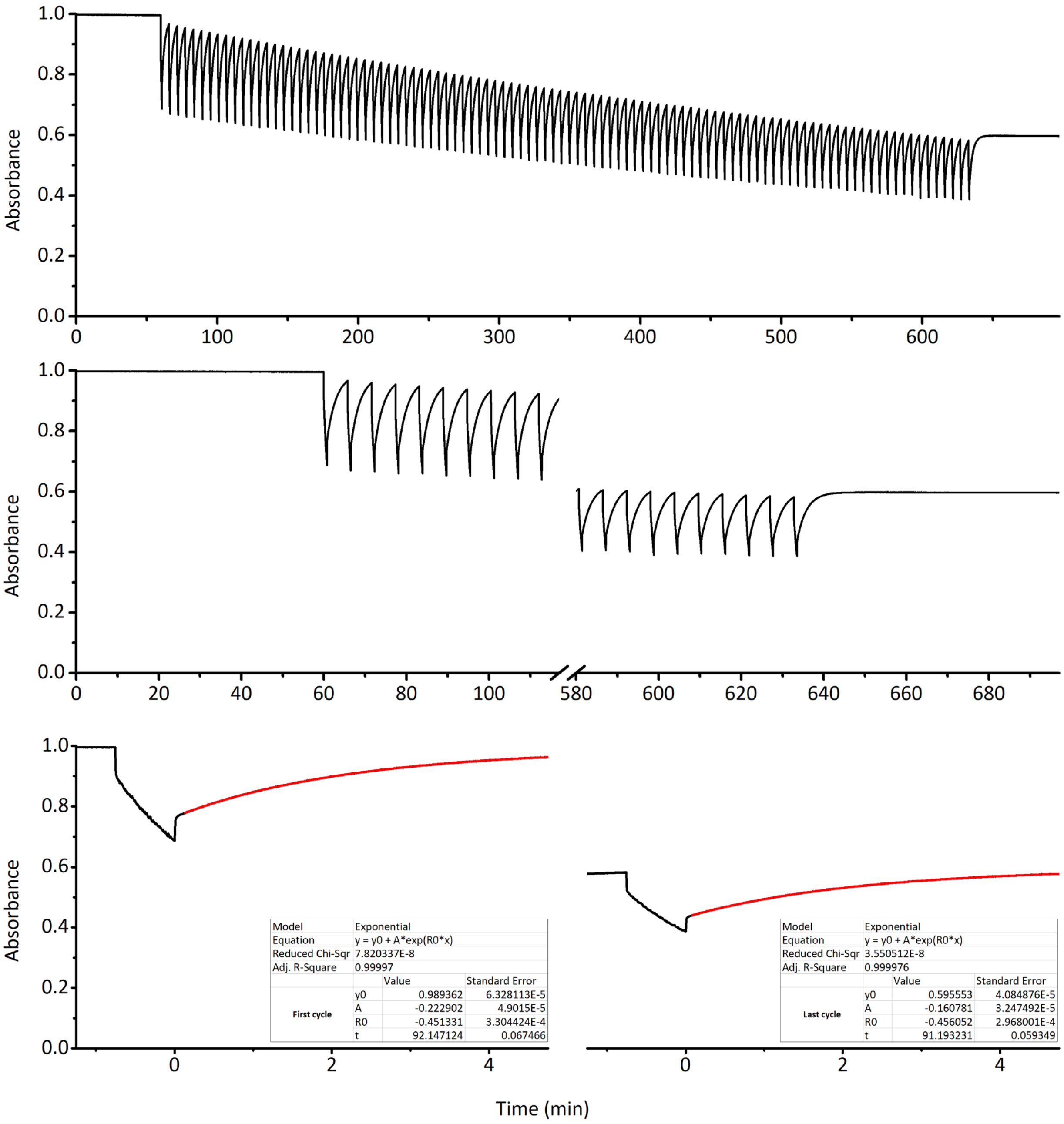

Figure S82. The fatigue resistance of **12** ($CHCl_3$, 298 K) during 100 switching cycles of irradiation by 567 nm light. The absorbance (black lines) was measured at λ_{max} = 570 nm. Top: The complete 100 switching cycles. The first hour of measurement was in the dark and no decomposition was observed. Middle: Expansion of the first 10 and last 10 switching cycles. Bottom: Expansions of the first (left) and last (right) switching cycle with exponential curves (red) fitted to determine the apparent thermal half-life time (table inserts, time in s).

28.13 Fatigue resistance and thermal half-life calculation for 13 in chloroform

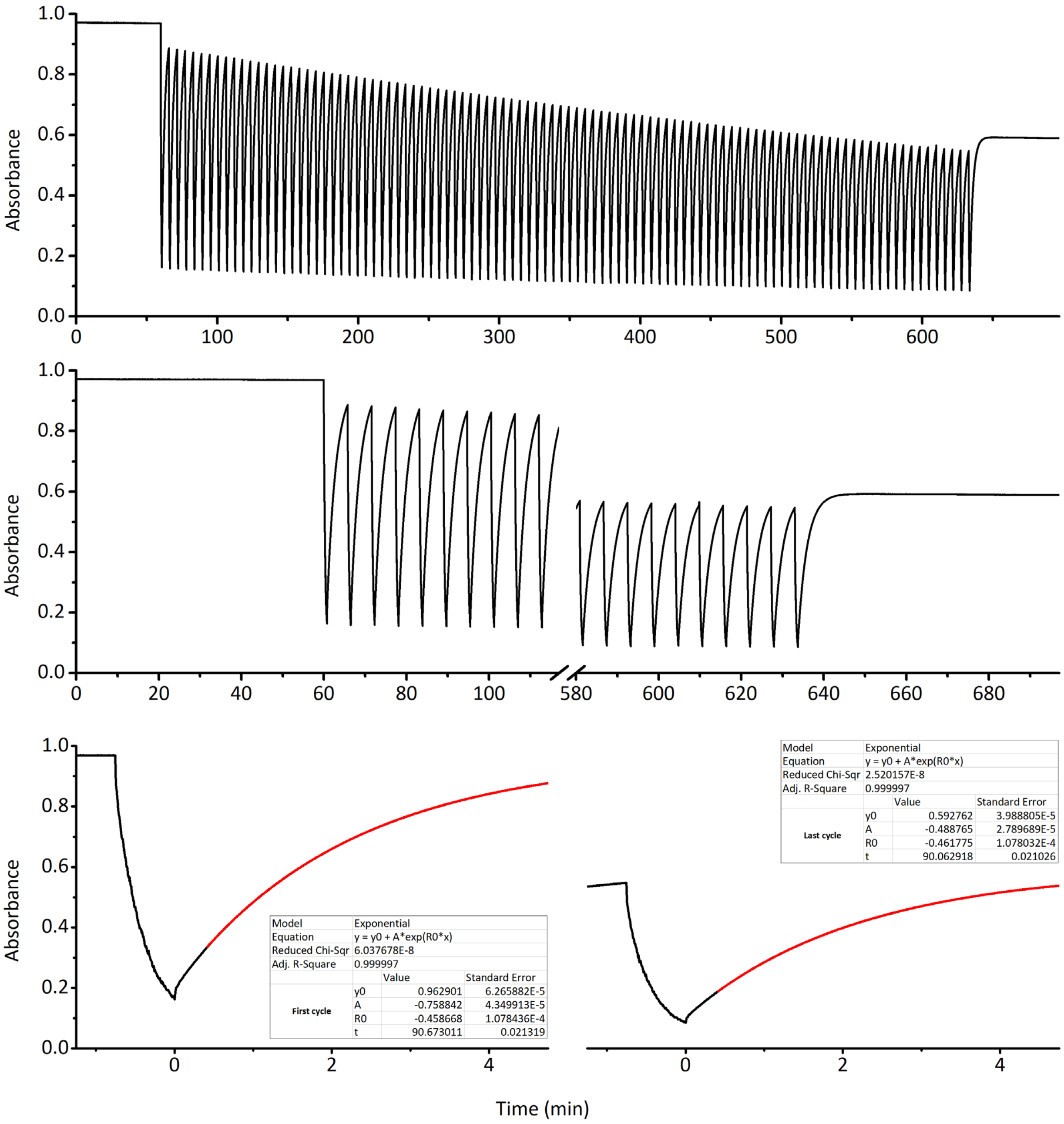

Figure S83. The fatigue resistance of **13** ($CHCl_3$, 298 K) during 100 switching cycles of irradiation by 567 nm light. The absorbance (black lines) was measured at λ_{max} = 566 nm. Top: The complete 100 switching cycles. The first hour of measurement was in the dark and no decomposition was observed. Middle: Expansion of the first 10 and last 10 switching cycles. Bottom: Expansions of the first (left) and last (right) switching cycle with exponential curves (red) fitted to determine the apparent thermal half-life time (table inserts, time in s).

28.14 Fatigue resistance and thermal half-life calculation for 14 in chloroform

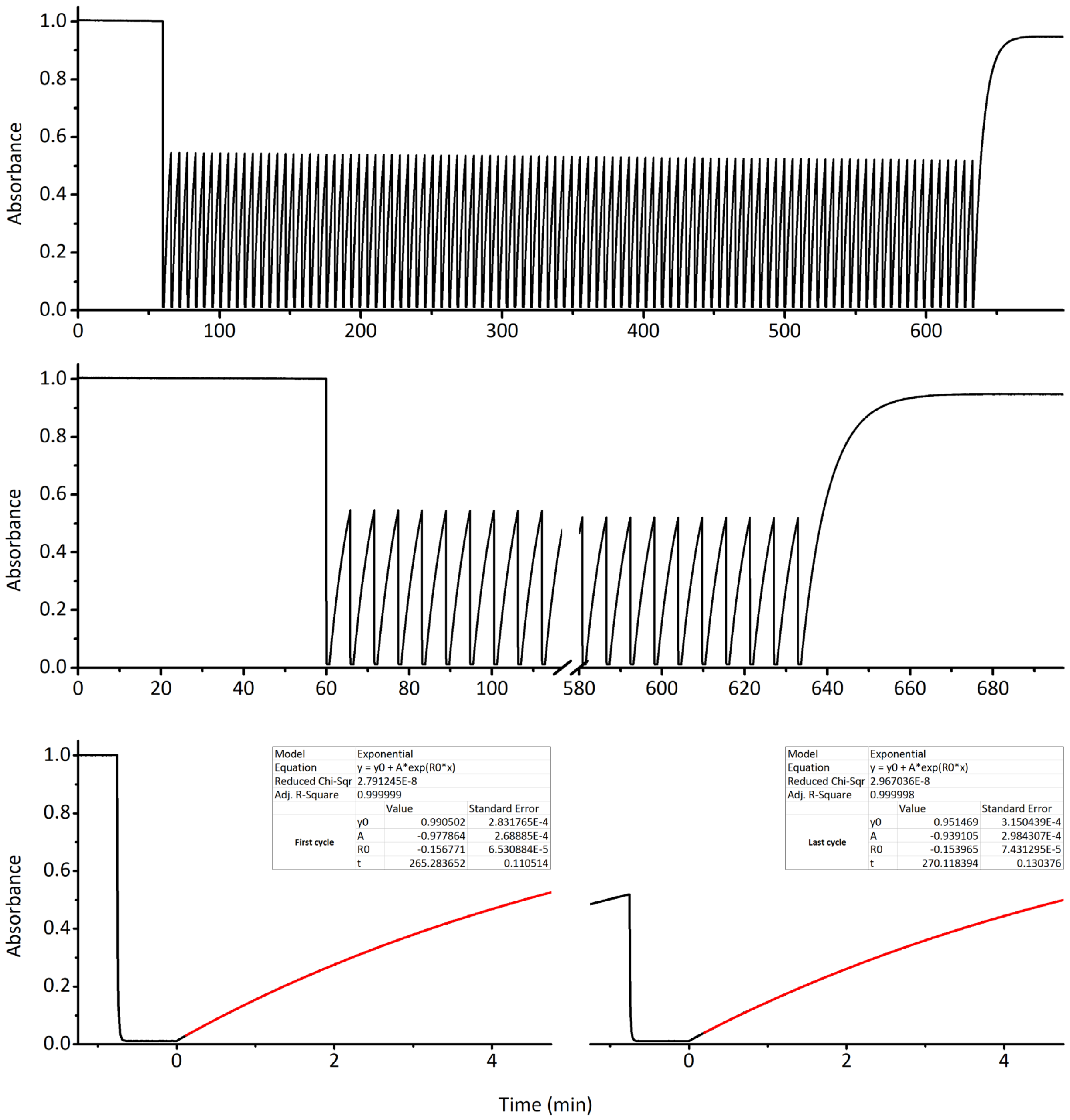

Figure S84. The fatigue resistance of **14** ($CHCl_3$, 298 K) during 100 switching cycles of irradiation by 567 nm light. The absorbance (black lines) was measured at λ_{max} = 588 nm. Top: The complete 100 switching cycles. The first hour of measurement was in the dark and no decomposition was observed. Middle: Expansion of the first 10 and last 10 switching cycles. Bottom: Expansions of the first (left) and last (right) switching cycle with exponential curves (red) fitted to determine the apparent thermal half-life time (table inserts, time in s).

28.15 Fatigue resistance of 4 using optimized conditions

Sections 28.1 to 28.14 show the fatigue resistance of compounds **1-14** under identical conditions (100 cycles of 45 s irradiation followed by 5 min thermal reversion). These experiments show that decomposition mainly takes place during irradiation. By shortening the period of irradiation the photodecomposition can be minimalized, extending the fatigue resistance of the switches. The example below shows DASA **4** undergoing 1000 cycles of irradiation and thermal recovery.

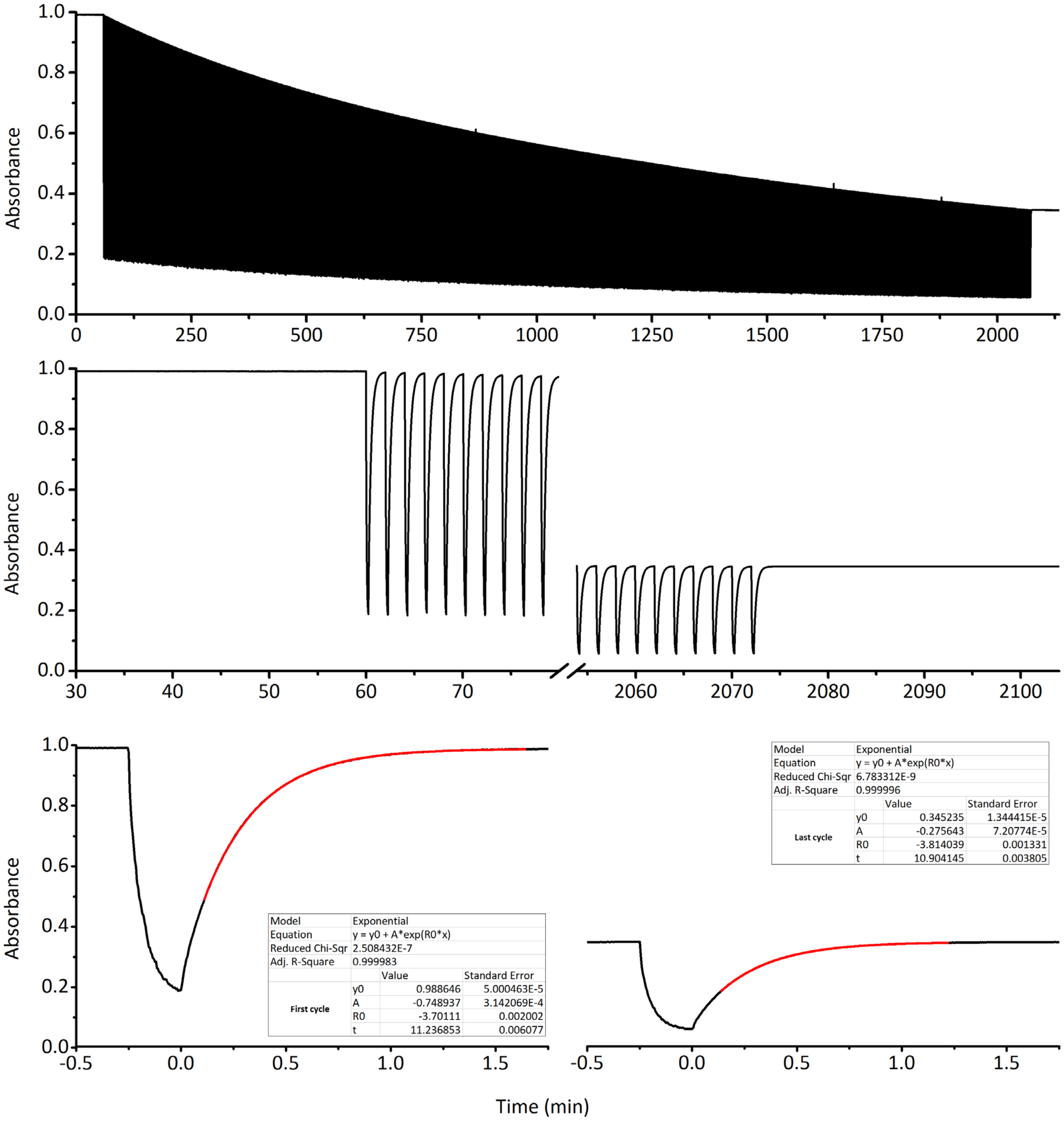

Figure S85. The fatigue resistance of **4** ($CHCl_3$, 298 K) during 1000 switching cycles. The sample was irradiated for 15 s by 567 nm light, followed by 105 s in the dark to thermally equilibrate. The absorbance (black lines) was measured at λ_{max} = 568 nm. Top: The complete 1000 switching cycles. The first hour of measurement was in the dark and no decomposition was observed. Middle: Expansion of the first 10 and last 10 switching cycles. Bottom: Expansions of the first (left) and last (right) switching cycle with exponential curves (red) fitted to determine the apparent thermal half-life time (table inserts, time in s).

28.16 Oxygen sensitivity in chloroform

All fatigue measurements were performed in the presence of oxygen, which has been shown to reduce the fatigue resistance in other photoswitches. To investigate if DASAs are sensitive to oxygen as well, a sample of **2** in $CHCl_3$ was deoxygenated by sparging with argon for 30 minutes. The fatigue resistance was compared with a sample prepared in the same manner but measured in the presence of oxygen.

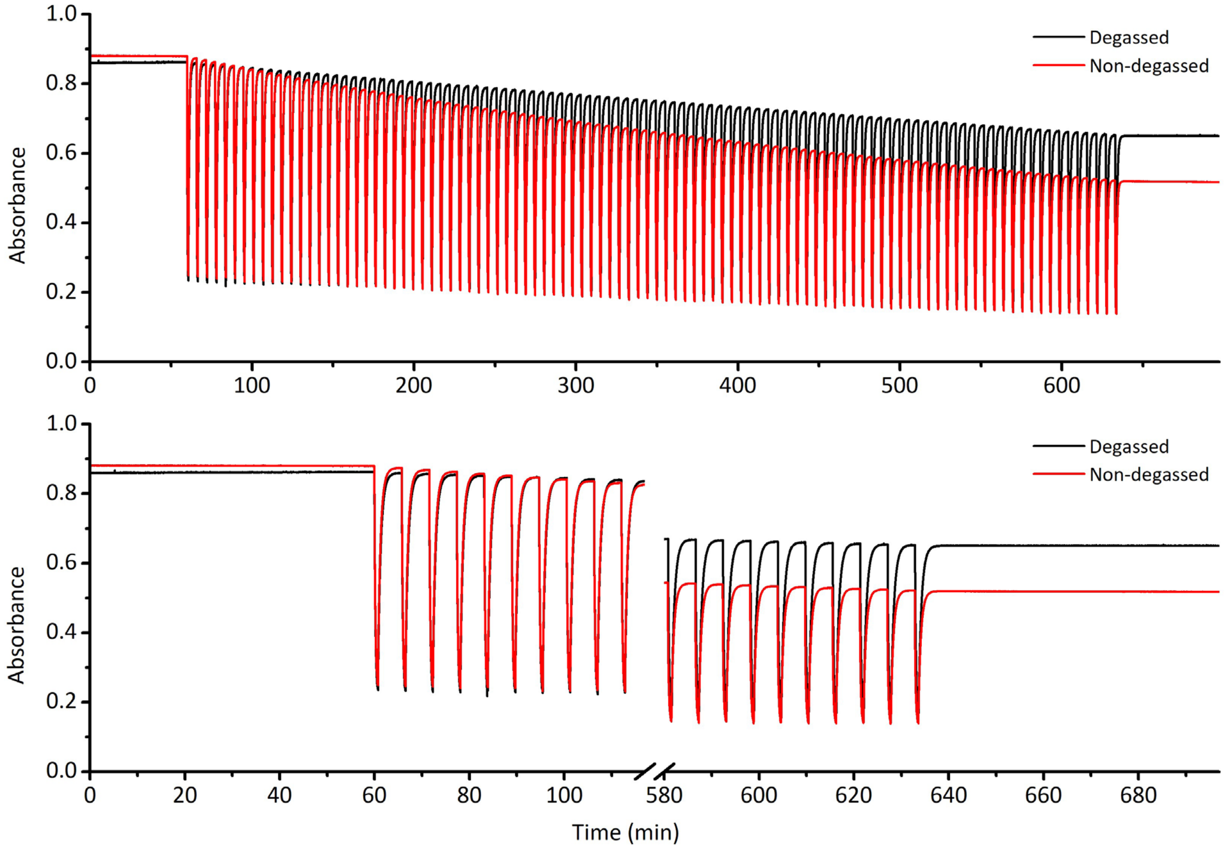

Figure S86. Fatigue measurements on **2** in $CHCl_3$, measured at λ_{max}= 565 nm under atmospheric conditions (red) and under an argon atmosphere (black). After 100 cycles of 45 s irradiation (567 nm LED) followed by 5 min equilibration in the dark, the degassed sample decomposed by 25%, while the non-degassed sample decomposed by 41%.

29 Modelled kinetic data

29.1 Modelled kinetic data for 1 in chloroform

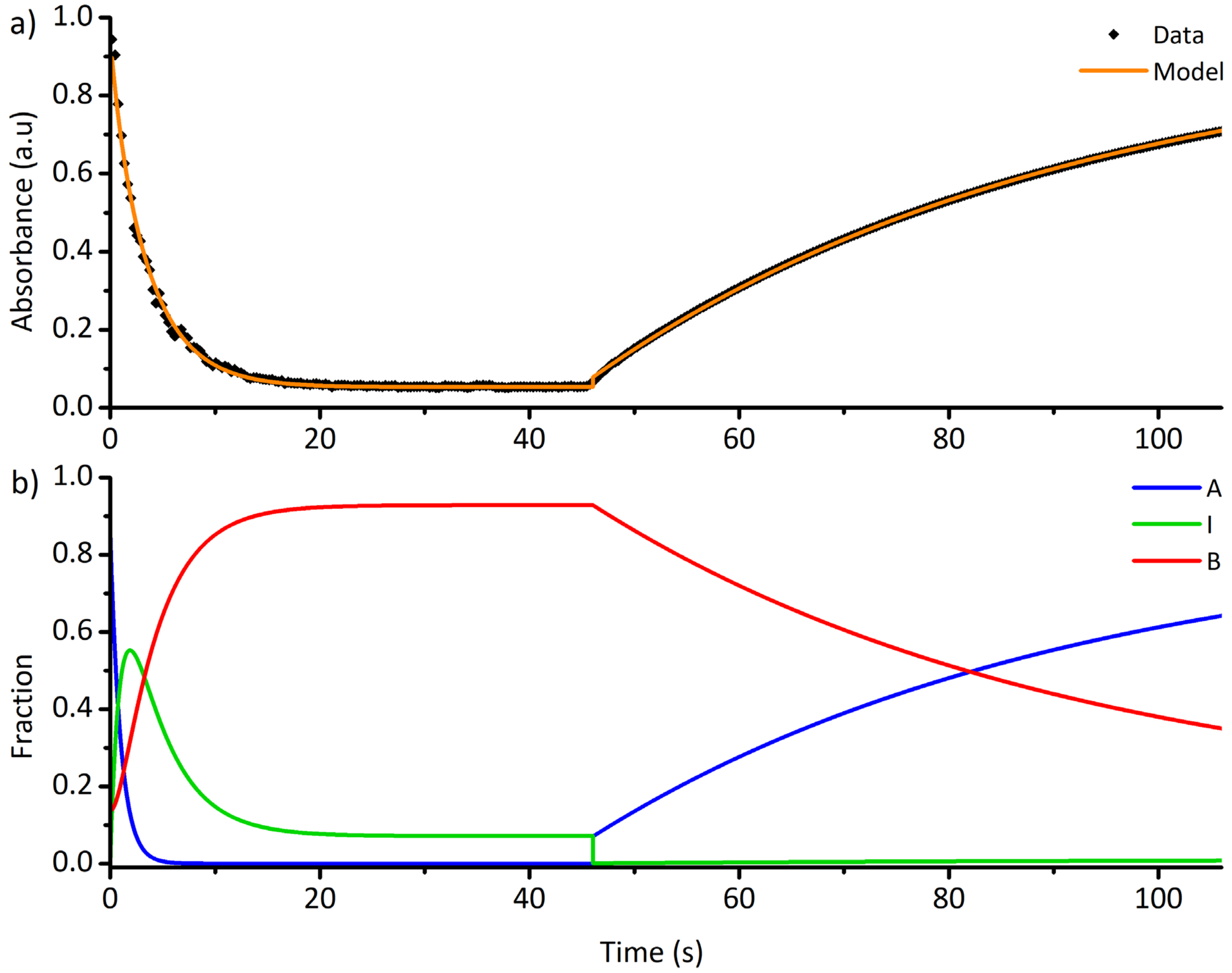

Figure S87. a) Absorbance (565 nm) measured during one photoswitching cycle on **1** in $CHCl_3$ and the predicted absorbance from the kinetic model. b) The predicted fractions A, I and B from the kinetic model.

29.2 Modelled kinetic data for 2 in chloroform

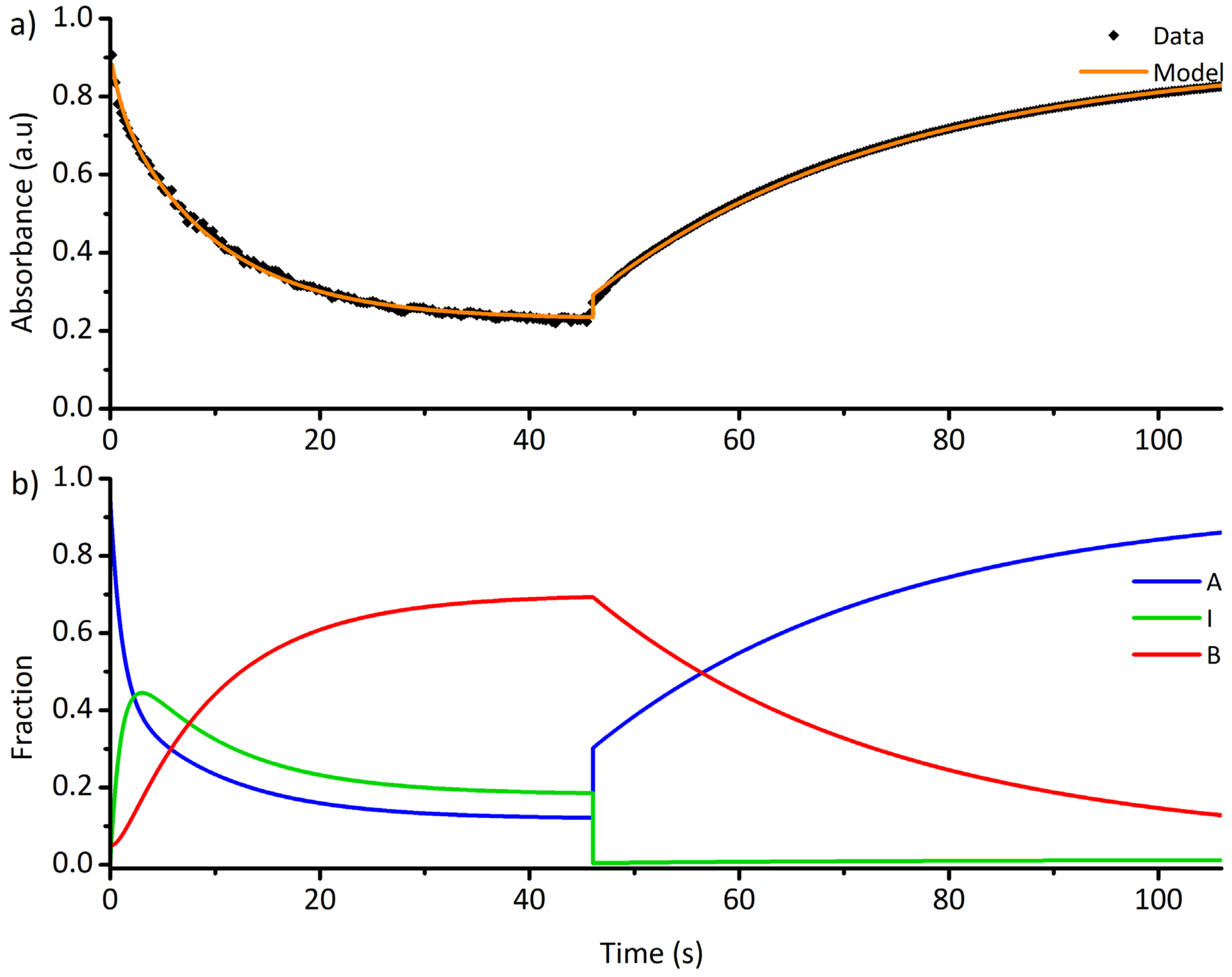

Figure S88. a) Absorbance (565 nm) measured during one photoswitching cycle on **2** in $CHCl_3$ and the predicted absorbance from the kinetic model. b) The predicted fractions A, I and B from the kinetic model.

29.3 Modelled kinetic data for 3 in chloroform

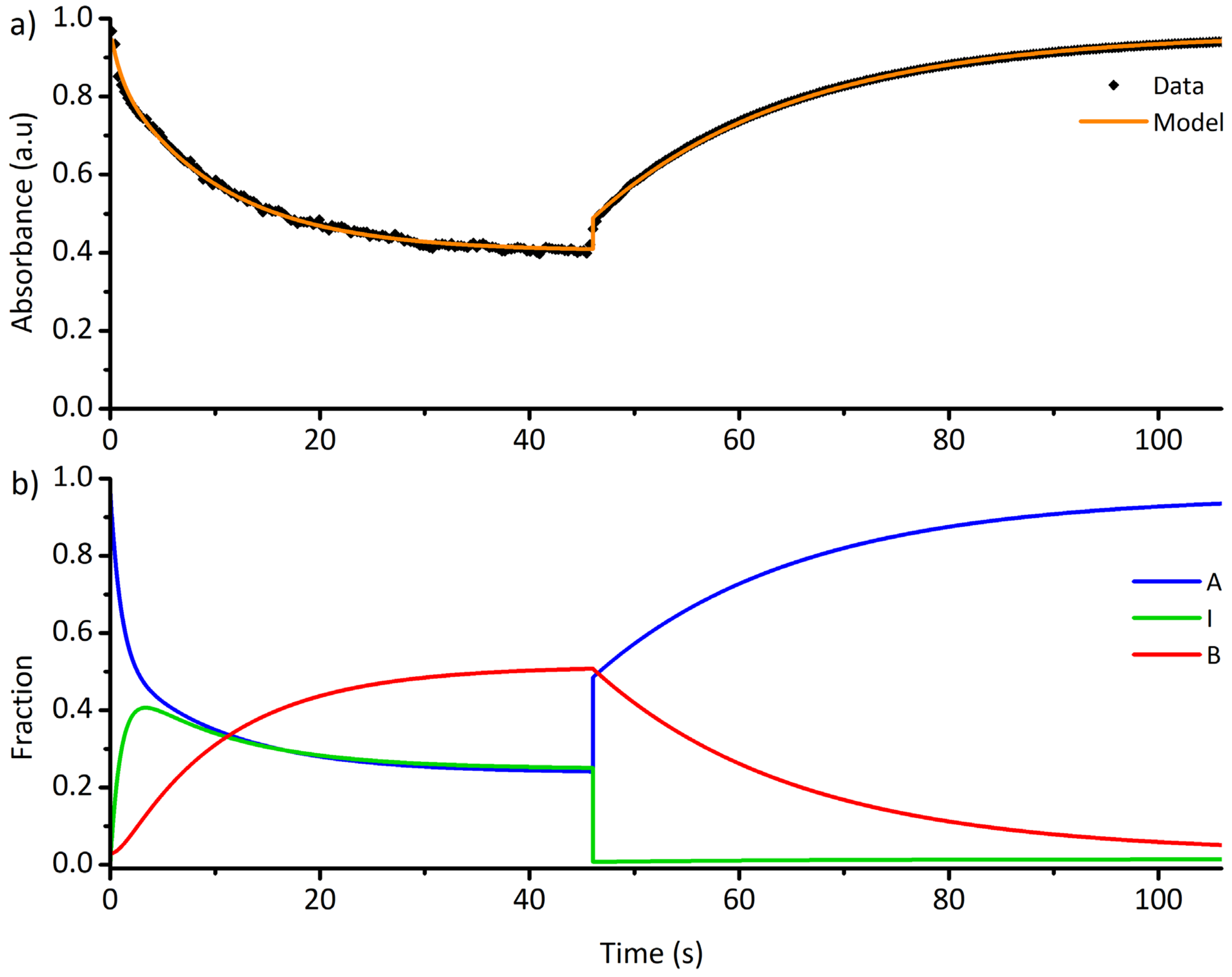

Figure S89. a) Absorbance (566 nm) measured during one photoswitching cycle on **3** in $CHCl_3$ and the predicted absorbance from the kinetic model. b) The predicted fractions A, I and B from the kinetic model.

29.4 Modelled kinetic data for 4 in chloroform

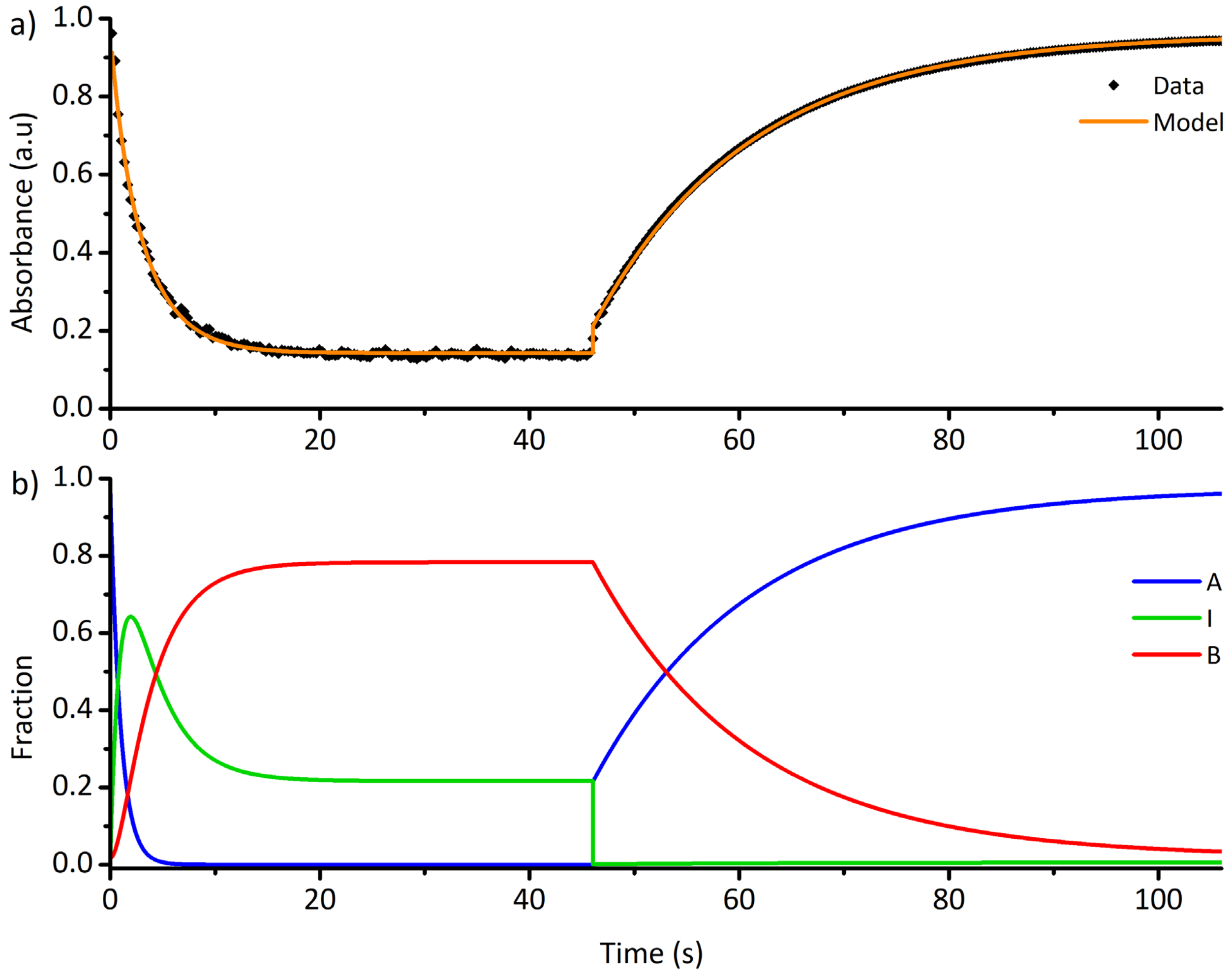

Figure S90. a) Absorbance (568 nm) measured during one photoswitching cycle on **4** in $CHCl_3$ and the predicted absorbance from the kinetic model. b) The predicted fractions A, I and B from the kinetic model.

29.5 Modelled kinetic data for 5 in chloroform

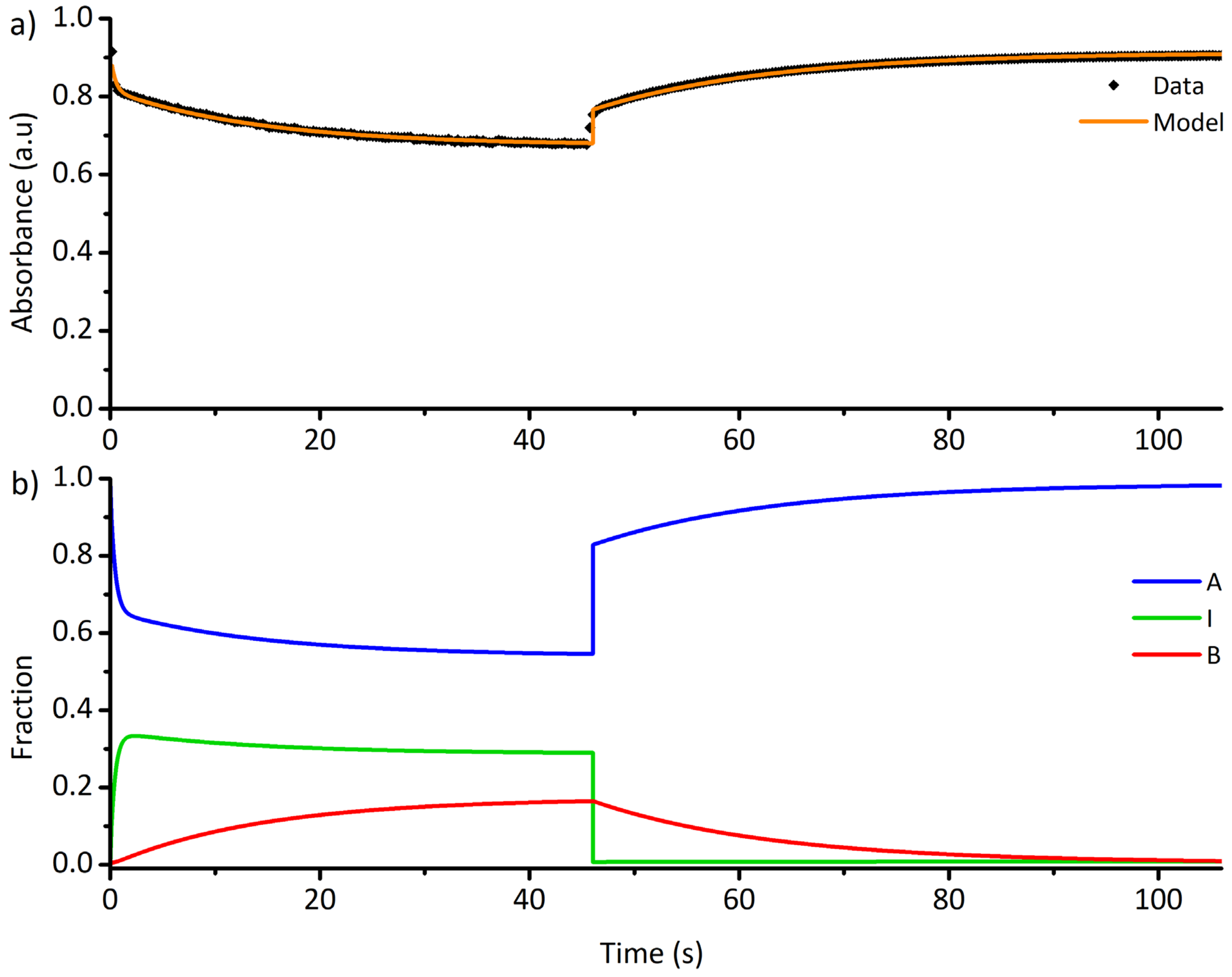

Figure S91. a) Absorbance (565 nm) measured during one photoswitching cycle on **5** in $CHCl_3$ and the predicted absorbance from the kinetic model. b) The predicted fractions A, I and B from the kinetic model.

29.6 Modelled kinetic data for 6 in chloroform

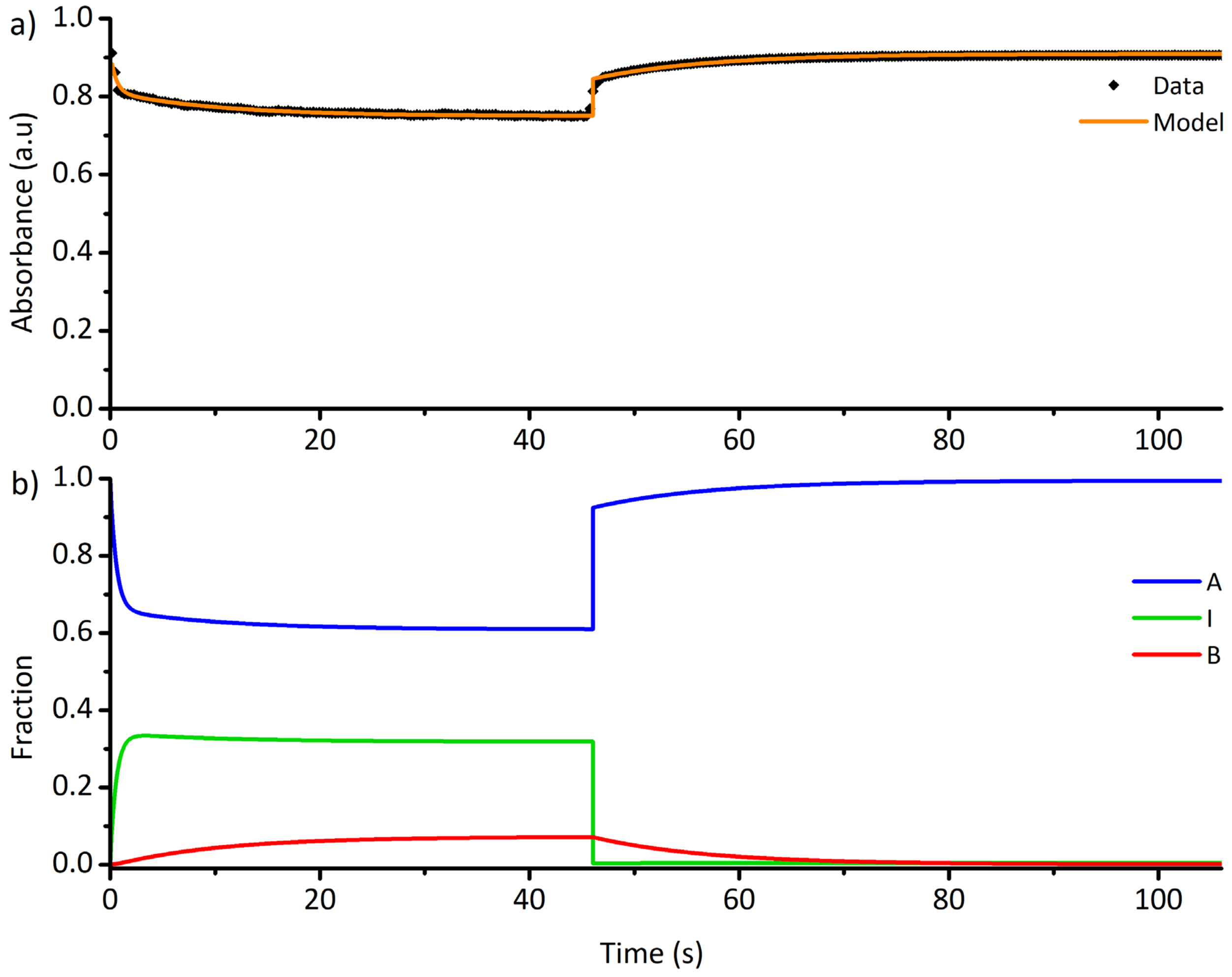

Figure S92. a) Absorbance (567 nm) measured during one photoswitching cycle on **6** in $CHCl_3$ and the predicted absorbance from the kinetic model. b) The predicted fractions A, I and B from the kinetic model.

29.7 Modelled kinetic data for 7 in chloroform

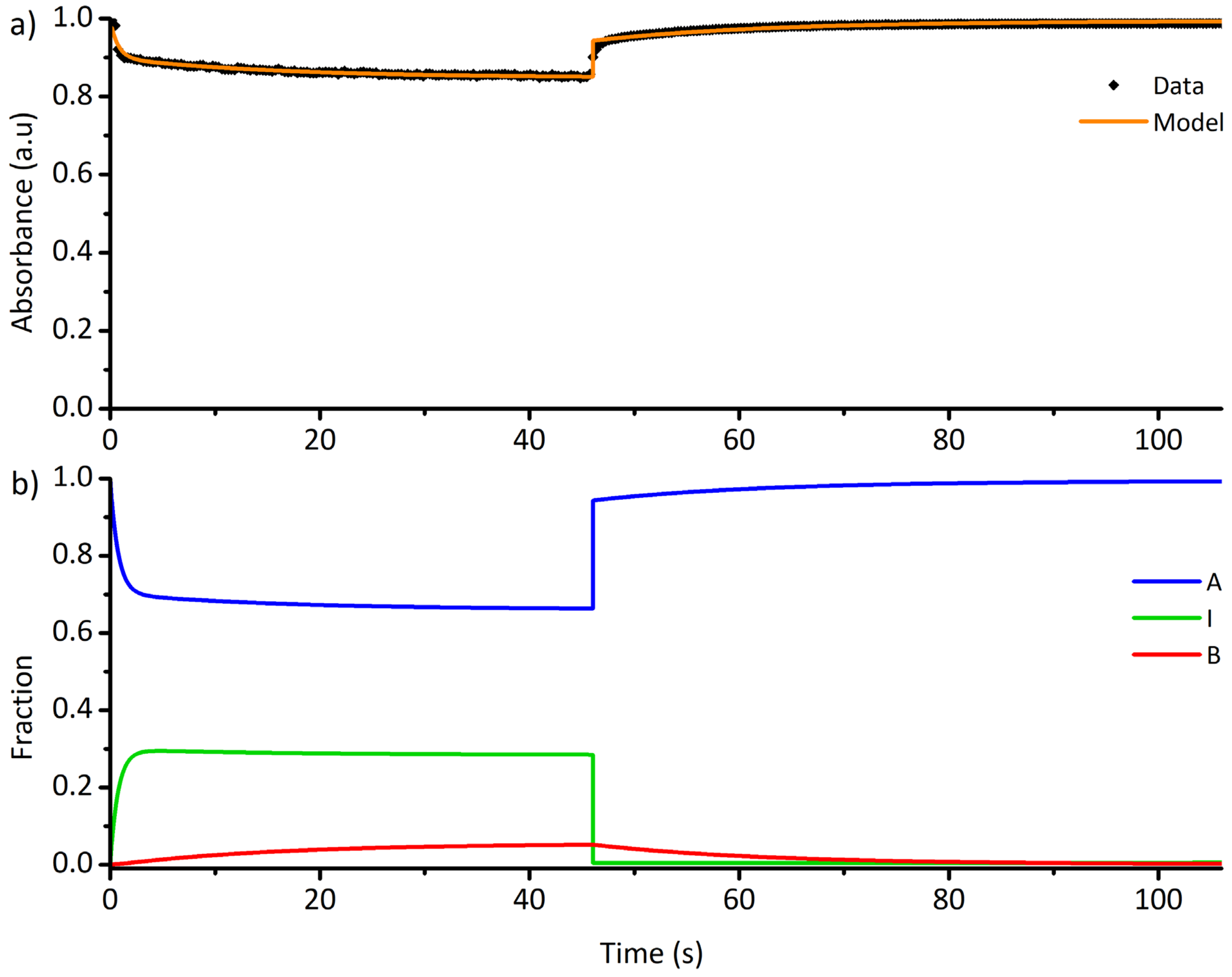

Figure S93. a) Absorbance (568 nm) measured during one photoswitching cycle on **7** in $CHCl_3$ and the predicted absorbance from the kinetic model. b) The predicted fractions A, I and B from the kinetic model.

29.8 Modelled kinetic data for 8 in chloroform

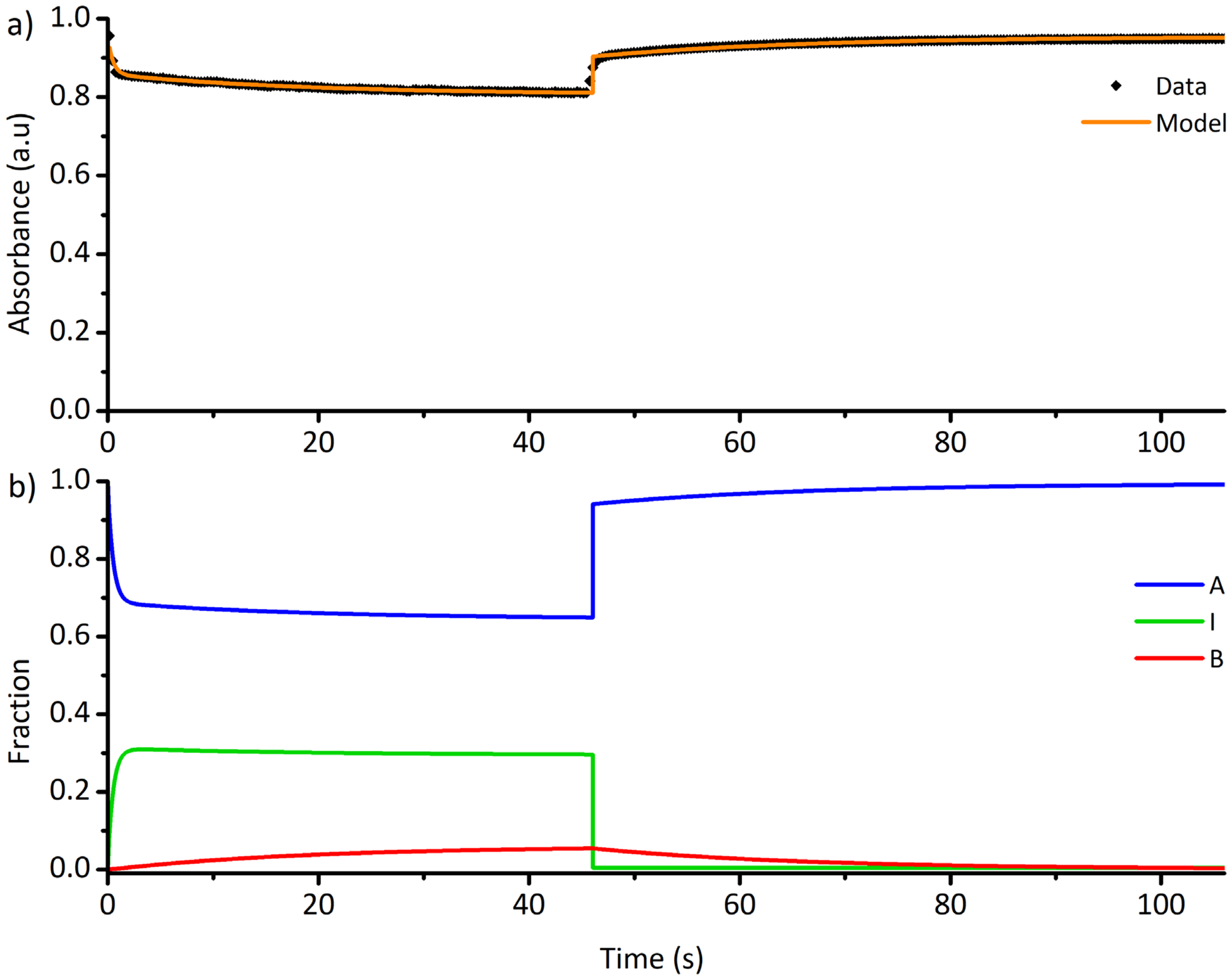

Figure S94. a) Absorbance (569 nm) measured during one photoswitching cycle on **8** in $CHCl_3$ and the predicted absorbance from the kinetic model. b) The predicted fractions A, I and B from the kinetic model.

29.9 Modelled kinetic data for 9 in chloroform

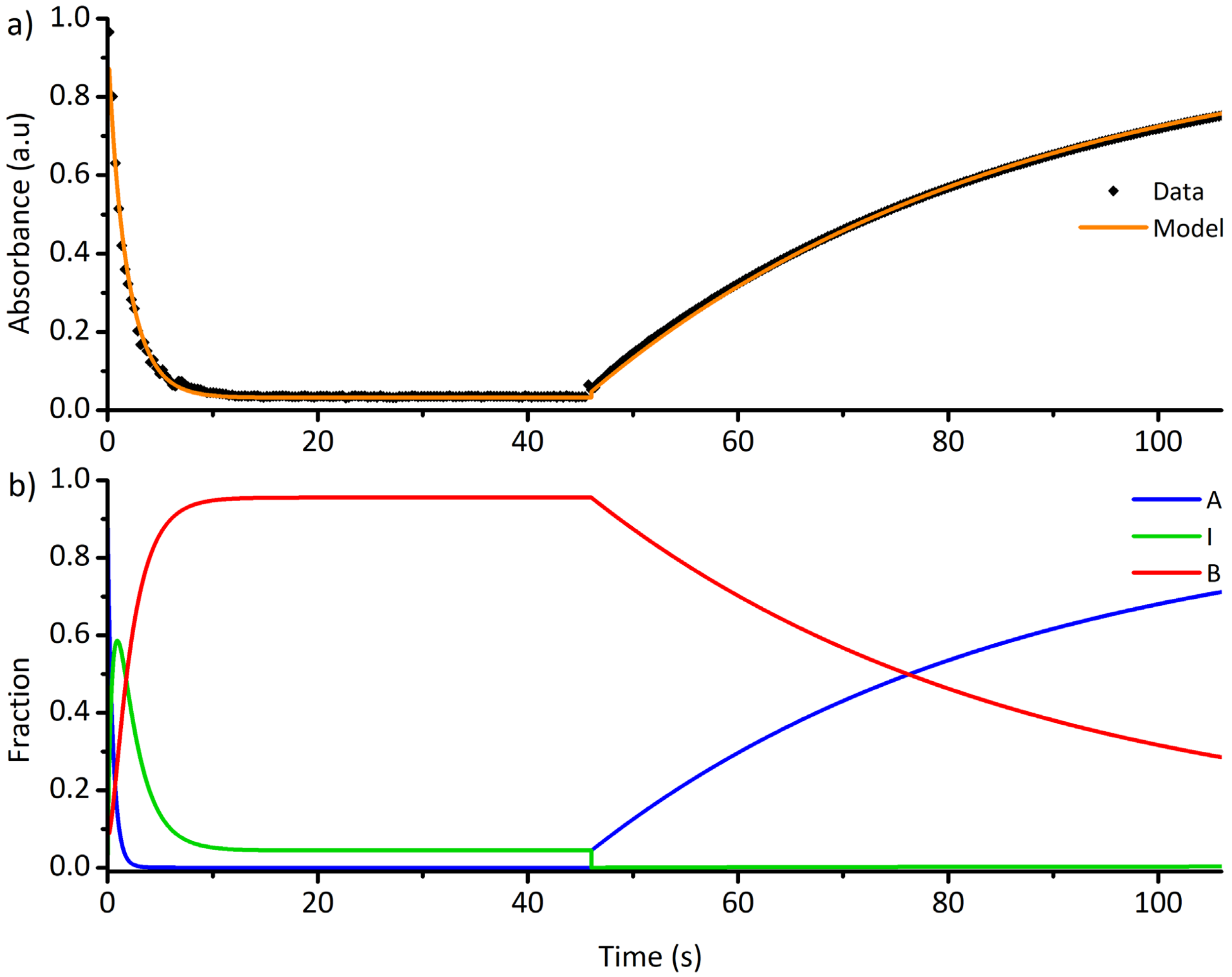

Figure S95. a) Absorbance (569 nm) measured during one photoswitching cycle on **9** in $CHCl_3$ and the predicted absorbance from the kinetic model. b) The predicted fractions A, I and B from the kinetic model.

29.10 Modelled kinetic data for 10 in chloroform

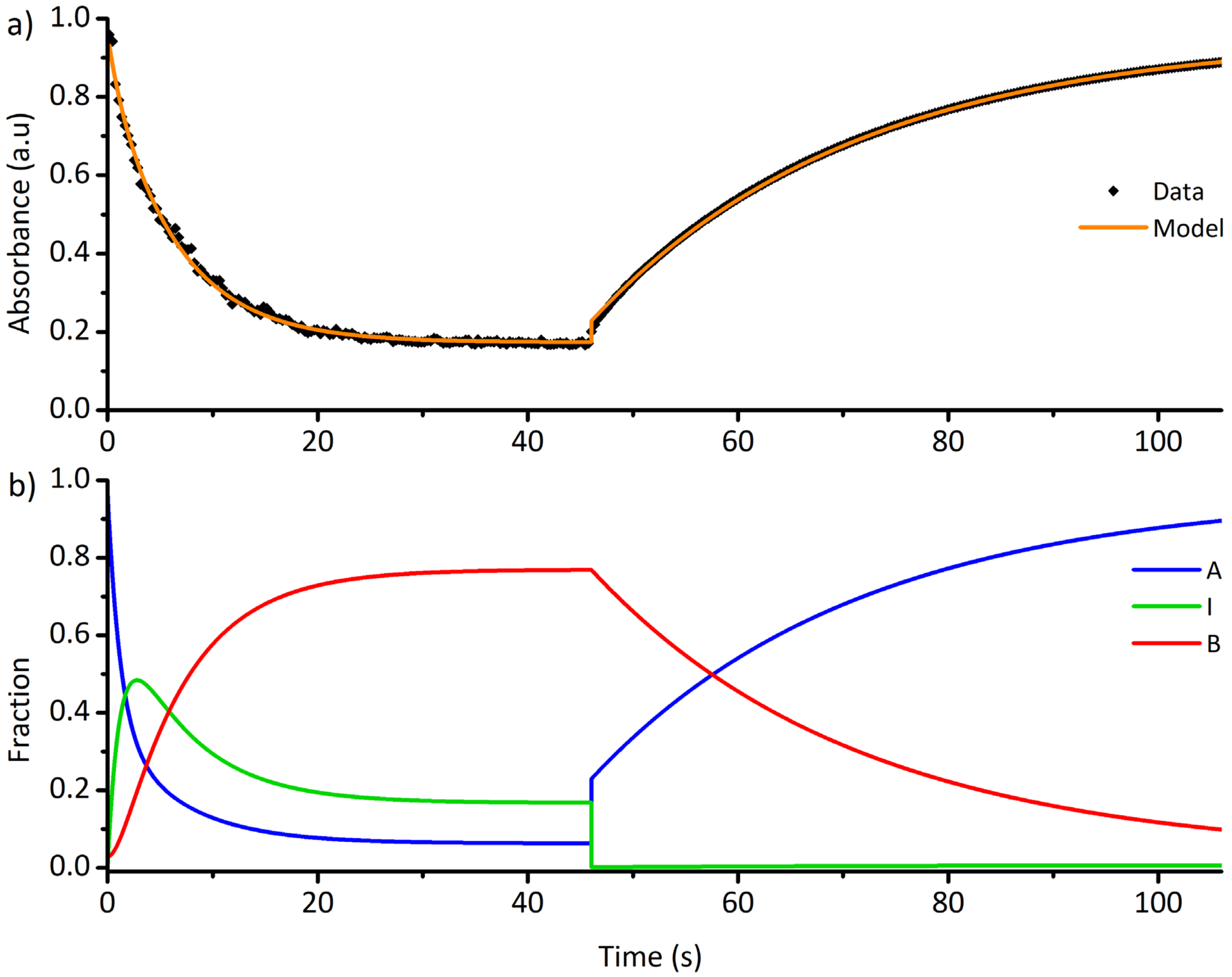

Figure S96. a) Absorbance (570 nm) measured during one photoswitching cycle on **10** in $CHCl_3$ and the predicted absorbance from the kinetic model. b) The predicted fractions A, I and B from the kinetic model.

29.11 Modelled kinetic data for 11 in chloroform

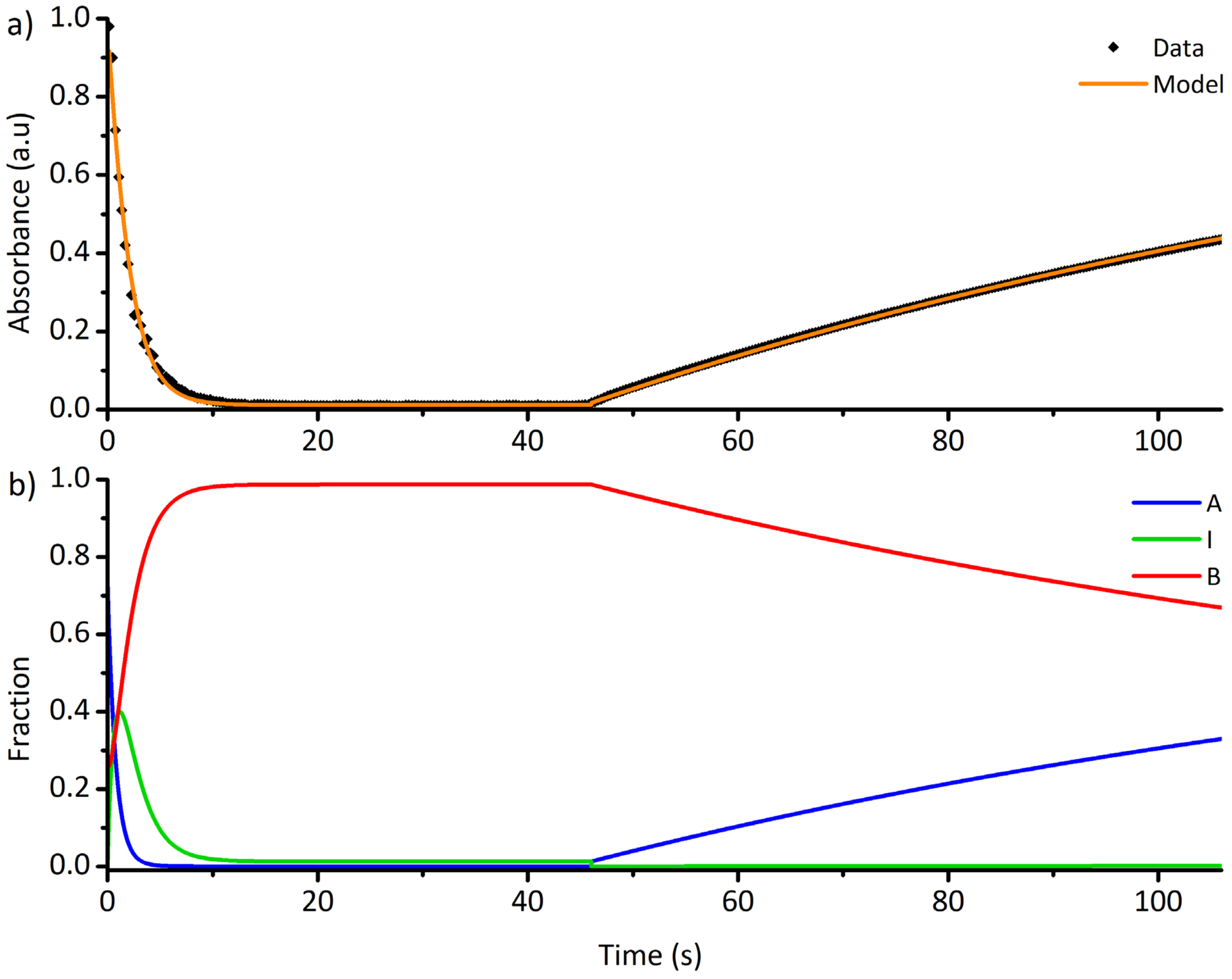

Figure S97. a) Absorbance (570 nm) measured during one photoswitching cycle on **11** in $CHCl_3$ and the predicted absorbance from the kinetic model. b) The predicted fractions A, I and B from the kinetic model.

29.12 Modelled kinetic data for 12 in chloroform

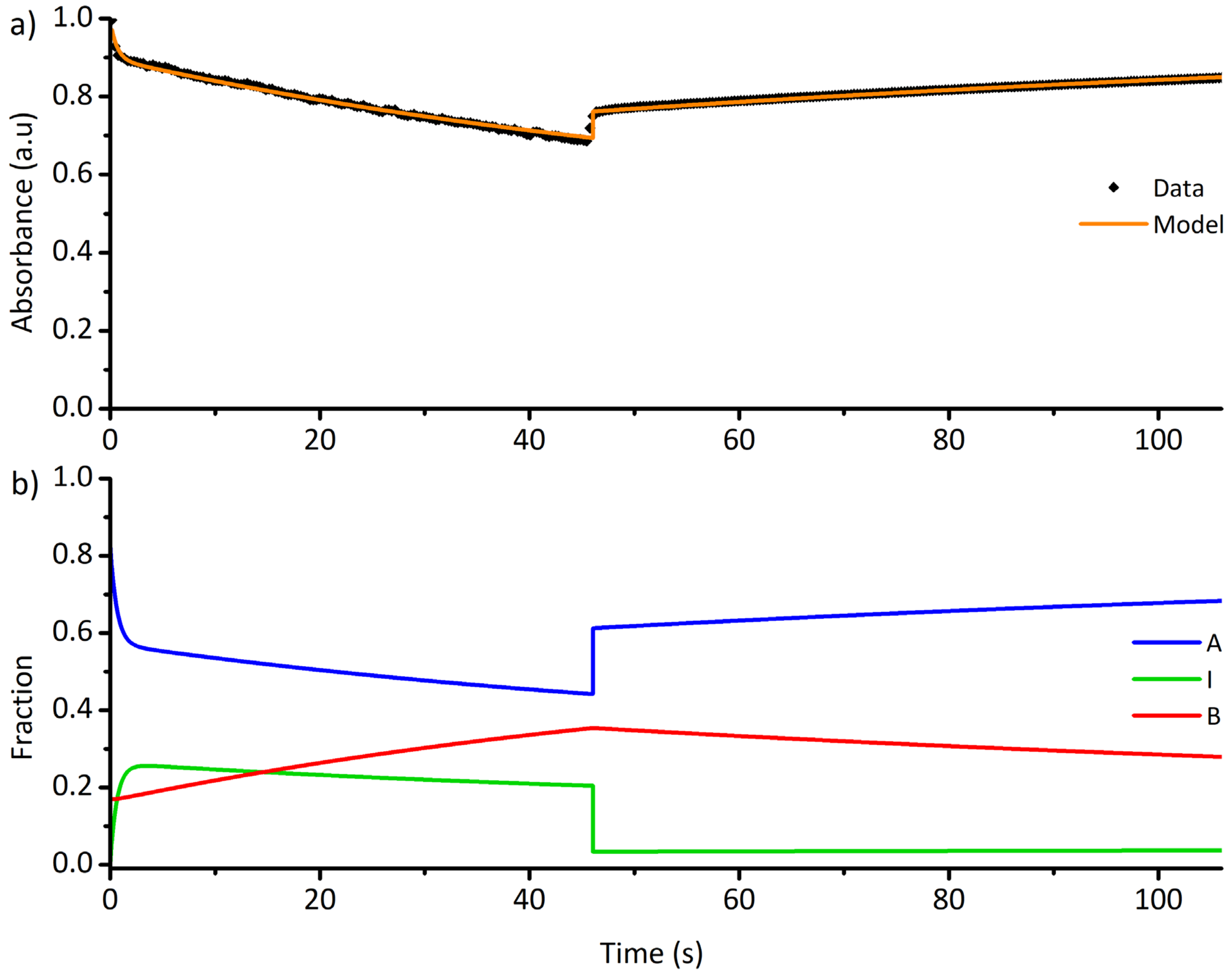

Figure S98. a) Absorbance (570 nm) measured during one photoswitching cycle on **12** in $CHCl_3$ and the predicted absorbance from the kinetic model. b) The predicted fractions A, I and B from the kinetic model.

29.13 Modelled kinetic data for 13 in chloroform

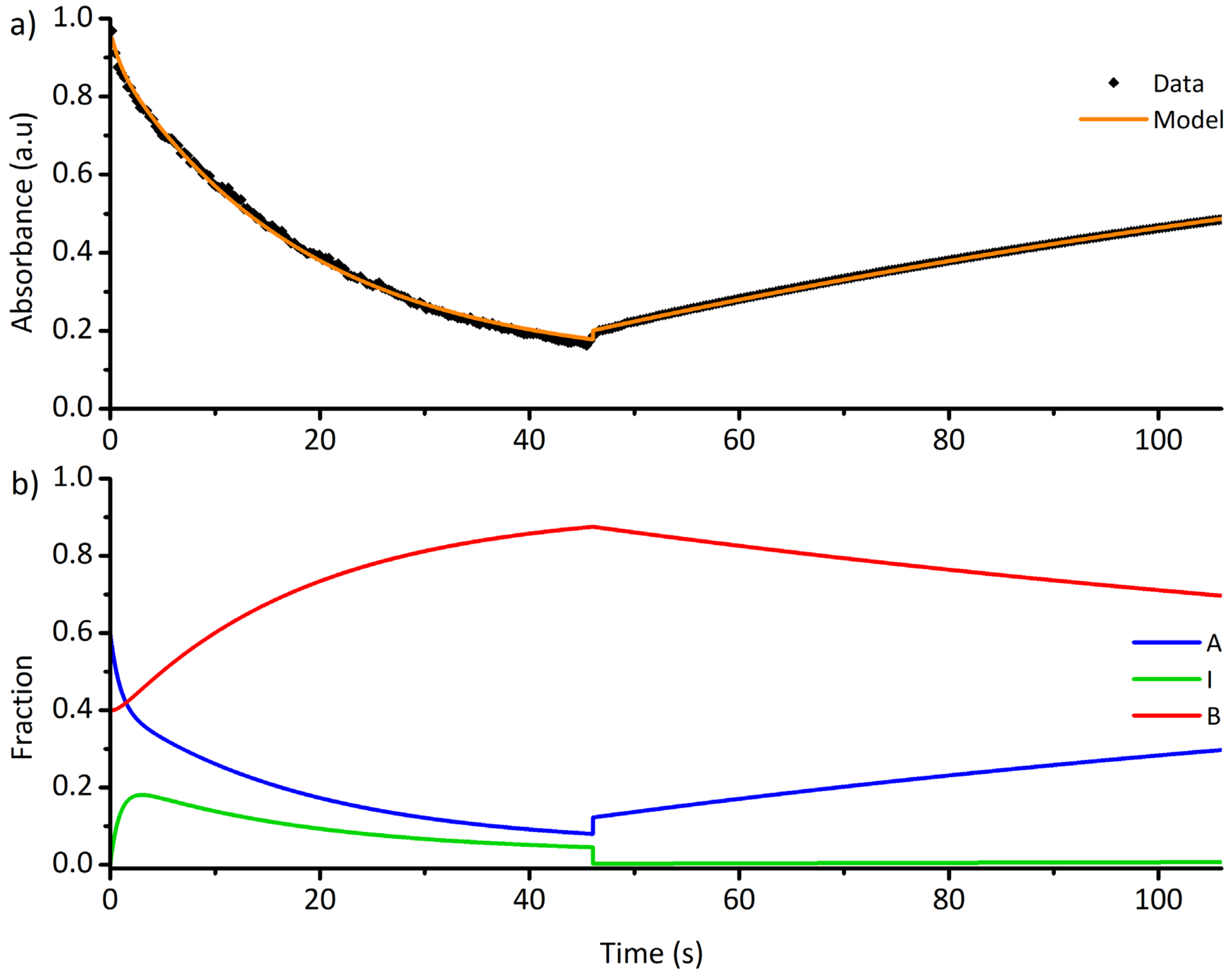

Figure S99. a) Absorbance (566 nm) measured during one photoswitching cycle on **13** in $CHCl_3$ and the predicted absorbance from the kinetic model. b) The predicted fractions A, I and B from the kinetic model.

29.14 Modelled kinetic data for 14 in chloroform

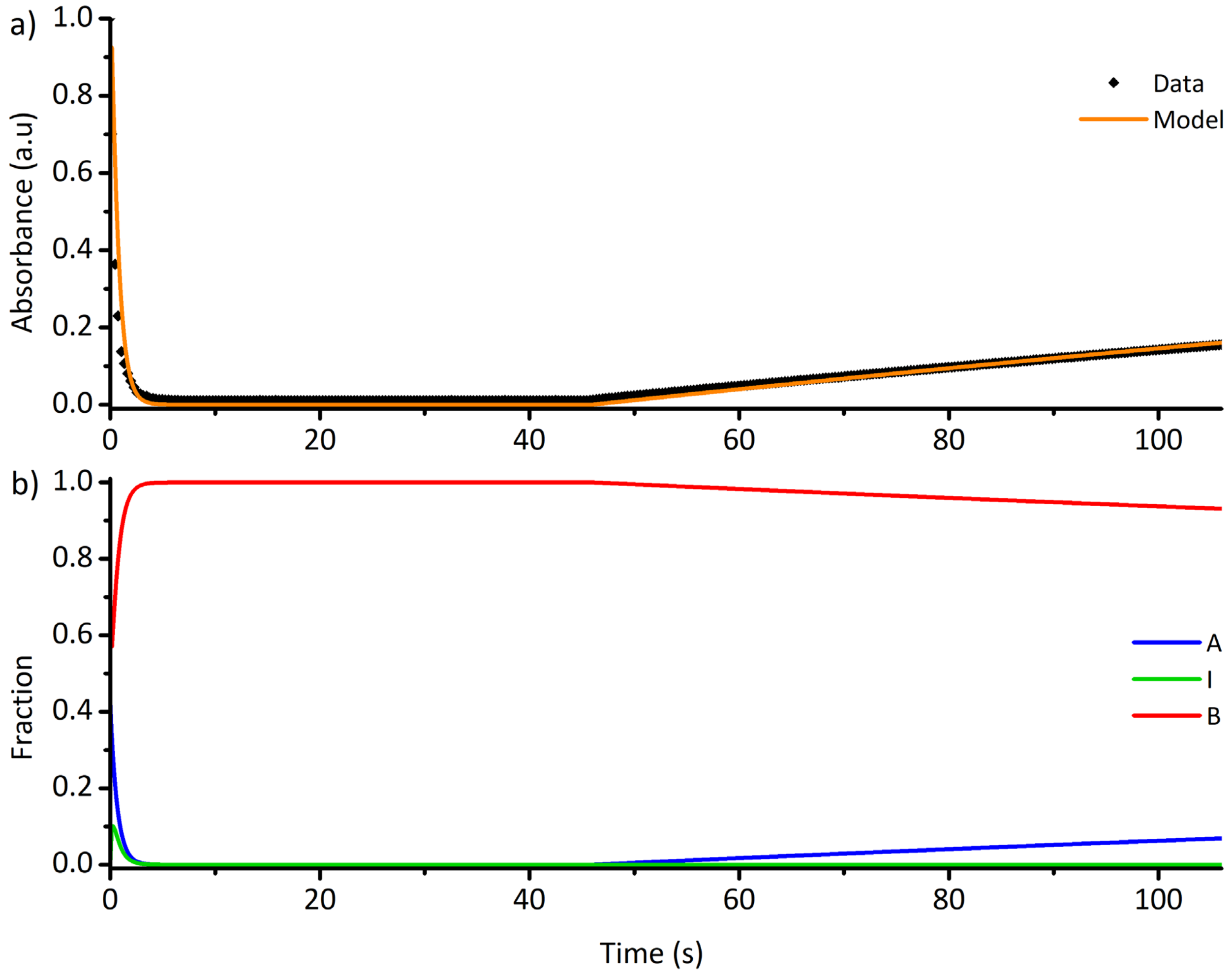

Figure S100. a) Absorbance (588 nm) measured during one photoswitching cycle on **14** in $CHCl_3$ and the predicted absorbance from the kinetic model. b) The predicted fractions A, I and B from the kinetic model.

30 Single switching cycles (full spectra) in MeTHF

30.1 UV-vis spectra of 1 in MeTHF during one photoswitching cycle

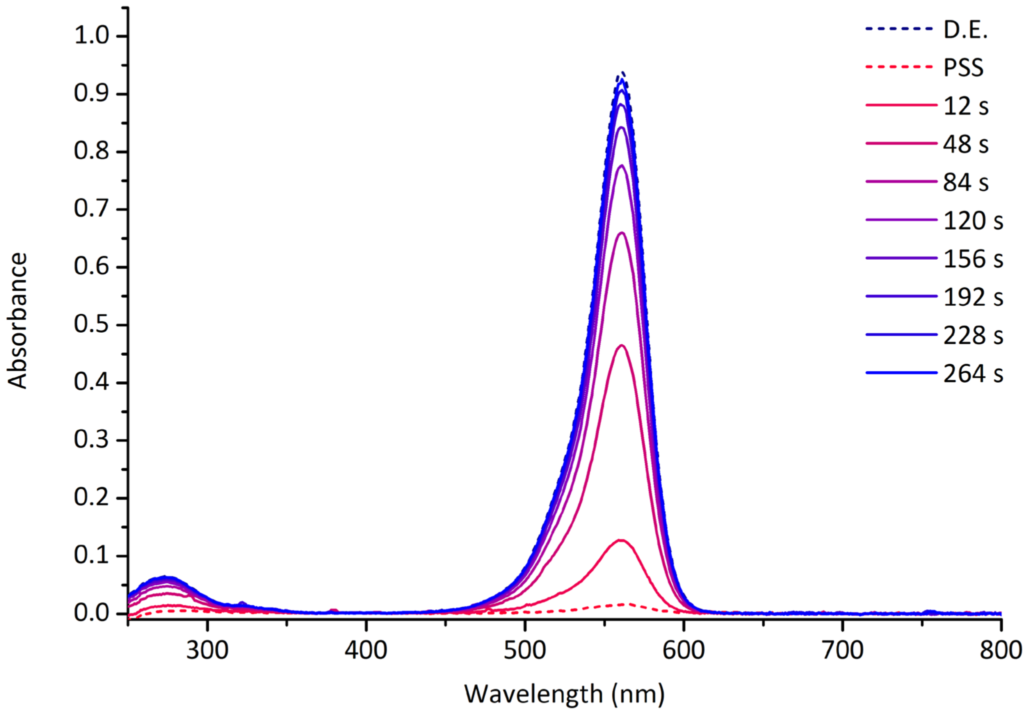

Figure S101. Photoswitching of **1** in MeTHF, followed by UV-vis spectroscopy. Before the switching cycle the sample was kept in the dark to reach a dark equilibrium (D.E.) state. The sample was irradiated (567 nm LED) for one minute, during which it reached a photostationary state (PSS), followed by 264 s in the dark. The times refer to time since the irradiation is switched off.

30.2 UV-vis spectra of 2 in MeTHF during one photoswitching cycle

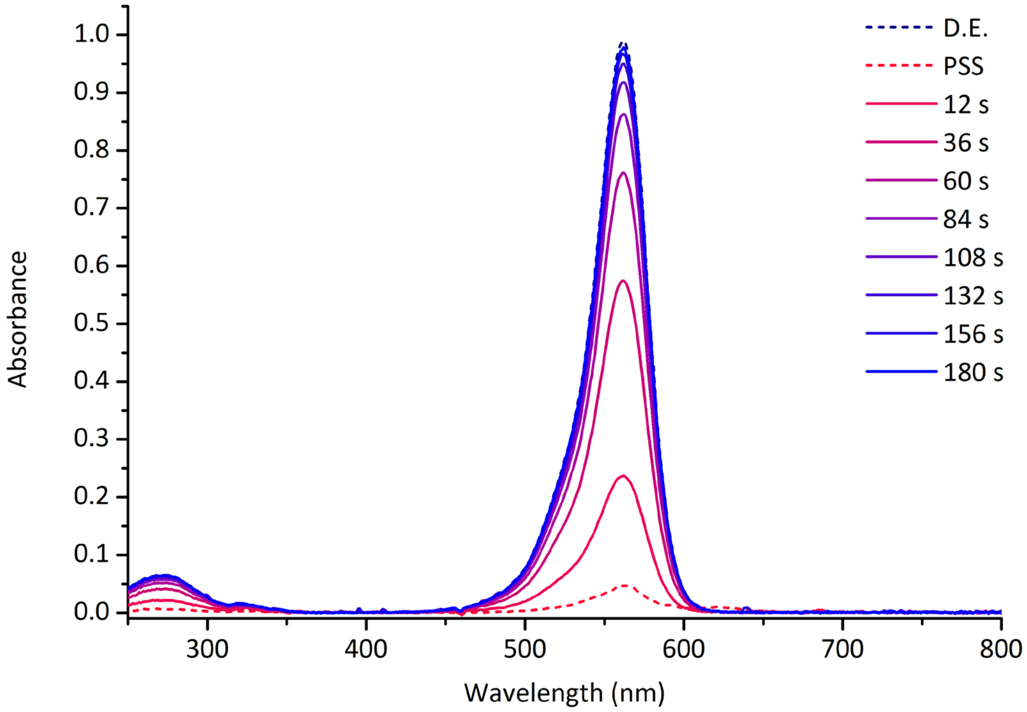

Figure S102. Photoswitching of **2** in MeTHF, followed by UV-vis spectroscopy. Before the switching cycle the sample was kept in the dark to reach a dark equilibrium (D.E.) state. The sample was irradiated (567 nm LED) for one minute, during which it reached a photostationary state (PSS), followed by 3 min in the dark. The times refer to time since the irradiation is switched off.

30.3 UV-vis spectra of 3 in MeTHF during one photoswitching cycle

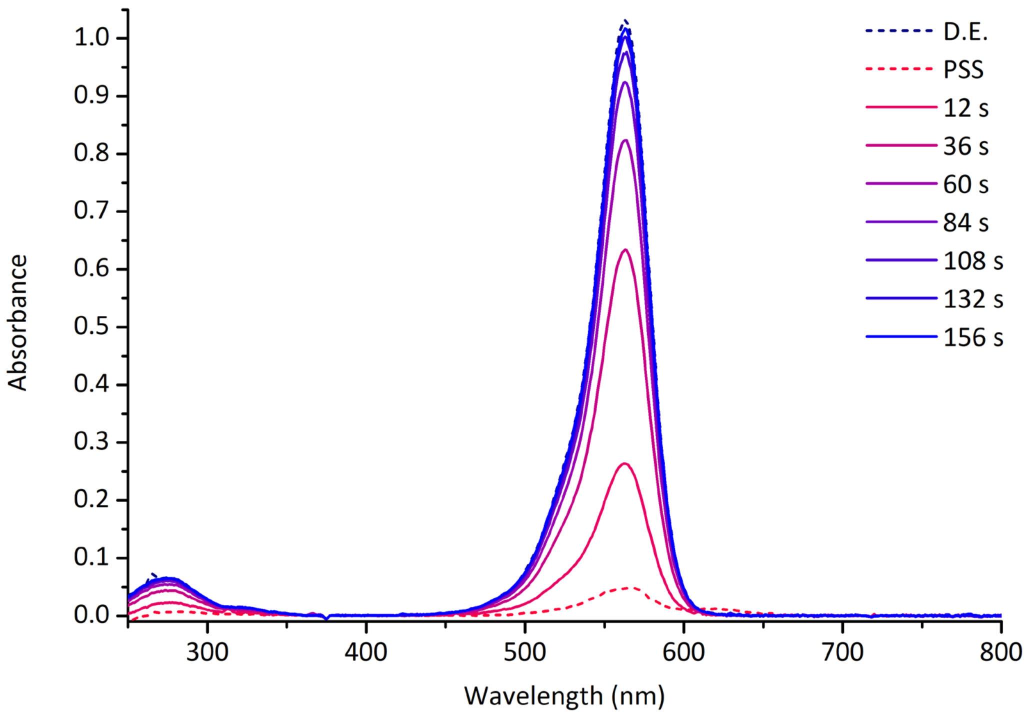

Figure S103. Photoswitching of **3** in MeTHF, followed by UV-vis spectroscopy. Before the switching cycle the sample was kept in the dark to reach a dark equilibrium (D.E.) state. The sample was irradiated (567 nm LED) for one minute, during which it reached a photostationary state (PSS), followed by 156 s in the dark. The times refer to time since the irradiation is switched off.

30.4 UV-vis spectra of 4 in MeTHF during one photoswitching cycle

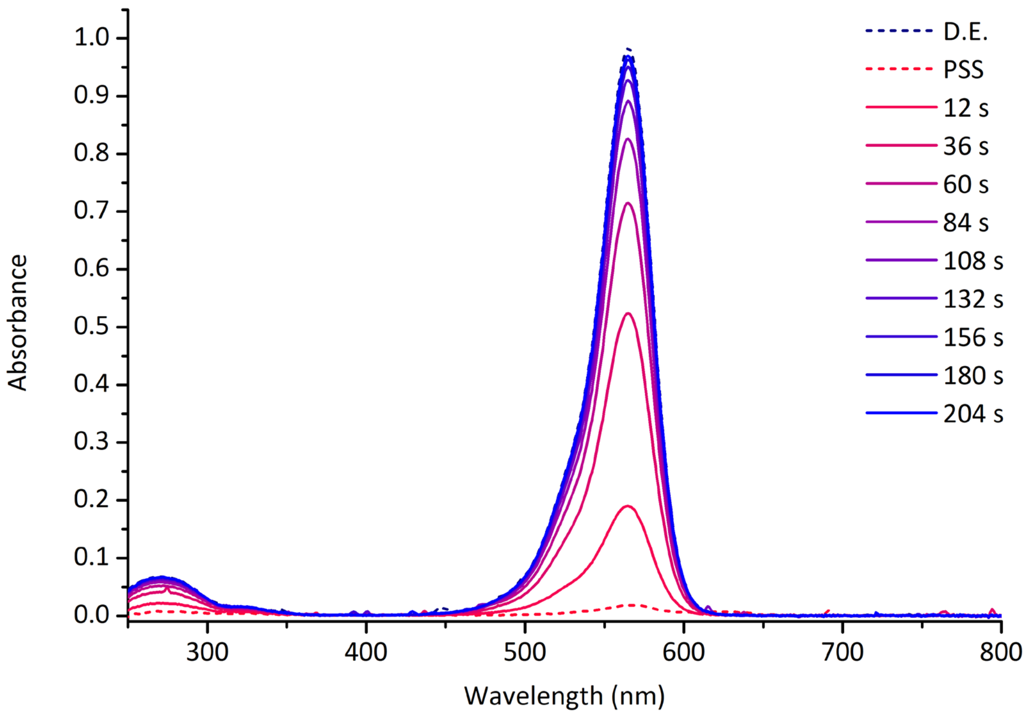

Figure S104. Photoswitching of **4** in MeTHF, followed by UV-vis spectroscopy. Before the switching cycle the sample was kept in the dark to reach a dark equilibrium (D.E.) state. The sample was irradiated (567 nm LED) for one minute, during which it reached a photostationary state (PSS), followed by 204 s in the dark. The times refer to time since the irradiation is switched off.

30.5 UV-vis spectra of 5 in MeTHF during one photoswitching cycle

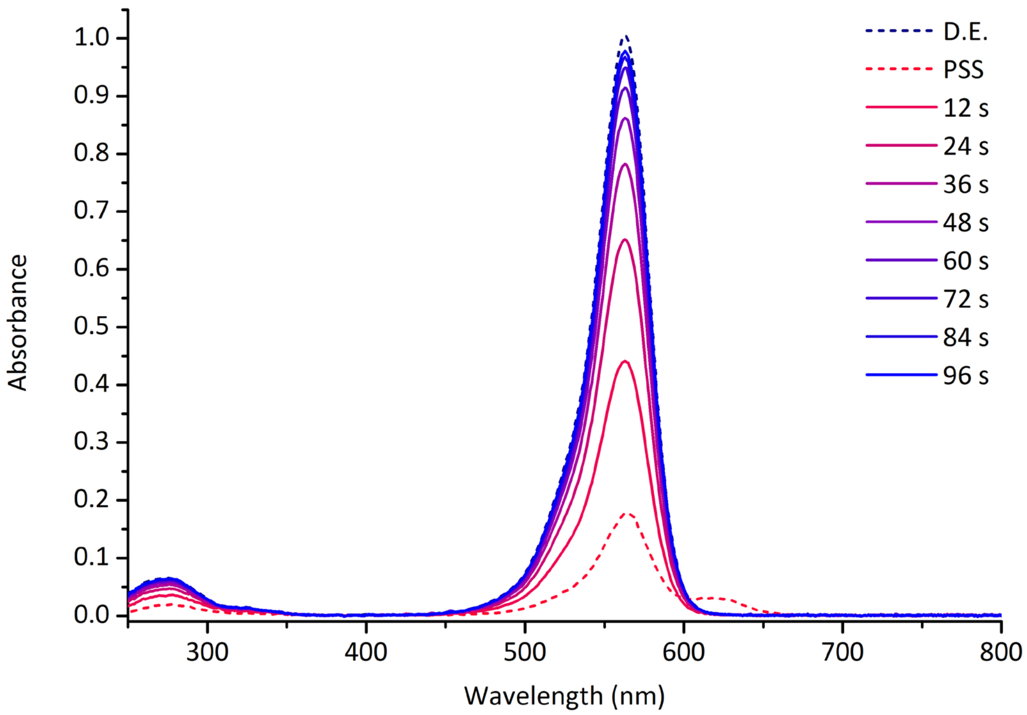

Figure S105. Photoswitching of **5** in MeTHF, followed by UV-vis spectroscopy. Before the switching cycle the sample was kept in the dark to reach a dark equilibrium (D.E.) state. The sample was irradiated (567 nm LED) for one minute, during which it reached a photostationary state (PSS), followed by 96 s in the dark. The times refer to time since the irradiation is switched off.

30.6 UV-vis spectra of 6 in MeTHF during one photoswitching cycle

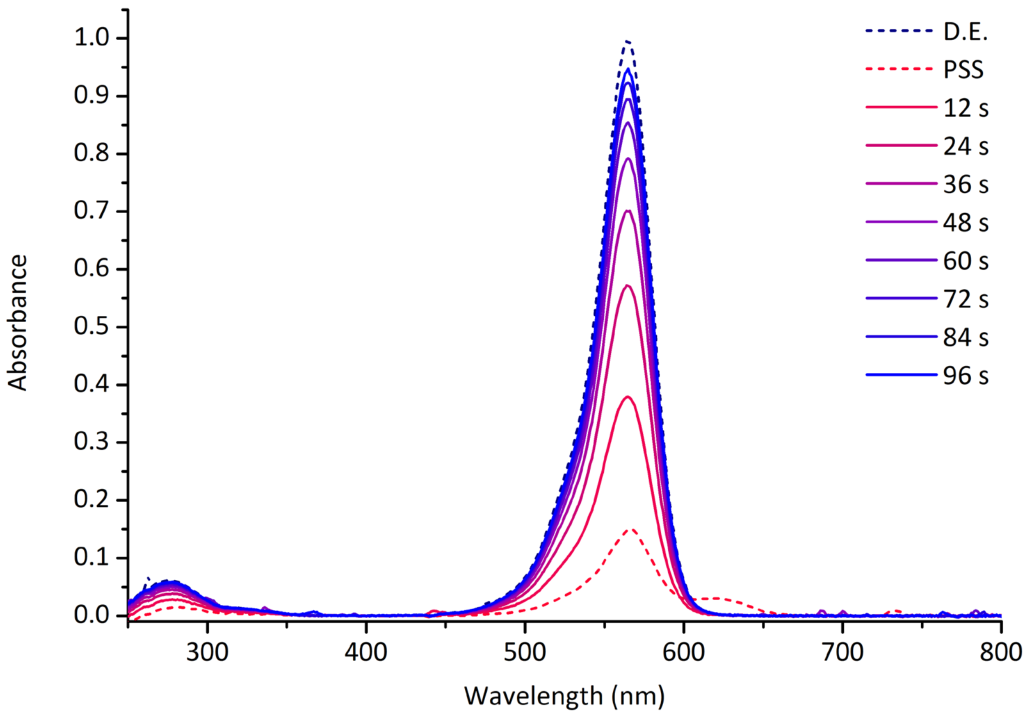

Figure S106. Photoswitching of **6** in MeTHF, followed by UV-vis spectroscopy. Before the switching cycle the sample was kept in the dark to reach a dark equilibrium (D.E.) state. The sample was irradiated (567 nm LED) for one minute, during which it reached a photostationary state (PSS), followed by 96 s in the dark. The times refer to time since the irradiation is switched off.

30.7 UV-vis spectra of 7 in MeTHF during one photoswitching cycle

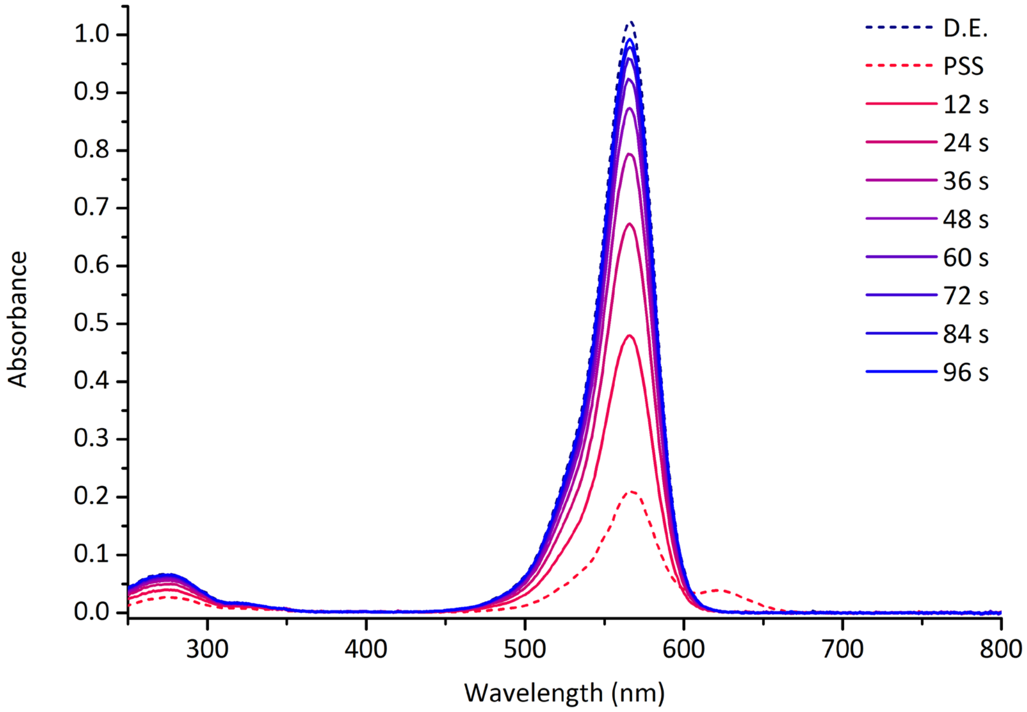

Figure S107. Photoswitching of **7** in MeTHF, followed by UV-vis spectroscopy. Before the switching cycle the sample was kept in the dark to reach a dark equilibrium (D.E.) state. The sample was irradiated (567 nm LED) for one minute, during which it reached a photostationary state (PSS), followed by 96 s in the dark. The times refer to time since the irradiation is switched off.

30.8 UV-vis spectra of **8** in MeTHF during one photoswitching cycle

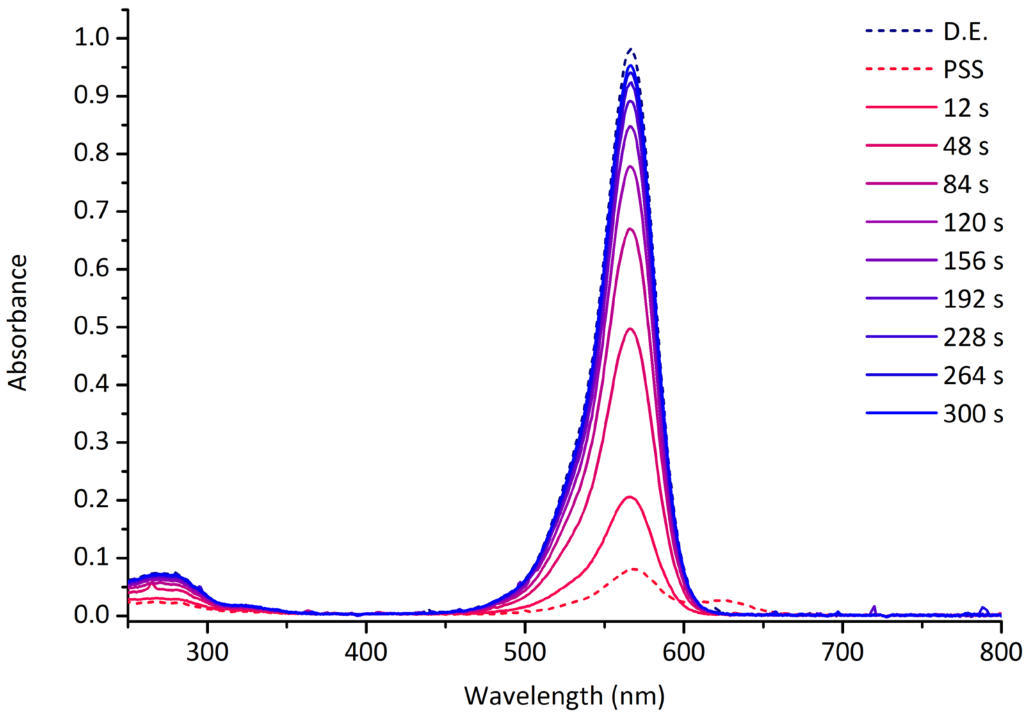

Figure S108. Photoswitching of **8** in MeTHF, followed by UV-vis spectroscopy. Before the switching cycle the sample was kept in the dark to reach a dark equilibrium (D.E.) state. The sample was irradiated (567 nm LED) for one minute, during which it reached a photostationary state (PSS), followed by 5 min in the dark. The times refer to time since the irradiation is switched off.

30.9 UV-vis spectra of **9** in MeTHF during one photoswitching cycle

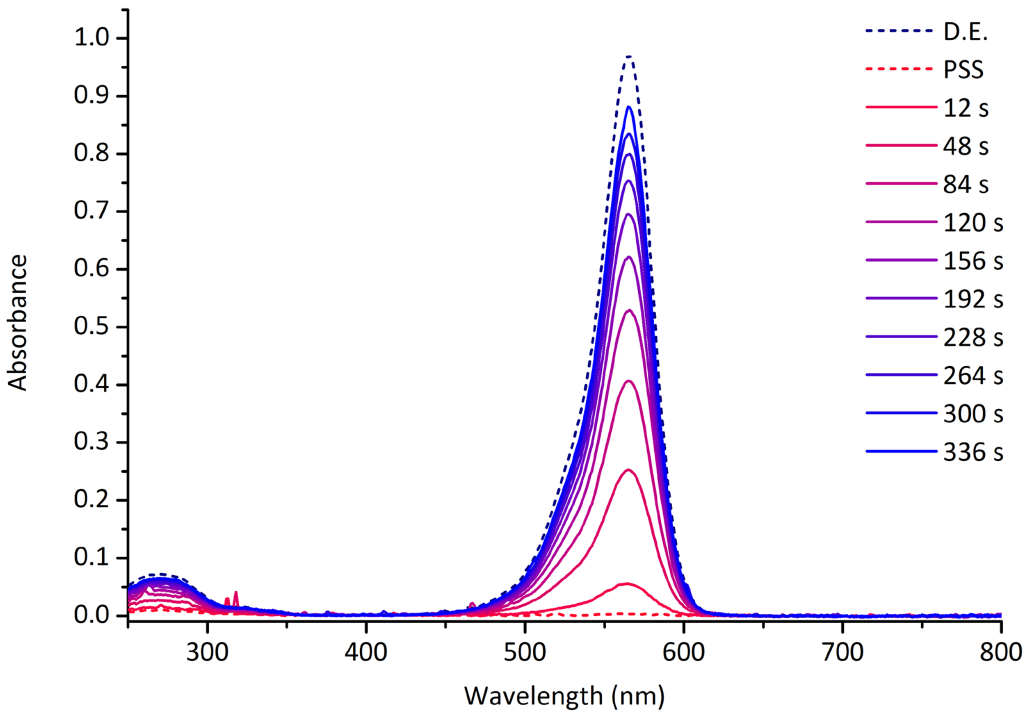

Figure S109. Photoswitching of **9** in MeTHF, followed by UV-vis spectroscopy. Before the switching cycle the sample was kept in the dark to reach a dark equilibrium (D.E.) state. The sample was irradiated (567 nm LED) for one minute, during which it reached a photostationary state (PSS), followed by 336 s in the dark. The times refer to time since the irradiation is switched off.

30.10 UV-vis spectra of 10 in MeTHF during one photoswitching cycle

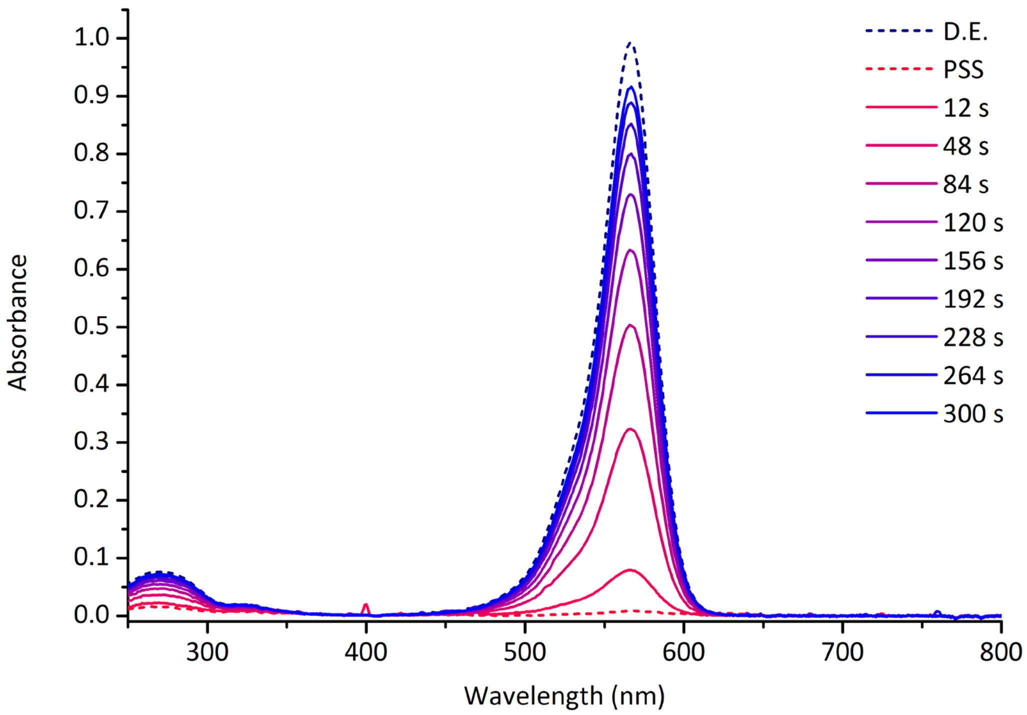

Figure S110. Photoswitching of **10** in MeTHF, followed by UV-vis spectroscopy. Before the switching cycle the sample was kept in the dark to reach a dark equilibrium (D.E.) state. The sample was irradiated (567 nm LED) for one minute, during which it reached a photostationary state (PSS), followed by 5 min in the dark. The times refer to time since the irradiation is switched off.

30.11 UV-vis spectra of 11 in MeTHF during one photoswitching cycle

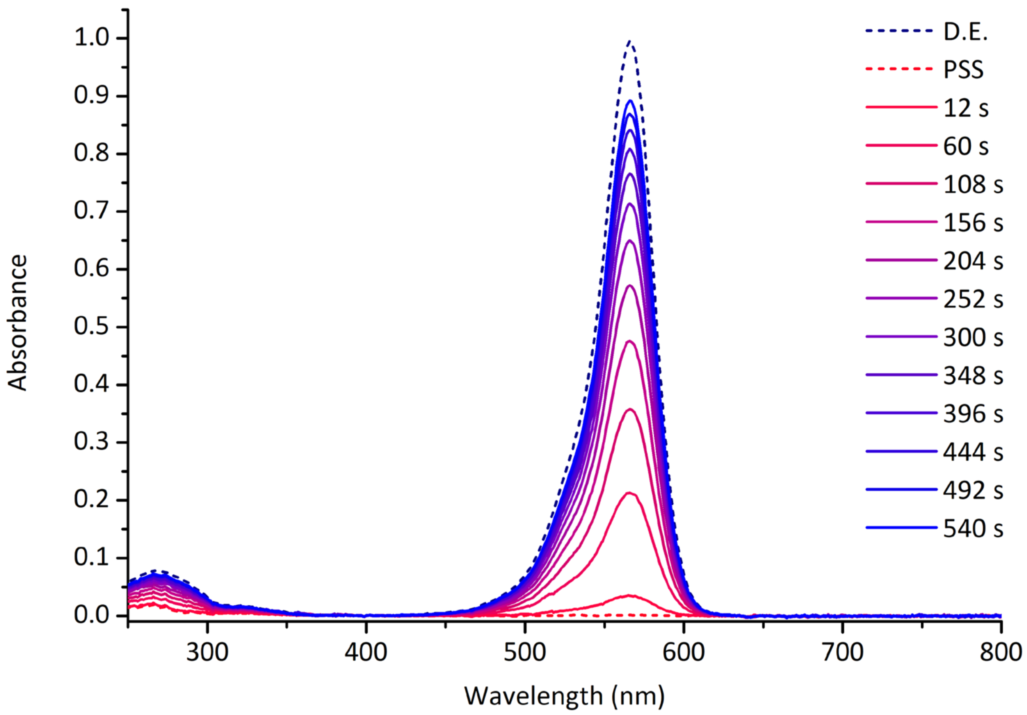

Figure S111. Photoswitching of **11** in MeTHF, followed by UV-vis spectroscopy. Before the switching cycle the sample was kept in the dark to reach a dark equilibrium (D.E.) state. The sample was irradiated (567 nm LED) for one minute, during which it reached a photostationary state (PSS), followed by 9 min in the dark. The times refer to time since the irradiation is switched off.

30.12 UV-vis spectra of 12 in MeTHF during one photoswitching cycle

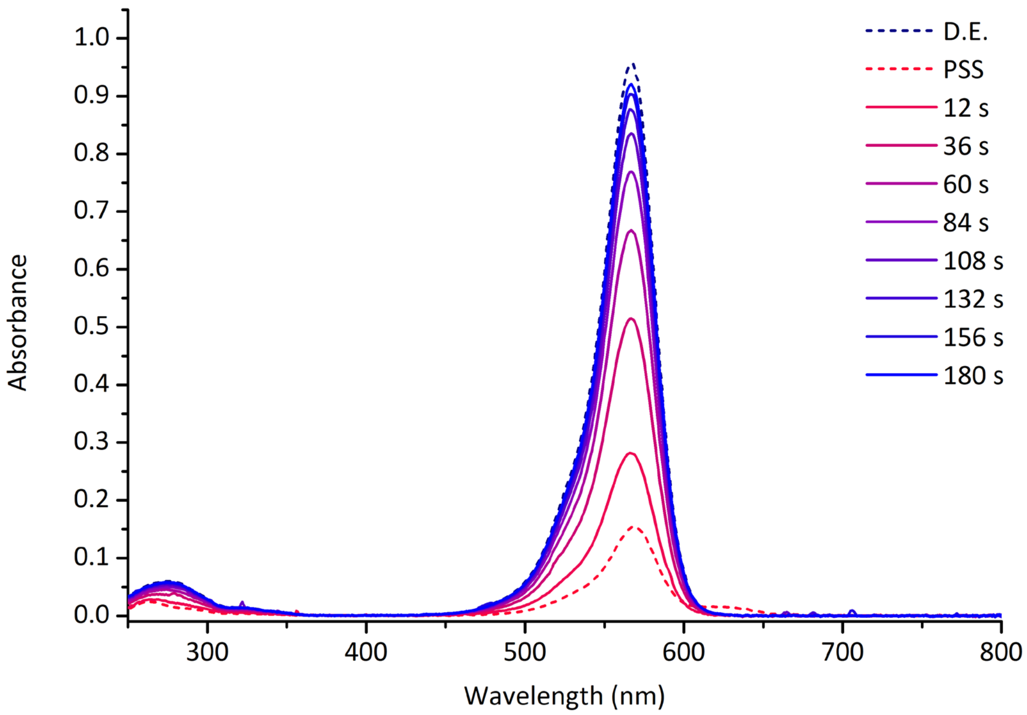

Figure S112. Photoswitching of **12** in MeTHF, followed by UV-vis spectroscopy. Before the switching cycle the sample was kept in the dark to reach a dark equilibrium (D.E.) state. The sample was irradiated (567 nm LED) for one minute, during which it reached a photostationary state (PSS), followed by 3 min in the dark. The times refer to time since the irradiation is switched off.

30.13 UV-vis spectra of 13 in MeTHF during one photoswitching cycle

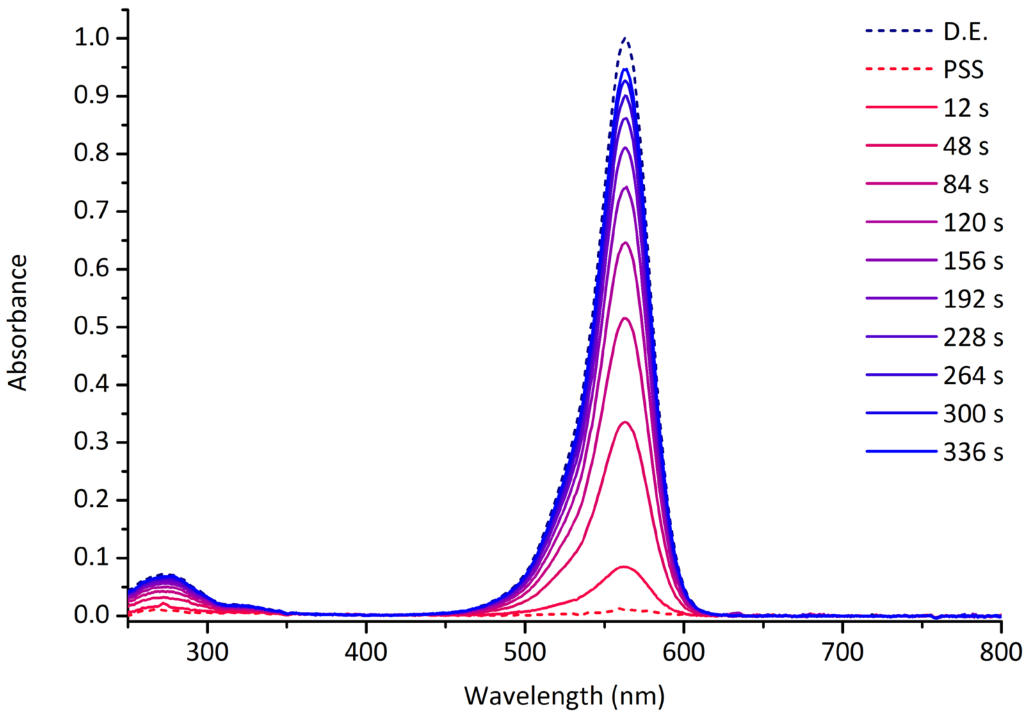

Figure S113. Photoswitching of **13** in MeTHF, followed by UV-vis spectroscopy. Before the switching cycle the sample was kept in the dark to reach a dark equilibrium (D.E.) state. The sample was irradiated (567 nm LED) for one minute, during which it reached a photostationary state (PSS), followed by 336 s in the dark. The times refer to time since the irradiation is switched off.

30.14 UV-vis spectra of 14 in MeTHF during one photoswitching cycle

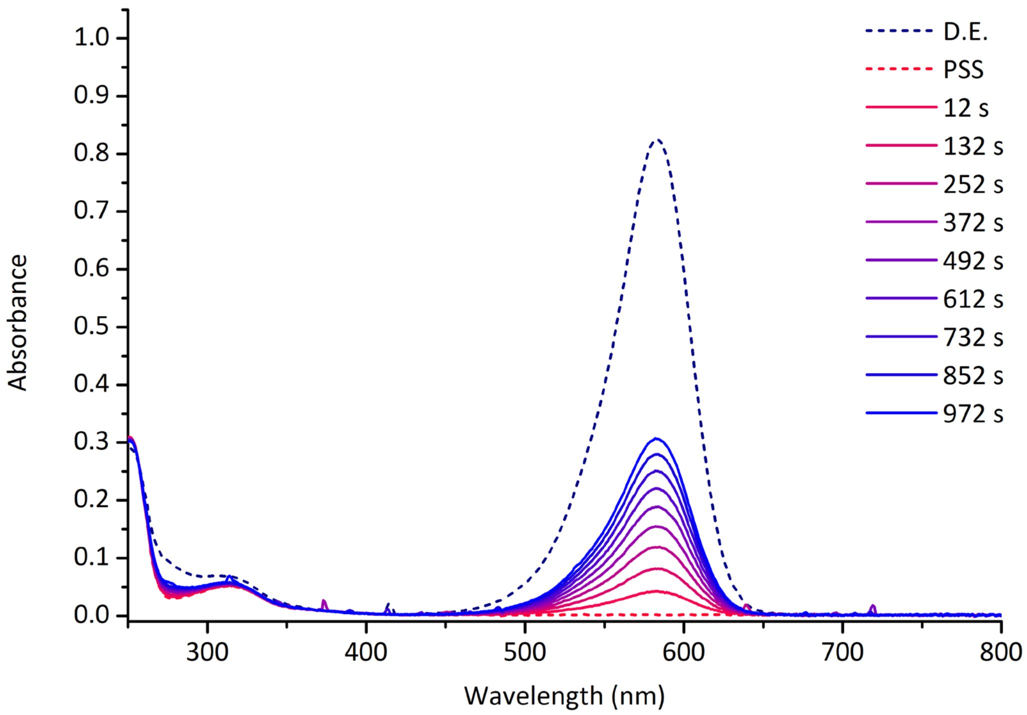

Figure S114. Photoswitching of **14** in MeTHF, followed by UV-vis spectroscopy. Before the switching cycle the sample was kept in the dark to reach a dark equilibrium (D.E.) state. The sample was irradiated (567 nm LED) for one minute, during which it reached a photostationary state (PSS), followed by 972 s in the dark. The times refer to time since the irradiation is switched off.

31 Fatigue resistance and thermal half-life calculations in MeTHF

Similar to the experiments described in SectionS28, the fatigue resistance of **1**-**14** was measured in 2-MeTHF at 298 K. The initial absorbance measured at λ_{max} (see Table S4) of the solutions was 1.00 ± 0.05 for **1-13**, and 0.81 for **14.** After equilibrating in the dark, the absorbance was measured for 1 hour without irradiating, to analyse for the decomposition in the dark. Subsequently, the samples were subjected to 50 photoswitching cycles of 45 s irradiation (567 nm LED), followed by 600 s in the dark. After the 50 cycles the absorbance was measured for a further 1 h in the dark to analyse for further decomposition in the dark. The temperature of the samples was kept at 298 K during the entire measurement.

31.1 Fatigue resistance and thermal half-life calculation for 1 in MeTHF

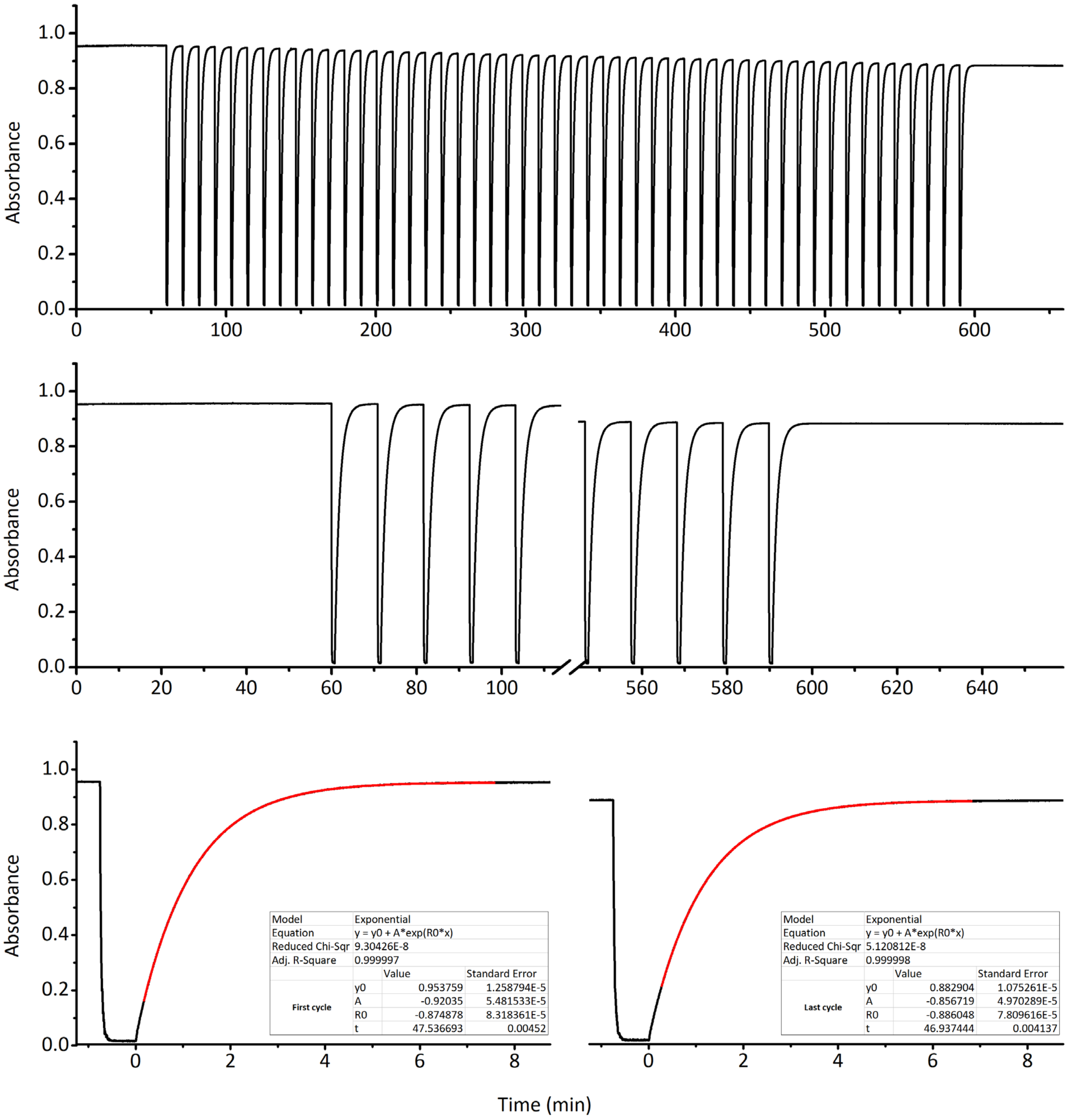

Figure S115. The fatigue resistance of **1** (MeTHF, 298 K) during 50 switching cycles of irradiation by 567 nm light. The absorbance (black lines) was measured at λ_{max} = 561 nm. Top: The complete 50 switching cycles. The first hour of measurement was in the dark and no decomposition was observed. Middle: Expansion of the first 5 and last 5 switching cycles. Bottom: Expansions of the first (left) and last (right) switching cycle with exponential curves (red) fitted to determine the apparent thermal half-life time (table inserts, time in s).

31.2 Fatigue resistance and thermal half-life calculation for 2 in MeTHF

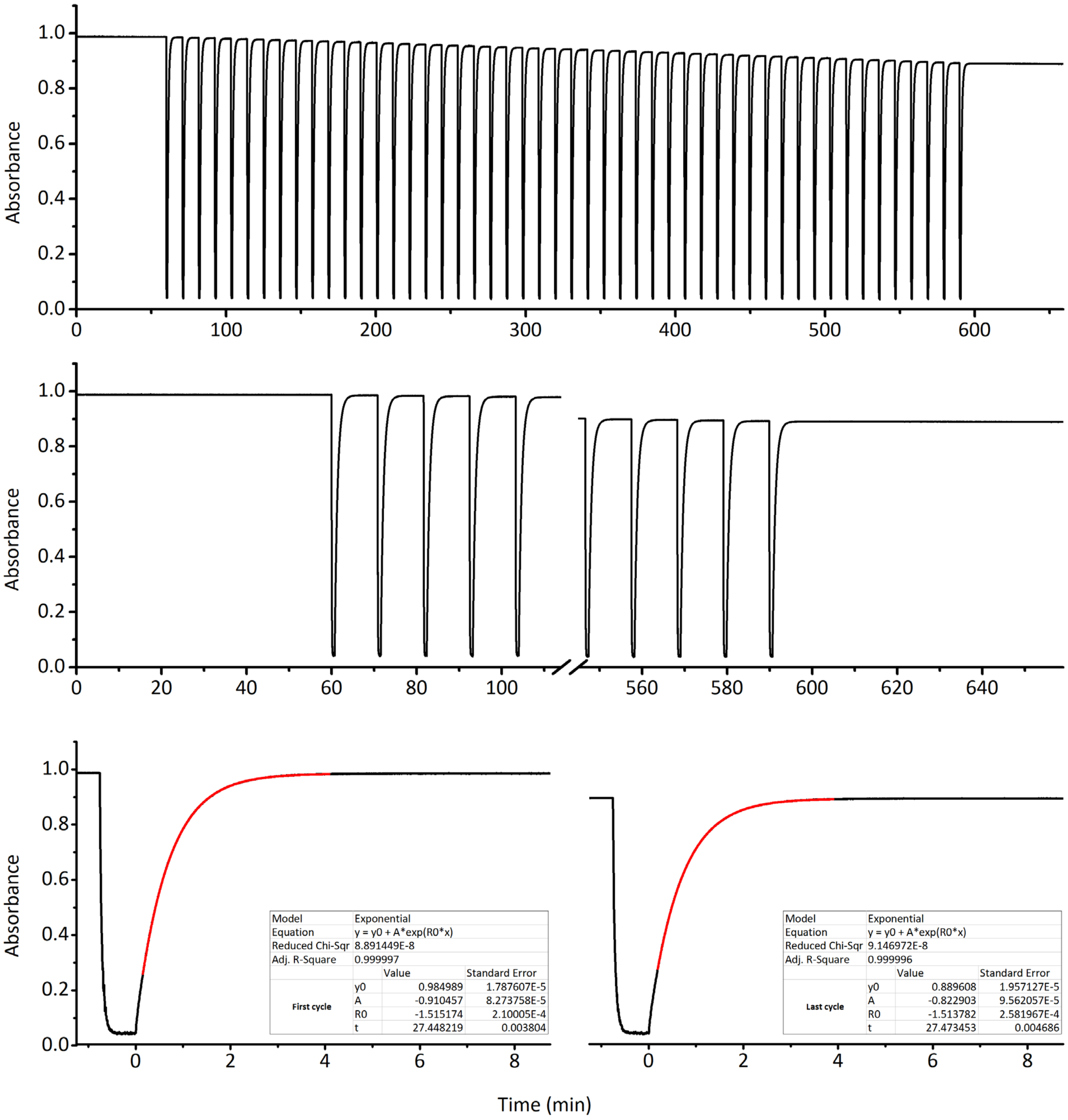

Figure S116. The fatigue resistance of **2** (MeTHF, 298 K) during 50 switching cycles of irradiation by 567 nm light. The absorbance (black lines) was measured at λ_{max} = 562 nm. Top: The complete 50 switching cycles. The first hour of measurement was in the dark and no decomposition was observed. Middle: Expansion of the first 5 and last 5 switching cycles. Bottom: Expansions of the first (left) and last (right) switching cycle with exponential curves (red) fitted to determine the apparent thermal half-life time (table inserts, time in s).

31.3 Fatigue resistance and thermal half-life calculation for 3 in MeTHF

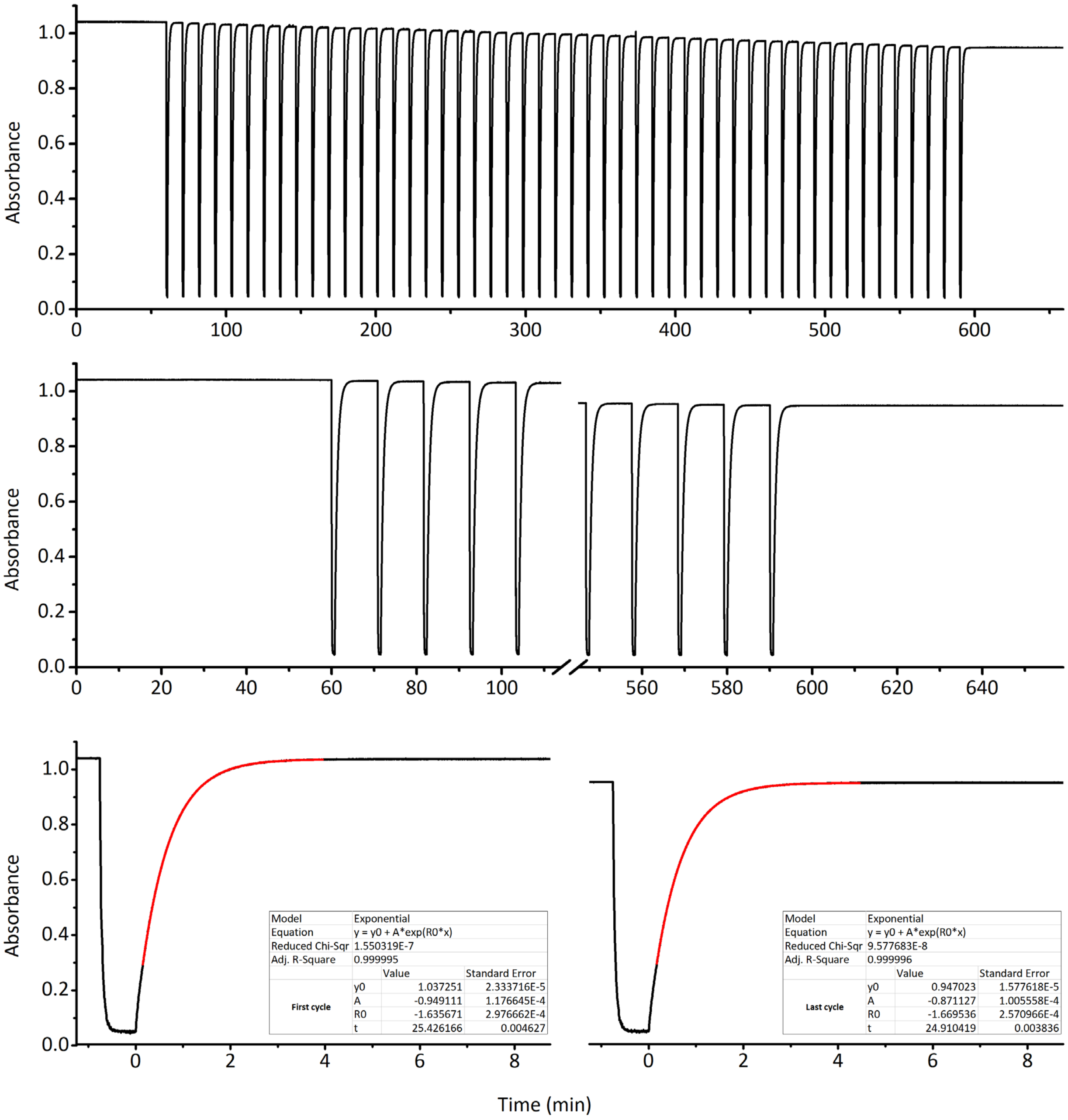

Figure S117. The fatigue resistance of **3** (MeTHF, 298 K) during 50 switching cycles of irradiation by 567 nm light. The absorbance (black lines) was measured at λ_{max} = 563 nm. Top: The complete 50 switching cycles. The first hour of measurement was in the dark and no decomposition was observed. Middle: Expansion of the first 5 and last 5 switching cycles. Bottom: Expansions of the first (left) and last (right) switching cycle with exponential curves (red) fitted to determine the apparent thermal half-life time (table inserts, time in s).

31.4 Fatigue resistance and thermal half-life calculation for 4 in MeTHF

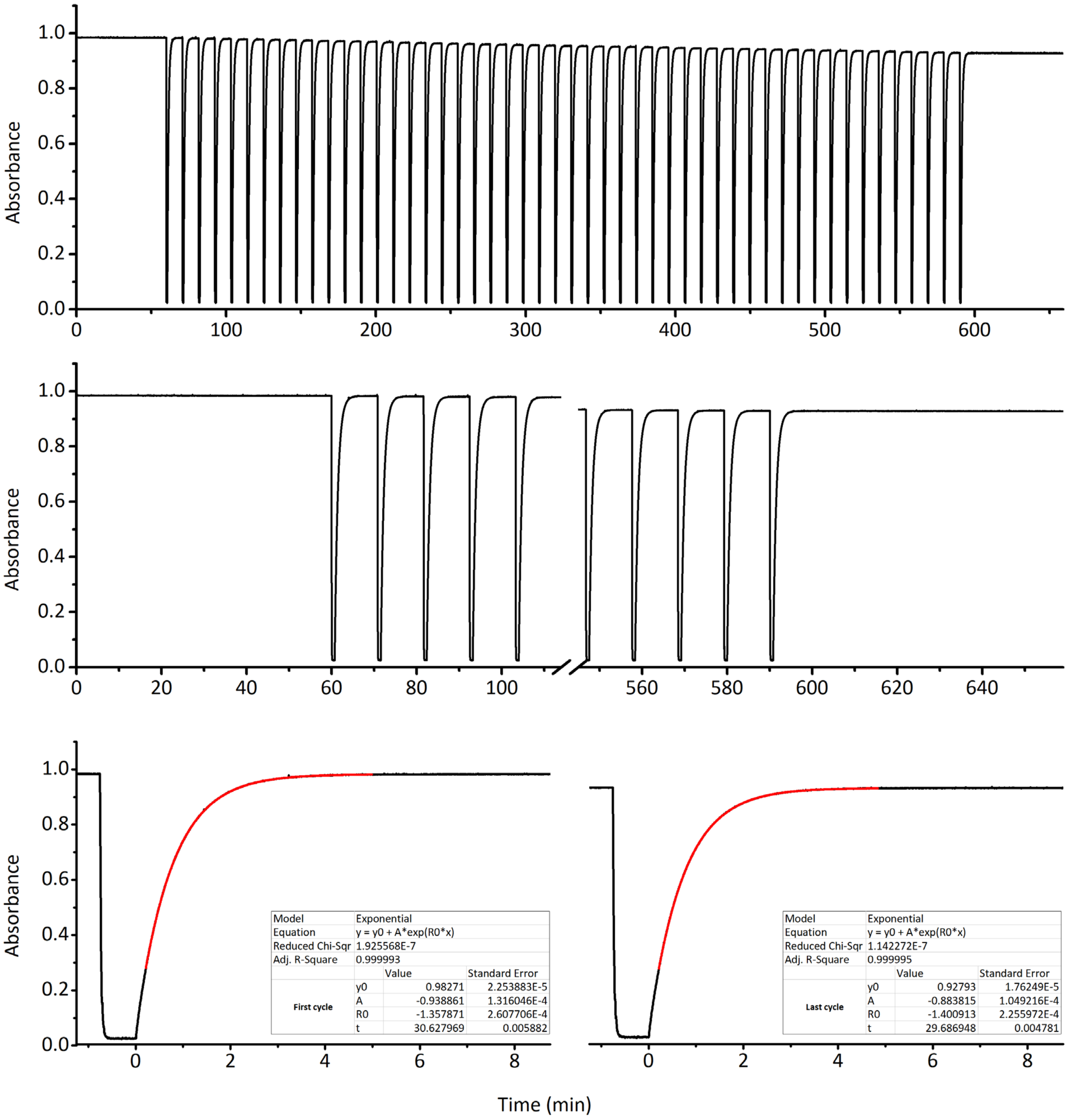

Model	Exponential		
Equation	y = y0 + A*exp(R0*x)		
Reduced Chi-Sqr	1.925568E-7		
Adj. R-Square	0.999993		
		Value	Standard Error
First cycle	y0	0.98271	2.253883E-5
	A	-0.938861	1.316046E-4
	R0	-1.357871	2.607706E-4
	t	30.627969	0.005882

Model	Exponential		
Equation	y = y0 + A*exp(R0*x)		
Reduced Chi-Sqr	1.142272E-7		
Adj. R-Square	0.999995		
		Value	Standard Error
Last cycle	y0	0.92793	1.76249E-5
	A	-0.883815	1.049216E-4
	R0	-1.400913	2.255972E-4
	t	29.686948	0.004781

Figure S118. The fatigue resistance of **4** (MeTHF, 298 K) during 50 switching cycles of irradiation by 567 nm light. The absorbance (black lines) was measured at λ_{max} = 565 nm. Top: The complete 50 switching cycles. The first hour of measurement was in the dark and no decomposition was observed. Middle: Expansion of the first 5 and last 5 switching cycles. Bottom: Expansions of the first (left) and last (right) switching cycle with exponential curves (red) fitted to determine the apparent thermal half-life time (table inserts, time in s).

31.5 Fatigue resistance and thermal half-life calculation for 5 in MeTHF

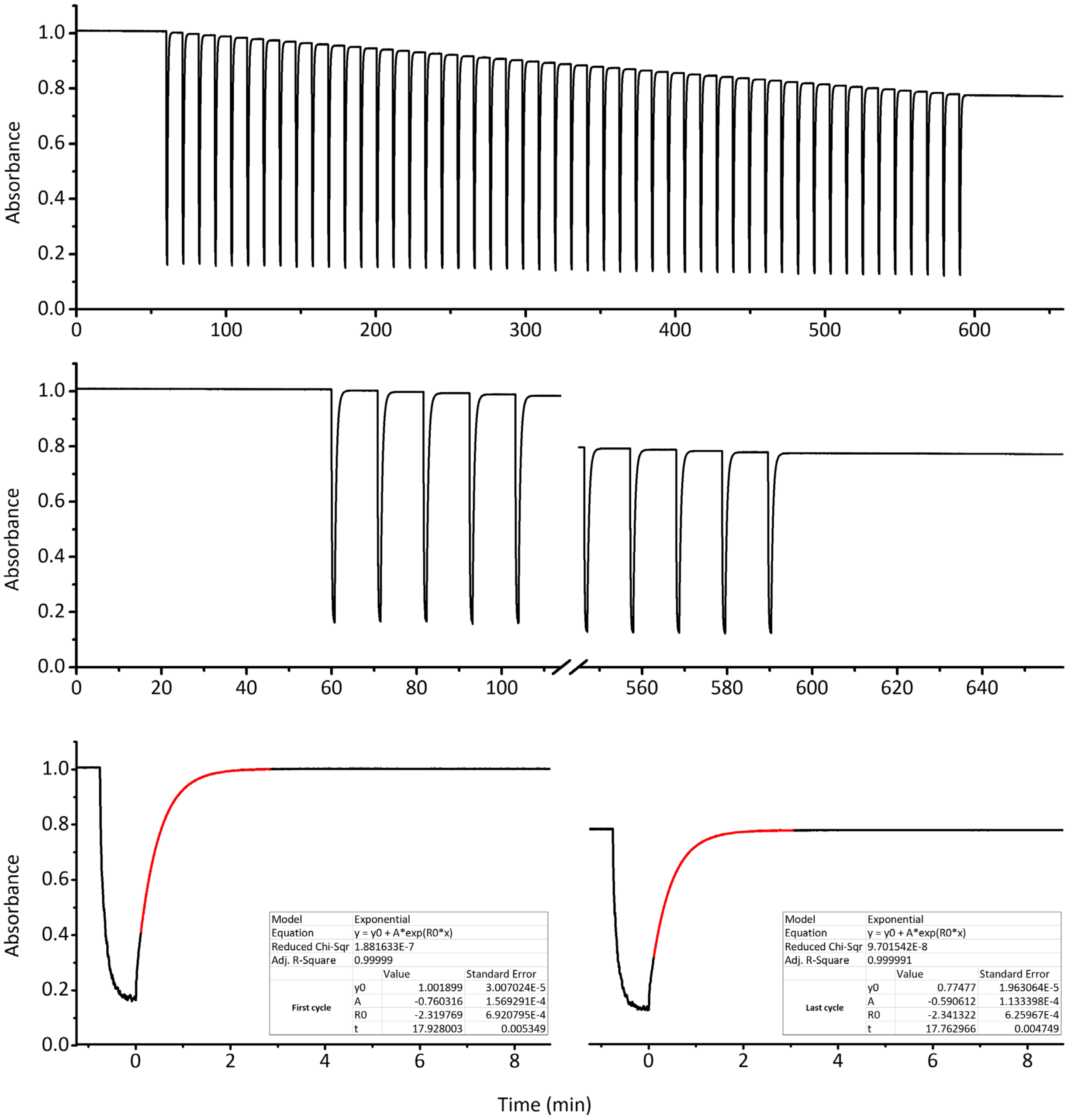

Figure S119. The fatigue resistance of **5** (MeTHF, 298 K) during 50 switching cycles of irradiation by 567 nm light. The absorbance (black lines) was measured at λ_{max} = 563 nm. Top: The complete 50 switching cycles. The first hour of measurement was in the dark and no decomposition was observed. Middle: Expansion of the first 5 and last 5 switching cycles. Bottom: Expansions of the first (left) and last (right) switching cycle with exponential curves (red) fitted to determine the apparent thermal half-life time (table inserts, time in s).

31.6 Fatigue resistance and thermal half-life calculation for 6 in MeTHF

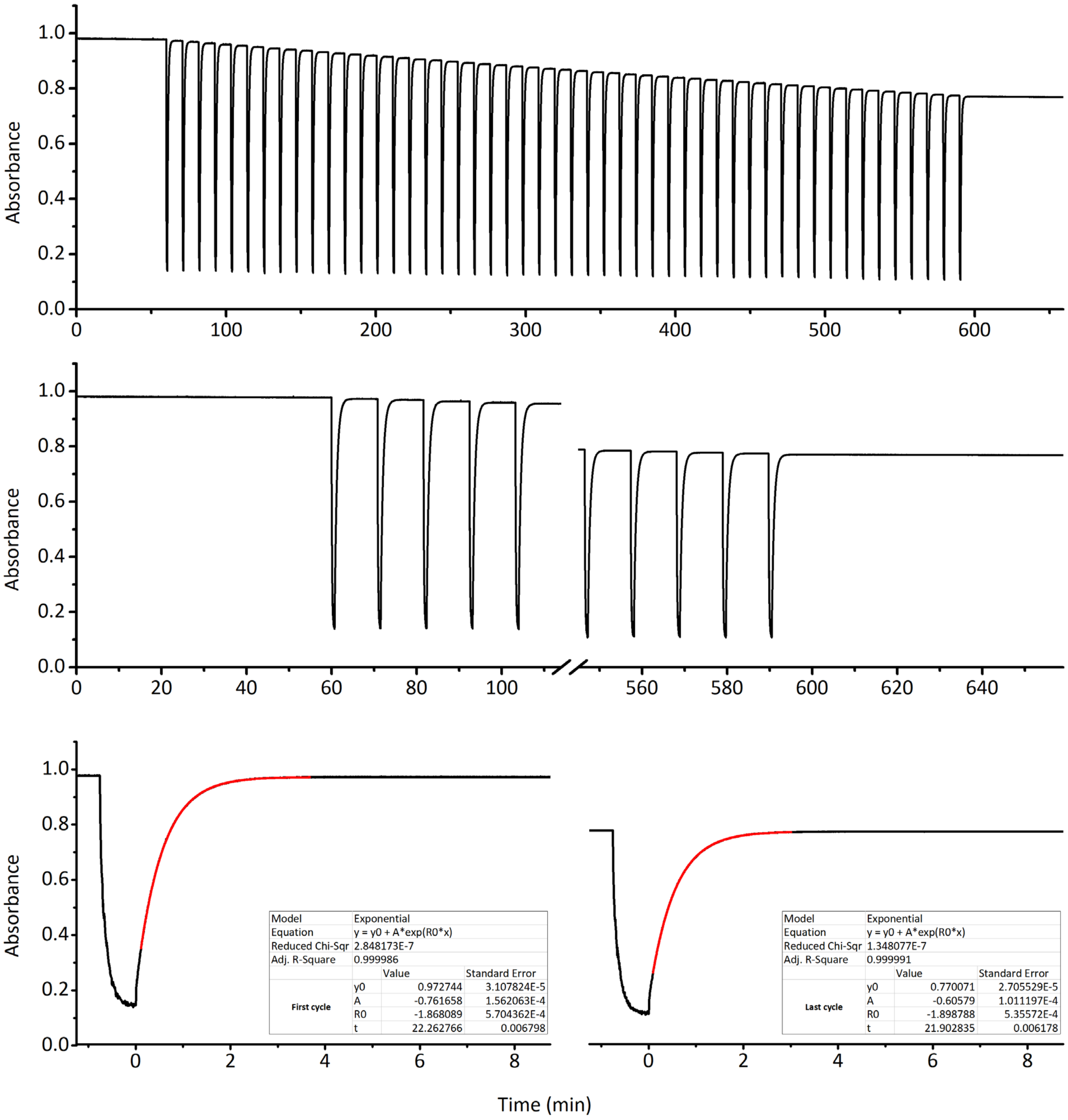

Figure S120. The fatigue resistance of **6** (MeTHF, 298 K) during 50 switching cycles of irradiation by 567 nm light. The absorbance (black lines) was measured at λ_{max} = 565 nm. Top: The complete 50 switching cycles. The first hour of measurement was in the dark and no decomposition was observed. Middle: Expansion of the first 5 and last 5 switching cycles. Bottom: Expansions of the first (left) and last (right) switching cycle with exponential curves (red) fitted to determine the apparent thermal half-life time (table inserts, time in s).

31.7 Fatigue resistance and thermal half-life calculation for 7 in MeTHF

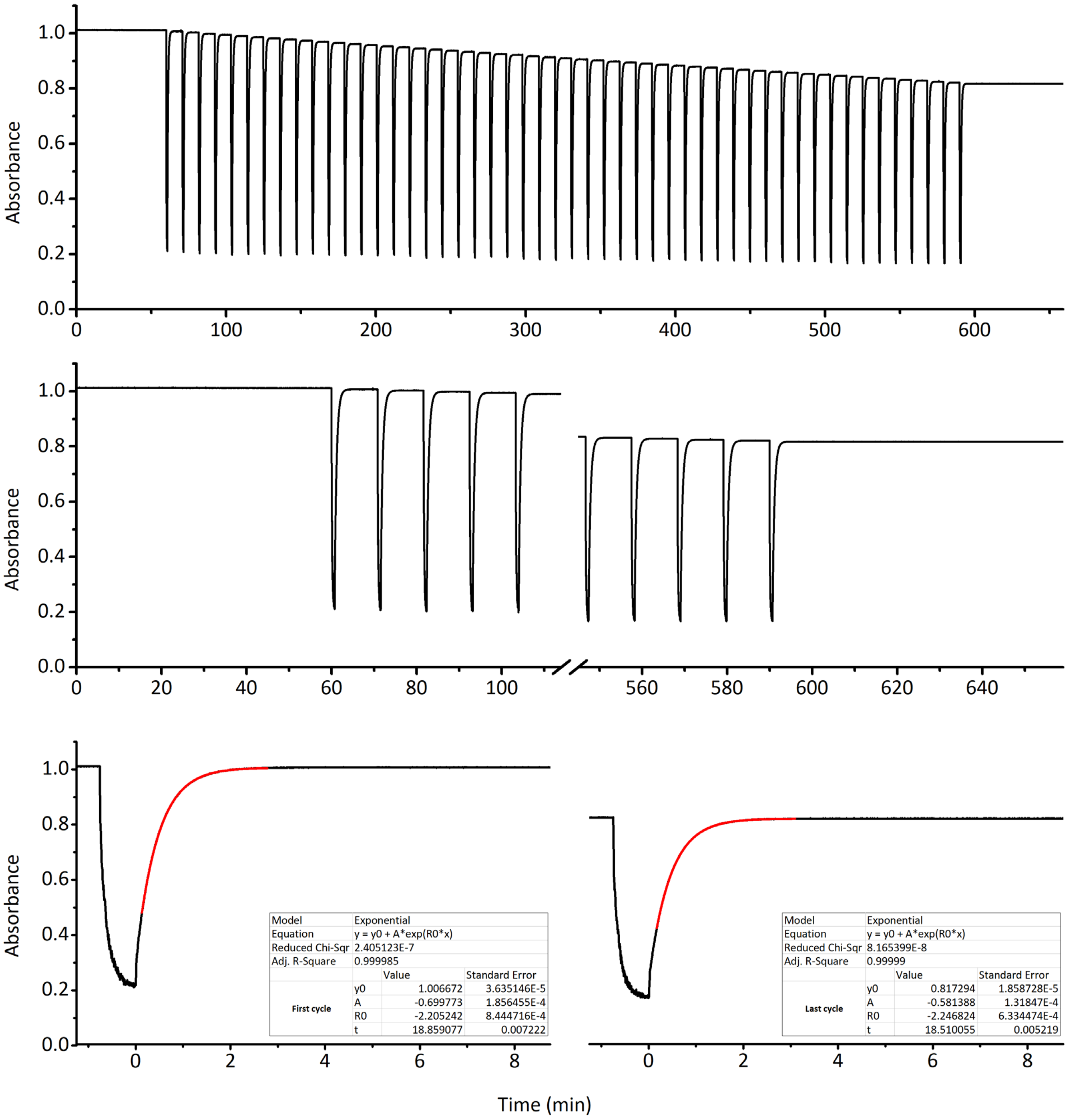

Figure S121. The fatigue resistance of **7** (MeTHF, 298 K) during 50 switching cycles of irradiation by 567 nm light. The absorbance (black lines) was measured at λ_{max} = 566 nm. Top: The complete 50 switching cycles. The first hour of measurement was in the dark and no decomposition was observed. Middle: Expansion of the first 5 and last 5 switching cycles. Bottom: Expansions of the first (left) and last (right) switching cycle with exponential curves (red) fitted to determine the apparent thermal half-life time (table inserts, time in s).

31.8 Fatigue resistance and thermal half-life calculation for 8 in MeTHF

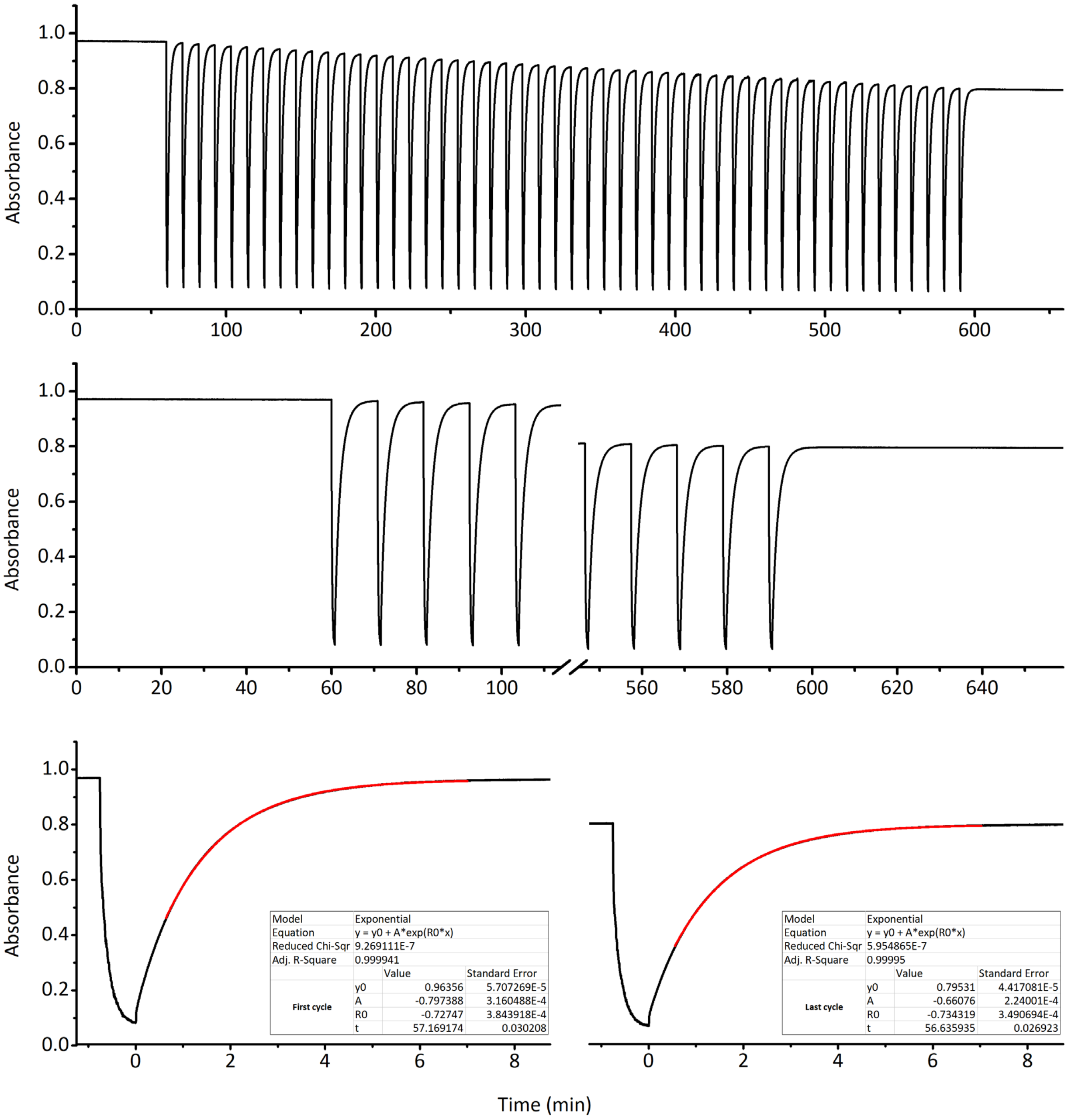

Figure S122. The fatigue resistance of **8** (MeTHF, 298 K) during 50 switching cycles of irradiation by 567 nm light. The absorbance (black lines) was measured at λ_{max} = 566 nm. Top: The complete 50 switching cycles. The first hour of measurement was in the dark and no decomposition was observed. Middle: Expansion of the first 5 and last 5 switching cycles. Bottom: Expansions of the first (left) and last (right) switching cycle with exponential curves (red) fitted to determine the apparent thermal half-life time (table inserts, time in s).

31.9 Fatigue resistance and thermal half-life calculation for 9 in MeTHF

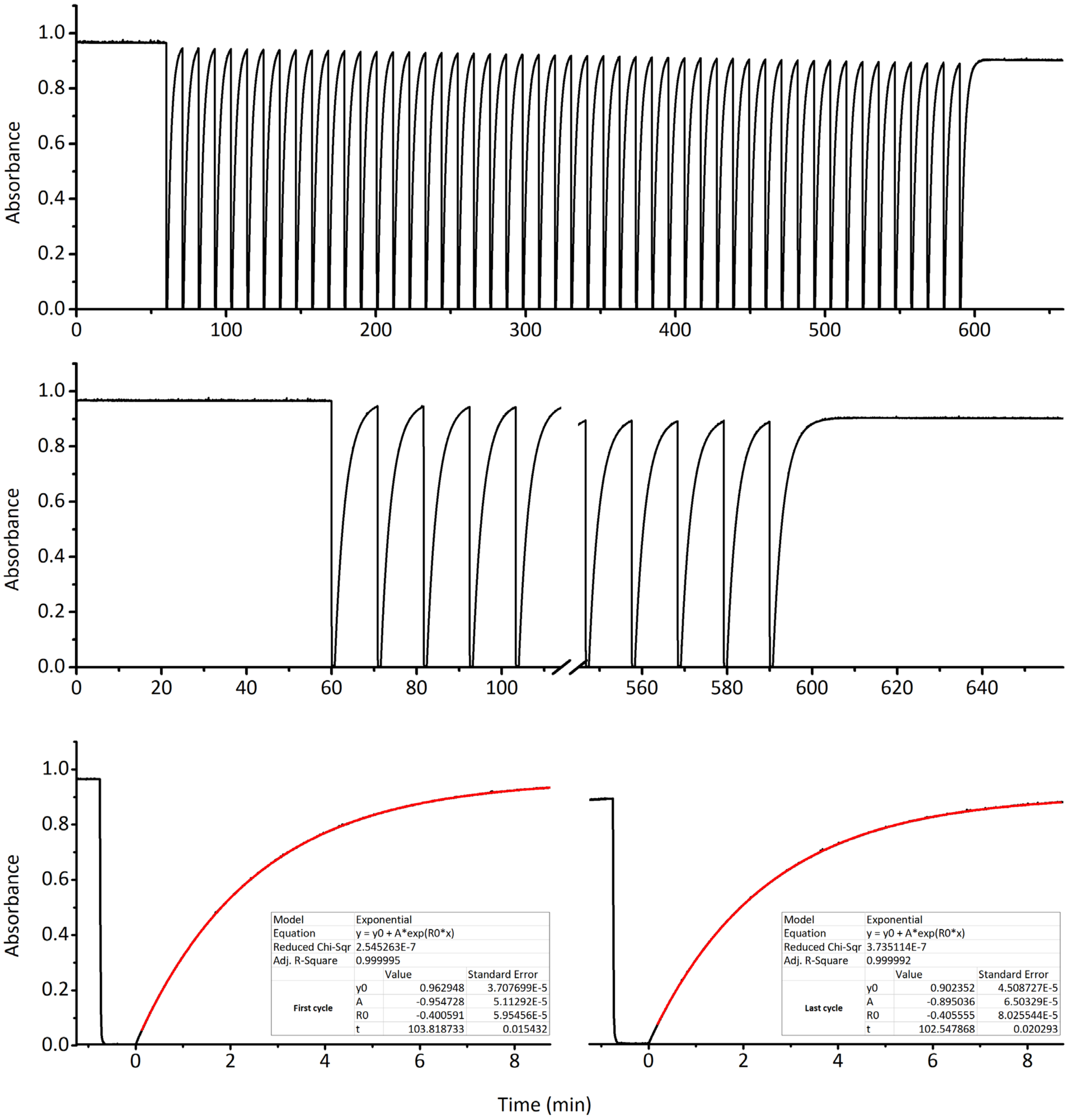

Figure S123. The fatigue resistance of **9** (MeTHF, 298 K) during 50 switching cycles of irradiation by 567 nm light. The absorbance (black lines) was measured at λ_{max} = 565 nm. Top: The complete 50 switching cycles. The first hour of measurement was in the dark and no decomposition was observed. Middle: Expansion of the first 5 and last 5 switching cycles. Bottom: Expansions of the first (left) and last (right) switching cycle with exponential curves (red) fitted to determine the apparent thermal half-life time (table inserts, time in s).

31.10 Fatigue resistance and thermal half-life calculation for 10 in MeTHF

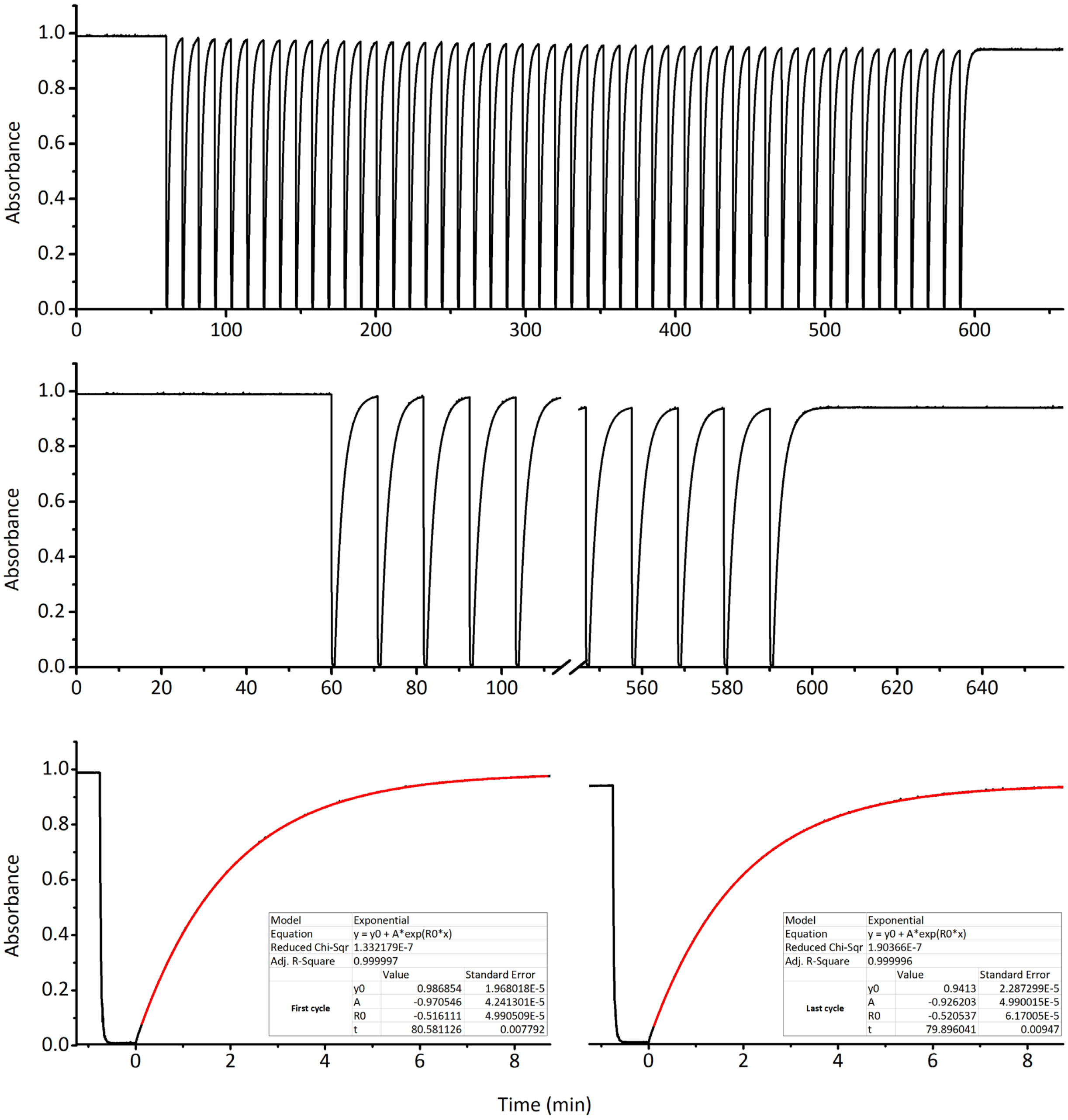

Figure S124. The fatigue resistance of **10** (MeTHF, 298 K) during 50 switching cycles of irradiation by 567 nm light. The absorbance (black lines) was measured at λ_{max} = 567 nm. Top: The complete 50 switching cycles. The first hour of measurement was in the dark and no decomposition was observed. Middle: Expansion of the first 5 and last 5 switching cycles. Bottom: Expansions of the first (left) and last (right) switching cycle with exponential curves (red) fitted to determine the apparent thermal half-life time (table inserts, time in s).

31.11 Fatigue resistance and thermal half-life calculation for 11 in MeTHF

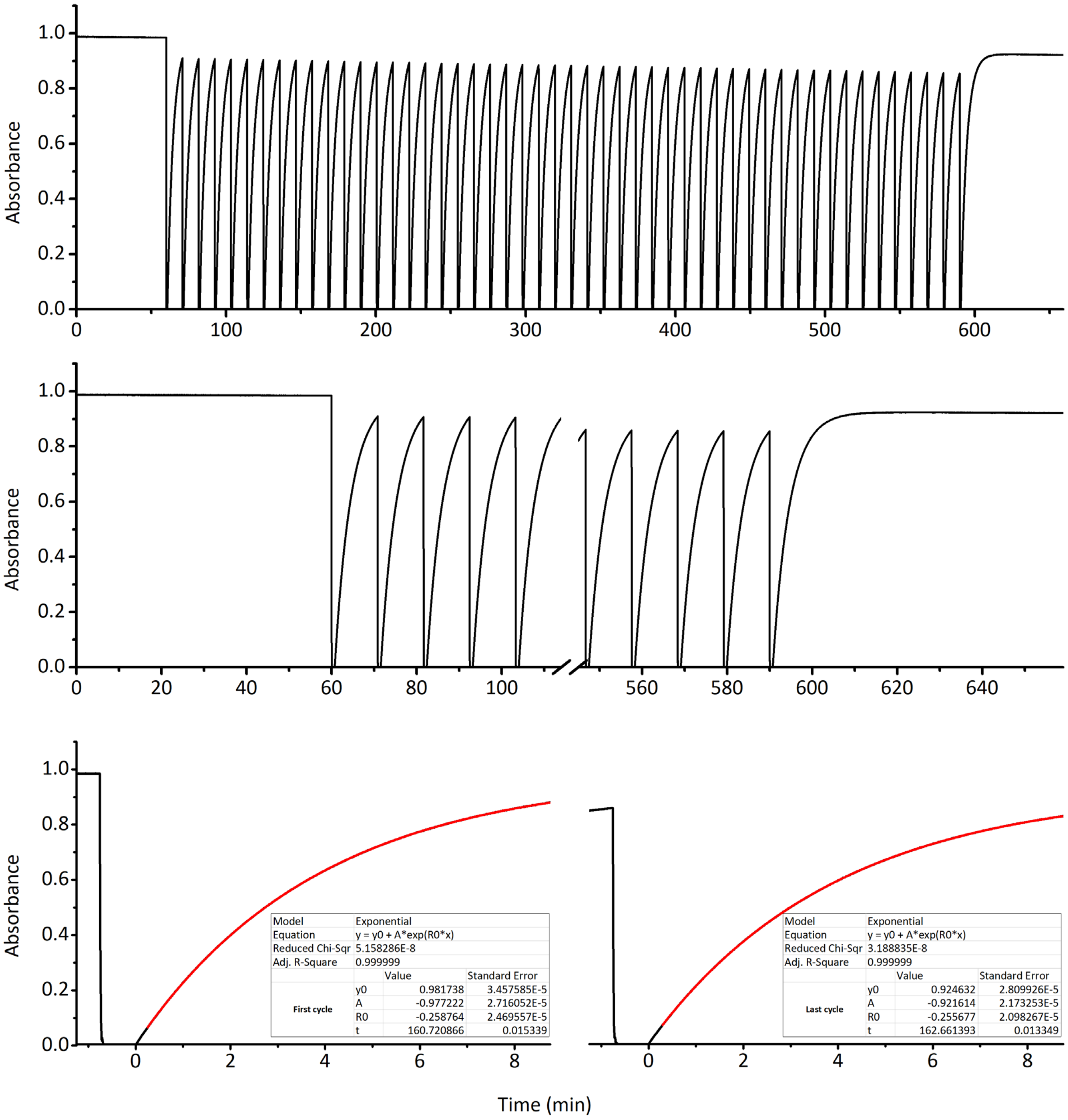

Figure S125. The fatigue resistance of **11** (MeTHF, 298 K) during 50 switching cycles of irradiation by 567 nm light. The absorbance (black lines) was measured at λ_{max} = 566 nm. Top: The complete 50 switching cycles. The first hour of measurement was in the dark and no decomposition was observed. Middle: Expansion of the first 5 and last 5 switching cycles. Bottom: Expansions of the first (left) and last (right) switching cycle with exponential curves (red) fitted to determine the apparent thermal half-life time (table inserts, time in s).

31.12 Fatigue resistance and thermal half-life calculation for 12 in MeTHF

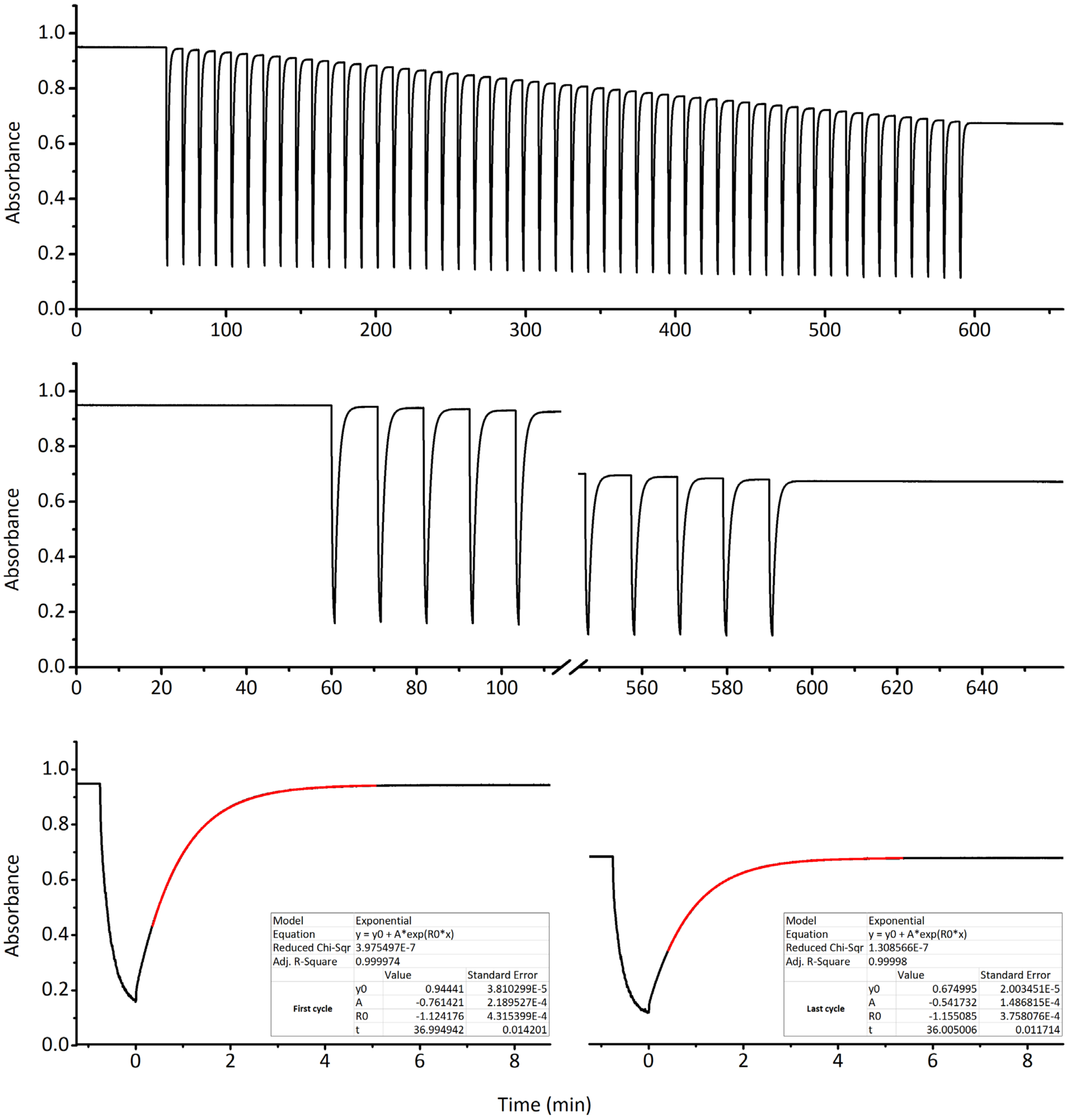

Figure S126. The fatigue resistance of **12** (MeTHF, 298 K) during 50 switching cycles of irradiation by 567 nm light. The absorbance (black lines) was measured at λ_{max} = 567 nm. Top: The complete 50 switching cycles. The first hour of measurement was in the dark and no decomposition was observed. Middle: Expansion of the first 5 and last 5 switching cycles. Bottom: Expansions of the first (left) and last (right) switching cycle with exponential curves (red) fitted to determine the apparent thermal half-life time (table inserts, time in s).

31.13 Fatigue resistance and thermal half-life calculation for 13 in MeTHF

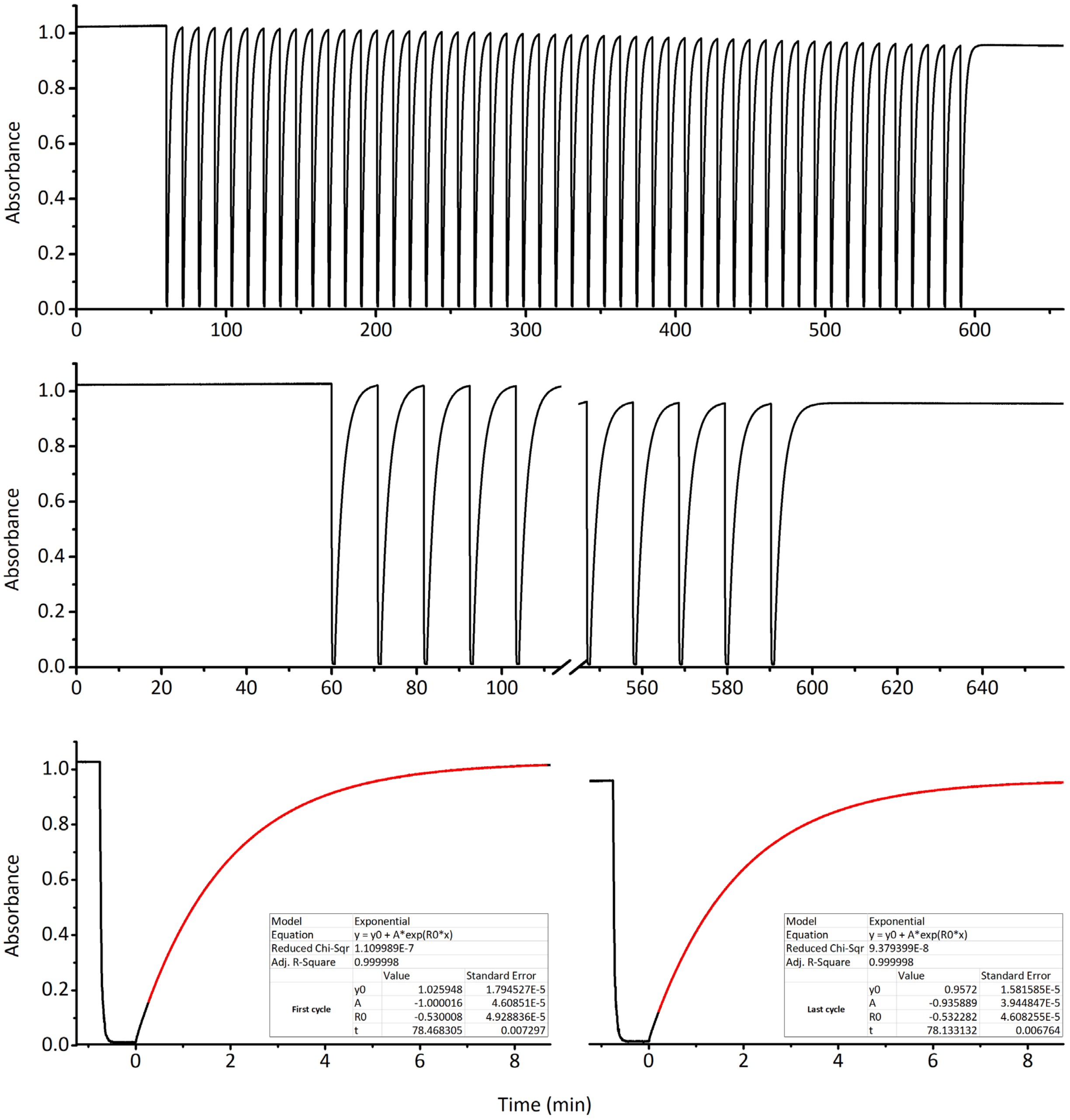

Figure S127. The fatigue resistance of **13** (MeTHF, 298 K) during 50 switching cycles of irradiation by 567 nm light. The absorbance (black lines) was measured at λ_{max} = 563 nm. Top: The complete 50 switching cycles. The first hour of measurement was in the dark and no decomposition was observed. Middle: Expansion of the first 5 and last 5 switching cycles. Bottom: Expansions of the first (left) and last (right) switching cycle with exponential curves (red) fitted to determine the apparent thermal half-life time (table inserts, time in s).

31.14 Fatigue resistance and thermal half-life calculation for 14 in MeTHF

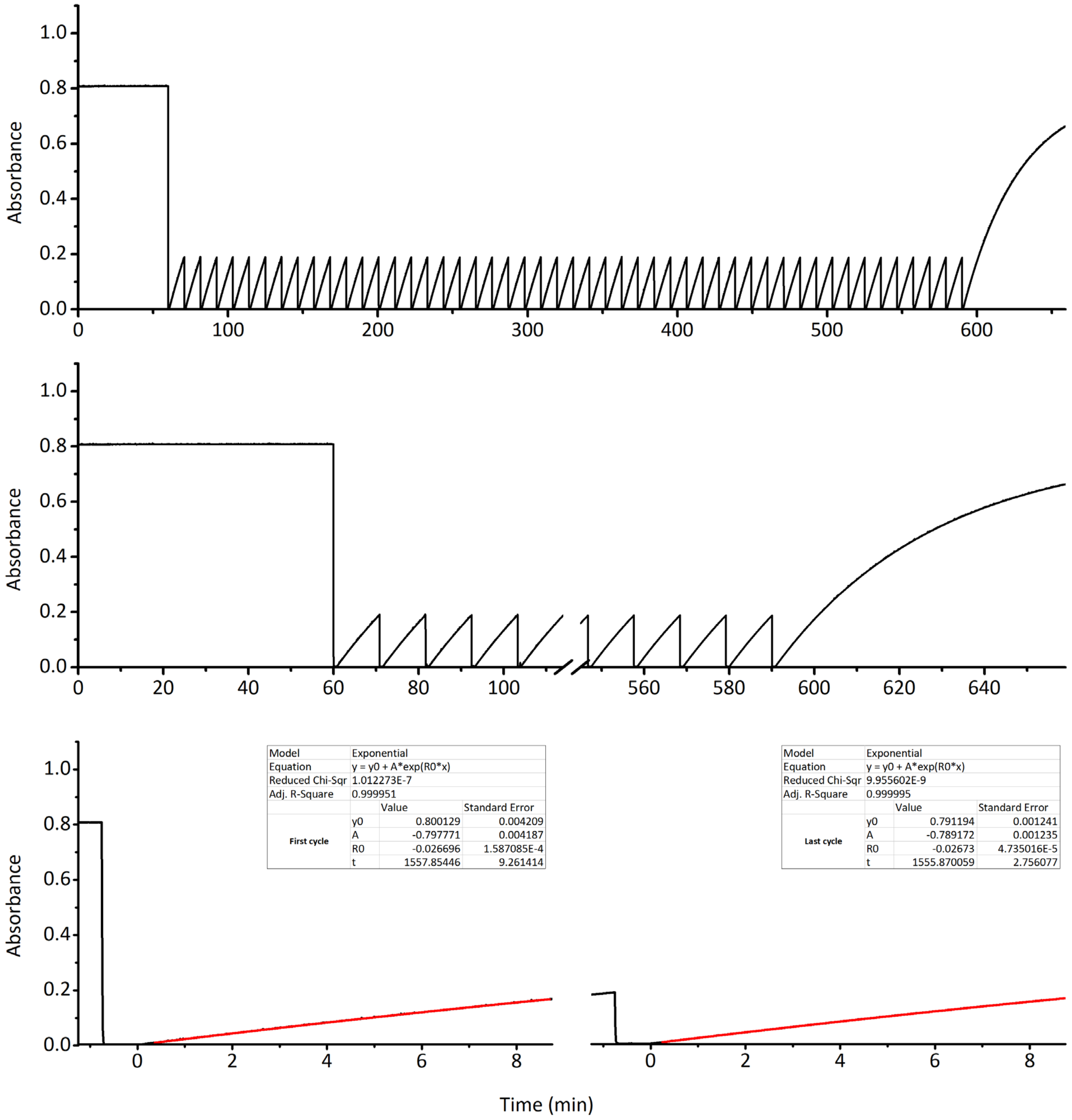

Figure S128. The fatigue resistance of **14** (MeTHF, 298 K) during 50 switching cycles of irradiation by 567 nm light. The absorbance (black lines) was measured at λ_{max} = 583 nm. Top: The complete 50 switching cycles. The first hour of measurement was in the dark and no decomposition was observed. Middle: Expansion of the first 5 and last 5 switching cycles. Bottom: Expansions of the first (left) and last (right) switching cycle with exponential curves (red) fitted to determine the apparent thermal half-life time (table inserts, time in s).

32 X-ray crystallography data

32.1 Single crystal X-ray structure of S1

Pale yellow blocks of **S1** were grown by slow evaporation of a solution of the compound in $CDCl_3$. The crystal of [$C_{11}H_{10}N_2O_4$] with dimensions of 0.1 x 0.1 x 0.1 mm, selected under the polarizing microscope (Leica M165Z), was picked up on a MicroMount (MiTeGen, USA) consisting of a thin polymer tip with a wicking aperture. The X-ray diffraction measurements were carried out on a Bruker kappa-II CCD diffractometer at 150 K using IμS Incoatec Microfocus Source with Mo-K*α* radiation (λ = 0.710723 Å). The single crystal, mounted on the goniometer using a cryo loop for intensity measurements, was coated with immersion oil type NVH and then quickly transferred to the cold nitrogen stream generated by an Oxford Cryostream 700 series. Symmetry related absorption corrections using the program SADABS[7] were applied and the data were corrected for Lorentz and polarisation effects using Bruker APEX3 software.[8] The structure was solved by program SHELXT[9] (with intrinsic phasing) and the full-matrix least-square refinements were carried out using SHELXL-2014[9] through Olex2[10] suite of software. The non-hydrogen atoms were refined anisotropically.

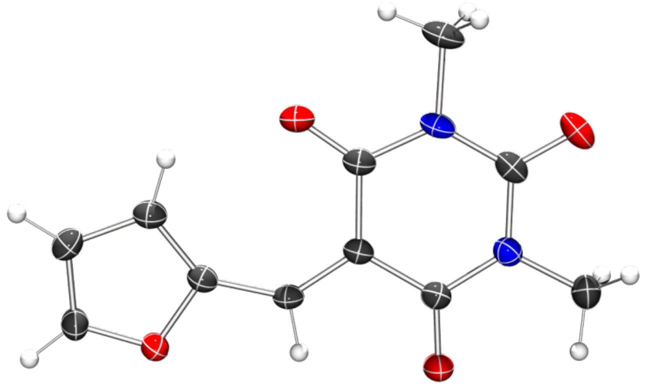

Figure S129. An ORTEP representation of the X-ray crystal structure of **S1**. Thermal ellipsoids are drawn at 50% probability.

32.2 Single crystal X-ray structure of 1b

Colourless blocks of **1b** were grown by slow evaporation of a solution of the compound in MeCN. The crystal of **1b** with dimensions of 0.2 x 0.2 x 0.05 mm, selected under the polarizing microscope (Leica M165Z), was picked up on a MicroMount (MiTeGen, USA) consisting of a thin polymer tip with a wicking aperture. The X-ray diffraction measurements were carried out on a Bruker kappa-II CCD diffractometer at 150 K using IμS Incoatec Microfocus Source with Mo-Kα radiation (λ = 0.710723 Å). The single crystal, mounted on the goniometer using a cryo loop for intensity measurements, was coated with immersion oil type NVH and then quickly transferred to the cold nitrogen stream generated by an Oxford Cryostream 700 series. Symmetry related absorption corrections using the program SADABS[7] were applied and the data were corrected for Lorentz and polarisation effects using Bruker APEX3 software.[8] The structure was solved by program SHELXT[9] (with intrinsic phasing) and the full-matrix least-square refinements were carried out using SHELXL-2014[9] through Olex2[10] suite of software. The non-hydrogen atoms were refined anisotropically.

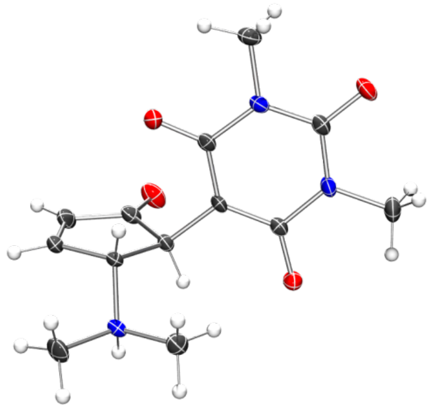

Figure S130. An ORTEP representation of the X-ray crystal structure of **1b**. Thermal ellipsoids are drawn at 50% probability.

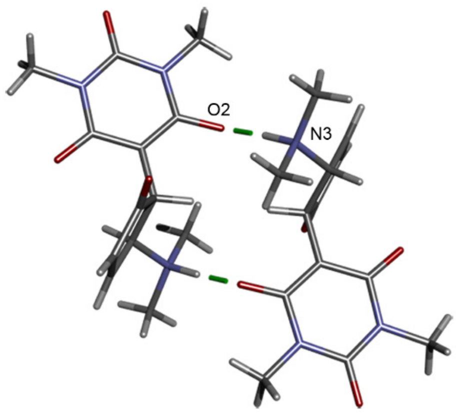

Figure S131. Molecules of **1b** form dimers via short intermolecular hydrogen bonds between the amine proton and the amide oxygen of an adjacent molecule. Hydrogen bonding between amine proton and oxygen of amide groups N3···O2 = 2.647(1) Å.

32.3 Single crystal X-ray structure of 2b·2H_2O

Colorless plates of **2b** were grown by slow evaporation of a solution of the compound in deuterated chloroform. The crystal of [**2b**·2(H_2O)] with dimensions 0.05 x 0.05 x 0.01 mm was coated in Paratone and transferred to the goniometer under a cold stream of 100 K. Diffraction measurements were carried out using Si<111> monochromated synchrotron X-ray radiation (λ = 0.71073 Å) on the MX1 Beamline at the Australian Synchrotron.[11] Data collection was carried out using Australian Synchrotron QEGUI software and unit cell refinement, data reduction and processing were carried out with XDS.[12] The structure was solved using dual space methods with SHELXT.[9] The least-squares refinement was carried out with SHELXL-2014[9] through Olex2[10] suite of software. The non-hydrogen atoms were refined anisotropically. The disorder in the ethyl group was modelled over two positions with occupancies of 0.63 and 0.37. Two solvent water molecules could be identified from the difference map and were included in the refinement.

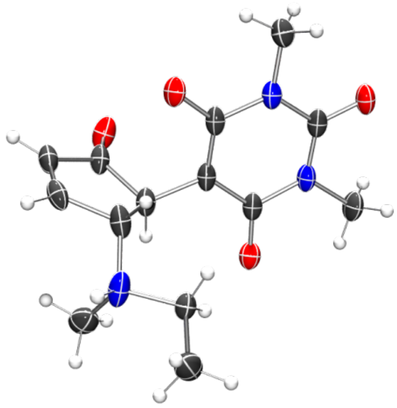

Figure S132 Top: an ORTEP representation of **2b** in the X-ray crystal structure of **2b**·2(H_2O). Thermal ellipsoids are drawn at 50% probability.

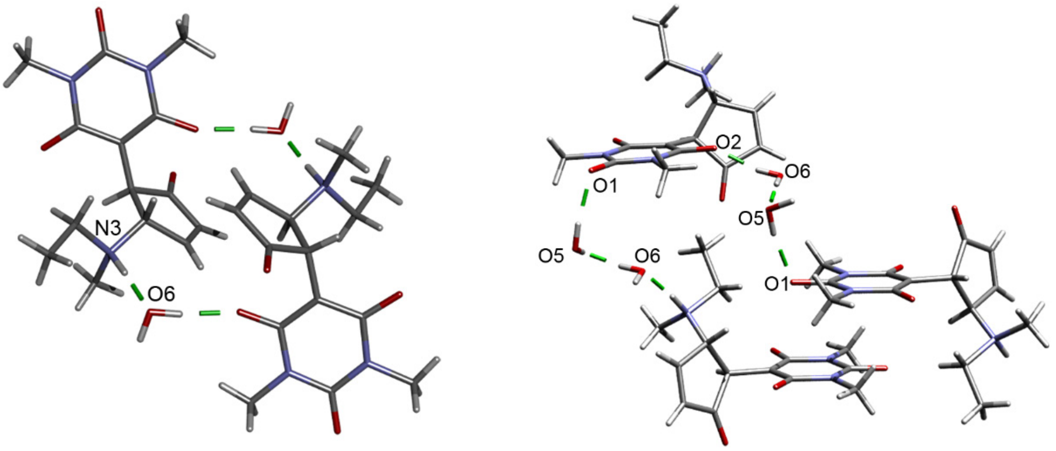

Figure S133. Representations of the intermolecular hydrogen bonds involved in the crystal packing of **2b**. The molecules form dimers (left) or trimers (right). Hydrogen bonding between amine proton and oxygen atom of a water molecule N3···O6 = 2.701(3) Å; hydrogen bonding between amide oxygen and water molecule O1···O5 = 2.811(2) Å, O2···O6 = 2.680(3) Å.

32.4 Single crystal X-ray structure of 4a·THF

Purple plate-like crystals of **4a** were grown from a saturated solution of the compound in THF/diethyl ether. The crystal of [**4a**·THF] with dimensions of 0.38 x 0.23 x 0.05 mm, selected under the polarizing microscope (Leica M165Z), was picked up on a MicroMount (MiTeGen, USA) consisting of a thin polymer tip with a wicking aperture. The X-ray diffraction measurements were carried out on a Bruker kappa-II CCD diffractometer at 150 K using IμS Incoatec Microfocus Source with Mo-Kα radiation (λ = 0.710723 Å). The single crystal, mounted on the goniometer using a cryo loop for intensity measurements, was coated with immersion oil type NVH and then quickly transferred to the cold nitrogen stream generated by an Oxford Cryostream 700 series. Symmetry related absorption corrections using the program SADABS[7] were applied and the data were corrected for Lorentz and polarisation effects using Bruker APEX3 software.[8] The structure was solved by program SHELXT[9] (with intrinsic phasing) and the full-matrix least-square refinements were carried out using SHELXL-2014[9] through Olex2[10] suite of software. The non-hydrogen atoms were refined anisotropically. One molecule of solvent tetrahydrofuran could be identified from the difference map and was included in the least-squares refinement.

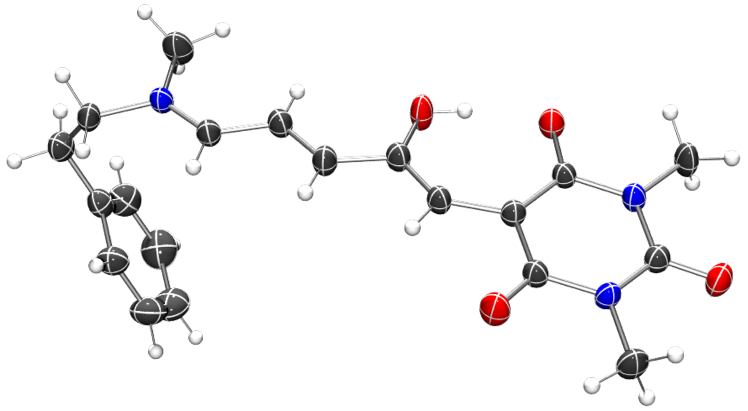

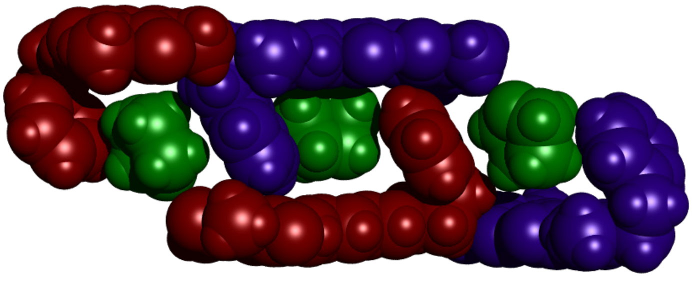

Figure S134 Top: an ORTEP representation of **4a** in the X-ray crystal structure of [$C_{20}H_{23}N_3O_4 \cdot C_4H_7O$]. Thermal ellipsoids are drawn at 50% probability. Bottom: a CPK representation of the crystal packing of **4a** (alternating red and blue) and solvent THF (green) showing the role of solvent in the lattice.

32.5 Single crystal X-ray structure of 2{9b}·7H_2O

Colorless block-like crystals of **9b** were grown by slow evaporation of an acetonitrile solution. The crystal of [2(**9b**)·7(H_2O)] with dimensions of 0.27 x 0.19 x 0.11 mm, selected under the polarizing microscope (Leica M165Z), was picked up on a MicroMount (MiTeGen, USA) consisting of a thin polymer tip with a wicking aperture. The X-ray diffraction measurements were carried out on a Bruker kappa-II CCD diffractometer at 150 K using IμS Incoatec Microfocus Source with Mo-Kα radiation (λ = 0.710723 Å). The single crystal, mounted on the goniometer using a cryo loop for intensity measurements, was coated with immersion oil type NVH and then quickly transferred to the cold nitrogen stream generated by an Oxford Cryostream 700 series. Symmetry related absorption corrections using the program SADABS[7] were applied and the data were corrected for Lorentz and polarisation effects using Bruker APEX3 software.[8] The structure was solved by program SHELXT[9] (with intrinsic phasing) and the full-matrix least-square refinements were carried out using SHELXL-2014[9] through Olex2[10] suite of software. The non-hydrogen atoms were refined anisotropically. Seven molecules of solvent water could be identified from the difference map and were included in the least-squares refinement.

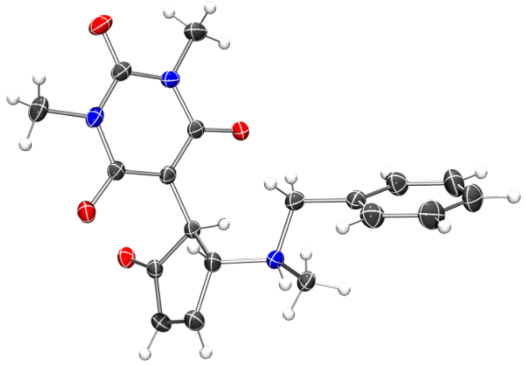

Figure S135. An ORTEP representation of **9b** in the X-ray crystal structure of [2($C_{19}H_{21}N_3O_4$)·7(H_2O)]. Thermal ellipsoids are drawn at 50% probability.

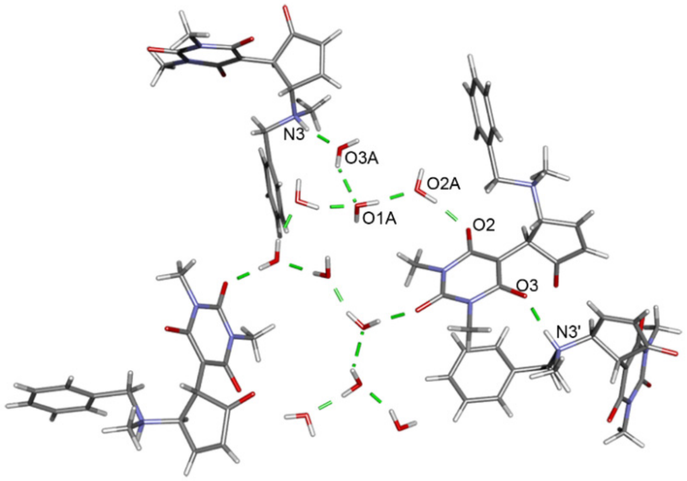

Figure S136. Representations of the intermolecular hydrogen bonds involved in the crystal packing. The two **9b** molecules in the asymmetric unit form hydrogen bonds from the amine proton to either a water molecule or an amide oxygen. Water molecules form a channel of solvent in the lattice. Hydrogen bonding between amine and amide oxygen **9b** N3'···O3 = 2.766(3) Å; hydrogen bonding between amine and water molecule **9b** N3···O3A = 2.717(4) Å; hydrogen bonding between bridging water molecules O3A···O1A = 2.878(4) Å, O1A···O2A = 2.711(5) Å.

32.6 Single crystal X-ray structure of 12a

Dark purple blocks of **12a** were grown by slow diffusion of diethyl ether into a solution of the compound in acetonitrile. The crystal of [$C_{15}H_{19}N_3O_4$] with dimensions of 0.12 x 0.07 x 0.05 mm was coated in immersion oil type OVH and transferred to the goniometer under a cold stream of 100 K. Diffraction measurements were carried out using Si<111> monochromated synchrotron X-ray radiation (λ = 0.71073 Å) on the MX1 Beamline at the Australian Synchrotron.[11] Data collection was carried out using Australian Synchrotron QEGUI software and unit cell refinement, data reduction and processing were carried out with XDS.[12] The structure was solved using dual space methods with SHELXT.[9] The least-squares refinement was carried out with SHELXL-2014[9] through Olex2[10] suite of software. The non-hydrogen atoms were refined anisotropically.

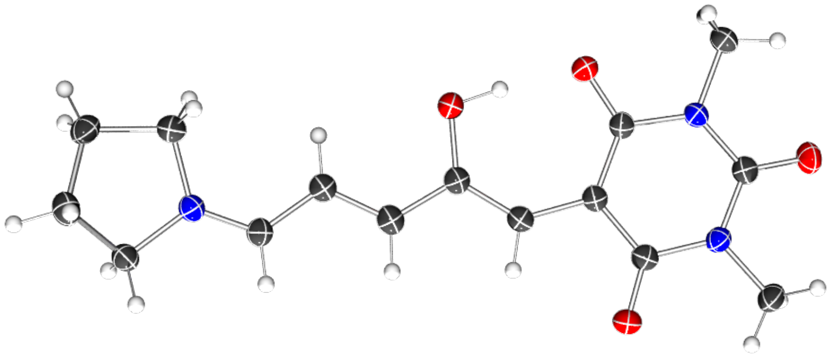

Figure S137. An ORTEP representation of the X-ray crystal structure of **12a**. Thermal ellipsoids are drawn at 50% probability.

32.7 Single crystal X-ray structure of 2{12b}·DCM·1.1H$_2$O

Colorless plates of [2(**12b**)CH_2Cl_2·1.1H_2O] were grown by slow diffusion of dichloromethane into a solution of **12b** in acetonitrile. The crystal of [2($C_{15}H_{19}N_3O_4$)·CH_2Cl_2·1.1H_2O] with dimensions of 0.01 x 0.2 x 0.9 mm was coated in immersion oil type OVH and transferred to the goniometer under a cold stream of 100 K. Diffraction measurements were carried out using Si<111> monochromated synchrotron X-ray radiation (λ = 0.71073 Å) on the MX1 Beamline at the Australian Synchrotron.[11] Data collection was carried out using Australian Synchrotron QEGUI software and unit cell refinement, data reduction and processing were carried out with XDS.[12] The structure was solved using dual space methods with SHELXT.[9] The least-squares refinement was carried out with SHELXL-2014[9] through Olex2[10] suite of software. The non-hydrogen atoms were refined anisotropically. One disordered solvent dichloromethane molecule could be identified from the difference map and was modelled over two positions with occupancies of 0.5 per site. Two partial occupancy solvent water molecules could be identified from the difference map and were modelled with occupancies of 0.46 and 0.64 respectively.

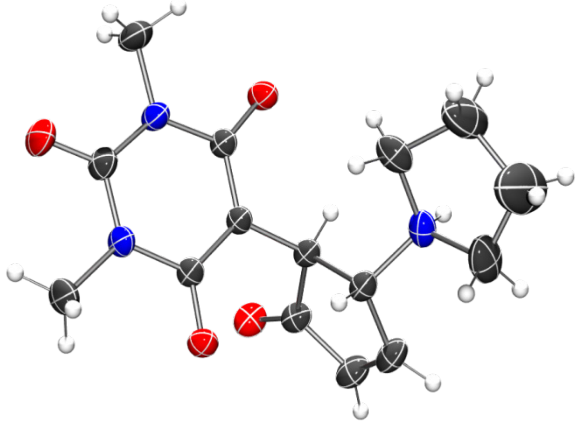

Figure S138 An ORTEP representation of **12b** in the X-ray crystal structure of [2(**12b**)·CH_2Cl_2·1.1H_2O]. Thermal ellipsoids are drawn at 50% probability.

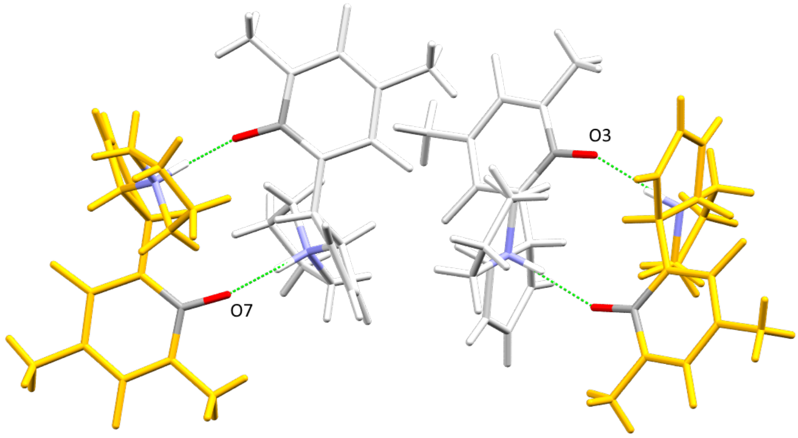

Figure S139 Representation of the intermolecular hydrogen bonding involved in the crystal packing of **12b**. The molecules form dimers between the *R*-enantiomer (orange) and the *S*-enantiomer (grey). Hydrogen bonding between amine and amide oxygen N6···O7 = 2.642(3) Å, N3···O3 = 2.701(4) Å.

32.8 Single crystal X-ray structure of 14a·$CDCl_3$

Dark blue needles of **14a** were grown by slow evaporation of a solution of the compound in deuterated chloroform. The crystal of [$C_{19}H_{21}N_3O_5 \cdot CDCl_3$] with dimensions 0.1 x 0.02 x 0.01 mm was coated in Paratone and transferred to the goniometer under a cold stream of 100 K. Diffraction measurements were carried out using Si<111> monochromated synchrotron X-ray radiation (λ = 0.71073 Å) on the MX1 Beamline at the Australian Synchrotron.[11] Data collection was carried out using Australian Synchrotron QEGUI software and unit cell refinement, data reduction and processing were carried out with XDS.[12] The structure was solved using dual space methods with SHELXT.[9] The least-squares refinement was carried out with SHELXL-2014[9] through Olex2[10] software. The crystal system was verified as monoclinic using PLATON's Addsym[13] function which suggested no missing higher symmetry. The non-hydrogen atoms were refined anisotropically. One molecule of deuterated chloroform could be identified and was included in the refinement.

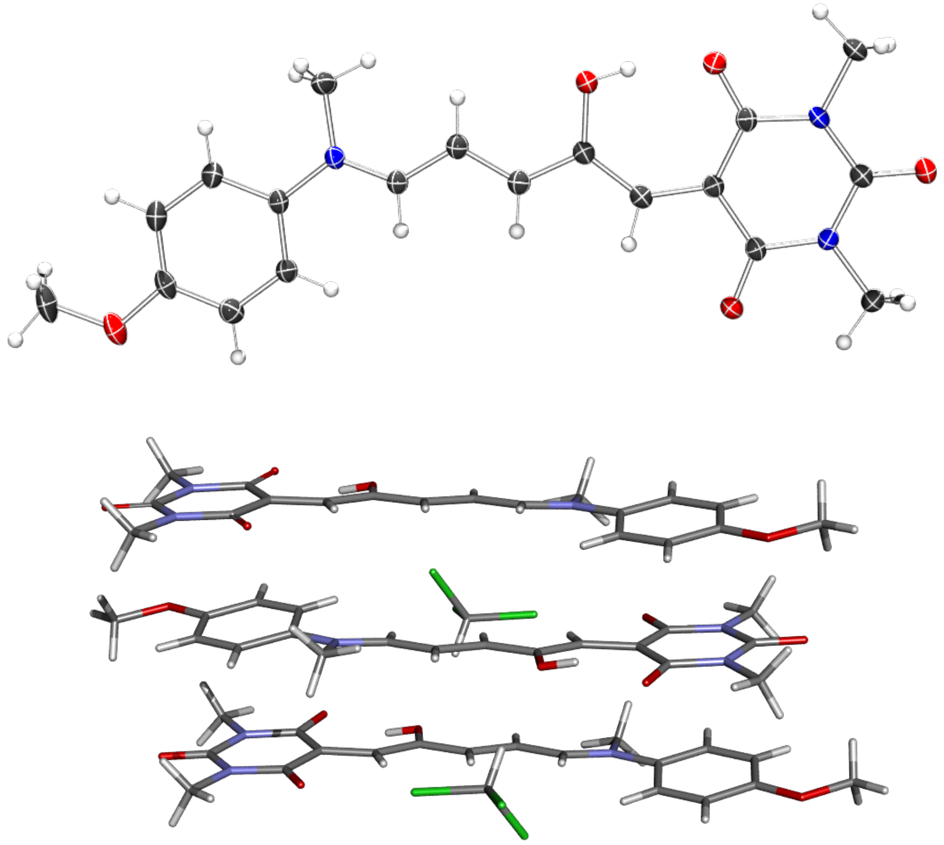

Figure S140. Top: an ORTEP representation of **14a** in the X-ray crystal structure of [$C_{19}H_{21}N_3O_4 \cdot CDCl_3$]. Thermal ellipsoids are drawn at 50% probability. Bottom: representation of the crystal packing of **14a** and solvent $CDCl_3$. The donor and acceptor groups alternate positions as the molecules stack along the *a* axis. The separations between the least-squares plane of the phenyl ring to the centroid of the barbituric acid ring 3.350 Å.

32.9 Single crystal X-ray structure of 14b'

Colorless plates of **14b'** were grown from a hexane/dichloromethane solution of **14b'**. The crystal of [$C_{19}H_{22}N_3O_5$] with dimensions 0.6 x 0.3 x 0.1 mm was coated in Paratone and transferred to the goniometer under a cold stream of 100 K. Diffraction measurements were carried out using Si<111> monochromated synchrotron X-ray radiation (λ = 0.71073 Å) on the MX1 Beamline at the Australian Synchrotron.[11] Data collection was carried out using Australian Synchrotron QEGUI software and unit cell refinement, data reduction and processing were carried out with XDS.[12] The structure was solved using dual space methods with SHELXT.[9] The least-squares refinement was carried out with SHELXL-2014[9] through Olex2[10] software. The non-hydrogen atoms were refined anisotropically.

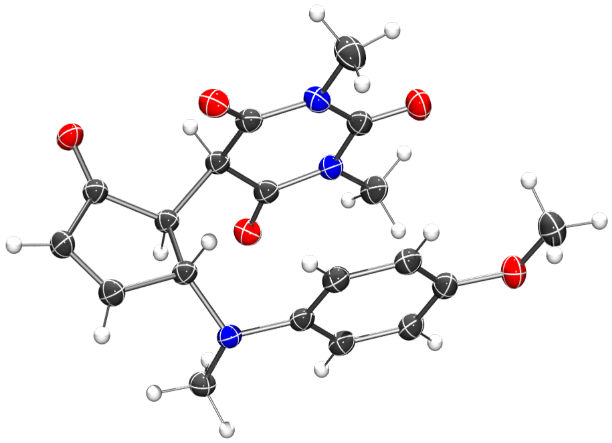

Figure S141. An ORTEP representation of the X-ray crystal structure of **14b'**. Thermal ellipsoids are drawn at 50% probability.

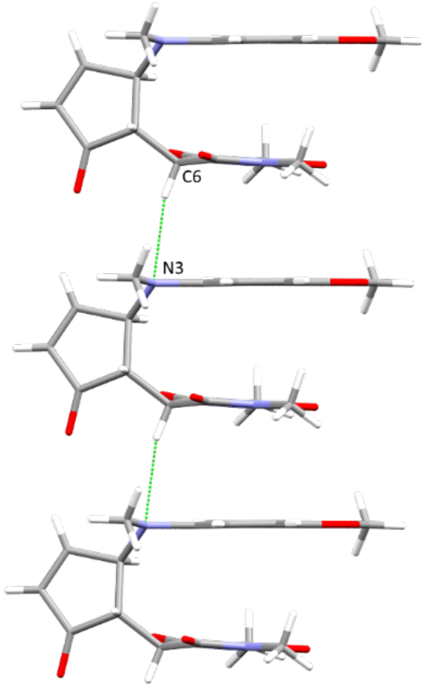

Figure S142. Intermolecular π- π interactions between adjacent molecules of **14b'** along the crystallographic *a*-axis in the solid state structure of **14b'**. The intramolecular separation between the least-squares plane of the phenyl ring to the centroid of the barbituric acid ring is 3.363 Å and the intermolecular separation between the least-squares plane of a neighbouring phenyl ring and the centroid of the barbituric acid ring is 3.318 Å. C-H···N interaction between C6 and N3 = 3.528(2) Å.

32.10 Comparison of X-ray structure data

32.10.1 Comparison of X-ray data of linear structures

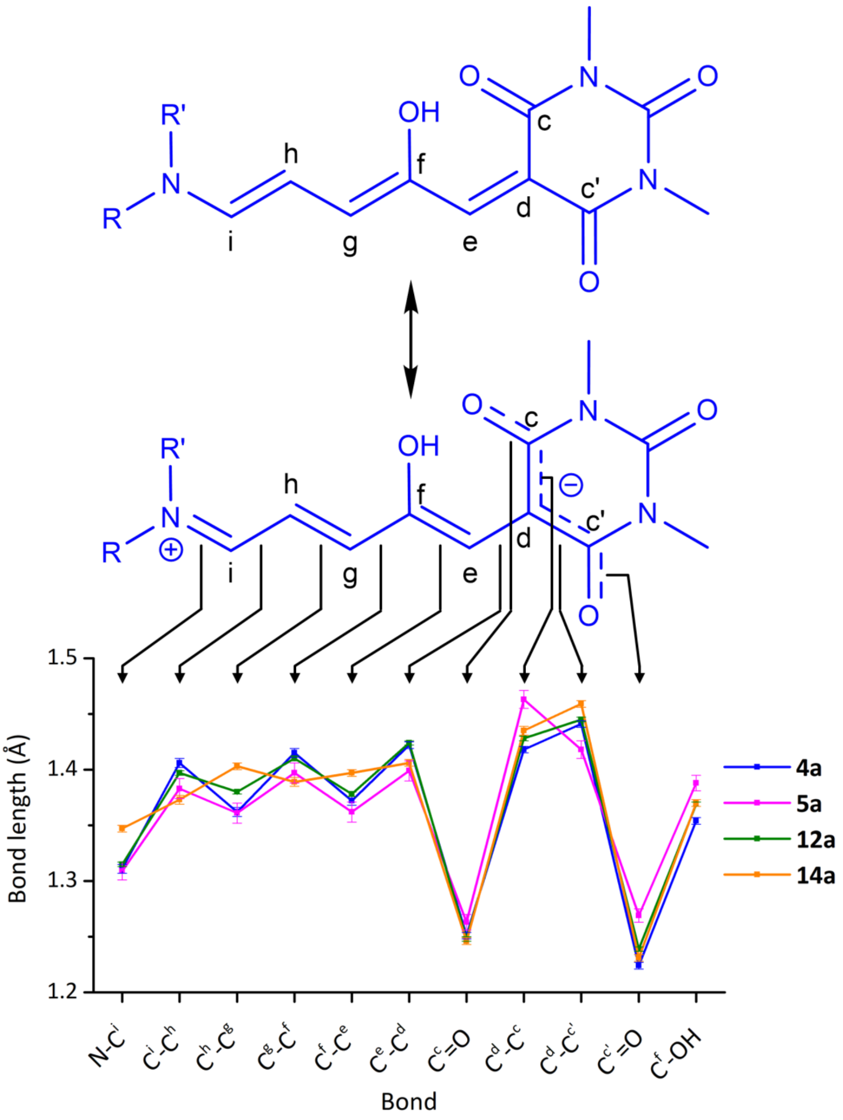

Figure S143. Comparisons of bond lengths between the linear isomers. Error bars represent estimated standard deviations (ESD) values. Resonance forms of linear DASA isomers are shown above; bond lengths along the triene chain are most consistent with the bottom structure. Structure **5a** was reported by Read de Alaniz and co-workers.[14]

Table S8. Selected bond lengths of linear isomers from single crystal X-ray structures.

Bond	**4a**	**5a**^{a}	**12a**	**14a**
N-C^{i}	1.311	1.309	1.315	1.347
C^{i}-C^{h}	1.406	1.383	1.397	1.373
C^{h}-C^{g}	1.362	1.361	1.380	1.403
C^{g}-C^{f}	1.415	1.397	1.410	1.389
C^{f}-C^{e}	1.372	1.362	1.378	1.397
C^{e}-C^{d}	1.422	1.399	1.424	1.406
C^{d}-C^{c}	1.418	1.463	1.428	1.435
C^{c}=O	1.251	1.263	1.248	1.246
C^{d}-$C^{c'}$	1.441	1.418	1.445	1.459
$C^{c'}$=O	1.224	1.269	1.239	1.231
C^{f}-OH	1.354	1.388	1.369	1.370

^{a} Structure **5a** was reported by Read de Alaniz and co-workers.[14]

32.10.2 Comparison of X-ray data of cyclic structures

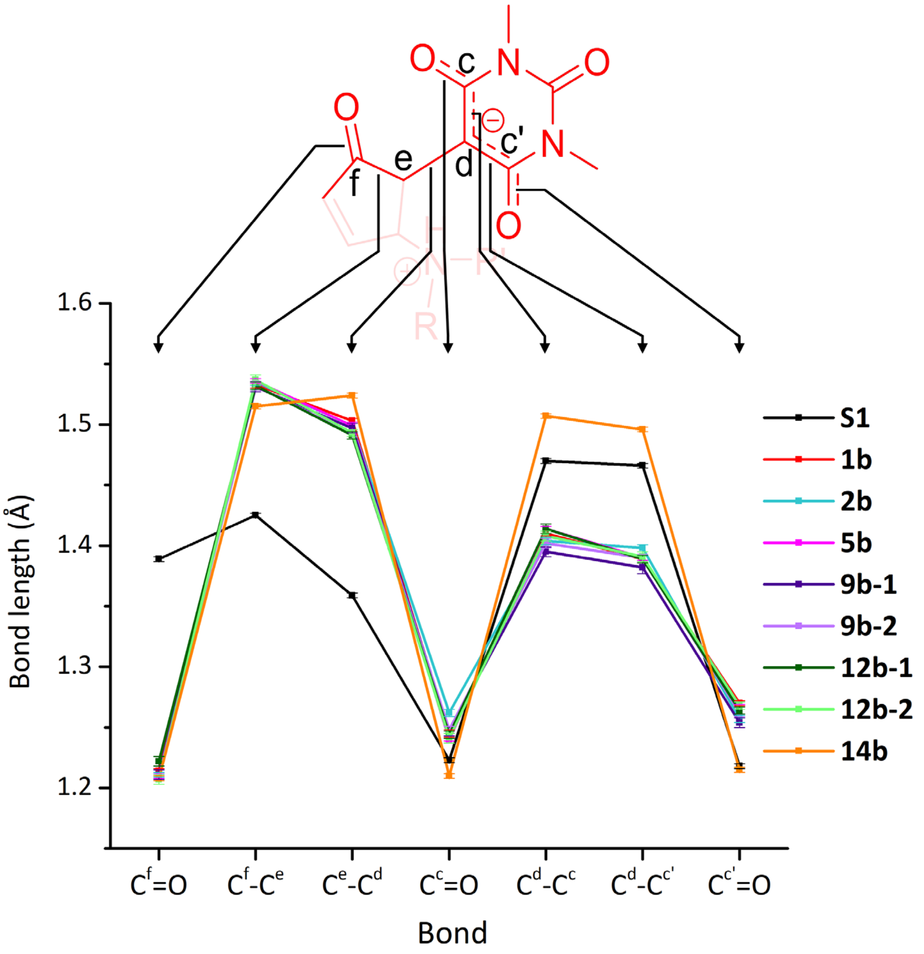

Figure S144. Comparison of bond lengths between the cyclic isomers, and starting material **S1**. Structures of **9b** and **12b** have two molecules each in the crystallographic asymmetric unit. Error bars represent estimated standard deviations (ESD) values. Structure **5b** was reported by Read de Alaniz and co-workers.[14]

Table S9. Selected bond lengths of cyclic isomers (Å)

Bond	S1	1b	2b	5b[a]	9b		12b		14b'
					(1/2)[b]	(2/2)[b]	(1/2)[b]	(2/2)[b]	
$C^{c'}$=O	1.218	1.27	1.257	1.267	1.254	1.262	1.264	1.268	1.215
$C^{c'}$-C^d	1.466	1.389	1.398	1.390	1.382	1.390	1.389	1.391	1.496
C^d-C^c	1.470	1.410	1.404	1.414	1.395	1.402	1.414	1.408	1.507
C^c=O	1.223	1.245	1.262	1.240	1.247	1.248	1.244	1.241	1.21
C^d-C^e	1.359	1.503	1.499	1.499	1.497	1.492	1.491	1.493	1.524
C^e-C^f	1.425	1.533	1.535	1.536	1.531	1.532	1.532	1.537	1.515
C^f=O	1.389	1.214	1.211	1.210	1.211	1.209	1.222	1.207	1.208
C'=O	1.218	1.27	1.257	1.267	1.254	1.262	1.264	1.268	1.215

[a] Structure **5b** was reported by Read de Alaniz and co-workers.[14]
[b] Structures of **9b** and **12b** have two non-equivalent molecules in the asymmetric unit.

33 Computational studies

33.1 Computational details

All electronic structure calculations were carried out using Gaussian16.[15] The molecular geometries were optimized at the M06-2X/6-31+G(d) level of theory[16] in conjunction with the SMD implicit solvent model[17] to simulate chloroform, and frequency calculations were carried out to confirm that these are indeed minimum energy structures or first order saddle points (transition states). Furthermore, intrinsic reaction coordinate (IRC) simulation were also carried out to confirm that these are the correct transition states that connect the reactants and products. Systematic conformer searches were carried out by scanning along selected rotatable bonds at 120° or 180° resolution (see below for example).

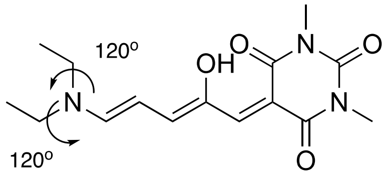

Figure S145. Structure of isomers to be modelled by DFT.

Single-point calculations using the M06-2X/6-311+G(3df,2p) and SMD model was performed on the M06-2X/6-31+G(d) optimized geometries and combined with corresponding thermal corrections based on the rigid rotor harmonic oscillator approximation to yield the final solution phase Gibbs free energies. All free energies changes reported in this work refer to energies and thermal corrections that are directly computed within the solvent reaction field. As detailed in a recent publication,[18] this approach gives comparable if not slightly better performance compared to corresponding thermodynamic-cycle based approaches. Gas phase basicity of amines in Table S12 were computed at the G3(MP2)-RAD[19] level of theory on M06-02X/6-31G(d) optimized geometries and thermal corrections.

33.2 DFT relative energies calculations for DASAs 1, 2, 5, 8 and 14.

Table S10. Relative calculated Gibbs free energies (kJ/mol at 298 K) of conformer **a** and cyclization **TS** for selected DASAs in chloroform.[a]

DASA	R	R'	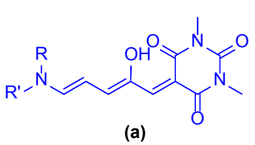 (a)	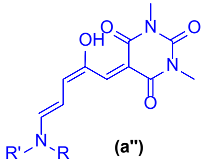 (a'')	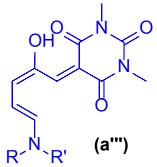 (a''')	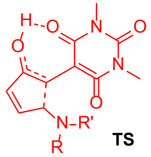 TS	**Energy difference of model imine from trigonal planar to pyramidal**
**1**	Me	Me	0.0	14.8	36.5	95.4	6.1
**2**	Me	Et	0.0	21.2	35.9	97.3	5.3
**5**	Et	Et	0.0	24.0	39.0	102.7	1.7
**8**	iBu	iBu	0.0	19.8	37.4	103.3	1.0
**14**	Me	C_6H_4OMe	0.0	18.8	38.9	91.5	-

[a] Computational details given in Section 33.1 above.

33.3 DFT Geometries and Relative Energies ΔE (M06-2X/6-31+G(d) in kJ mol^{-1}) of cyclic conformers of DASA 14

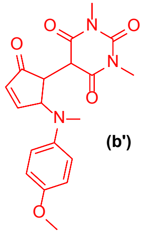

(b')

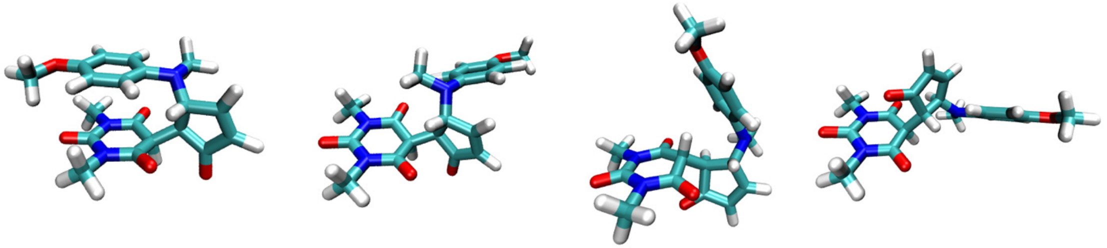

0.0 35.6 25.0 41.3

33.4 Gas Phase basicity values

Table S11. Gas phase basicity values for amines used to prepare DASAs **1**–**14**.[a]

DASA	Amine	Gas phase basicity[a]
**1**	NMe_2	214.9 (214.3)[20]
**2**	N(Me)Et	217.8
**3**	N(Me)Bu	220.6
**4**	$N(Me)CH_2Bz$	222.2
**5**	$N(Et)_2$	219.9 (221.2)[21]
**6**	$N(Pr)_2$	221.9 (223.2)[21]
**7**	$N(Oct)_2$	-
**8**	N(*i*-Bu)$_2$	224.3 (224.8)[21]
**9**	N(Me)Bz	219.7
**10**	N(Et)Bz	222.5
**11**	Q	218.9
**12**	Pyr	219.4 (218.8)[22]
**13**	Pip	220.4 (220.0)[23]
**14**	N(Me)(PhOMe)	215.4

[a] calculated gas phase basicities, and experimental values (in parenthesis)

33.5 Gaussian archives for M06-2X/6-31+G(d)+SMD(chloroform) optimized geometries

33.5.1 1a

1\1\GINC-R42\FOpt\RM062X\Gen\C13H17N3O4\ROOT\07-May-2018\0\\#m062X/gen
6D OPT freq=noraman INT(grid=ultrafine) SCRF=(SMD,Solvent=chloroform)
\\1.smd\\0,1\N,1.6697476631,2.6540006241,-0.1472035273\C,0.4357465281,
2.018951088,-0.0903517663\C,0.4220466161,0.5912443804,0.0816006181\C,1
.681683511,-0.1321182428,0.1892556296\N,2.8546598945,0.6321842903,0.11
59767433\C,2.8904602792,2.0014759737,-0.0489901026\O,-0.5727563793,2.7
421402207,-0.1935751428\O,3.9541311334,2.6019903326,-0.104676494\O,1.7
636431667,-1.3501734752,0.3375674803\C,1.6649235756,4.1065958586,-0.32
25050204\C,4.1543799608,-0.0302102281,0.2165858283\C,-0.7346809628,-0.
2083735992,0.1597205443\C,-2.1045635678,0.0467500739,0.1062140568\C,-2
.9702888617,-1.050174102,0.2254210226\H,-0.5002790101,-1.2615748935,0.
2913715319\O,-2.691605467,1.2604663567,-0.0512951447\H,-2.5146725882,-
2.0296741476,0.3519864429\H,-1.9739577832,1.9452101556,-0.1215919597\C
,-4.3597249823,-0.953595717,0.1908257798\C,-5.13113417,-2.1082888217,0
.3183791293\N,-6.4522849681,-2.1577332335,0.302703491\C,-7.2393115789,
-0.9413126578,0.1418989382\C,-7.1808204096,-3.411300208,0.4421800528\H
,-4.8224124563,0.0194054807,0.0649328459\H,-4.6300451137,-3.0672912901
,0.4430590059\H,-7.0303616644,-0.242920291,0.9587416117\H,-8.297217755
3,-1.2031303512,0.1570671954\H,-7.0023141639,-0.4587664324,-0.81185911
16\H,-6.475762668,-4.235890141,0.5547325209\H,-7.797562779,-3.58142486
64,-0.4457546367\H,-7.8285703095,-3.3665166949,1.3231570583\H,4.706458
7757,0.3631489113,1.0726738964\H,3.9752445451,-1.0946486173,0.34330709
33\H,4.7337356036,0.1484910213,-0.6916334306\H,2.6964068741,4.44782023
3,-0.3471586158\H,1.1614119027,4.3626139284,-1.2567553883\H,1.13312160
92,4.5754520815,0.507695824\\Version=ES64L-G16RevA.03\State=1-A\HF=-97
0.4497285\RMSD=9.351e-09\RMSF=5.960e-06\Dipole=-5.7463189,-2.9260657,0
.2615114\Quadrupole=24.3407435,-6.0384196,-18.3023239,25.183755,-2.347
6514,-1.088624\PG=C01 [X(C13H17N3O4)]\\@

33.5.2 1a''

1\1\GINC-R4431\FOpt\RM062X\Gen\C13H17N3O4\ROOT\05-May-2018\0\\#m062X/g
en 6D OPT freq=noraman INT(grid=ultrafine) SCRF=(SMD,Solvent=chlorofor
m)\\1a.smd.chloroform.smd\\0,1\N,-0.5610462004,1.9254136677,-0.0315118
978\C,-1.8410170738,1.3783067084,-0.022550321\C,-1.9451842492,-0.05896
11323,-0.015354371\C,-0.7364722317,-0.8757061371,-0.0177253555\N,0.485
5935243,-0.1927790126,-0.0270692797\C,0.60679515,1.1806786579,-0.03405
25939\O,1.7017647171,1.7252178328,-0.0421218144\O,-0.7393782037,-2.105
9357021,-0.0119801743\O,-2.808194288,2.1597939177,-0.0212002988\C,-3.1
488255513,-0.7905822601,-0.0058913734\C,-4.5025290813,-0.4656714171,-0
.0005655592\C,-5.4779878325,-1.4794937818,0.0090540296\C,-5.3353923138
,-2.8674267685,0.0148845206\C,-6.4874854792,-3.6505135959,0.0241030747
\N,-6.5300874205,-4.9732185994,0.030498355\O,-5.0179473948,0.797918616
4,-0.0038917017\C,-0.3857437151,3.3783274059,-0.0390945746\C,1.7390051
717,-0.9449711312,-0.0299560554\H,-2.9604321613,-1.8596577533,-0.00187
88573\H,-6.5008784898,-1.0990329478,0.0123095454\H,-4.3591434534,-3.34
06044327,0.0123219805\H,-7.4578529939,-3.1577686061,0.0264620103\C,-5.
3047786185,-5.7619995997,0.0283783617\C,-7.7955273004,-5.6936014181,0.
0395652128\H,-4.2624257601,1.4402134153,-0.0110896818\H,-4.7051266015,
-5.537351055,0.91664341\H,-4.7139672709,-5.5463277983,-0.8680070147\H,
-5.5686469489,-6.8197141882,0.0350076983\H,-8.6203708139,-4.9794764329
,0.0398970466\H,-7.8600536792,-6.3212303643,0.9339195829\H,-7.86803973
14,-6.3295753697,-0.848259955\H,2.317555857,-0.6993283479,-0.922972739
5\H,2.3266504695,-0.690987805,0.8547487892\H,1.4913025929,-2.003250882
9,-0.0237268507\H,0.1750526434,3.6866023708,0.8455034127\H,0.165540568
7,3.6784969795,-0.9324221033\H,-1.371137836,3.8356649677,-0.0359164571
\\Version=ES64L-G16RevA.03\State=1-A\HF=-970.4409848\RMSD=7.859e-09\RM
SF=8.682e-06\Dipole=-3.780393,-4.7679085,0.0405615\Quadrupole=3.115973
2,11.9834315,-15.0994047,28.7564296,-0.2204127,-0.2706502\PG=C01 [X(C1
3H17N3O4)]\\@

33.5.3 1a'''

1\1\GINC-R3945\FOpt\RM062X\Gen\C13H17N3O4\ROOT\05-May-2018\0\\#m062X/g
en 6D OPT freq=noraman INT(grid=ultrafine) SCRF=(SMD,Solvent=chlorofor
m)\\1b.smd.chloroform.smd\\0,1\N,1.6365689821,2.7149673183,0.032330693
7\C,0.4798844313,1.9431743017,0.1142635095\C,0.613262457,0.5295263991,
-0.1050234487\C,1.9273818659,-0.0524193587,-0.3292621776\N,3.007301587
4,0.8377235235,-0.388349509\C,2.9012878323,2.199272509,-0.1949741548\O
,-0.5906325386,2.5173833694,0.3912859783\O,3.8846619701,2.9267168443,-
0.2215246268\O,2.1306147541,-1.2592526365,-0.4638569232\C,1.5661936967
,4.1609003675,0.2433890304\C,4.3552086393,0.3271818095,-0.6300119868\H
,0.517897271,4.4416846075,0.2966646869\H,2.0540346891,4.6728979603,-0.
5871433567\H,2.0723139439,4.4297546987,1.1739408274\H,4.2731382107,-0.
732446595,-0.8583196115\H,4.9778816574,0.4712218246,0.2565306394\H,4.8
039238855,0.8613374108,-1.4691504214\C,-0.4473398911,-0.3992246982,-0.
025150287\C,-1.8345625549,-0.2603100638,-0.0433254647\C,-2.6638130706,
-1.3994274487,0.0538898142\H,-0.1028518272,-1.4290949863,-0.0512079672
\O,-2.4876254512,0.9012863952,-0.323606974\C,-2.3522554796,-2.62663896
16,0.6277734715\H,-1.8725546853,1.651951223,-0.1137119206\C,-1.3423470
76,-2.7726849517,1.5917903078\N,-0.8836658415,-3.9211140841,2.05180613
49\C,-1.2985555113,-5.1946636047,1.478011939\C,-0.0086816678,-3.979705
9903,3.2155311849\H,-3.6583299151,-1.2834546992,-0.3767338898\H,-2.988
1221977,-3.4780929648,0.4026247884\H,-0.9245644757,-1.8838025836,2.060
5899258\H,-1.3289107453,-5.1196434232,0.3885638966\H,-2.2877650414,-5.
4822840646,1.8524617426\H,-0.5742252644,-5.9586685218,1.764181399\H,0.
2053174403,-2.9685810022,3.563822531\H,0.9256603395,-4.4809553769,2.94
70624654\H,-0.4989394189,-4.5419345466,4.0169847529\\Version=ES64L-G16
RevA.03\State=1-A\HF=-970.4367424\RMSD=6.387e-09\RMSF=8.937e-06\Dipole
=-1.0556245,-4.5148098,2.2528815\Quadrupole=-15.8956029,16.4411722,-0.
5455693,10.2010684,-1.5455482,-12.5278376\PG=C01 [X(C13H17N3O4)]\\@

33.5.4 1-TS

1\1\GINC-R48\FTS\RM062X\Gen\C13H17N3O4\ROOT\07-May-2018\0\\#m062X/gen
6D OPT=(CalcFC,TS,Noeigen) freq=noraman INT(grid=ultrafine) SCRF=(SMD,
Solvent=chloroform)\\1-TS.smd.chloroform.smd\\0,1\N,2.3169326986,-1.04
4749811,-0.6317775875\C,1.0410089946,-1.3943343258,-0.2142402916\C,0.1
939495405,-0.4013160058,0.3026972596\C,0.6861747081,0.9368034897,0.467
4286157\N,1.9907878282,1.1918656314,0.0077946653\C,2.8211833802,0.2430
718626,-0.5475306624\O,0.7410101979,-2.6267313076,-0.3357660159\O,3.94
71231818,0.5315843522,-0.9356645104\O,0.0291731729,1.8595873759,0.9643
656113\C,3.1765107635,-2.1026449407,-1.1636124652\C,2.54608059,2.53882
90563,0.1197454119\H,3.3286131465,-2.8716904767,-0.4038795076\H,4.1278
696994,-1.6543936259,-1.43824707\H,2.708606001,-2.5552083847,-2.039780
5785\H,3.4440487093,2.5213543462,0.7413759241\H,1.7889173744,3.1728253
699,0.5741217086\H,2.8100838955,2.9160019212,-0.8707338455\C,-1.196402
7487,-0.613901573,0.7087488929\C,-1.8505681711,-1.8612034362,0.9437272
834\C,-3.2861318221,-1.7317800897,1.0380075315\O,-1.3006182151,-3.0354
378164,0.9102966549\C,-3.7156294063,-0.6755083584,0.3127192641\H,-0.33
35069411,-2.9401070745,0.4265556441\C,-2.6593803665,-0.0397894157,-0.4
934713463\N,-2.7201779541,1.2901543039,-0.7685056931\C,-3.1023553822,2
.2340335611,0.270926039\C,-1.8501290175,1.8539173029,-1.7888174238\H,-
3.9108907224,-2.4524950496,1.5540748586\H,-4.7371162458,-0.3017073409,
0.2941702043\H,-3.7123660981,1.7390412292,1.0297536625\H,-3.6906105835
,3.0448696117,-0.1701192329\H,-2.2051451483,2.6522832383,0.7459993944\
H,-1.5857696597,1.0859938368,-2.519434628\H,-0.9351626934,2.263028876,
-1.339989767\H,-2.3769574601,2.6631478944,-2.30503529\H,-2.3117075377,
-0.6342256495,-1.3369367173\H,-1.5354447083,0.169974422,1.3835800066\\
Version=ES64L-G16RevA.03\State=1-A\HF=-970.4155941\RMSD=5.960e-09\RMSF
=1.907e-06\Dipole=-3.0118966,0.7819815,-0.4811966\Quadrupole=5.1678919
,-5.2313898,0.0634979,-4.9459725,1.4296726,-1.8953351\PG=C01 [X(C13H17
N3O4)]\\@

33.5.5 2a

1\1\GINC-R3867\FOpt\RM062X\Gen\C14H19N3O4\ROOT\05-May-2018\0\\#m062X/g
en 6D OPT freq=noraman INT(grid=ultrafine) SCRF=(SMD,Solvent=chlorofor
m)\\2.smd.chloroform.a3.global.smd\\0,1\N,1.652216367,2.6673171494,-0.
1164928246\C,0.4096747287,2.0409325703,-0.091454929\C,0.3939587699,0.6
117820763,0.0791103092\C,1.6474080852,-0.1199558052,0.2149830165\N,2.8
252538047,0.6367796845,0.1717088596\C,2.8623322997,2.0055964324,0.0135
053841\O,-0.6036730081,2.7525473595,-0.2184425701\O,3.9208279388,2.617
8201183,-0.0126840115\O,1.7185404769,-1.3388637888,0.3625595205\C,1.73
91742443,4.1179127806,-0.2883429852\C,4.120532486,-0.0273996786,0.3038
884408\H,0.729237006,4.5059967001,-0.3862762083\H,2.3196311397,4.34835
33197,-1.1838945735\H,2.2313316804,4.5634803122,0.5785202791\H,3.93729
3179,-1.093416589,0.4105138714\H,4.6459785349,0.3533772358,1.182312066
\H,4.7277002185,0.163347353,-0.5834353228\C,-0.7654881718,-0.187246964
4,0.1268271211\C,-2.135124025,0.0635071257,0.0504404919\C,-2.998687823
9,-1.0379344305,0.1443599528\H,-0.5325877508,-1.2410334698,0.255307245
6\O,-2.7249415493,1.2765937086,-0.1015601797\H,-2.5417318352,-2.017696
1776,0.2644203359\H,-2.0073137026,1.961615602,-0.1621791217\C,-4.38779
173,-0.9419660938,0.098018058\C,-5.163807041,-2.0947314177,0.214047838
8\N,-6.4851797493,-2.1448730697,0.1760951321\C,-7.2699176986,-0.926333
8159,0.0158537492\C,-7.2108637543,-3.3952538691,0.4138373315\H,-4.8479
215706,0.0333576805,-0.0164550857\H,-4.6662515341,-3.0548490399,0.3481
083237\H,-7.1495953974,-0.2698833304,0.8845758273\H,-8.3214632325,-1.1
955158279,-0.0864561382\H,-6.9506679529,-0.3918834791,-0.8836656063\C,
-7.8357877239,-3.4292526833,1.8030357692\H,-6.5050003954,-4.2195491753
,0.2871996403\H,-7.978612326,-3.4870576639,-0.3613483594\H,-8.38153843
73,-4.3678030738,1.9406206358\H,-7.0619767167,-3.3602320875,2.57421449
02\H,-8.5407478324,-2.6034286779,1.9429972253\\Version=ES64L-G16RevA.0
3\State=1-A\HF=-1009.747197\RMSD=9.203e-09\RMSF=8.222e-06\Dipole=-5.78
47569,-2.9662646,0.2807893\Quadrupole=20.6901415,-5.1223121,-15.567829
4,21.6173892,-1.9010369,-0.9339136\PG=C01 [X(C14H19N3O4)]\\@

33.5.6 2a''

1\1\GINC-R3718\FOpt\RM062X\Gen\C14H19N3O4\ROOT\05-May-2018\0\\#m062X/g
en 6D OPT freq=noraman INT(grid=ultrafine) SCRF=(SMD,Solvent=chlorofor
m)\\2a.smd.chloroform.a2.global.smd\\0,1\N,-0.5616565319,1.8704810452,
0.0744742163\C,-1.8503434713,1.3442956642,0.0907362558\C,-1.9780701749
,-0.0905594591,0.0676623336\C,-0.7845167928,-0.9260581352,-0.004636519
4\N,0.4487904911,-0.2637088655,-0.0078411324\C,0.5929936669,1.10731089
58,0.0200879586\O,1.696484702,1.6340482388,-0.0025466964\O,-0.80892590
7,-2.1549699178,-0.0558162835\O,-2.8051690256,2.1407156265,0.111627538
5\C,-3.1943062654,-0.8005927607,0.0744355188\C,-4.5370416159,-0.454330
3687,0.1982432301\C,-5.5338132922,-1.4468834648,0.1642860447\C,-5.4210
554737,-2.8257246262,-0.0164676088\C,-6.5810188324,-3.5970258501,0.003
6969657\N,-6.6455213655,-4.9124311869,-0.1346337365\O,-5.0165599775,0.
8084570451,0.3931930341\C,-0.3626792152,3.320161541,0.0906216055\C,1.6
883271999,-1.0363223215,-0.0655914959\H,-3.0293611332,-1.8688168243,-0
.0250975227\H,-6.5439180164,-1.0573198431,0.302448805\H,-4.4593778676,
-3.3005615351,-0.1793877916\H,-7.5396379081,-3.0998914436,0.1455166609
\C,-7.9344237041,-5.604063138,-0.2051047464\C,-5.4398350877,-5.7011997
767,-0.3541328207\H,-4.2591302467,1.4427801832,0.3084514976\H,-8.69404
70247,-4.9287046292,0.195702372\H,-7.8802071682,-6.4785518446,0.451827
5136\C,-8.2766345425,-6.0184508929,-1.6307439058\H,-4.990628152,-5.464
9487855,-1.3252909941\H,-5.70290958,-6.7592070949,-0.3296847935\H,-8.3
516562488,-5.1396817943,-2.2792055875\H,-9.2367810183,-6.5435199609,-1
.643842424\H,-7.517316352,-6.6908945414,-2.0430591883\H,-4.710845637,-
5.4989106975,0.4360700553\H,2.2212436408,-0.8202987633,-0.9945571486\H
,2.3265972012,-0.7719256829,0.779506085\H,1.4253450854,-2.0900877281,-
0.0229698069\H,0.2517484558,3.5966346762,0.9493091285\H,0.1429133657,3
.6349497447,-0.824845426\H,-1.3384961806,3.7926352724,0.1595798094\\Ve
rsion=ES64L-G16RevA.03\State=1-A\HF=-1009.7387674\RMSD=7.914e-09\RMSF=
7.982e-06\Dipole=-3.9300055,-4.7135155,-0.3911018\Quadrupole=3.5701173
,9.0796686,-12.6497859,25.110595,1.8704548,1.3098783\PG=C01 [X(C14H19N
3O4)]\\@

33.5.7 2a'''

1\1\GINC-R3742\FOpt\RM062X\Gen\C14H19N3O4\ROOT\05-May-2018\0\\#m062X/g
en 6D OPT freq=noraman INT(grid=ultrafine) SCRF=(SMD,Solvent=chlorofor
m)\\2b.smd.chloroform.smd\\0,1\N,1.6598312927,2.651503667,0.0438620364
\C,0.4981470376,1.8884626157,0.1345812745\C,0.6185168489,0.4732980847,
-0.0804415744\C,1.9284429827,-0.1211906786,-0.2939975065\N,3.018220539
5,0.7580994068,-0.3392861525\C,2.9237691505,2.121809945,-0.1539408806\
O,-0.565878393,2.4743196642,0.4130116194\O,3.916260805,2.8372027781,-0
.1648087761\O,2.120798925,-1.3299780044,-0.4279744556\C,1.5924569115,4
.0991433293,0.2430913134\C,4.3650915537,0.2329617087,-0.5540085935\H,0
.5514370847,4.4018331604,0.1675917555\H,2.1888037905,4.5932817436,-0.5
238271595\H,1.9849735561,4.3657095813,1.2283629562\H,4.2773992977,-0.8
2989008,-0.7643143944\H,4.9768036848,0.3888405662,0.3380697601\H,4.829
467125,0.7479569464,-1.3966940402\C,-0.4525262425,-0.4442268138,-0.001
9941152\C,-1.8368861413,-0.2875260962,-0.0304846746\C,-2.6845371211,-1
.4136020617,0.0671009348\H,-0.1204953748,-1.4785446911,-0.0192543635\O
,-2.4723344263,0.8811414462,-0.3234690568\C,-2.4013259709,-2.646638711
2,0.6426295574\H,-1.8525625309,1.6252598284,-0.1038215656\C,-1.3974311
462,-2.8224329174,1.6085941466\N,-0.9895425984,-3.9846957342,2.0809087
014\C,-1.4731112221,-5.2378385427,1.5124012184\C,-0.0137633378,-4.0593
335455,3.1745489556\H,-3.6733856651,-1.2812021868,-0.3720637428\H,-3.0
543953038,-3.4825679708,0.4093421981\H,-0.943801467,-1.9468087741,2.06
93848495\H,-1.345775092,-5.2340545988,0.4260798461\H,-2.5324836892,-5.
3793594392,1.7524312121\H,-0.9025188163,-6.0633003522,1.9374947399\H,0
.0176485448,-3.0791454029,3.655504727\C,1.3676076788,-4.4558575585,2.6
699653461\H,-0.3898340574,-4.7817087702,3.9064585998\H,2.0647769584,-4
.506953376,3.5119441627\H,1.3527433513,-5.4361439838,2.1831267929\H,1.
7407004765,-3.717397182,1.9536603479\\Version=ES64L-G16RevA.03\State=1
-A\HF=-1009.7341665\RMSD=2.971e-09\RMSF=7.864e-06\Dipole=-1.0310927,-4
.5649966,2.2378531\Quadrupole=-13.9007831,14.420678,-0.5198949,10.1040
351,-1.2211665,-10.9537283\PG=C01 [X(C14H19N3O4)]\\@

33.5.8 2-TS

1\1\GINC-R98\FTS\RM062X\Gen\C14H19N3O4\ROOT\07-May-2018\0\\#m062X/gen
6D OPT=(CalcFC,TS,Noeigen) freq=noraman INT(grid=ultrafine) SCRF=(SMD,
Solvent=chloroform)\\2-TS.smd.chloroform.smd\\0,1\N,2.3391262624,-1.09
23353936,-0.6228697532\C,1.0565834683,-1.4110873138,-0.2008725085\C,0.
2220495539,-0.3913286543,0.2798377144\C,0.7305186456,0.9447806012,0.40
72474265\N,2.0424306269,1.166858249,-0.0499350159\C,2.8622815829,0.189
8114943,-0.5711054209\O,0.7413186905,-2.6435265617,-0.2849599674\O,3.9
952697706,0.4502205769,-0.9589030704\O,0.0822030805,1.8902692183,0.871
1843007\C,3.1860228949,-2.1778437424,-1.1180688095\C,2.6160888441,2.50
8450594,0.0256510908\H,2.7065630585,-2.6614849363,-1.9709075285\H,3.33
92731293,-2.9171783135,-0.329405247\H,4.1383433797,-1.7486705506,-1.41
85118246\H,3.5108772458,2.4966083703,0.6518931477\H,1.865685432,3.1654
585598,0.4581831669\H,2.889584891,2.8534578295,-0.9740301572\C,-1.1697
890389,-0.5712411009,0.6998874254\C,-1.851552253,-1.8020125182,0.95233
35818\C,-3.2806589075,-1.6262375913,1.0611862262\O,-1.3334412503,-2.98
94857532,0.9216930403\C,-3.6807230574,-0.5581862778,0.3345760831\H,-0.
3537487271,-2.9190655572,0.4507758814\C,-2.6092129858,0.0364027268,-0.
4852153294\N,-2.6279521799,1.3671532924,-0.7595171252\C,-3.0315776407,
2.324896814,0.267491355\C,-1.7901636557,1.9026104137,-1.8198993635\H,-
3.9242402869,-2.3228124323,1.5871899548\H,-4.6882063587,-0.1473167822,
0.3261163818\H,-3.2183189629,1.7741334909,1.1970119814\C,-4.2717312851
,3.1169760459,-0.1308651643\H,-2.183718305,2.9945224878,0.4578500403\H
,-1.5658257163,1.1169880618,-2.5447784513\H,-0.8530140088,2.3030555772
,-1.4114636025\H,-2.3191310026,2.7090931159,-2.337857873\H,-2.29631239
19,-0.5704201709,-1.3339205591\H,-1.4764761031,0.2194428915,1.38223527
77\H,-4.5216766167,3.8424867554,0.6501002906\H,-4.1047782216,3.6688734
776,-1.0617137489\H,-5.130576601,2.4534140061,-0.2760138468\\Version=E
S64L-G16RevA.03\State=1-A\HF=-1009.7121487\RMSD=4.598e-09\RMSF=2.507e-
06\Dipole=-3.02889,0.9103185,-0.4460798\Quadrupole=3.182052,-4.4186523
,1.2366003,-4.2007208,1.1474261,-1.4994054\PG=C01 [X(C14H19N3O4)]\\@

33.5.9 5a

1\1\GINC-R4159\FOpt\RM062X\Gen\C15H21N3O4\ROOT\05-May-2018\0\\#m062X/g
en 6D OPT freq=noraman INT(grid=ultrafine) SCRF=(SMD,Solvent=chlorofor
m)\\5.smd.chloroform.smd\\0,1\N,1.6980279044,2.6379861835,-0.060953646
3\C,0.4418044872,2.0378247475,-0.0491695676\C,0.3944964804,0.608822929
7,0.1053953112\C,1.6297910794,-0.1507474597,0.2432980776\N,2.824616468
9,0.580315706,0.2143666854\C,2.8926694289,1.9487254328,0.065047842\O,-
0.5554324504,2.7733586254,-0.1724294139\O,3.9650334745,2.5372224955,0.
0447198232\O,1.6741258757,-1.3726084914,0.3803910986\C,1.8166387562,4.
088170159,-0.2151913709\C,4.104454741,-0.1134388709,0.3446560941\H,0.8
145857136,4.5006426847,-0.2922603197\H,2.3885225811,4.3183432851,-1.11
6447281\H,2.3322013026,4.5097579162,0.649893221\H,3.8967416058,-1.1745
586399,0.4555124012\H,4.6410060859,0.2584512364,1.220061616\H,4.713490
142,0.0600006806,-0.5450758238\C,-0.7839525385,-0.1657470777,0.1391826
142\C,-2.1452413602,0.1146929583,0.0490029846\C,-3.0342971383,-0.96908
22796,0.1344509023\H,-0.5744307506,-1.2247029643,0.2659545679\O,-2.708
93908,1.3394002272,-0.1118978667\H,-2.5980535193,-1.9572849308,0.26115
08105\H,-1.9766776477,2.0104149676,-0.1530461081\C,-4.4188131941,-0.85
01144918,0.0683886926\C,-5.2052190931,-1.9993087225,0.1738457401\N,-6.
5250739789,-2.0647613396,0.148329972\C,-7.3597521065,-0.8707417589,-0.
0170430585\C,-7.2186955308,-3.3496210989,0.2872289701\H,-4.862244454,0
.1304273206,-0.0668898653\H,-4.7017553495,-2.9592574568,0.2914353618\H
,-6.9327491101,-0.0580939461,0.5770888225\H,-8.3402026408,-1.104203964
4,0.4064498612\C,-7.4894689293,-0.4765435886,-1.4843726086\H,-6.536485
9784,-4.1400058796,-0.0353459971\H,-8.0672099508,-3.3398222951,-0.4046
529898\C,-7.6844205403,-3.5886377791,1.7180710249\H,-8.1263569829,0.40
84609277,-1.5758421017\H,-6.5116408184,-0.2435777575,-1.9164926579\H,-
7.9413804676,-1.2872790685,-2.0649342799\H,-8.2254452762,-4.5379508093
,1.7792947845\H,-6.8273646005,-3.6327754307,2.3974732104\H,-8.35411164
02,-2.7918513825,2.0567574667\\Version=ES64L-G16RevA.03\State=1-A\HF=-
1049.0436227\RMSD=4.587e-09\RMSF=2.700e-05\Dipole=-6.0576765,-2.947770
4,0.1085434\Quadrupole=17.8137644,-4.1800988,-13.6336656,19.3036918,-1
.0810253,-0.7652212\PG=C01 [X(C15H21N3O4)]\\@

33.5.10 5a''

1\1\GINC-R4313\FOpt\RM062X\Gen\C15H21N3O4\ROOT\05-May-2018\0\\#m062X/g
en 6D OPT freq=noraman INT(grid=ultrafine) SCRF=(SMD,Solvent=chlorofor
m)\\5a.smd.chloroform.smd\\0,1\N,-0.6180505571,1.8921671725,-0.0413520
34\C,-1.8917647096,1.3291674582,-0.0587564683\C,-1.9804710863,-0.10544
54489,0.0218556889\C,-0.7648133009,-0.9072630347,0.0836002973\N,0.4497
696559,-0.2103733228,0.0933708046\C,0.5567815108,1.1626051193,0.029629
2877\O,1.646073407,1.7192502516,0.0335914834\O,-0.7539997336,-2.137071
1308,0.1302498094\O,-2.866521729,2.0965656051,-0.1521598566\C,-3.17815
00541,-0.8500259798,0.0201576974\C,-4.5326706461,-0.5370167721,0.02987
28908\C,-5.5013743916,-1.5605879019,0.0244710059\C,-5.3414146411,-2.94
39590986,-0.0006497253\C,-6.4835595248,-3.7450481852,0.0052363539\N,-6
.5279878451,-5.0669909152,-0.0346717164\O,-5.058704785,0.7218110302,0.
0691259025\C,-0.4582506469,3.3445339965,-0.1200981435\C,1.7101371445,-
0.9472147111,0.1631841183\H,-2.9798475734,-1.9170382034,0.0248160637\H
,-6.5280560255,-1.1908266663,0.0445574336\H,-4.3576832667,-3.400930031
,-0.019647272\H,-7.4569552614,-3.2573107097,0.0462275867\C,-5.31315339
91,-5.8858473512,-0.0796602043\C,-7.8135470712,-5.7723123063,-0.032197
1093\H,-4.3124161252,1.3701219631,-0.0121037244\H,-4.596592196,-5.4153
720068,-0.7590232413\H,-5.5907072311,-6.8480507675,-0.5180470649\C,-4.
7150825467,-6.0825243226,1.3093826128\H,-8.5591001471,-5.1099712307,0.
4146102709\H,-7.709145566,-6.6447827521,0.6213641112\C,-8.2350485376,-
6.1900908251,-1.4354462848\H,-4.4361393162,-5.1241981398,1.7579125933\
H,-3.8181428971,-6.7056371809,1.2428787816\H,-5.4312383251,-6.57907044
74,1.9721519006\H,-8.3702291823,-5.3104884444,-2.0727908017\H,-9.18258
81312,-6.7360447883,-1.3914194551\H,-7.4878890779,-6.8423583441,-1.898
3808573\H,2.3072217839,-0.7499005964,-0.7299114841\H,2.2743008488,-0.6
33325192,1.0437531524\H,1.472770586,-2.0059090234,0.2274622712\H,0.101
5442102,3.6994986122,0.7470577956\H,0.0877983115,3.6088822412,-1.02830
31537\H,-1.4483329305,3.7913303804,-0.1365103168\\Version=ES64L-G16Rev
A.03\State=1-A\HF=-1049.0351309\RMSD=6.685e-09\RMSF=7.270e-06\Dipole=-
3.8928368,-5.0216909,-0.0729764\Quadrupole=2.3561898,8.7618933,-11.118
0831,22.6232956,0.3900488,0.5508873\PG=C01 [X(C15H21N3O4)]\\@

33.5.11 5a'''

1\1\GINC-R4437\FOpt\RM062X\Gen\C15H21N3O4\ROOT\05-May-2018\0\\#m062X/g
en 6D OPT freq=noraman INT(grid=ultrafine) SCRF=(SMD,Solvent=chlorofor
m)\\5b.smd.chloroform.smd\\0,1\N,1.701320579,2.6174061971,0.1028456298
\C,0.5331614246,1.8594503499,0.1641959877\C,0.645099943,0.4530950259,-
0.0963021572\C,1.947634427,-0.1416144985,-0.3420506799\N,3.0430300937,
0.7325084212,-0.3687775184\C,2.9570459306,2.0903231479,-0.1438586573\O
,-0.5288540688,2.4418459328,0.461332735\O,3.9509394367,2.8044783315,-0
.1588817804\O,2.131283925,-1.3461482035,-0.5219630153\C,1.6519182975,4
.0584640614,0.3485604146\C,4.3827772272,0.2100623405,-0.6283486792\H,0
.6083820199,4.3488081326,0.434192204\H,2.1245015783,4.5854267411,-0.48
17641142\H,2.183988013,4.3002124249,1.2719375814\H,4.2894205211,-0.851
992667,-0.8403730668\H,5.0212260347,0.3633544018,0.2450135732\H,4.8214
513788,0.7285344314,-1.4830550103\C,-0.4348186096,-0.4604788002,-0.042
3723646\C,-1.8146738027,-0.2877773746,-0.0872416574\C,-2.67719063,-1.4
091163631,-0.0314845791\H,-0.1114969196,-1.4973480877,-0.0743683307\O,
-2.4335621944,0.8934352127,-0.3631338977\C,-2.4199093152,-2.6434679558
,0.5454469643\H,-1.8112346747,1.6252622135,-0.1095531689\C,-1.44083731
83,-2.8184797606,1.5418978994\N,-1.0181445879,-3.9691728797,2.02483963
33\C,-1.4313754386,-5.2574574708,1.4602460355\C,-0.0943713601,-4.00767
16208,3.166610758\H,-3.6479566515,-1.2671541943,-0.506455915\H,-3.0736
937953,-3.4734680447,0.2951916273\H,-1.0216893849,-1.934043701,2.02071
9756\H,-1.570694028,-5.1316439854,0.383510591\C,-2.6962353453,-5.78397
87095,2.1300589276\H,-0.6013928601,-5.954346506,1.606084898\H,-0.22794
07827,-3.0866746402,3.7386092904\C,1.351607589,-4.1601843033,2.7132394
904\H,-0.3984452094,-4.843457906,3.8049247183\H,-2.9688870461,-6.75102
87257,1.6968680735\H,-2.5395699836,-5.9220728897,3.2045731993\H,-3.532
9402066,-5.0928160825,1.9899968026\H,2.0084042831,-4.2078924147,3.5872
539076\H,1.4940566528,-5.0750968945,2.1298687111\H,1.6536778583,-3.306
8676864,2.0981801833\\Version=ES64L-G16RevA.03\State=1-A\HF=-1049.0310
759\RMSD=2.658e-09\RMSF=2.677e-06\Dipole=-1.2700855,-4.7789647,2.33528
89\Quadrupole=-12.5597026,12.3887469,0.1709557,9.5013112,-0.4846196,-9
.2879883\PG=C01 [X(C15H21N3O4)]\\@

33.5.12 5-TS

1\1\GINC-R61\FTS\RM062X\Gen\C15H21N3O4\ROOT\08-May-2018\0\\#m062X/gen
6D OPT=(CalcFC,TS,Noeigen) freq=noraman INT(grid=ultrafine) SCRF=(SMD,
Solvent=chloroform)\\5-TS.smd.chloroform.a1b2.global.smd\\0,1\N,2.2616
156345,-1.0431183412,-0.6478230635\C,1.0019025268,-1.4209434669,-0.197
5971819\C,0.173287858,-0.444063997,0.375314431\C,0.6490747845,0.899320
805,0.552882722\N,1.9353805973,1.1795413857,0.0595339747\C,2.747726400
4,0.248441348,-0.5487211658\O,0.6840922008,-2.6464032532,-0.3379982296
\O,3.8579566162,0.542839887,-0.9764764973\O,-0.0060013386,1.8021714371
,1.0868887275\C,3.1637164434,-2.0214842432,-1.2552557982\C,2.482588158
,2.5282889643,0.1805196615\H,4.0971048956,-2.0616065077,-0.6901632573\
H,3.3853430409,-1.7308019972,-2.2844234433\H,2.6726634563,-2.990386716
8,-1.2376809925\H,3.4064094858,2.5046798206,0.7628548364\H,1.739423691
8,3.1431367762,0.6822912307\H,2.700359833,2.9327046388,-0.8106374176\C
,-1.2122802938,-0.6715664194,0.7880174262\C,-1.859419064,-1.9239254556
,1.0291809077\C,-3.2930660501,-1.7873003042,1.1175384783\O,-1.30093558
72,-3.0947494239,0.996663307\C,-3.7050953487,-0.7245193887,0.387590922
7\H,-0.3549217691,-2.98806106,0.487517595\C,-2.6274564507,-0.108051221
5,-0.4099055117\N,-2.6649823173,1.2230794153,-0.6898725306\C,-3.126330
4288,2.1606925632,0.3324256796\C,-1.799155638,1.8212737717,-1.71117100
45\H,-3.9287748869,-2.5028758082,1.6272010227\H,-4.7219393446,-0.33851
11283,0.3604453949\H,-3.286499721,1.6059480274,1.2650410196\C,-4.40647
67597,2.883844972,-0.0717792205\H,-2.3181702002,2.8768891648,0.5261560
048\H,-0.9214231716,2.2568162737,-1.2128820034\H,-2.3606578442,2.65075
64902,-2.1560106206\C,-1.3578913222,0.8701138341,-2.8113894906\H,-2.30
3607264,-0.7203074259,-1.2480374027\H,-1.5421165284,0.1005689175,1.481
2363617\H,-4.6971096623,3.5979584833,0.7057818228\H,-4.2681083318,3.43
98649716,-1.0049236503\H,-5.2284421137,2.1748313457,-0.2157121148\H,-0
.811189784,1.4422043251,-3.5668717314\H,-0.6844058817,0.090332017,-2.4
39164337\H,-2.2138405206,0.3942395236,-3.3025928619\\Version=ES64L-G16
RevA.03\State=1-A\HF=-1049.0078644\RMSD=8.722e-09\RMSF=1.793e-06\Dipol
e=-2.9752405,0.8276734,-0.5510047\Quadrupole=3.1449883,-4.3123252,1.16
7337,-3.3727766,0.5456959,-1.7896785\PG=C01 [X(C15H21N3O4)]\\@

33.5.13 8a

1\1\GINC-R227\FOpt\RM062X\Gen\C19H29N3O4\ROOT\07-May-2018\0\\#m062X/ge
n 6D OPT freq=noraman INT(grid=ultrafine) SCRF=(SMD,Solvent=chloroform
)\\8.smd.chloroform.smd\\0,1\N,-5.1291328512,1.0006814875,-0.462703902
4\C,-3.7427046261,0.9111730016,-0.4036389251\C,-3.1694840008,-0.306734
5955,0.0984721618\C,-4.0511129138,-1.3832226672,0.5216618575\N,-5.4286
347716,-1.1620627659,0.4147877625\C,-6.004535326,-0.0022278092,-0.0669
64548\O,-3.0991774431,1.9035248084,-0.7942629238\O,-7.2166931722,0.138
837222,-0.1426219676\O,-3.6670795183,-2.4653589681,0.9641583788\C,-5.6
895919478,2.2499226661,-0.9800849814\C,-6.3023914821,-2.2521449266,0.8
464120288\C,-1.7918166603,-0.5694815376,0.2323225965\C,-0.6312034565,0
.1511727617,-0.041716062\C,0.5962370026,-0.4675092114,0.2416656137\H,-
1.5972877842,-1.5572909265,0.6424023525\O,-0.5665176032,1.4037399059,-
0.5616678314\H,0.5593722303,-1.4715715606,0.6584733589\H,-1.4954059788
,1.7281226517,-0.7060832132\C,1.8368719731,0.1239660079,0.0198308073\C
,2.9965461791,-0.5896681588,0.3262543499\N,4.246280231,-0.1820281266,0
.1688318037\C,4.5528207033,1.1507275792,-0.3584032279\C,5.3668804008,-
1.0884438479,0.4315363029\H,1.87897655,1.1272270444,-0.3899210628\H,2.
8909684384,-1.5958785416,0.7314721186\H,3.9209595575,1.3344173398,-1.2
353537117\H,5.5927440978,1.1331724464,-0.7012919146\C,4.376358354,2.26
76588445,0.6792770068\H,5.0208091634,-1.8613528391,1.1270353192\H,6.15
23291623,-0.512811558,0.9329006325\C,5.9217844078,-1.7429671321,-0.840
4925334\C,4.650982654,3.6116727784,0.0069833466\H,3.3343492468,2.25468
34187,1.023598372\C,5.2865565586,2.0569515399,1.8877691939\C,7.1321459
216,-2.5974250348,-0.4643186619\C,4.8578465527,-2.5759420277,-1.551993
6965\H,6.2552624756,-0.9443756452,-1.5177353675\H,4.5097823941,4.43397
90664,0.7159890154\H,5.6843627858,3.6588883236,-0.3598870567\H,3.98050
80867,3.7804257762,-0.8431705216\H,7.577469701,-3.0513284396,-1.355556
089\H,7.9057597197,-2.0032768158,0.0349906339\H,6.8378355234,-3.409169
3946,0.2128564933\H,5.14940308,2.8646319372,2.6147729236\H,5.072031221
7,1.111763353,2.3992490002\H,6.3419960478,2.0542388336,1.5853122293\H,
5.2783039225,-3.053818362,-2.443309846\H,4.4821162526,-3.3699837921,-0
.8930826225\H,4.0054960687,-1.9675601918,-1.874022762\H,-6.7727357513,
2.165006862,-0.9706813059\H,-5.3719701225,3.0838601521,-0.3512081386\H
,-5.3339804176,2.4176221206,-1.9984556855\H,-7.333994823,-1.9354219062
,0.7180465898\H,-6.1040179153,-3.1418036165,0.2451457043\H,-6.10708109
94,-2.4841705298,1.8952016044\\Version=ES64L-G16RevA.03\State=1-A\HF=-
1206.2268341\RMSD=3.538e-09\RMSF=1.949e-06\Dipole=6.5911064,-0.3798539
,0.2017175\Quadrupole=9.1456976,-4.5119525,-4.633745,-5.6265813,1.1270
776,1.6019793\PG=C01 [X(C19H29N3O4)]\\@

33.5.14 8a''

1\1\GINC-R190\FOpt\RM062X\Gen\C19H29N3O4\ROOT\08-May-2018\0\\#m062X/ge
n 6D OPT freq=noraman INT(grid=ultrafine) SCRF=(SMD,Solvent=chloroform
)\\8a.smd.chloroform.a2b3.global.smd\\0,1\N,5.1507859145,-0.6886442368
,0.0977915113\C,3.8593061888,-1.1686151129,0.2789881655\C,2.7654408178
,-0.2822148704,-0.0185356181\C,3.0437533834,1.0724612209,-0.4772071725
\N,4.3923848191,1.4253764828,-0.6201544546\C,5.4558049449,0.5904972037
,-0.3438218698\O,6.6090168005,0.9717524614,-0.4853037915\O,2.172934933
5,1.9007244286,-0.7416271353\O,3.7476060343,-2.3360800055,0.6950159567
\C,1.3980193311,-0.5952113628,0.1134131692\C,0.6814126427,-1.730959221
4,0.4764500305\C,-0.7257549119,-1.7036321706,0.5237301256\C,-1.6122846
007,-0.6628983542,0.2520347395\C,-2.9790891776,-0.9054106455,0.3841825
541\N,-3.9661544093,-0.0440475528,0.1824942097\O,1.2222984298,-2.94089
84227,0.8020640585\C,6.2506845487,-1.601176195,0.411133488\C,4.7422415
914,2.7673080236,-1.0837023613\H,0.7713019454,0.2559841862,-0.13340234
12\H,-1.1705198542,-2.6534723347,0.8256129567\H,-1.2587888916,0.317828
918,-0.047812835\H,-3.2987781318,-1.9042648448,0.6788049956\C,-3.70414
85919,1.3446268685,-0.2043580424\C,-5.3612487245,-0.483949529,0.259498
1162\H,2.2104736718,-2.8510735981,0.8001167861\H,-4.6333515732,1.74607
4124,-0.622450068\H,-2.955540139,1.3517560441,-1.0052882332\C,-3.25044
9254,2.2266408051,0.967076603\H,-5.9312196574,0.2968970322,0.77450597\
H,-5.3943755441,-1.3880873044,0.8779817386\C,-4.3002201081,2.267760062
5,2.0758295945\C,-2.9477402963,3.6277058058,0.4387613324\H,-2.32703830
58,1.8008035952,1.3806204767\C,-5.9808184206,-0.7709599082,-1.11452541
69\H,-5.9366675457,0.1515434488,-1.7098744874\C,-7.4484829168,-1.15209
50987,-0.9207565359\C,-5.2187629813,-1.8684169613,-1.8537795356\H,-5.2
474003899,2.6772430539,1.7006329253\H,-3.9621392936,2.9069717883,2.898
4963627\H,-4.495128771,1.2716538147,2.4890728576\H,-3.8487734636,4.083
2629263,0.0082697678\H,-2.1736757518,3.6072771795,-0.3366431177\H,-2.5
973428987,4.2780371568,1.2468714441\H,-8.0039523187,-0.364462021,-0.39
92337951\H,-7.5343071422,-2.0745302109,-0.3325700852\H,-7.9342451565,-
1.3253592236,-1.8864951241\H,-4.1762555858,-1.5901656008,-2.0438043711
\H,-5.6876689174,-2.0755082201,-2.8217000099\H,-5.2230540164,-2.801165
2301,-1.2742670168\H,5.32138526,3.2869130413,-0.3172490969\H,5.3397575
569,2.7000130382,-1.9950173514\H,3.8169682224,3.3024308176,-1.28013039
19\H,7.1860048916,-1.0833688752,0.2160963432\H,6.1959090834,-1.8967060
332,1.4607304591\H,6.1770787295,-2.4934563834,-0.2134274792\\Version=E
S64L-G16RevA.03\State=1-A\HF=-1206.218439\RMSD=6.606e-09\RMSF=5.157e-0
6\Dipole=-6.1605351,0.7536247,-0.0016147\Quadrupole=12.3801692,-8.9917
546,-3.3884146,0.4699748,0.8745241,3.0067363\PG=C01 [X(C19H29N3O4)]\\@

33.5.15 8a'''

1\1\GINC-R3828\FOpt\RM062X\Gen\C19H29N3O4\ROOT\05-May-2018\0\\#m062X/g
en 6D OPT freq=noraman INT(grid=ultrafine) SCRF=(SMD,Solvent=chlorofor
m)\\8b.smd.chloroform.smd\\0,1\N,1.7723157975,2.5079226111,0.187269788
7\C,0.6021106386,1.7534289501,0.2390552456\C,0.6874388534,0.3744245208
,-0.1478131632\C,1.9648136557,-0.1955162666,-0.5416154436\N,3.07350741
43,0.6630689953,-0.5034381659\C,3.0164000448,1.9892460868,-0.130330455
8\O,-0.4383496564,2.3126389844,0.6394588707\O,4.0232165134,2.683956196
3,-0.0883121505\O,2.116191651,-1.3644808458,-0.8939105769\C,1.73866342
3,3.9187790584,0.571457353\C,4.3952548344,0.156054036,-0.8653333379\H,
0.7044555208,4.2518630178,0.5432168771\H,2.3427718173,4.4930809863,-0.
1304659234\H,2.141202257,4.0491357041,1.580101235\H,4.2785928564,-0.87
79363397,-1.1799700112\H,5.0664492926,0.213168269,-0.0051669569\H,4.81
10996989,0.7531188241,-1.6793568438\C,-0.3931760846,-0.5361637942,-0.0
866368022\C,-1.7719170919,-0.3394958518,-0.0738367097\C,-2.6446516875,
-1.4512663069,-0.0311837764\H,-0.0867610053,-1.5757306195,-0.173822146
7\O,-2.3729624731,0.8618529669,-0.2974179254\C,-2.3830364456,-2.690058
5369,0.5389923328\H,-1.7437925116,1.5698298164,0.0015029475\C,-1.42465
14851,-2.8469207503,1.5567173882\N,-1.0146727763,-3.9857614276,2.08453
28509\C,-1.4177310686,-5.283307577,1.5336002196\C,-0.060782895,-3.9838
475092,3.2009132695\H,-3.6172522771,-1.3008728552,-0.4998806588\H,-3.0
231568703,-3.5250207896,0.2738015943\H,-1.0103290749,-1.9506222167,2.0
14864201\H,-1.4091973367,-5.2065704791,0.4404099917\C,-2.787059764,-5.
7751078502,2.0258636767\H,-0.6482054019,-6.0084258524,1.8202283409\H,-
0.1351761767,-3.0134285744,3.7035276588\C,1.3878427397,-4.2296715161,2
.7604838615\H,-0.3752740485,-4.7548386784,3.9112032636\C,2.2779262988,
-4.234602422,4.0034369661\H,1.4419074666,-5.2215623251,2.2905125257\C,
1.846158871,-3.1818301226,1.7501714483\C,-3.0856261979,-7.117707146,1.
3594265065\C,-2.8450989219,-5.8931145919,3.5477551224\H,-3.5491944023,
-5.0515200306,1.7101830297\H,3.3162572611,-4.4515130702,3.7327751481\H
,2.2586349515,-3.2548987691,4.4971487062\H,1.9535574178,-4.9876271438,
4.7306769915\H,-4.0751266574,-7.4827590747,1.6532513372\H,-3.064819662
4,-7.0394046638,0.2665717065\H,-2.3472939899,-7.8719296282,1.659737700
1\H,-3.8237403558,-6.2730447944,3.8603084887\H,-2.0824781516,-6.591716
5159,3.9157905737\H,-2.6948710187,-4.9256459047,4.0395790695\H,2.89556
06735,-3.3373647724,1.4774755062\H,1.2642399282,-3.2154567042,0.824087
3406\H,1.7580196115,-2.1695317066,2.1689359139\\Version=ES64L-G16RevA.
03\State=1-A\HF=-1206.2136055\RMSD=1.659e-09\RMSF=5.021e-06\Dipole=-1.
2724439,-4.7026443,2.3345301\Quadrupole=-5.7988495,4.9194538,0.8793957
,6.7101887,0.8396869,-3.8654319\PG=C01 [X(C19H29N3O4)]\\@

33.5.16 8-TS

1\1\GINC-R100\FTS\RM062X\Gen\C19H29N3O4\ROOT\07-May-2018\0\\#m062X/gen
 6D OPT=(CalcFC,TS,Noeigen) freq=noraman INT(grid=ultrafine) SCRF=(SMD
,Solvent=chloroform)\\8-TS.smd.chloroform.smd\\0,1\N,3.6403893702,-0.1
316496587,0.3803118272\C,2.6787495755,0.8681951876,0.336163631\C,1.518
3889514,0.6676817916,-0.4246094752\C,1.3876481796,-0.5278635669,-1.206
727236\N,2.4127112229,-1.4801826462,-1.09524817\C,3.5348174468,-1.3333
203545,-0.3045319858\O,2.9413732473,1.9188847515,1.0086405737\O,4.3932
435714,-2.2030835182,-0.2166819251\O,0.4304199129,-0.7795044431,-1.948
328547\C,4.8361183376,0.112122508,1.1886588441\C,2.2578785781,-2.70786
08639,-1.8726736347\H,5.344404138,1.0092303848,0.8308973041\H,5.488069
0061,-0.7519799931,1.0925462909\H,4.5554116334,0.2550803792,2.23397975
36\H,3.1438889884,-3.3199992105,-1.7251965694\H,2.1421336113,-2.458180
5319,-2.9289087953\H,1.3689571994,-3.2491066146,-1.5395741234\C,0.3932
981783,1.5993332771,-0.4976496182\C,0.3953007953,2.9822472502,-0.13032
68489\C,-0.9343851179,3.5332096477,-0.06137289\O,1.4221999482,3.664556
5216,0.2714870673\C,-1.8323728739,2.5633148728,0.2312709702\H,2.197632
2579,2.9609871292,0.561527131\C,-1.2121756649,1.2717104279,0.573625160
1\N,-1.8508255341,0.0890522805,0.3242601022\C,-2.5787190028,-0.0793687
52,-0.9351842277\C,-1.256740131,-1.1250042184,0.8940320763\H,-1.142735
5642,4.5935801112,-0.1521913361\H,-2.9097801097,2.6978022246,0.2351133
576\H,-2.4126626705,0.8164127455,-1.5525528701\C,-4.0936033036,-0.3068
960375,-0.8110131341\H,-2.1208113863,-0.9206540671,-1.4730701861\H,-0.
5792426912,-0.8143953259,1.698831425\H,-0.6533511796,-1.6170547189,0.1
178019247\C,-2.2563002702,-2.134420195,1.4655579566\H,-0.6878338125,1.
274718533,1.5283059786\H,-0.2139852405,1.4060931568,-1.3802544382\C,-4
.6539499142,-0.6047340191,-2.2023842466\H,-4.2623173785,-1.1857730054,
-0.1773432508\C,-4.820355485,0.8795520654,-0.1830465126\C,-1.473945276
3,-3.3158734036,2.0410128659\C,-3.1531098792,-1.502479743,2.5260833976
\H,-2.8787122749,-2.5135816621,0.6443049307\H,-5.7328160142,-0.7883546
119,-2.1580381718\H,-4.4866335755,0.2448762232,-2.8770830386\H,-4.1782
454997,-1.4858454991,-2.6475943127\H,-3.8546474371,-2.2385857312,2.934
4307723\H,-2.548053786,-1.1174819307,3.3579444695\H,-3.7342451145,-0.6
677889606,2.1207424414\H,-2.153882008,-4.088365799,2.4156438311\H,-0.8
253664651,-3.7747336252,1.2856997562\H,-0.8414130872,-2.991736374,2.87
73202135\H,-5.8883085785,0.6602048683,-0.0733507504\H,-4.4265560311,1.
1210355093,0.8105933302\H,-4.728362792,1.7714322344,-0.8173630879\\Ver
sion=ES64L-G16RevA.03\State=1-A\HF=-1206.1895149\RMSD=3.427e-09\RMSF=6
.431e-07\Dipole=-3.1392606,0.3209933,0.5482237\Quadrupole=-0.6833417,0
.0441093,0.6392324,-0.5446256,-0.0835075,-3.1131426\PG=C01 [X(C19H29N3
O4)]\\@

33.5.17 14a

1\1\GINC-R225\FOpt\RM062X\Gen\C19H21N3O5\ROOT\07-May-2018\0\\#m062X/ge
n 6D INT(grid=ultrafine) OPT Freq=noraman SCRF=(SMD,Solvent=chloroform
)\\14.smd.chloroform.freq\\0,1\N,1.6697289183,2.6863362599,-0.10740042
88\C,0.428905626,2.0680313093,-0.0211545448\C,0.4097060254,0.625316151
,0.0296786449\C,1.667337853,-0.1200071427,0.0221995027\N,2.843708785,0
.6349868194,-0.0306363386\C,2.8821208561,2.0133269308,-0.0724053097\O,
-0.5816572607,2.7908369829,0.0036667767\O,3.9420398577,2.6209417462,-0
.0852253774\O,1.7352702214,-1.345662405,0.0643373722\C,1.7459271705,4.
1471049548,-0.1643582421\C,4.1407164904,-0.0402512941,-0.0145171339\C,
-0.7438456062,-0.1673902073,0.0967020421\C,-2.1214194634,0.0906534669,
0.1283428\C,-2.9738280605,-1.0118887819,0.1975063343\H,-0.5123299413,-
1.2288284943,0.129710277\O,-2.7074274131,1.3134456073,0.096093853\H,-2
.5148291669,-1.9977353599,0.219211888\H,-1.9912370727,2.0002059596,0.0
596590061\C,-4.3720765567,-0.920811057,0.2387212428\C,-5.1233212546,-2
.082967004,0.3003750212\N,-6.4574212599,-2.1558776579,0.3211673471\C,-
7.2521225381,-0.9315365001,0.2352241889\C,-7.1125431271,-3.418839054,0
.4317612132\H,-4.8392486401,0.0577235697,0.2138905345\H,-4.6104764242,
-3.0425074652,0.3162526037\H,-6.9498842298,-0.2425802964,1.0303032561\
H,-8.3044455627,-1.1768166434,0.3684404911\H,-7.1056260641,-0.44618122
49,-0.7351454\C,-8.2067409654,-3.7152506924,-0.3911066983\C,-8.8351379
505,-4.9459449714,-0.296298295\C,-8.3766812094,-5.9070833333,0.6157124
037\C,-7.2866495425,-5.615683218,1.4386012763\C,-6.6670325328,-4.36788
37925,1.3478360715\H,-8.5540875457,-2.9869791231,-1.1185805481\H,-9.68
04292699,-5.1894305437,-0.9329624651\O,-9.0532752443,-7.0823894118,0.6
299073542\H,-6.9216072149,-6.3345237319,2.163184301\H,-5.840578445,-4.
1329925886,2.0131937488\C,-8.6177999623,-8.0851789122,1.5349945659\H,-
9.2825617963,-8.9352734705,1.3807170283\H,-7.5852529749,-8.3849885597,
1.322389028\H,-8.6995343494,-7.7409537187,2.5722509132\H,2.4893764296,
4.4353632575,-0.9068423501\H,0.7661304446,4.5265942972,-0.4415978648\H
,2.0365097342,4.549231374,0.8101356556\H,4.6982916815,0.245172963,0.88
02607356\H,3.9574776356,-1.1115900028,-0.0147045316\H,4.7156829159,0.2
439140093,-0.8978879497\\Version=ES64L-G16RevA.03\State=1-A\HF=-1276.6
054609\RMSD=3.560e-09\RMSF=2.544e-06\Dipole=-4.5588552,-3.017732,0.664
3896\Quadrupole=-4.7345226,6.4459661,-1.7114435,2.7967199,-2.1701894,-
8.9306886\PG=C01 [X(C19H21N3O5)]\\@

33.5.18 14a''

1\1\GINC-R4472\FOpt\RM062X\Gen\C19H21N3O5\ROOT\06-May-2018\0\\#m062X/g
en 6D INT(grid=ultrafine) OPT Freq=noraman SCRF=(SMD,Solvent=chlorofor
m)\\14a.a2.smd.chloroform.freq\\0,1\N,-0.5666674894,1.9298271115,-0.06
63081127\C,-1.8449484506,1.3858855591,-0.0772087372\C,-1.950399827,-0.
0563499164,-0.0390506179\C,-0.7364411094,-0.8737318802,0.010125516\N,0
.4821386424,-0.1896961141,0.013011961\C,0.6017776441,1.1844124471,-0.0
211806907\O,1.6948119231,1.7295477904,-0.0128293683\O,-0.7423522609,-2
.1014695087,0.0473133907\O,-2.8119800844,2.1627802985,-0.1165078364\C,
-3.1449828418,-0.7862156017,-0.0380564927\C,-4.5082917499,-0.465855163
1,-0.0803645047\C,-5.4709176237,-1.4794379255,-0.0598608509\C,-5.31688
39873,-2.8746999885,-0.0005846752\C,-6.4540676529,-3.6624634561,0.0102
552525\N,-6.4844518543,-4.99873328,0.0929294564\O,-5.0192011944,0.7959
88593,-0.1457845875\C,-0.3891716299,3.3827476217,-0.0985012884\C,1.737
2154147,-0.939031063,0.0577612876\H,-2.9554269169,-1.8541924692,0.0048
332371\H,-6.4970376155,-1.110034792,-0.0966476586\H,-4.3346076418,-3.3
325171605,0.0420378604\H,-7.4291865529,-3.1816656851,-0.0318601287\C,-
5.2363735138,-5.7450631196,0.2362635426\C,-7.723537511,-5.7008229852,0
.0236367809\H,-4.2662964412,1.4398325451,-0.1415933028\H,-4.7495122677
,-5.5009560427,1.1861825355\H,-4.5610173593,-5.4945445223,-0.587992215
4\H,-5.4496846112,-6.8118752658,0.1979424725\H,2.339429553,-0.70478966
46,-0.8223850867\H,2.298170132,-0.6695850189,0.9550701163\H,1.49202107
86,-1.9977457393,0.0731797067\H,0.1456450738,3.7078583056,0.7959683657
\H,0.1887137256,3.6634014912,-0.9810801752\H,-1.3735399198,3.840835674
6,-0.1334961126\C,-7.9831289326,-6.7561898621,0.9078859003\C,-9.189703
9699,-7.4337480825,0.8484063807\C,-10.1651523535,-7.0643564958,-0.0883
983025\C,-9.9120689521,-6.0121413488,-0.9709669992\C,-8.6875320575,-5.
3436656715,-0.9156920285\H,-7.2454044002,-7.0357850051,1.6548477849\H,
-9.4031199988,-8.2500193394,1.5319816649\O,-11.3138457302,-7.785729806
4,-0.0644977805\H,-10.6415562935,-5.7138470329,-1.7152084232\H,-8.4818
778167,-4.5494920112,-1.6282296872\C,-12.327529849,-7.4480508583,-0.99
85655323\H,-13.1501219973,-8.1377134447,-0.8086105167\H,-12.6723069974
,-6.4183185566,-0.849756632\H,-11.972741732,-7.5766765596,-2.027615868
1\\Version=ES64L-G16RevA.03\State=1-A\HF=-1276.5970628\RMSD=6.097e-10\
RMSF=1.303e-05\Dipole=-3.4324758,-3.795667,-0.3876876\Quadrupole=6.423
477,-5.4579766,-0.9655004,5.3055055,8.9460464,1.2029522\PG=C01 [X(C19H
21N3O5)]\\@

33.5.19 14a'''

1\1\GINC-R4472\FOpt\RM062X\Gen\C19H21N3O5\ROOT\06-May-2018\0\\#m062X/g
en 6D INT(grid=ultrafine) OPT Freq=noraman SCRF=(SMD,Solvent=chlorofor
m)\\14b.smd.chloroform.freq\\0,1\N,1.6435498844,2.7094860369,-0.005119
4683\C,0.4828742896,1.9535465341,0.0604979808\C,0.5997296441,0.5263668
324,-0.1164025578\C,1.9202362379,-0.0711593296,-0.2999175816\N,3.00664
20258,0.809747669,-0.3420241401\C,2.9142073875,2.1792504894,-0.1890037
983\O,-0.5802316638,2.5571877603,0.2855323134\O,3.9116240262,2.8837462
472,-0.215782206\O,2.1110352996,-1.2790193243,-0.4137332745\C,1.508050
5679,4.1561426797,0.1712999455\C,4.3567517639,0.280735023,-0.534709211
\H,0.7908594281,4.5430513059,-0.5541525453\H,2.4833518416,4.6095450955
,0.0155890345\H,1.1487292316,4.375568736,1.1791851561\H,4.273649274,-0
.7906215262,-0.6974887908\H,4.9659553243,0.4791411727,0.3500245296\H,4
.8189421281,0.7594623987,-1.3997227474\C,-0.460340084,-0.3836897305,-0
.0370897014\C,-1.8588733957,-0.2368961895,-0.0502859697\C,-2.683774535
7,-1.3594707865,0.0843469187\H,-0.1264320522,-1.4173159006,-0.05880077
36\O,-2.4958743493,0.9249920214,-0.3620593947\C,-2.3556952681,-2.60697
26953,0.639068121\H,-1.8749970192,1.6757980982,-0.1779106542\C,-1.3463
749551,-2.7694227224,1.5802175728\N,-0.8937396923,-3.9427291447,2.0337
331872\C,-1.3436059012,-5.1870178905,1.4136596284\C,-0.0530389651,-3.9
903003087,3.1860244166\H,-3.7000949619,-1.231972846,-0.2882389097\H,-2
.987251661,-3.4545173367,0.3882285459\H,-0.8917965703,-1.8931611171,2.
0376977598\H,-1.1851333619,-5.1421795863,0.3325347921\H,-2.4086759917,
-5.3460364913,1.6179745724\H,-0.7827611816,-6.0214564636,1.8309305954\
C,1.0916074134,-4.7986084109,3.1879269864\C,1.9169996766,-4.8303310908
,4.2989679395\C,1.6212679568,-4.0478897183,5.425051515\C,0.4850417375,
-3.2367009515,5.4255514792\C,-0.3511300363,-3.2209403803,4.3071617754\
H,1.3463405298,-5.3856988162,2.3098209001\H,2.8110749383,-5.4463758468
,4.3104797863\O,2.4915806459,-4.1470584388,6.4603871041\H,0.2281859408
,-2.6282308964,6.2851335584\H,-1.2515294904,-2.6125469434,4.3223153214
\C,2.2297261755,-3.3736650871,7.6212818101\H,3.0375040151,-3.598088919
5,8.3180033242\H,2.2336670776,-2.3024381412,7.3894877685\H,1.270227674
8,-3.6531900691,8.0712703857\\Version=ES64L-G16RevA.03\State=1-A\HF=-1
276.5924406\RMSD=5.696e-09\RMSF=3.391e-06\Dipole=-0.8343948,-3.246701,
2.4688457\Quadrupole=-9.7517387,1.1766533,8.5750855,9.1590055,0.133752
4,5.4973185\PG=C01 [X(C19H21N3O5)]\\@

33.5.20 14-TS

1\1\GINC-R3711\FTS\RM062X\6-31+G(d)\C19H21N3O5\ROOT\07-May-2018\0\\#m0
62x/6-31+g(d) opt=(calcfc,ts,noeigen) freq=noraman scrf=(smd,solvent=c
hloroform)\\Title Card Required\\0,1\N,0.0168727607,2.6857492323,-0.60
5068019\C,1.128433865,1.8640912609,-0.7165413737\C,1.3866544437,0.9310
614997,0.3048691639\C,0.5414184695,0.8834564093,1.465952977\N,-0.59429
37479,1.7098722017,1.4468052702\C,-0.8870716722,2.6094700934,0.4439920
788\O,1.8486054418,2.0283190997,-1.7482035806\O,-1.8879056088,3.314067
1041,0.4927735093\O,0.7463594183,0.1559625674,2.4443008907\C,-0.220310
7767,3.6624615661,-1.6675936704\C,-1.5376500395,1.6627579777,2.5611460
838\H,-0.4501729535,3.149958932,-2.6047026589\H,0.6733358491,4.2726197
289,-1.8055486207\H,-1.0600230164,4.2855628818,-1.3705222125\H,-2.5517
404871,1.5554295495,2.1737083141\H,-1.477616526,2.5831723997,3.1489802
746\H,-1.2754550176,0.810651931,3.1839523331\C,2.4194021932,-0.0869004
233,0.2377398888\C,3.5568358025,-0.1481697408,-0.6228165389\C,4.153727
8158,-1.4572061744,-0.6756888104\O,3.9551153335,0.799097288,-1.4272220
603\C,3.2256374659,-2.417655787,-0.4353544927\H,3.1409365658,1.4618141
985,-1.5658700672\C,1.8595126857,-1.8932098263,-0.3957367279\N,0.88132
38813,-2.4582285971,0.3733322317\C,1.2309939427,-2.9400464893,1.706731
6621\C,-0.4784326979,-2.1062005395,0.1252445369\H,5.1888212952,-1.6241
683544,-0.9532941819\H,3.4409337322,-3.4732946196,-0.2851023056\H,2.23
52910355,-3.3652466947,1.687329881\H,0.5322749482,-3.7263727034,2.0010
763486\H,1.1992696604,-2.1215236589,2.4368820723\H,1.496403245,-1.4981
340663,-1.3393214315\H,2.5942381388,-0.5497915257,1.2068643188\C,-1.35
20924833,-1.8189190237,1.1841038365\C,-2.6658000682,-1.4497957298,0.93
16226365\C,-3.1392472184,-1.3507145074,-0.3809900279\C,-2.2832770914,-
1.6582446968,-1.4411398945\C,-0.9689096594,-2.0468063427,-1.1807312219
\H,-1.0022905573,-1.8527374693,2.209850922\H,-3.3407390039,-1.21903715
09,1.7509957332\O,-4.4352980787,-0.9663221794,-0.5252314283\H,-2.62451
0347,-1.6177656424,-2.4694719103\H,-0.3326239788,-2.3182266222,-2.0183
825148\C,-4.9380186947,-0.82681353,-1.8436869706\H,-5.9695468577,-0.49
01463222,-1.7368097971\H,-4.3667957206,-0.0789870786,-2.4060765654\H,-
4.9212436866,-1.7847634257,-2.3764668806\\Version=ES64L-G16RevA.03\Sta
te=1-A\HF=-1276.5739335\RMSD=7.740e-09\RMSF=3.138e-06\Dipole=0.4821019
,-2.9412082,-0.8084157\Quadrupole=1.8101028,-1.1091342,-0.7009686,-8.3
143507,8.8795471,-0.0647513\PG=C01 [X(C19H21N3O5)]\\@

34 References

1. N. Mallo, P. T. Brown, H. Iranmanesh, T. S. C. MacDonald, M. J. Teusner, J. B. Harper, G. E. Ball and J. E. Beves, *Chem. Commun.*, 2016, **52**, 13576-13579.
2. S. Helmy, F. A. Leibfarth, S. Oh, J. E. Poelma, C. J. Hawker and J. Read de Alaniz, *J. Am. Chem. Soc.*, 2014, **136**, 8169-8172.
3. M. M. Lerch, S. J. Wezenberg, W. Szymanski and B. L. Feringa, *J. Am. Chem. Soc.*, 2016, **138**, 6344-6347.
4. M. M. Lerch, M. J. Hansen, W. A. Velema, W. Szymanski and B. L. Feringa, *Nat. Commun.*, 2016, **7**, 12054.
5. M. M. Lerch, M. Medved, A. Lapini, A. D. Laurent, A. Iagatti, L. Bussotti, W. Szymanski, W. J. Buma, P. Foggi, M. Di Donato and B. L. Feringa, *J. Phys. Chem. A*, 2018, **122**, 955-964.
6. N. Isaacs, *Physical Organic Chemistry*, Longman Group, Harlow, UK, 2nd edn., 1995.
7. Sheldrick, G., *SADABS*, University of Göttingen, Germany, 1996.
8. APEX3, *Bruker AXS Inc.,* Madison, Wisconsin, USA, 2016.
9. G. Sheldrick, *Acta Crystallograph. Sect. A*, 2015, **71**, 3-8.
10. O. V. Dolomanov, L. J. Bourhis, R. J. Gildea, J. A. K. Howard and H. Puschmann, *J. Appl. Crystallogr.*, 2009, **42**, 339-341.
11. N. P. Cowieson, D. Aragao, M. Clift, D. J. Ericsson, C. Gee, S. J. Harrop, N. Mudie, S. Panjikar, J. R. Price, A. Riboldi-Tunnicliffe, R. Williamson and T. Caradoc-Davies, *J. Synchrotron Rad.*, 2015, **22**, 187-190.
12. W. Kabsch, *Acta Crystallograph. Sect. D*, 2010, **66**, 125-132.
13. A. Spek, *J. Appl. Crystallogr.*, 2003, **36**, 7-13.
14. S. Helmy, S. Oh, F. A. Leibfarth, C. J. Hawker and J. Read de Alaniz, *J. Org. Chem.*, 2014, **79**, 11316-11329.
15. R. B. Gaussian 16, Frisch, M. J.; Trucks, G. W.; Schlegel, H. B.; Scuseria, G. E.; Robb, M. A.; Cheeseman, J. R.; Scalmani, G.; Barone, V.; Petersson, G. A.; Nakatsuji, H.; Li, X.; Caricato, M.; Marenich, A. V.; Bloino, J.; Janesko, B. G.; Gomperts, R.; Mennucci, B.; Hratchian, H. P.; Ortiz, J. V.; Izmaylov, A. F.; Sonnenberg, J. L.; Williams-Young, D.; Ding, F.; Lipparini, F.; Egidi, F.; Goings, J.; Peng, B.; Petrone, A.; Henderson, T.; Ranasinghe, D.; Zakrzewski, V. G.; Gao, J.; Rega, N.; Zheng, G.; Liang, W.; Hada, M.; Ehara, M.; Toyota, K.; Fukuda, R.; Hasegawa, J.; Ishida, M.; Nakajima, T.; Honda, Y.; Kitao, O.; Nakai, H.; Vreven, T.; Throssell, K.; Montgomery, J. A., Jr.; Peralta, J. E.; Ogliaro, F.; Bearpark, M. J.; Heyd, J. J.; Brothers, E. N.; Kudin, K. N.; Staroverov, V. N.; Keith, T. A.; Kobayashi, R.; Normand, J.; Raghavachari, K.; Rendell, A. P.; Burant, J. C.; Iyengar, S. S.; Tomasi, J.; Cossi, M.; Millam, J. M.; Klene, M.; Adamo, C.; Cammi, R.; Ochterski, J. W.; Martin, R. L.; Morokuma, K.; Farkas, O.; Foresman, J. B.; Fox, D. J. Gaussian, Inc., Wallingford CT, 2016., *Journal*.
16. Y. Zhao and D. G. Truhlar, *Theor. Chem. Acc.*, 2008, **120**, 215-241.
17. A. V. Marenich, C. J. Cramer and D. G. Truhlar, *J. Phys. Chem. B*, 2009, **113**, 6378-6396.
18. J. Ho and M. Z. Ertem, *J. Phys. Chem. B*, 2016, **120**, 1319-1329.
19. D. J. Henry, M. B. Sullivan and L. Radom, *J. Chem. Phys.*, 2003, **118**, 4849-4860.
20. E. D. Raczyńska, M. K. Cyrański , M. Gutowski, J. Rak, J. F. Gal, P. C. Maria, M. Darowska and K. Duczmal, *J. Phys. Org. Chem.*, 2003, **16**, 91-106.
21. D. H. Aue, H. M. Webb and M. T. Bowers, *J. Am. Chem. Soc.*, 1976, **98**, 318-329.
22. T. Rodima, I. Kaljurand, A. Pihl, V. Mäemets, I. Leito and I. A. Koppel, *J. Org. Chem.*, 2002, **67**, 1873-1881.
23. E.-I. Rõõm, A. Kütt, I. Kaljurand, I. Koppel, I. Leito, I. A. Koppel, M. Mishima, K. Goto and Y. Miyahara, *Chem.-Eur. J.*, 2007, **13**, 7631-7643.
